# The Role of Non‐Covalent Interactions on Cluster Formation: Pentamer, Hexamers and Heptamer of Difluoromethane

**DOI:** 10.1002/anie.202103900

**Published:** 2021-06-24

**Authors:** Camilla Calabrese, Berhane Temelso, Imanol Usabiaga, Nathan A. Seifert, Francisco J. Basterretxea, Giacomo Prampolini, George C. Shields, Brooks H. Pate, Luca Evangelisti, Emilio J. Cocinero

**Affiliations:** ^1^ Departament of Physical Chemistry University of the Basque Country (UPV/EHU) Barrio Sarriena, S/N 48940 Leioa Spain; ^2^ Instituto Biofisika (UPV/EHU, CSIC) 48940 Leioa Spain; ^3^ Fundación Biofísica Bizkaia/Biofisika Bizkaia Fundazioa (FBB) 48940 Leioa Spain; ^4^ George Mason University Information Technology Services Fairfax VA 22030 USA; ^5^ Dipartimento di Chimica “Giacomo Ciamician” Università degli Studi di Bologna via S. Alberto 163 48100 Ravenna Italy; ^6^ Department of Chemistry University of Virginia McCormick Road Charlottesville VA 22903 USA; ^7^ Istituto di Chimica dei Composti OrganoMetallici (ICCOM-CNR) Area della Ricerca via G. Moruzzi 1 56124 Pisa Italy; ^8^ Department of Chemistry Furman University Greenville SC 29609 USA

**Keywords:** difluoromethane, molecular clusters, non covalent interactions, quantum chemical calculations, rotational spectroscopy

## Abstract

The role of non‐covalent interactions (NCIs) has broadened with the inclusion of new types of interactions and a *plethora* of weak donor/acceptor partners. This work illustrates the potential of chirped‐pulse Fourier transform microwave technique, which has revolutionized the field of rotational spectroscopy. In particular, it has been exploited to reveal the role of NCIs’ in the molecular self‐aggregation of difluoromethane where a pentamer, two hexamers and a heptamer were detected. The development of a new automated assignment program and a sophisticated computational screening protocol was essential for identifying the homoclusters in conditions of spectral congestion. The major role of dispersion forces leads to less directional interactions and more distorted structures than those found in polar clusters, although a detailed analysis demonstrates that the dominant interaction energy is the pairwise interaction. The tetramer cluster is identified as a structural unit in larger clusters, representing the maximum expression of bond between dimers.

## Graphical abstract

The size‐limit of cluster investigation is pushed forward in this high‐resolution gas phase rotational spectroscopy study. This is the first detection of a pentamer, two hexamers and one heptamer of difluoromethane, through a synergic work of theoretical protocols and advanced experiments. This intricate balance of weak non‐covalent interactions is analyzed in‐depth.

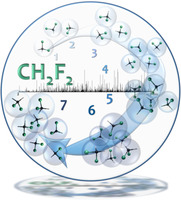

Non‐covalent interactions[^1^, ^2^, ^3^] (NCIs) play a key role in many fields and their understanding is pivotal in research areas as diverse as molecular biology, drug design,^[4]^ self‐assembly,^[5]^ crystal engineering^[6]^ and nano‐materials.^[7]^ NCIs’ ubiquity can be traced back to their capability to span a wide range of binding energies and a broad variety of structural patterns. In the past decade, theory and experiments have revealed a much wider collection of NCIs, shifting the focus to new interactions, which involve different weak donor/acceptor partners. In this context, the hydrogen bond (HB) definition was revised by IUPAC in 2011,^[8]^ whereas new NCIs classes, as π‐π,^[9]^ anion‐π,^[10]^ halogen bonding (XB),[^11^, ^12^, ^13^] chalcogen bonds,^[14]^ pnictogen bonds,^[15]^ tetrel bonding,^[16]^ or coinage‐metals^[17]^ began to be recognized and more precisely defined.

Experimental references on NCIs are abundant, mainly focused on the condensed and crystalline phases. However, both could be *biased* by the environment. In this context, *isolated studies in the gas‐phase* emerge as a powerful strategy to investigate and calibrate our understanding of these NCIs. A wide variety of laser‐based spectroscopy experiments has generated invaluable information, although they depend on the quality of the theoretical calculations and the assignments are not always conclusive. In this sense, rotational spectroscopy is emerging as a potent tool due to its unrivaled resolution (5–20 kHz≈10^−7^ cm^−1^). Although the technique is not new and was initially limited to very small systems, recent advances such as *chirped‐pulses*,^[18]^ *three‐wave mixing* ^[19]^ or *laser‐vaporization* ^[20]^ have revolutionized the field and greatly expanded its applicability. Hence, it is one of the most powerful approaches to study NCIs although it is still largely unknown to most of the scientific community.

In this work, the power of microwave spectroscopy, in particular the chirped‐pulse Fourier transform microwave (CP‐FTMW) technique, has been exploited to reveal the role of NCIs in molecular self‐aggregation or cluster formation.

This field is complex and the properties exhibited by weakly bound molecular aggregates help to bridge the gap between the different states of matter.[^21^, ^22^, ^23^, ^24^] The gas‐phase approach can facilitate the design and generation of tailor‐made molecular clusters to study the specific behavior of NCIs. However, despite their conceptual potential, in practice there are very few examples with molecular homoclusters larger than two units,[^25^, ^26^, ^27^, ^28^, ^29^, ^30^, ^31^, ^32^, ^33^] with the water pentadecamer (H_2_O)_15_ being the largest cluster observed rotationally to date.^[34]^ This cluster adopts a 3D geometrical shape stabilized by moderate O−H⋅⋅⋅O HBs.

In this project, we have focused on the self‐aggregation of a halocarbon (difluoromethane, or DFM hereafter) which may be stabilized by a subtle balance of C−H⋅⋅⋅F weak hydrogen bonds (WHBs), C−F⋅⋅⋅F XBs or F−C⋅⋅⋅F tetrel bonds, which were previously observed in (CH_2_F_2_)_*n*_⋅⋅⋅(H_2_O)_*m*_ (with *n*=1–2 and *m*=1–3).^[35]^

This project is not only an experimental challenge but also a theoretical one, where an accurate description of NCIs is still necessary. In fact, NCI's delicate balance makes it impossible to predict by chemical intuition the geometric shapes that these clusters will adopt. Each DFM molecule is equally likely to act as a donor or acceptor in single or bifurcated NCIs.^[36]^ Finally, the importance (or not) of cooperativity of NCIs still lacks reported examples.^[37]^

In the past two decades, a deeper insight into DFM clusters has been achieved by successfully combining quantum mechanical calculations and rotational spectroscopy.[^35^, ^38^] So far, the rotational studies on DFM have shown that the self‐aggregated structures of the smaller clusters (from the dimer to the tetramer) preserve planarity of the carbon framework, due to a forced rearrangement to maximize the attractive interactions (see upper part of Figure 1 and blue points of the diagram).[^38^, ^39^, ^40^, ^41^] The low number of units together with the small size of the molecule are not enough to create a repeatable geometric stability pattern.

**Figure 1:**
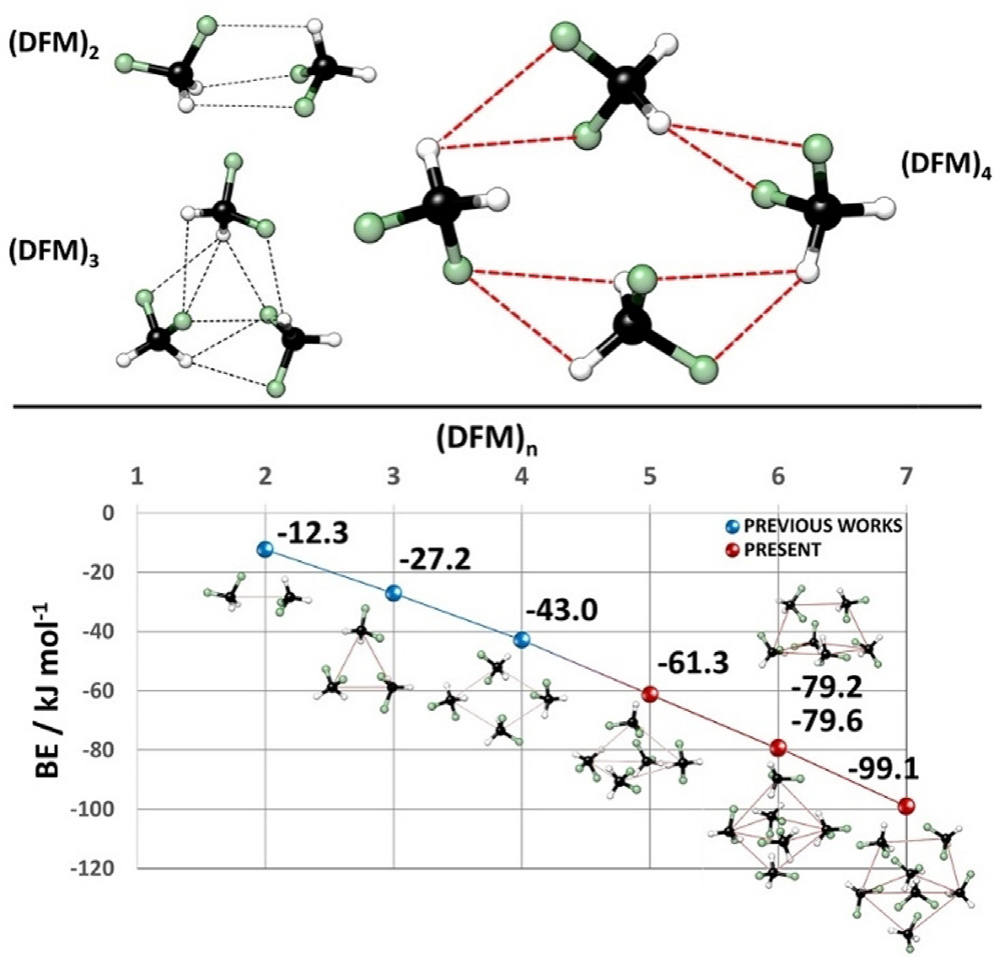
Geometries of previously observed DFM clusters. The structures and binding energy were calculated at the MP2/VTZ‐F12//MP2/aVDZ level of theory. For more details, please see Tables S8‐S11.

Exploiting broadband rotational spectroscopy, we were able to access larger clusters, with emergent 3D organized geometric structures and cooperative networks of weak NCIs (see Figure 1, red points). *In this work, we present the generation and detection of several higher clusters of DFM (one pentamer, two hexamers and one heptamer)*.

The experiments were first performed with a CP‐FTMW spectrometer covering the frequency range 2–8 GHz at the University of Virginia (see the obtained spectrum in Figure 2). Subsequently, a second experiment was performed with similar equipment at the University of the Basque Country, to expand the frequency range to 6–18 GHz. Experimental details as well as the operation principles have been described elsewhere^[42]^ and are also summarized in the SI.

**Figure 2:**
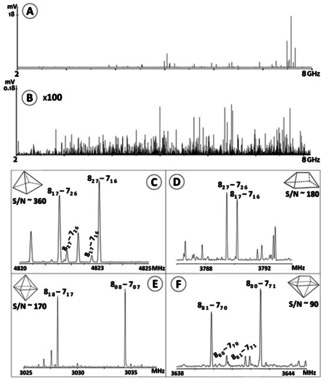
Experimental CP‐FTMW spectrum of DFM recorded in neon backing gas. Panel A shows the complete spectrum, the spectrum in panel B (on an expanded vertical scale, ×100) is the result after removing the already assigned species ((DFM)_*n*_, *n*=1–4). The four lower panels show portions of the assigned rotational transitions for each cluster detected (C: pentamer, D: hexamer‐I, E: hexamer‐II, F: heptamer). A rough value of the signal‐to‐noise (S/N) ratio is also given.

The treatment of the rotational spectra of these (DFM)_*n*_ clusters, with *n*=5–7, is a challenge, since these systems have very small rotational constants, which results in very congested and overlapping spectra of all the clusters. In summary, there are plenty of lines in all spectral regions and it is almost impossible to identify clear patterns of each cluster with traditional procedures. Hence, several automated methods had to be developed to analyze the data originated from the CP‐FTMW due to the abundance of observable transitions in the spectra.[^43^, ^44^, ^45^] As described in an interesting paper by Watson,^[46]^ asymptotic symmetric top limits are observed, producing in these large molecular systems regular 2*C* spacings, where *C* is the experimental rotational constant in the oblate limit.^[47]^ For this reason, the fit procedure which we developed was focused on the search for these regular spacings and it was implemented through a Python script.^[48]^ The program led to the experimental identification of the clusters automatically and the determination of the *C* rotational constants for the different DFM clusters a priori, *without the aid of theoretical rotational constants*. Correspondence between observed clusters and predicted structures was subsequently been carried out by high level ab initio calculations. In all cases, based on the rotational constants and the relative intensities of the three types of transitions, a unique structure could be assigned among the most stable predicted minima (see Tables S9–S11).

The final fits were done using the Watson's Hamiltonian with CALPGM. The values of the experimental rotational constants are reported in Table 1, while the complete set of spectroscopic parameters can be found in the SI.

**Table 1:** Experimental and theoretical rotational constants of the observed homoclusters of DFM, calculated at the MP2/VTZ‐F12//MP2/aVDZ level. The complete set of theoretical and experimental parameters can be found in SI (Tables S9–S11 and S1, respectively).

	PENTAMER [**5‐I**]	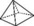	HEXAMER [**6‐I**]	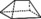
	Exp.	Theo.	Exp.	Theo.
A [MHz]^[a]^	426.61950(7)^[d]^	441	356.1116(7)	367
B [MHz]	358.42855(5)	375	211.52526(4)	225
C [MHz]	281.54415(5)	292	185.23390(5)	196
*σ*_fit_^[b]^ [kHz]	8.1		8.2	
N_lines_^[c]^	725		404

	HEXAMER [**6‐II**]	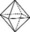	HEPTAMER [**7‐II**]	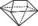
A [MHz]	304.72043(7)	324	229.5257(1)	233
B [MHz]	266.17778(5)	277	215.0008(1)	222
C [MHz]	226.35912(5)	237	163.3655(1)	162
*σ*_fit_ [kHz]	6.2		9.7	
N_lines_	462		571

[a] Rotational constants. [b] MW root‐mean‐square deviation of the fit. [c] Number of fitted transitions. [d] Error in parentheses in units of the last digit.

The combination of experimental and computational protocols allowed us to detect a pentamer, two hexamers and a heptamer for DFM. A careful analysis of all the intermolecular CH⋅⋅⋅F distances of the assigned clusters allowed us to rationalize their geometries. First, we dimensioned the significant interval of intermolecular NCIs able to describe cluster aggregation to 2.3 Å<CH⋅⋅⋅F≤2.9 Å, as smaller values go beyond typical intermolecular contacts, and larger values are less relevant. In fact, regardless of the size of the cluster (from the tetramer up), almost all intermolecular contacts of the CH⋅⋅⋅F type lie within this range, making them decisive for the formation of the complexes as also confirmed by applying the Atom in Molecules (AIM) theory (see Figure S13).^[49]^ Secondly, the types of C−H⋅⋅⋅F interactions characterizing DFM homoclusters can be divided into two families: single and bifurcated. The first characterizes the observed dimer (see Figure 1). The bifurcated C−H⋅⋅⋅F interactions also divide into two varieties: in the first, two C−H groups point towards a single fluorine atom, 2(C−H)⋅⋅⋅F, and in the second, a single C−H group links two different fluorine atoms, C−H⋅⋅⋅2(F). These bifurcated intermolecular interactions characterize the two (unobserved) dimers with energies above the (observed) global minimum, see Figure S2. All these types of interactions can coexist within each isolated cluster and give rise to its final structural arrangement, as we will see below.

Our analysis begins with the observed tetramer of DFM by Feng et al.,^[38]^ the first cluster with a non‐zero dipole moment by symmetry. It presents a total of 15 CH⋅⋅⋅F interactions inside the limiting range. In particular, 8 CH⋅⋅⋅F bifurcated interactions (that correspond to the ones shown in Figure 1) support its rhomboid cyclic structure, where each molecule acts simultaneously as both donor and acceptor of 2 CH⋅⋅⋅F intermolecular HBs. The remaining interactions (purple lines of Figure 3) elude the cyclic structure but reinforce the cohesion of the tetramer. Moreover, the four carbon atoms characterizing the tetramer are almost co‐planar, with a deviation of less than 20°. *The cyclic rhomboid structure of the tetramer acts as a scaffold for the higher clusters, which we use as a common structural reference point*.

**Figure 3:**
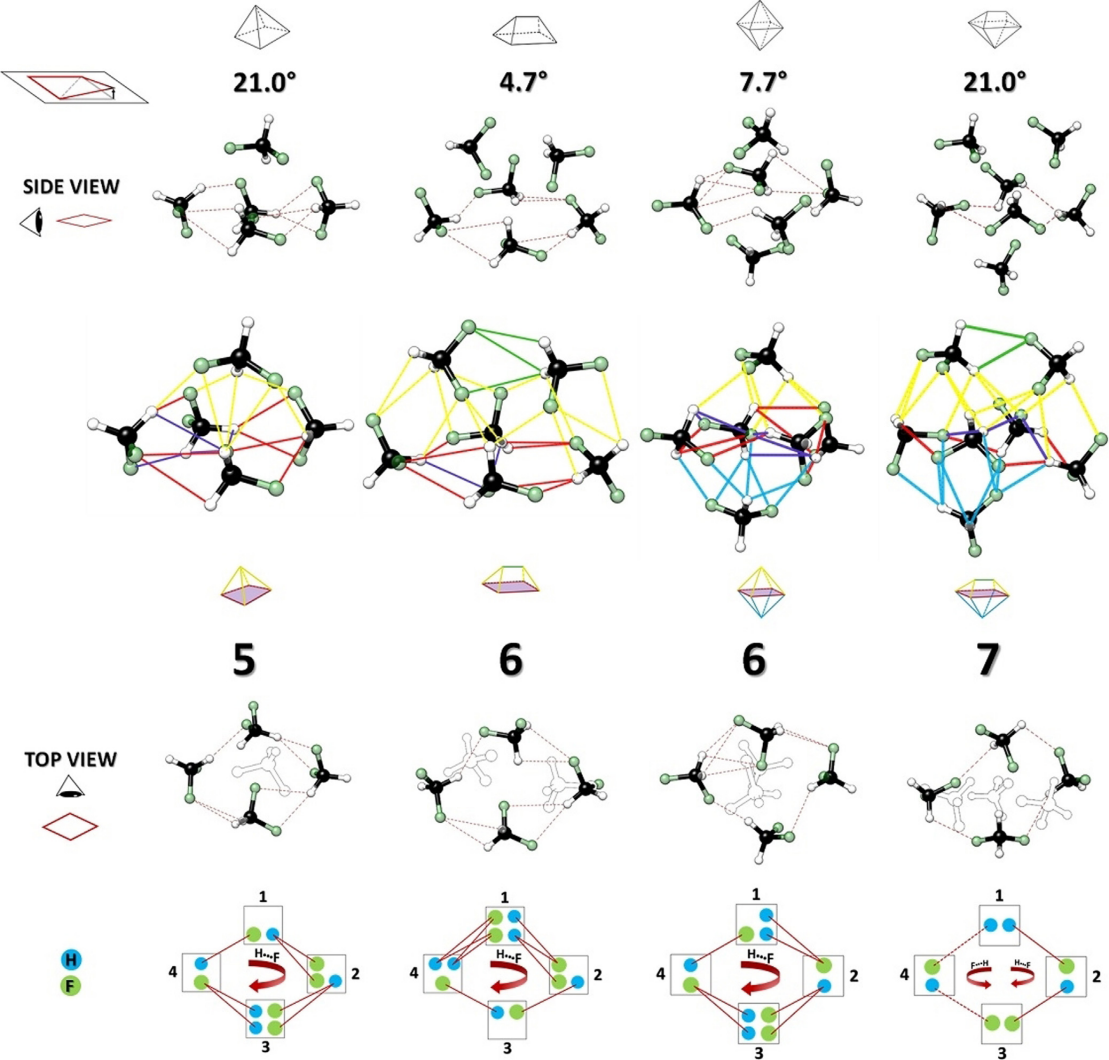
Different perspectives of the structures of the DFM clusters computed at MP2/VTZ‐F12 level of theory. The deviation out of the plane that identifies the tetramer scaffold is reported for each cluster (see text for more details). Five different categories of CH⋅⋅⋅F can be recognized which define the geometries of the clusters: (red lines) interactions belong to the rhomboid cyclic structure which resemble the tetramer one; (purple lines) other interactions inside the tetramer scaffold; (yellow lines) interactions of the upper vertices with the tetramer scaffold; (blue lines) CH⋅⋅⋅F contacts involving the lower vertices and the tetramer scaffold, and (green lines) interactions between the higher vertices that do not involve the rhomboid cyclic structure. The interactions taken into consideration fall in the range 2.3 Å<CH⋅⋅⋅F≤2.9 Å.

The pentamer of DFM (**5‐I**) is an oblique pyramid, where the tetramer scaffold represents the base (see Figure 3). There are 18 interactions where 2.3 Å<CH⋅⋅⋅F≤2.9 Å, 3 more than in the tetramer. They are distributed as follows: 11 involving the tetramer scaffold, 7 defining the rhomboid cyclic structure (red lines) and the remaining 4 lying within the tetrameric scaffold (purple lines). Moreover, another type of C−H⋅⋅⋅F interaction appears, which involves the upper vertex of the pyramid with the tetramer scaffold (7 yellow lines).

The hexamer was observed in two different isomeric forms, calculated to be nearly isoenergetic at the MP2/VTZ‐F12//MP2/aVDZ level (energy difference less than 0.5 kJ mol^−1^, see Table S10). The lowest energy structure (label: **6‐I,** see detail of the assignment in Figure S1) is a generic polyhedron, that in a less formal but more effective way for the purposes of the analysis, we can name as a *two‐vertex pyramid* (see structure in Figure 3). Once again, the tetramer scaffold provides the base of the structure above which lie the two remaining units of DFM, with a widened rhomboidic structure that allows insertion of the two additional subunits. There is now a total of 25 interactions, an expected increase due to the increasing of the cluster size. In particular, 10 tetrameric interactions are conserved (7 red and 2 purple lines); additionally, 13 CH⋅⋅⋅F interactions involve the two vertices and the tetramer base (yellow lines); and a new type of contact which implicates only the higher vertices (3 green lines).

The second observed hexamer (label: **6‐II**) is an *octahedron*. This is the only other hexameric arrangement that conserves the tetrameric scaffold, where the two remaining DFM moieties lie above and below the plane of the tetramer, forming the two vertices of the *octahedron*. As with the other hexamer, the tetramer scaffold widens, but less so in this case with only one molecule accommodated on each side, with the same number of 25 CH⋅⋅⋅F contacts but now distributed differently. In particular, 7 of the interactions sustain the rhomboid cyclic structure (red lines); 5 lie inside the tetramer scaffold (purple lines); 6 (yellow lines) and 7 (blue lines) connect the upper and lower vertices with the tetramer base, respectively.

Finally, a heptamer (**7‐II**) could be assigned to the second predicted lowest energy structure (see Tables S1 and S11), almost isoenergetic with the global minimum (about 0.3 kJ mol^−1^). It combines the two hexameric structures, hence it incorporates a tetramer scaffold, capped with two molecules above and one below. There are now 30 CH⋅⋅⋅F interactions: 7 lie within the tetramer scaffold (4 red and 3 purple lines, see Figure 3); 13 lie above it (yellow lines) with 2 more linking the pair of capping DFM molecules (green lines, cf. the structure of **6‐I**); and 8 (blue lines) bind the single molecule at the base.

Focusing on the tetramer scaffold, the degrees of deviation from the unique near co‐planar structure defined by three of the carbon atoms, with the fourth minimizing the deviation, are reported in Figure 3 for all the larger clusters. The values for the pentamer and heptamer clusters (21°), are very close to that of the isolated tetramer (18.5°) while the hexamer structures deviate rather less (<8°). The maximum C−C distance (6.10 Å) of the rhomboid in the tetramer is also maintained in the pentamer and the *octahedral* hexamer (6.14 Å and 6.03 Å, respectively), but in the two capped structures the distance increases to 6.76 Å (**6‐I**) and 6.51 Å (**7‐II**). The rhomboid cyclic structure is sustained by CH→F bifurcated interactions that follow a clockwise direction for all the clusters except for the heptamer, where the generalized increase in contacts further diversifies the type and orientation of interactions (see lower part of Figure 3 and Sketch 1 in SI).

In general, it can be noted that each DFM molecule acts simultaneously as both donor and acceptor, maximizing the network of contacts. As expected, there is an increase in the number of interactions as cluster size increases (tetramer → 15, pentamer → 18, hexamers → 25, heptamer → 30). But this growth of cooperative networks is also enough to raise the trend in the number of interactions per unit of DFM (tetramer → 3.8, pentamer → 3.6, hexamers → 4.2, heptamer → 4.3). All these data go hand in hand with the increase in binding energy (see Figure 1) through weak C−H⋅⋅⋅F interactions.

Beyond understanding the structures of the observed clusters, it is also possible to deduce further insights into their bonding by exploring the interaction between monomers in a many‐body decomposition (MBD)^[50]^ analysis of the interaction energy. In MBD, the total interaction energy is split into one‐body (1b, monomer relaxation), two‐body (2b, pairwise interactions), and cooperative or anti‐cooperative many‐body interactions (three‐body, four‐body, etc). This analysis is described in detail in Section 2.3 of the SI. MBD analysis of DFM clusters indicates that the most dominant interaction in these clusters is the two‐body or pairwise interaction between all possible dimers in the clusters. Each monomer distorts to optimize its pairwise interactions with its neighbours. These pairwise two‐body interactions can be very strong (8–12 kJ mol^−1^), weak (4–8 kJ mol^−1^) or very weak (<4 kJ mol^−1^) in magnitude, as illustrated in Figure S10, and interestingly this distribution is independent of the size of the cluster. The fact that the isolated dimer itself has many low‐energy minima and transition states (see Figure S11) explains the presence of these various pairwise interactions and confers vast conformational flexibility to the larger clusters. This fact is supported by SAPT analysis results (see section 2.4 of SI). In fact, while DFM has a permanent dipole moment and large electron density around the fluorine atom, the electrostatic component in DFM dimer is about half that of water dimer. On the other hand, the dispersion component is larger, and the increased importance of dispersion leads to less directional interaction and more distorted structures. Three‐ and higher‐body interactions are very small, generally accounting for less than 1 kJ mol^−1^ or 2 % of the total interaction energy, but they are indeed cooperative. In contrast to DFM clusters, cooperative many‐body interactions in water clusters account for about 20 % of the total interaction energy (see Figures S8 and S9).^[51]^ This difference in the role of cooperative many‐body interactions between DFM and water clusters can partly be attributed to the fact that dispersion is more important in DFM clusters than in water clusters and many‐body dispersion is smaller or negligible.^[52]^

Our interpretation of the geometric analysis discussed earlier agrees with these MBD calculations since the tetramer scaffold is the ideal arrangement for optimizing interactions between pairs of dimers, which represent the most relevant energy component as shown by the MBD. These analyses suggest that very small interactions can lead to the preservation of distinct structural features as clusters grow. These results can be useful to clarify the chemical and physical behaviour of new fluorinated compounds such as DFM and to try to extrapolate their properties in the bulk.

In summary, the combination of microwave spectroscopy, in particular CP‐FTMW spectrometers, with sophisticated computational screening protocols has allowed the observation of larger oligomeric clusters, four different homoclusters of DFM (*a pentamer, two hexamers and a heptamer*) were detected and analysed. The study shows the importance of NCIs where DFM act as both donor and proton acceptor. As expected, the number of NCIs increases with the size of the cluster, with the tetramer being the structural core in all of them, in order to maximize the stabilizing effects of the most dominant pairwise interactions.

The ability of rotational spectroscopy to access the structures of large, isolated molecular aggregates through the application of the CP‐FTMW technique demonstrates its unique power and future potential: it represents a remarkable technological breakthrough. The present work proves the successful synergy of rotational spectroscopy and computational modelling in enabling an in‐depth study of a large cluster characterized only by London dispersion interactions. These types of tailor‐made studies of NCIs represent a new challenge in structural analysis, which is important for understanding the behaviour of weak NCIs and potential applications in areas such as supramolecular engineering or biological molecular interactions.

## Conflict of interest

The authors declare no conflict of interest.

## Supporting information

As a service to our authors and readers, this journal provides supporting information supplied by the authors. Such materials are peer reviewed and may be re‐organized for online delivery, but are not copy‐edited or typeset. Technical support issues arising from supporting information (other than missing files) should be addressed to the authors.

Supplementary
