# Supplementary material for: The Role of Non‐Covalent Interactions on Cluster Formation: Pentamer, Hexamers and Heptamer of Difluoromethane
Source: Angew Chem Int Ed Engl. 2021 Jun 24;60(31):16894–9. doi: 10.1002/anie.202103900 (PMC8362204; doi:10.1002/anie.202103900)
Supplement: Supplementary file 1 — Supplementary [file ANIE-60-16894-s001.pdf]

Angewandte Chemie
Eine Zeitschrift der Gesellschaft Deutscher Chemiker

Supporting Information

# The Role of Non-Covalent Interactions on Cluster Formation: Pentamer, Hexamers and Heptamer of Difluoromethane

*Camilla Calabrese, Berhane Temelso, Imanol Usabiaga, Nathan A. Seifert, Francisco J. Basterretxea, Giacomo Prampolini, George C. Shields, Brooks H. Pate,* Luca Evangelisti,* and Emilio J. Cocinero**

anie_202103900_sm_miscellaneous_information.pdf

# Table of Contents

List of Figures ........ 3
List of Tables ........ 4
1 Experimental Results ........ 5
1.1 Experimental Details ........ 5
1.2 Fit results ........ 5
1.3 Structural Analysis ........ 7
2 Computations ........ 13
2.1 Structure Determination ........ 13
2.2 Rotational Constants, Principal Dipole Moments and Energies ........ 14
2.3 Many-Body Decomposition of Cluster Interaction Energies ........ 18
2.5 Cartesian Coordinates ........ 37
2.6 Quantitative analysis for difluoromethane tetramer ........ 44
References ........ 45

At the end of the file, the complete set of rotational transitions for all the DFM clusters observed are provided as separated files, in the following order: 5-I, 6-I, 6-II, 7-II.

# List of Figures

Figure S1. Observed $\mu_b$-transitions belonging to cluster 6-I but not included in the fit due to their low S/N ratio....6
Figure S2. The three lowest energy isomers of $(CH_2F_2)_2$. The MBD of the interaction energy of (I) is reported in the table below..........20
Figure S3.The lowest energy isomers of $(CH_2F_2)_3$. The carbon labels (C1,C2,C3) correspond to the monomer/fragment numbers in the table below..........21
Figure S4. The third lowest energy isomer of $(CH_2F_2)_4$. The carbon labels (C1,C2, …) correspond to the monomer/fragment numbers in the table below..........22
Figure S5.The lowest energy isomers of $(CH_2F_2)_5$. The carbon labels (C1,C2,C3) correspond to the monomer/fragment numbers in the table below..........24
Figure S6.The two lowest energy isomers of $(CH_2F_2)_6$. The carbon labels (C1,C2,C3) correspond to the monomer/fragment numbers in the table below..........26
Figure S7.The lowest energy isomers of $(CH_2F_2)_7$. The carbon labels (C1,C2,C3) correspond to the monomer/fragment numbers in the table below..........28
Figure S8. N-body contributions to the total interaction of $(CH_2F_2)_n$, n=2-7. 2-body interactions are dominant while larger N-body contributions are essentially zero..........30
Figure S9. N-body contributions to the total interaction of $(H_2O)_n$, n=2-7. While 2-body interactions account for about 75% of the total interaction energy, 3-body interactions are significant (~20%) as well..........31
Figure S10. The distribution of the two-body interaction energies in the $(CH_2F_2)_{n=2-7}$ clusters investigated in this study. The figures show a histogram (top) or shadow histogram fitted to a sum of three normal distribution functions (bottom)..........32
Figure S11. The interaction energy of many low energy minima and first-order transition states of difluoromethane dimer..........33
Figure S12. SAPT2+3/aVDZ interaction energy components (top) as a percentage of the total interaction energy (bottom)..........35
Figure S13. Quantitative analysis of attractive and repulsive interactions using the QTAIM procedure and NCI iso-surfaces. The carbon-carbon interaction distances are also reported. Blue- and green-colored areas indicate the presence of strong and weak attractive interactions, respectively. Red color signifies repulsive interactions. Bond-critical-point (BCP) paths are represented as blue lines. The iso-surface of the NCI analysis was build using s=0.5 and a blue-green-red color scale from $-0.02 < \mathrm{sign}(\lambda_2)\,\rho(r) < +0.01$ a.u...........44

# List of Tables

Table S1. Spectroscopic parameters obtained for the DFM clusters observed. ........................................................5
Table S2. List of the 2.3 Å < CH···F ≤ 2.9 Å for the 4-III structure..........................................................................8
Table S3. List of the 2.3 Å < CH···F ≤ 2.9 Å for the 5-I structure .............................................................................9
Table S4. List of the 2.3 Å < CH···F ≤ 2.9 Å for the 6-I structure ...........................................................................10
Table S5. List of the 2.3 Å < CH···F ≤ 2.9 Å for the 6-II structure.........................................................................11
Table S6. List of the 2.3 Å < CH···F ≤ 2.9 Å for the 7-II structure.........................................................................12
Table S7.Details of GA runs for $(CH_2F_2)_n$, where n = 2 − 7 .........................................................................13
Table S8. MP2/aVDZ rotational constants, principal dipole moments and MP2- F12/VTZ-F12//MP2/aVDZ relative energies of low energy isomers of $(CH_2F_2)_n$, where n = 2 − 4.............................................................14
Table S9. MP2/aVDZ rotational constants, principal dipole moments and MP2- F12/VTZ-F12//MP2/aVDZ relative energies of low energy isomers of $(CH_2F_2)_n$, where n = 5.................................................................15
Table S10. MP2/aVDZ rotational constants, principal dipole moments and MP2- F12/VTZ-F12//MP2/aVDZ relative energies of low energy isomers of $(CH_2F_2)_n$, where n = 6.................................................................16
Table S11. MP2/aVDZ rotational constants, principal dipole moments and MP2- F12/VTZ-F12//MP2/aVDZ relative energies of low energy isomers of $(CH_2F_2)_n$, where n = 7.................................................................17
Table S12. Many-body decomposition of the MP2-F12/VTZ-F12//MP2/aVDZ binding energy of $(CH_2F_2)_2$-I......20
Table 13. Many-body decomposition of the MP2-F12/VTZ-F12//MP2/aVDZ binding energy of $(CH_2F_2)_3$-I........21
Table S14. Many-body decomposition of the MP2-F12/VTZ-F12//MP2/aVDZ binding energy of $(CH_2F_2)_4$-III...23
Table S15. Many-body decomposition of the MP2-F12/VTZ-F12//MP2/aVDZ binding energy of $(CH_2F_2)_5$-I......25
Table S16. Many-body decomposition of the MP2-F12/VTZ-F12//MP2/aVDZ binding energy of $(CH_2F_2)_6$-I and $(CH_2F_2)_6$-II..........................................................................................................................................27
Table S17. Many-body decomposition of the MP2-F12/VTZ-F12//MP2/aVDZ binding energy of $(CH_2F_2)_7$-II ....28
Table S18. N-body contribution to the interaction energy as a fraction of the total interaction energy (E_nb/E_total) ..........................................................................................................................................30
Table S19. SAPT2+3/aVDZ interaction energy components of three prototypical dimers ....................................34
Table S20. MP2/aVDZ optimized Cartesian coordinates of DFM ($CH_2F_2$) monomer ...........................................37
Table S21. MP2/aVDZ optimized Cartesian coordinates of $(CH_2F_2)_2$.....................................................................37
Table S22. MP2/aVDZ optimized Cartesian coordinates of $(CH_2F_2)_3$.....................................................................38
Table S23. MP2/aVDZ optimized Cartesian coordinates of $(CH_2F_2)_4$.....................................................................39
Table S24. MP2/aVDZ optimized Cartesian coordinates of $(CH_2F_2)_5$-I .................................................................40
Table S25. MP2/aVDZ optimized Cartesian coordinates of $(CH_2F_2)_6$-I .................................................................41
Table S26. MP2/aVDZ optimized Cartesian coordinates of $(CH_2F_2)_6$-II ................................................................42
Table S27. MP2/aVDZ optimized Cartesian coordinates of $(CH_2F_2)_7$-II ................................................................43

# 1 Experimental Results

## 1.1 Experimental Details

The microwave spectra of DFM were registered in Pate's group at the University of Virginia using a CP-FTMW spectrometer, which covers the frequency ranges 2–8 GHz, while the upper spectra were measured by means of the CP-FTMW in Cocinero's group at the University of the Basque Country (UPV/EHU), which covers the frequency range 6-18 GHz. Both equipment were already described in details elsewhere.[1–3] The experiment in Virginia was carried out using 5 nozzles at the same time, and applying 8 chirps for each molecular pulse. On the other hand, the Bilbao's experiment was performed with 3 nozzles at the same time, and using 20 chirps for each molecular pulse. For the first spectrum, the number of final acquisitions is 3 million and 200 thousands, while for the second one registered in Bilbao it is 5 million.
The DFM sample was obtained from Sigma Aldrich. In both experiments, a gas mixture of ~1% $CH_2F_2$ in Ne was used at a backing pressure of 350 kPa, and then adiabatically expanded into the spectrometer by a few hundred μs duration pulses.

## 1.2 Fit results

The final fits were performed using Pickett's program, and all rotational frequencies were fitted with a Watson *S*-reduced Hamiltonian in the $I^r$ representation.[4] The errors in parenthesis are in units of the last digit. $\sigma$ is the root-mean-square deviation of the fit. *N* is the number of distinct frequency lines in the fit. The complete set of rotational transitions together with the fit results are given at the end of this Supporting Information file.

Table S1. Spectroscopic parameters obtained for the DFM clusters observed.

| | **5-I** | **6-I** | **6-II** | **7-II** |
|---|---|---|---|---|
| *A*/MHz | 426.61950(7) | 356.1116(7) | 304.72043(7) | 229.5257(1) |
| *B*/MHz | 358.42855(5) | 211.52526(4) | 266.17778(5) | 215.0008(1) |
| *C*/MHz | 281.54415(5) | 185.23390(5) | 226.35912(5) | 163.3655(1) |
| $D_J$/kHz | 0.0961(1) | 0.02118(5) | 0.0207(1) | 0.0084(3) |
| $D_{JK}$/kHz | -0.0405(7) | 0.0135(4) | 0.0580(5) | 0.068(1) |
| $D_K$ /kHz | 0.0706(6) | 0.33(2) | -0.0374(5) | -0.055(1) |
| $d_1$/kHz | -0.02690(5) | [0.][a] | -0.00175(5) | -0.0028(1) |
| $d_2$/kHz | -0.00306(3) | -0.00076(3) | -0.00173(3) | -0.00318(8) |
| $\mu_a$ | weak | strong | medium | medium |
| $\mu_b$ | strong | weak | - | strong |
| $\mu_c$ | medium | - | weak | weak |
| *N* | 474 | 328 | 385 | 348 |
| $\sigma$ /kHz | 8.1 | 8.2 | 6.2 | 9.7 |

[a] Fixed to zero.

### 1.2.1 6-I Details of Assignment

The assignment of cluster 6-I is based not only on the values of the rotational constants and on its maximum energy stability, but also on the type of lines observed in the spectrum. To avoid possible assignment doubts with the 6-VI isomer (see the following figures and tables), the very few observed $\mu_b$-lines belonging to 6-I are reported. $\mu_b$-lines are present in the spectrum (see the following picture) since predicted more than 20 times weaker with respect to the $\mu_a$, but their intensity touches the limit of detections, so even if their S/N is much less than 4 (the threshold chosen by the authors to measure lines), the authors decided to include them anyway. On the other hand, the $\mu_b$-lines of the 6-VI cluster are predicted more than 300 times weaker, so for sure not detectable at all. Moreover, the $\mu_c$-lines of 6-VI were not observed and they should be stronger that $\mu_b$ and only 38 times weaker than $\mu_a$ -lines observed of 6-I. All these considerations support the 6-I assignment.

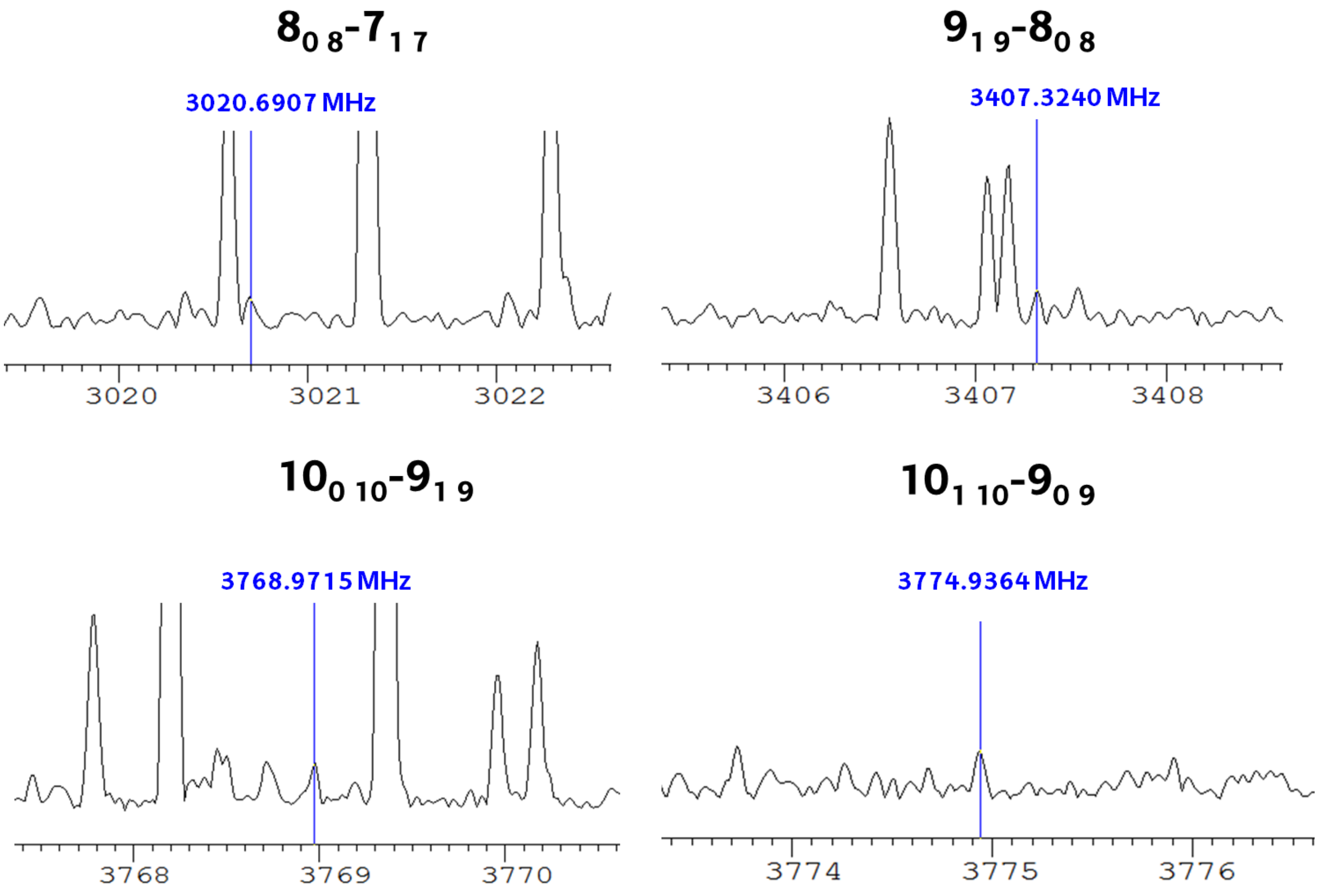


Figure S1. Observed $\mu_b$-transitions belonging to cluster 6-I but not included in the fit due to their low S/N ratio.

## 1.3 Structural Analysis

For each observed structure for (DFM)n, with n ≥ 4, the analysis of the WHB was performed taking into account the 2.3 Å < CH···F ≤ 2.9 Å range. The following tables contain the complete list of intermolecular interactions for each cluster. The structures of the DFM clusters were computed at MP2/VTZ-F12 level of theory. The color code of the CH···F interactions follow the criteria described in the paper: (red lines) interactions belong to the rhomboid circular structure which resemble the tetramer one; (purple lines) other interactions inside the tetramer scaffold; (yellow lines) interactions of the upper vertices with the tetramer scaffold; (blue lines) CH···F contacts involving the lower vertices and the tetramer scaffold, and (green lines) interactions between the higher vertices that do not involve the rhomboid circular structure.

Moreover, in the following sketch, intuitive schemes of the principal CH···F interactions involved in the tetramer scaffold are shown.

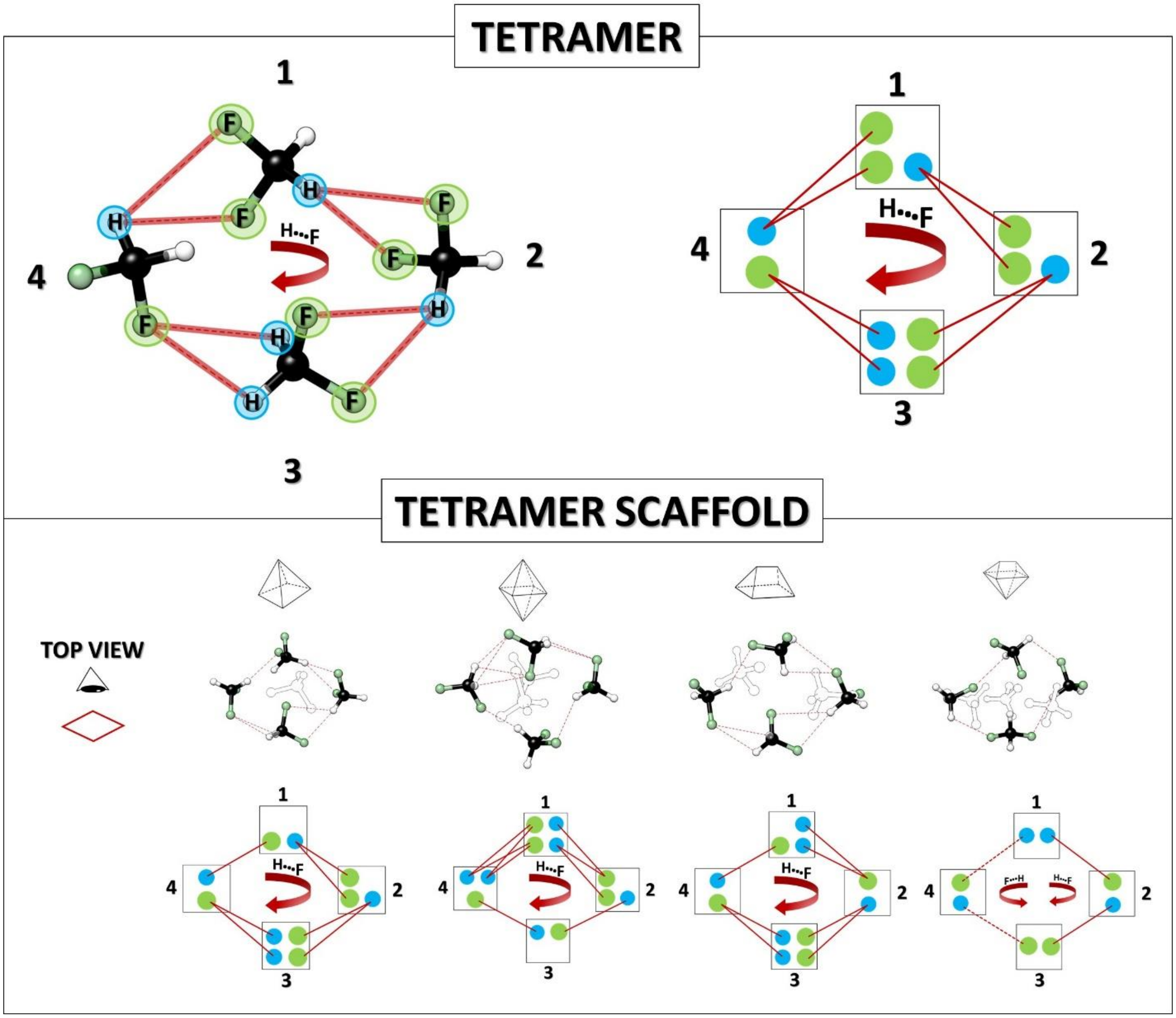


Sketch 1: The interactions characterizing the tetramer (upper panel) are schematized in order to make them more identifiable in the larger clusters (lower panel).

Table S2. List of the 2.3 Å < CH···F ≤ 2.9 Å for the 4-III structure

**4-III**

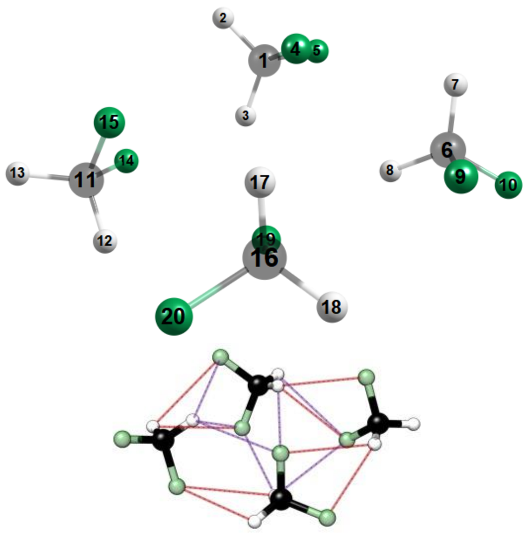


| F | H | Å |
|---|---|---|
| 20 | 12 | 2.459 |
| 19 | 3 | 2.502 |
| 4 | 17 | 2.520 |
| 14 | 3 | 2.539 |
| 9 | 18 | 2.557 |
| 19 | 8 | 2.607 |
| 19 | 12 | 2.661 |
| 15 | 3 | 2.671 |
| 15 | 17 | 2.761 |
| 4 | 7 | 2.768 |
| 4 | 8 | 2.848 |
| 9 | 17 | 2.851 |
| 5 | 8 | 2.868 |
| 5 | 7 | 2.881 |
| 15 | 2 | 2.986 |

Table S3. List of the 2.3 Å < CH···F ≤ 2.9 Å for the 5-I structure

**5-I**

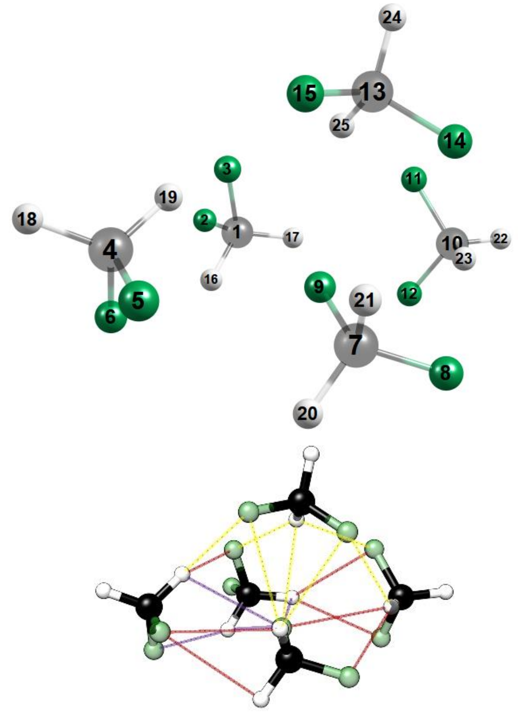


| F | H | Å |
|---|---|---|
| 3 | 25 | 2.375 |
| 8 | 23 | 2.408 |
| 3 | 19 | 2.434 |
| 11 | 25 | 2.441 |
| 6 | 16 | 2.464 |
| 15 | 19 | 2.477 |
| 12 | 17 | 2.499 |
| 14 | 23 | 2.564 |
| 9 | 25 | 2.572 |
| 11 | 17 | 2.575 |
| 9 | 23 | 2.611 |
| 9 | 19 | 2.619 |
| 14 | 21 | 2.687 |
| 9 | 16 | 2.690 |
| 5 | 21 | 2.690 |
| 5 | 20 | 2.718 |
| 15 | 21 | 2.736 |
| 9 | 17 | 2.880 |

Table S4. List of the 2.3 Å < CH···F ≤ 2.9 Å for the 6-I structure

**6-I**

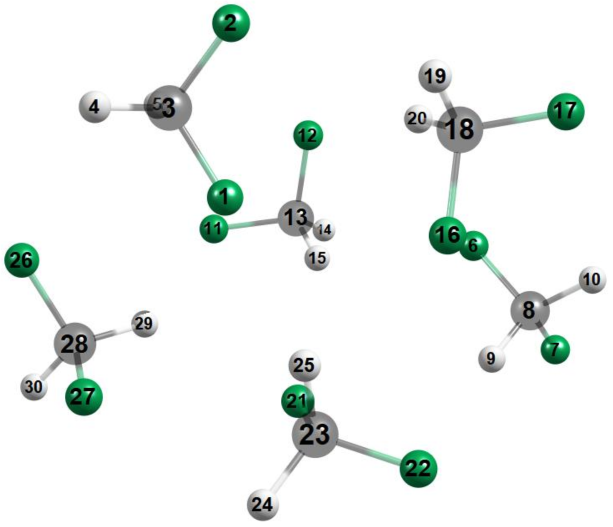


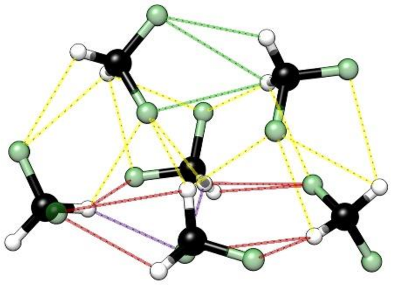

| F | H | Å | F | H | Å |
|---|---|---|---|---|---|
| 21 | 15 | 2.333 | 27 | 24 | 2.655 |
| 11 | 29 | 2.367 | 6 | 20 | 2.686 |
| 12 | 20 | 2.376 | 2 | 19 | 2.704 |
| 16 | 25 | 2.455 | 17 | 10 | 2.734 |
| 22 | 9 | 2.471 | 11 | 5 | 2.737 |
| 1 | 25 | 2.509 | 6 | 14 | 2.776 |
| 6 | 15 | 2.524 | 27 | 25 | 2.892 |
| 21 | 29 | 2.544 | 16 | 10 | 2.894 |
| 1 | 15 | 2.562 | 26 | 5 | 2.901 |
| 26 | 4 | 2.612 | 21 | 9 | 2.909 |
| 1 | 29 | 2.621 | 2 | 20 | 2.957 |
| 16 | 9 | 2.628 | 1 | 20 | 2.961 |
| 12 | 5 | 2.655 | | | |

Table S5. List of the 2.3 Å < CH···F ≤ 2.9 Å for the 6-II structure

**6-II**

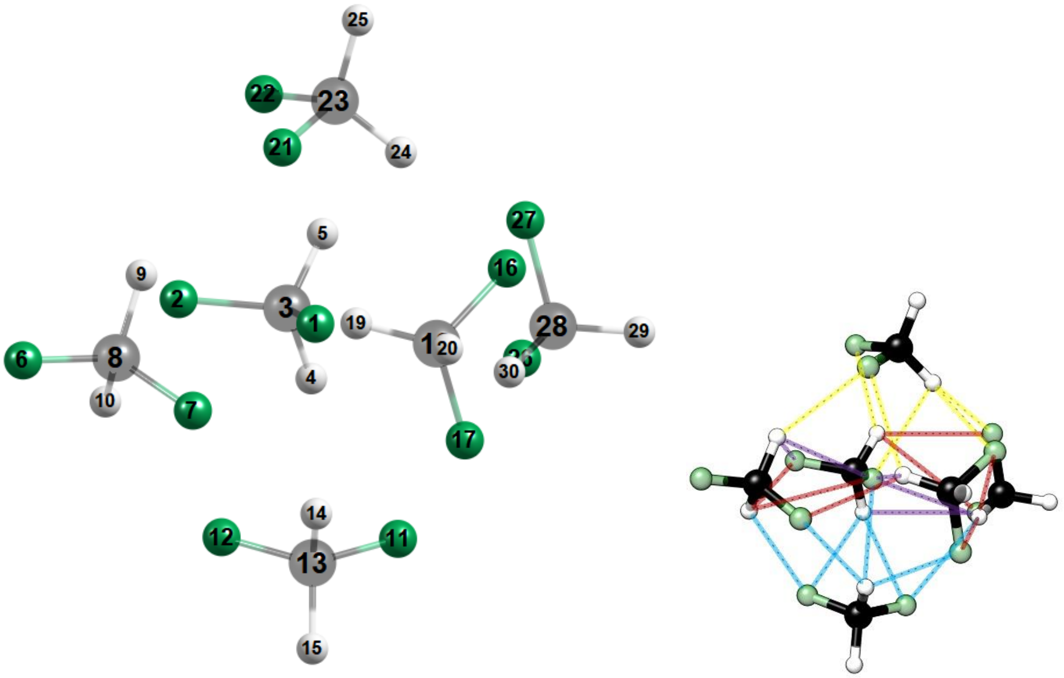


| F | H | Å | F | H | Å |
|---|---|---|---|---|---|
| 7 | 19 | 2.334 | 22 | 5 | 2.612 |
| 27 | 24 | 2.354 | 11 | 4 | 2.615 |
| 17 | 14 | 2.418 | 2 | 10 | 2.616 |
| 21 | 19 | 2.418 | 1 | 14 | 2.616 |
| 7 | 14 | 2.427 | 12 | 4 | 2.668 |
| 16 | 24 | 2.474 | 26 | 4 | 2.679 |
| 21 | 9 | 2.498 | 16 | 30 | 2.719 |
| 1 | 30 | 2.525 | 1 | 9 | 2.732 |
| 17 | 30 | 2.534 | 27 | 5 | 2.754 |
| 1 | 19 | 2.563 | 1 | 10 | 2.907 |
| 1 | 24 | 2.566 | 2 | 9 | 2.946 |
| 12 | 10 | 2.574 | 26 | 5 | 2.996 |
| 11 | 30 | 2.605 | | | |

Table S6. List of the 2.3 Å < CH···F ≤ 2.9 Å for the 7-II structure

**7-II**

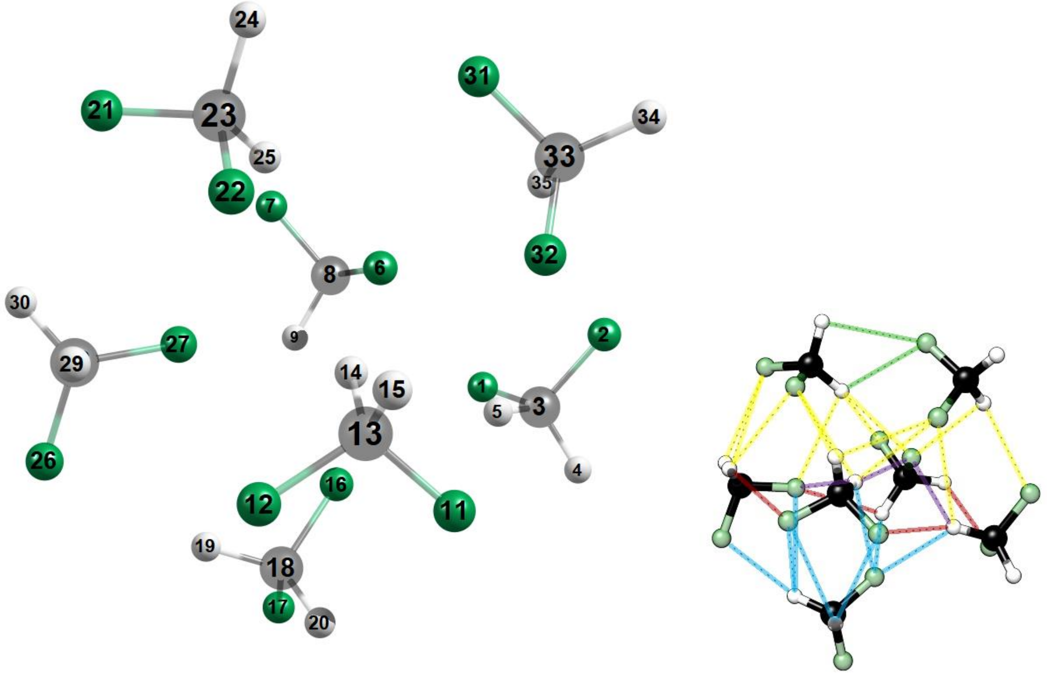


| F | H | Å | F | H | Å |
|---|---|---|---|---|---|
| 6 | 14 | 2.379 | 22 | 29 | 2.655 |
| 6 | 25 | 2.402 | 16 | 14 | 2.655 |
| 26 | 19 | 2.411 | 21 | 30 | 2.661 |
| 16 | 9 | 2.427 | 22 | 15 | 2.661 |
| 6 | 35 | 2.464 | 27 | 25 | 2.667 |
| 2 | 35 | 2.472 | 32 | 15 | 2.672 |
| 11 | 5 | 2.488 | 22 | 14 | 2.696 |
| 27 | 14 | 2.498 | 11 | 20 | 2.731 |
| 16 | 5 | 2.506 | 31 | 24 | 2.731 |
| 27 | 9 | 2.515 | 27 | 19 | 2.758 |
| 7 | 25 | 2.559 | 12 | 20 | 2.760 |
| 1 | 10 | 2.565 | 31 | 25 | 2.766 |
| 6 | 5 | 2.568 | 12 | 19 | 2.809 |
| 12 | 29 | 2.592 | 32 | 5 | 2.824 |
| 32 | 14 | 2.640 | 21 | 29 | 2.953 |

# 2 Computations

## 2.1 Structure Determination

### 2.1.1 Genetic Algorithm Search on Semi-empirical PES[S4]

Determining the structure of the most stable molecular clusters is challenging because it requires an accurate potential energy surface (PES). This is particularly difficult for weakly bound clusters like those composed of DFM.

To overcome these challenges, we employed a genetic algorithm (GA)[6] approach on semi-empirical PES. The GA approach is implemented in the OGOLEM[7] package which interfaces with the PM7[8] method implemented in MOPAC[9] and the HF-3c[10] method implemented in ORCA.[11]

The details of the GA runs are summarized in Table S7.

Table S7.Details of GA runs for $(CH_2F_2)_n$, where n = 2 − 7

| | GA package | SE package | Method | Pool size$^a$ | Number of cycles$^b$ |
|---|---|---|---|---|---|
| n=2-4 | OGOLEM | MOPAC | PM7 | 50-100 | 2,000 - 5,000 |
| | OGOLEM | ORCA | HF-3c | 20-50 | 1,000 |
| n=5-7 | OGOLEM | MOPAC | PM7 | 500 | 20,000 |
| | OGOLEM | ORCA | HF-3c | 250 | 5,000 |

$^a$ The size of the initial and/or maintained population.

$^b$ Number of matings or cycles for which the population is evolved.

GA searches on semi-empirical (PM7, HF-3c) PESs yield a large number of stable isomers which are subsequently subject to refinements using more robust *ab initio* methods.

### 2.1.2 Refinements using *ab initio* Methods

The low energy isomers from the previous step that are within 5 kcal mol$^{-1}$ of the putative global minimum for each method (PM7, HF-3c) are first optimized using second-order Møller-Plesset perturbation theory (MP2) with a split-valence 6-31+G(d) basis set. Those isomers that are within 3 kcal mol$^{-1}$ of the MP2/6-31+G(d) global minimum are further optimized using the larger double-zeta basis set (aug-cc-pVDZ or aVDZ). For each isomer which is within 2 kcal mol$^{-1}$ of the MP2/aVDZ global minimum, we do further geometric optimizations using very tight convergence criteria and calculate the harmonic vibrational calculations using ORCA.

The MP2 complete basis set limit (MP2/CBS) energy is estimated using explicitly correlated MP2-F12 method with cc-pVTZ-F12 orbital basis set along with cc-pVTZ- F12-CABS Complementary Auxiliary Basis Set (CABS) and cc-pVQZ/C auxiliary basis at the MP2/aVDZ optimized geometry. These methods are implemented in ORCA 4.2.1.[11] This method should fully be described as RI-MP2-F12/cc-pVTZ-F12//RI-MP2/aug-cc-pVDZ, however we will abbreviate it as MP2-F12/VTZ-F12//MP2/aVDZ in the rest of this manuscript for the sake of brevity.

These MP2-F12/VTZ-F12//MP2/aVDZ electronic energy ($E_e$) estimates are combined with the MP2/aVDZ harmonic zero-point and thermodynamic corrections to get zero-point corrected energies ($E_0$) and Gibbs free energies at finite temperatures [G(T)].

## 2.2 Rotational Constants, Principal Dipole Moments and Energies

### 2.2.1 $(CH_2F_2)_n$, where n = 2 − 4

The equilibrium theoretical rotational constants (A, B, C) and the principal dipole moment components ($\mu_a$, $\mu_b$, $\mu_c$) are reported in the table below. The binding energy ($\Delta E_e$, ΔE[0k], ΔG) for a cluster *k* is defined as the energy difference between the cluster ($E_k$) and its constituent monomers($E_1$). The relative energy ($\Delta\Delta E_e$, ΔΔE[0k], ΔΔG) for a cluster *k* is defined as the energy difference between the most stable cluster and cluster *k* ($E_k$).

$$\Delta E_k = E_k - n * E_1$$

$$\Delta\Delta E_k = E_k - \min_i\{E_i\}$$

The DFM clusters containing 2-4 monomers have already been studied by Prampolini et al.[12], but we have applied the above protocol to regenerate the structures and energies. The three most stable isomers for each cluster size are summarized in the table below.

Table S8. MP2/aVDZ rotational constants, principal dipole moments and MP2- F12/VTZ-F12//MP2/aVDZ relative energies of low energy isomers of $(CH_2F_2)_n$, where n = 2 − 4

| | Rot. Consts (MHz) | | | Prin. Dipole Moment (Debye) | | | | (kJ/mol) | |
|---|---|---|---|---|---|---|---|---|---|
| Label | A | B | C | $\mu_a$ | $\mu_b$ | $\mu_c$ | $\mu_{type}$ | $\Delta E_e$ | $\Delta\Delta E_e$ |
| 2-I | 6162 | 1333 | 1278 | 2.64 | -0.61 | 0.25 | a | -12.3 | 0.0 |
| 2-II | 12015 | 1004 | 938 | 0.00 | 0.00 | 0.00 | - | -10.8 | 1.5 |
| 2-III | 4516 | 1832 | 1435 | 0.00 | 0.01 | 0.00 | - | -9.4 | 2.9 |
| 3-I | 1386 | 840 | 622 | -2.58 | -0.76 | -0.69 | a | -27.2 | 0.0 |
| 3-II | 1222 | 1002 | 703 | 0.78 | 1.14 | 0.46 | b | -26.3 | 1.0 |
| 3-III | 1256 | 797 | 552 | 2.03 | -1.35 | -0.13 | a | -25.6 | 1.6 |
| 4-I | 663 | 661 | 383 | 0.00 | 0.00 | 0.00 | - | -45.7 | 0.0 |
| 4-II | 840 | 499 | 372 | 0.00 | 0.00 | 0.00 | - | -43.2 | 2.5 |
| 4-III | 857 | 437 | 333 | 3.41 | 0.11 | -0.52 | a | -43.0 | 2.7 |

[a] Ordered by relative electronic energy (ΔΔE), with the global minimum labeled 'I', followed by 'II', 'III', 'IV' ... *etc* for higher energy isomers
[b] MP2-F12/VTZ-F12//MP2/aVDZ single point energies combined with thermodynamic corrections at MP2/aVDZ level of theory

### 2.2.2 $(CH_2F_2)_5$

Pentamer data

Table S9. MP2/aVDZ rotational constants, principal dipole moments and MP2- F12/VTZ-F12//MP2/aVDZ relative energies of low energy isomers of $(CH_2F_2)_n$, where n = 5

| | Rot. Consts (MHz) | | | Prin. Dipole Moment (Debye) | | | | (kJ/mol) | |
|---|---|---|---|---|---|---|---|---|---|
| Label | A | B | C | $\mu_a$ | $\mu_b$ | $\mu_c$ | $\mu_{type}$ | $\Delta E_e$ | $\Delta\Delta E_e$ |
| 5-I | 441 | 375 | 292 | 0.2 | -2.2 | -0.9 | b | -61.3 | 0.0 |
| 5-II | 483 | 316 | 295 | -3.1 | 0.4 | -0.8 | a | -61.0 | 0.3 |
| 5-III | 495 | 331 | 231 | 1.4 | -0.4 | 0.2 | a | -60.8 | 0.4 |
| 5-IV | 495 | 330 | 279 | 4.0 | -0.1 | -1.2 | a | -60.3 | 1.0 |
| 5-V | 461 | 350 | 281 | 0.5 | 1.1 | -1.0 | | -60.2 | 1.0 |
| 5-VI | 437 | 340 | 291 | 0.9 | -0.5 | 1.9 | c | -58.8 | 2.5 |
| 5-VII | 426 | 351 | 270 | -1.3 | -1.3 | -0.5 | | -56.3 | 5.0 |
| Exp. 5-I | 426.61950(7) | 358.42855(5) | 281.54415(5) | weak | strong | medium | | | |

[a] Ordered by relative electronic energy (ΔΔE), with the global minimum labeled 'I', followed by 'II', 'III', 'IV' ... *etc* for higher energy isomers
[b] MP2-F12/VTZ-F12//MP2/aVDZ single point energies combined with thermodynamic corrections at MP2/aVDZ level of theory

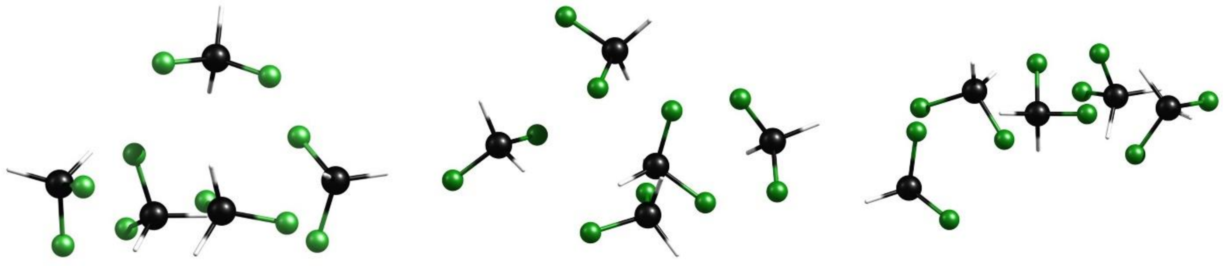

**5-I** **5-II** **5-III**

### 2.2.3 $(CH_2F_2)_6$

## Hexamer data

Table S10. MP2/aVDZ rotational constants, principal dipole moments and MP2- F12/VTZ-F12//MP2/aVDZ relative energies of low energy isomers of $(CH_2F_2)_n$, where n = 6

| | Rot. Consts (MHz) | | | Prin. Dipole Moment (Debye) | | | | (kJ/mol) | |
|---|---|---|---|---|---|---|---|---|---|
| Label | A | B | C | $\mu_a$ | $\mu b$ | $\mu_c$ | $\mu_{type}$ | $\Delta E_e$ | $\Delta\Delta E_e$ |
| 6-I | 367 | 224 | 196 | 3.6 | -0.8 | -0.4 | a | -79.6 | 0.0 |
| 6-II | 324 | 277 | 237 | -2.6 | -0.8 | -2.2 | | -79.2 | 0.4 |
| 6-III | 363 | 232 | 214 | -0.3 | 0.1 | -1.1 | c | -79.0 | 0.6 |
| 6-IV | 312 | 281 | 229 | -0.8 | 0.0 | -0.6 | a | -79.0 | 0.6 |
| 6-V | 339 | 245 | 224 | 0.6 | 0.5 | -2.2 | c | -78.9 | 0.7 |
| 6-VI | 372 | 226 | 190 | 3.7 | -0.2 | 0.6 | a | -78.9 | 0.7 |
| 6-VII | 347 | 224 | 207 | 1.9 | 0.4 | 0.1 | a | -78.8 | 0.8 |
| 6-VIII | 291 | 266 | 243 | 2.4 | 2.3 | -1.5 | | -78.6 | 1.0 |
| 6-IX | 342 | 259 | 217 | 1.0 | 1.5 | -0.9 | | -78.5 | 1.1 |
| 6-X | 298 | 269 | 223 | -2.0 | 0.9 | 0.2 | a | -77.5 | 2.1 |
| 6-XI | 362 | 230 | 210 | 0.8 | -0.3 | 1.4 | c | -76.7 | 2.7 |
| Exp. 6-I | 356.1116(7) | 211.52526(4) | 185.23390(5) | strong | weak | - | | | |
| Exp. 6-II | 304.72043(7) | 266.17778(5) | 226.35912(5) | medium | - | weak | | | |

[a] Ordered by relative electronic energy (ΔΔE), with the global minimum labeled 'I', followed by 'II', 'III', 'IV' ... *etc* for higher energy isomers
[b] MP2-F12/[illegible] of theory.

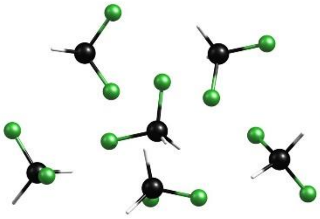

6-I

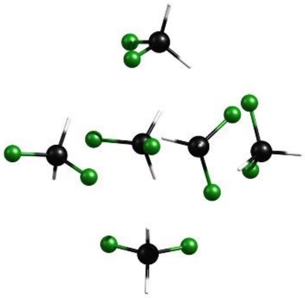

6-II

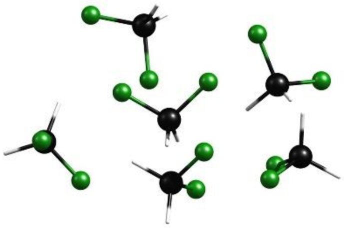

6-III

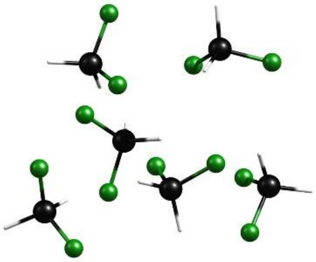

6-VI

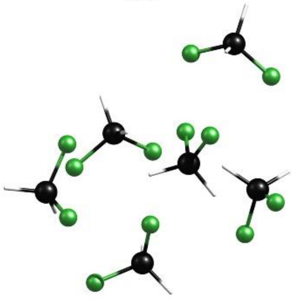

6-V

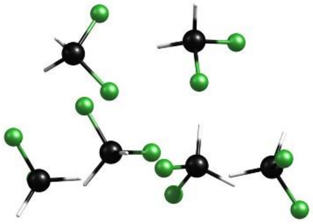

6-VI

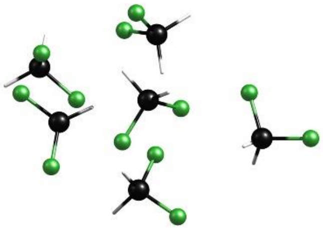

6-VII

### 2.2.4 $(CH_2F_2)_7$

Heptamer data

Table S11. MP2/aVDZ rotational constants, principal dipole moments and MP2- F12/VTZ-F12//MP2/aVDZ relative energies of low energy isomers of $(CH_2F_2)_n$, where n = 7

| | Rot. Consts (MHz) | | | Prin. Dipole Moment (Debye) | | | | (kJ/mol) | |
|---|---|---|---|---|---|---|---|---|---|
| Label | A | B | C | $\mu_a$ | $\mu_b$ | $\mu_c$ | $\mu_{type}$ | $\Delta E_e$ | $\Delta\Delta E_e$ |
| 7-I | 243 | 226 | 174 | 0.4 | 1.9 | 0.8 | b | -99.5 | 0.0 |
| 7-II | 233 | 222 | 162 | -1.8 | 2.1 | -0.5 | | -99.1 | 0.3 |
| 7-III | 232 | 222 | 159 | 2.1 | 0.6 | -1.2 | a | -98.4 | 1.0 |
| Exp. 7-II | 229.5257(1) | 215.0008(1) | 163.3655(1) | medium | strong | weak | | | |

[a] Ordered by relative electronic energy (ΔΔE), with the global minimum labeled 'I', followed by 'II', 'III', 'IV' ... *etc* for higher energy isomers
[b] MP2-F12/VTZ-F12//MP2/aVDZ single point energies combined with thermodynamic corrections at MP2/aVDZ level of theory

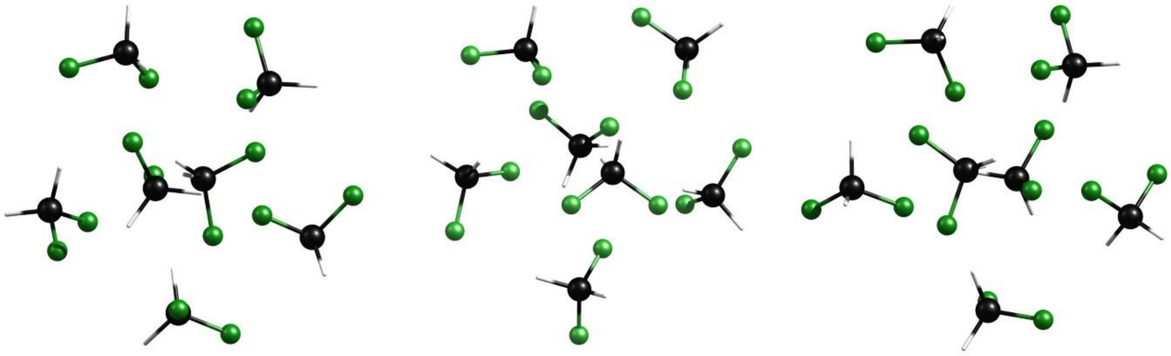

7-I 7-II 7-III

## 2.3 Many-Body Decomposition of Cluster Interaction Energies

### 2.3.1 Definition of Many-Body Decomposition

The total binding energy of a molecular cluster (E$_{bind}$) is often calculated in super-molecular approaches as the difference in energy between the cluster ($E_c$) and the $N$ monomers constituting the cluster ($E_m^i$) in their isolated gas phase minimum geometries.

$$E_{bind} = E_c - \sum_{i}^{N} E_m^i$$

While binding energies calculated this way are meaningful, it is much more interesting to examine the energy associated with distortion of the monomers as they form a cluster, and the interactions between the monomers or groups thereof. When formulated that way, the binding energy is a sum of the monomer distortion energy (E$_{dist}$) and interaction energy (E$_{int}$) of acluster.

$$E_{bind} = E_{dist} + E_{int}$$

The monomer distortion or relaxation energy (E$_{dist}$), which is also referred to as one-body energy (E$_{1B}$), is difference in energy between monomers in their isolated gas phase geometry ($E_m^i$) and cluster form ($E_c^i$)

$$E_{dist} = E_{iB} = \sum_{i}^{N} (E_c^i - E_m^i)$$

The interaction energy ($E_{int}$) can be expanded into its many-body components. The total interaction energy of a molecular cluster composed of $N$ monomers is the sum of its two-body (2B), three-body (3B), four-body (4B), ... , $N$-body (NB) components.

$$E_{int} = E_{iB} + E_{2B} + E_{3B} + \ldots + E_{NB}$$

$E_{2B}$ is the pair-wise interaction between all pairs of monomers in their cluster geometry.

$$E_{2B} = \sum_{i>j}^{N} \left[E_c^{ij} - \left(E_c^i + E_c^j\right)\right]$$

$E_{3B}$ is the interaction between all sets of three monomers in their cluster geometry, excluding the 2B contribution.

$$E_{3B} = \sum_{i>j>k}^{N} \left[E_c^{ijk} - \left(E_c^i + E_c^j + E_c^k\right)\right] - E_{2B}$$

E$_{4B}$ is the interaction between all sets of four monomers in their cluster geometry, excluding the smaller many-body contributions.

$$E_{4B} = \sum_{i>j>k>l}^{N} \left[E_c^{ijkl} - \left(E_c^i + E_c^j + E_c^k + E_c^l\right)\right] - E_{3B} - E_{2B}$$

and the general n-body contribution is

$$E_{\mathrm{n}B} = \sum_{i>j>\dots n}^{N} \left[E_c^{ij\dots n} - \left(E_c^i + E_c^j + \dots + E_c^n\right)\right] - \sum_{a=2}^{n-1} [E_{aB}]$$

The monomer distortion or relaxation energy ($E_{dist}$, $E_{iB}$) is a generally positive quantity because monomers are adopting higher energy configurations with higher energies than in their isolated gas phase geometry in order to optimize their interactions with the other monomers constituting the cluster. Two-body interactions ($E_{2B}$) are the largest contributor to the stabilization of clusters, accounting for more than 80% of the total interaction energy in water clusters. Three-body interactions ($E_{3B}$) are also important especially in clusters composed of polar molecules where long-range forces are strong. $E_{3B}$ generally accounts for 10-15% of the interaction energy in water clusters. Four- and other higher-body interactions may be non-negligible depending on the system and the desired level of accuracy.

For our purposes, many-body decomposition of the interaction energy provides insights into the strength of interactions between any pairs or triplets of monomers. Using a many-body decomposition code, we calculated all individual many-body interaction energy components between all sets of monomers to isolate different interactions.

Just to emphasize the subtle difference between the total interaction energy and binding energy, the two terms are defined below. Binding energy is the difference between the energy of the cluster and the energy of the isolated monomers in their minimum configuration. Interaction energy is the difference between the energy of the cluster and the energy of the isolated monomers in the cluster geometry. The difference between binding and interaction energy is the monomer distortion or relaxation energy ($E_{dist}$, $E_{iB}$)

### 2.3.2 Definition of Cooperativity

Cooperativity in non-covalent interactions is an interesting phenomenon worth investigating. Positive cooperativity implies that the total interaction energy is greater than the sum of all pairwise interactions. Based on the definition of MBD terms above, the cooperativity energy ($E_{coop}$) would them be the sum of all n-body contributions past 2-body interactions:

$$E_{\mathrm{coop}} = E_{3B} + E_{4B} + E_{5B} + \dots$$

$$E_{\mathrm{coop}} = \sum_{a=3}^{n-1} [E_{aB}]$$

### 2.3.3 $(CH_2F_2)_2$

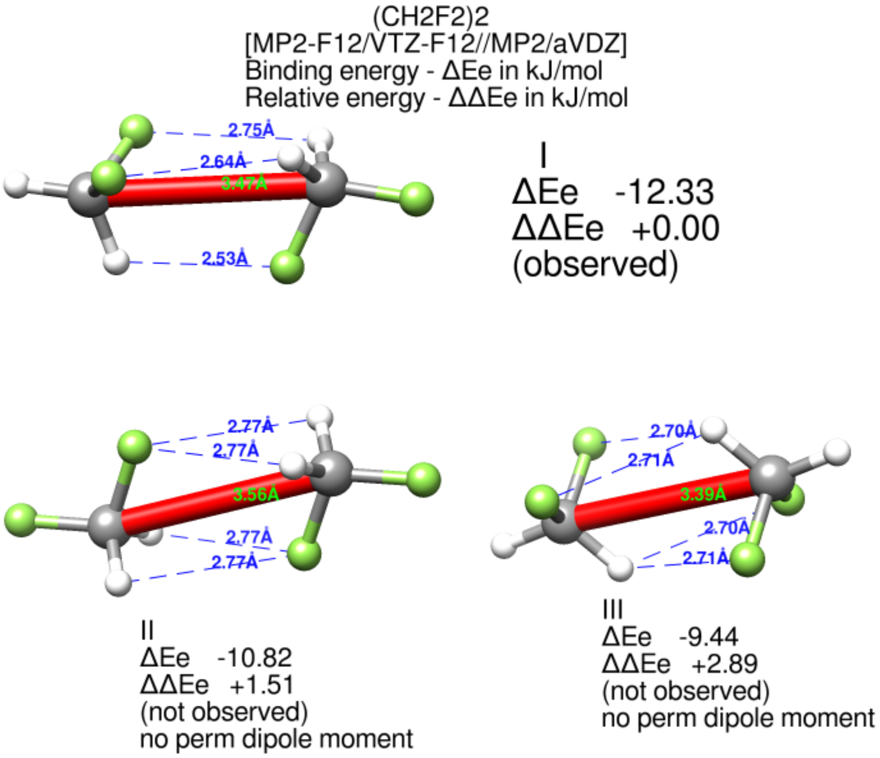


Figure S2. The three lowest energy isomers of $(CH_2F_2)_2$. The MBD of the interaction energy of (I) is reported in the table below.

Table S12. Many-body decomposition of the MP2-F12/VTZ-F12//MP2/aVDZ binding energy of $(CH_2F_2)_2$-I

| All energies in kJ/mol | $(CH_2F_2)_2$-I |
|---|---|
| Individual 1-body contributions: | |
| Fragment 1(1) : | 0.5 |
| Fragment 2(2) : | 0.5 |
| | |
| Individual 2-body contributions: | |
| Fragment 1(1-2) : | -13.4 |
| | |
| Many-body energies: | |
| 1-body = | 1.0 |
| 2-body = | -13.3 |
| | |
| Interaction Energy = | -13.3 |
| **Binding Energy =** | **-12.3** |

### 2.3.4 $(CH_2F_2)_3$

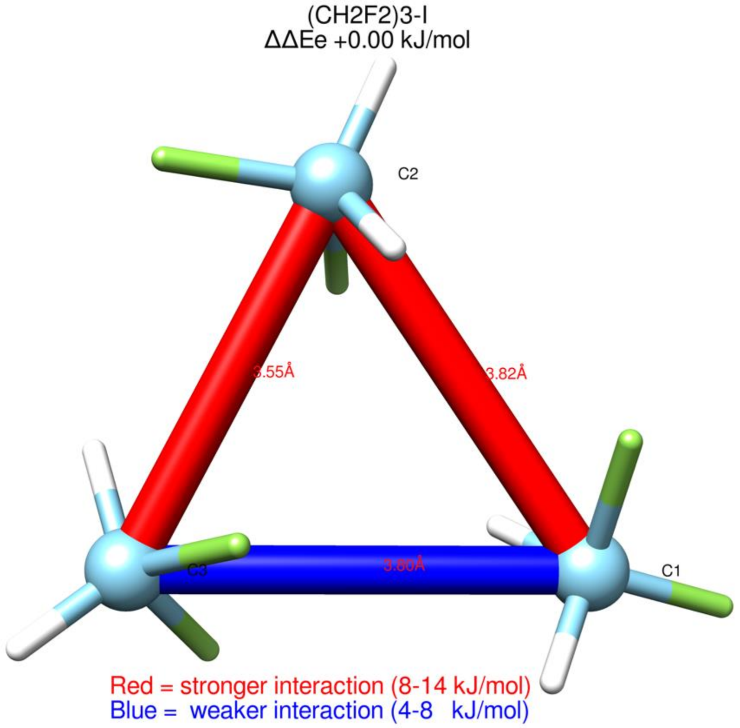


Figure S3.The lowest energy isomers of $(CH_2F_2)_3$. The carbon labels (C1,C2,C3) correspond to the monomer/fragment numbers in the table below.

Table 13. Many-body decomposition of the MP2-F12/VTZ-F12//MP2/aVDZ binding energy of $(CH_2F_2)_3$-I

| | $(CH_2F_2)_3$-I |
|---|---|
| Individual 1-body contributions: | |
| Fragment 1(1) : | 0.8 |
| Fragment 2(2) : | 1.0 |
| Fragment 3(3) : | 0.7 |
| Individual 2-body contributions: | |
| Fragment 1(1-2) : | -10.3 |
| Fragment 2(1-3) : | -7.4 |
| Fragment 3(2-3) : | -11.4 |
| Individual 3-body contributions: | |
| Fragment 1(1-2-3) : | -0.5 |
| Many-body energies: | |
| 1-body = | 2.5 |
| 2-body = | -29.2 |
| 3-body = | -0.5 |
| Interaction Energy = | -29.7 |
| **Binding Energy =** | **-27.2** |

## 2.3.5 $(CH_2F_2)_4$

Please note that the rotational spectrum of the two lowest energy isomers was not observed because they did not have a permanent dipole moment. Therefore, we report results for the observed isomer, $(CH_2F_2)_4$-III.

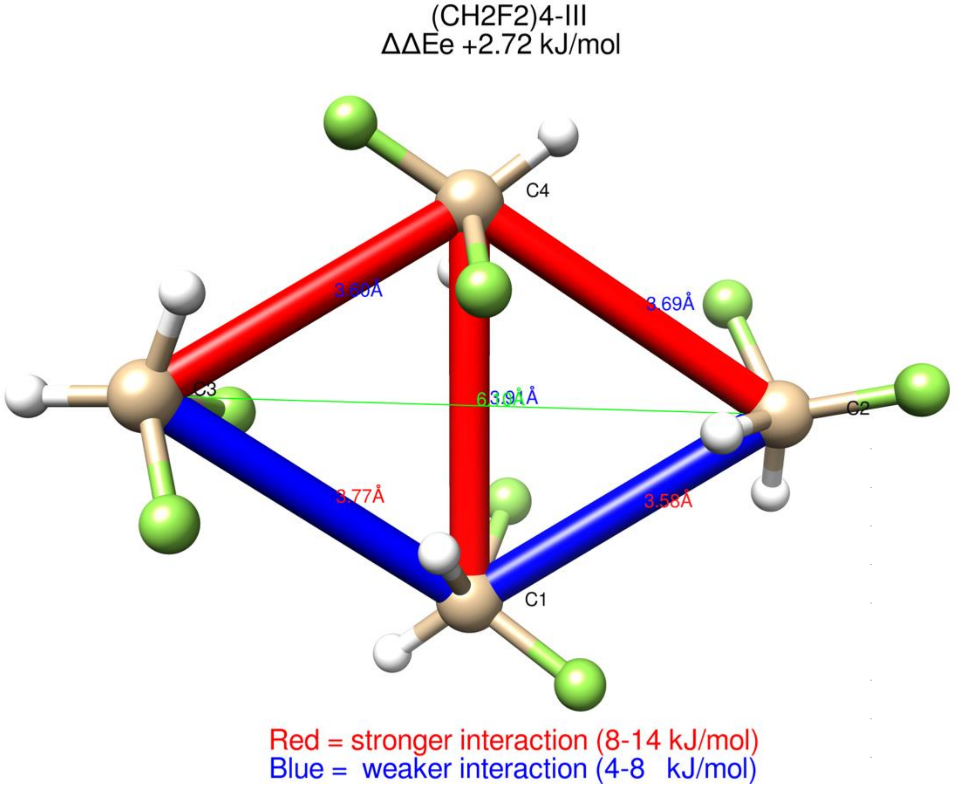


Figure S4. The third lowest energy isomer of $(CH_2F_2)_4$. The carbon labels (C1,C2, …) correspond to the monomer/fragment numbers in the table below.

Table S14. Many-body decomposition of the MP2-F12/VTZ-F12//MP2/aVDZ binding energy of $(CH_2F_2)_4$-III

| | $(CH_2F_2)_4$-III |
|---|---|
| Individual 1-body contributions: | |
| Fragment 1(1) : | 1.0 |
| Fragment 2(2) : | 0.6 |
| Fragment 3(3) : | 0.8 |
| Fragment 4(4) : | 1.5 |
| Individual 2-body contributions: | |
| Fragment 1(1-2) : | -5.5 |
| Fragment 2(1-3) : | -6.7 |
| Fragment 3(1-4) : | -10.6 |
| Fragment 4(2-3) : | -1.4 |
| Fragment 5(2-4) : | -10.6 |
| Fragment 6(3-4) : | -10.9 |
| Individual 3-body contributions: | |
| Fragment 1(1-2-3) : | -0.7 |
| Fragment 2(1-2-4) : | 0.0 |
| Fragment 3(1-3-4) : | -0.5 |
| Fragment 4(2-3-4) : | 0.0 |
| Individual 4-body contributions: | |
| Fragment 1(1-2-3-4) : | -0.1 |
| Many-body energies: | |
| 1-body = | 4.0 |
| 2-body = | -45.8 |
| 3-body = | -1.1 |
| 4-body = | -0.1 |
| Interaction Energy = | -47.0 |
| **Binding Energy** = | **-43.0** |

### 2.3.6 $(CH_2F_2)_5$

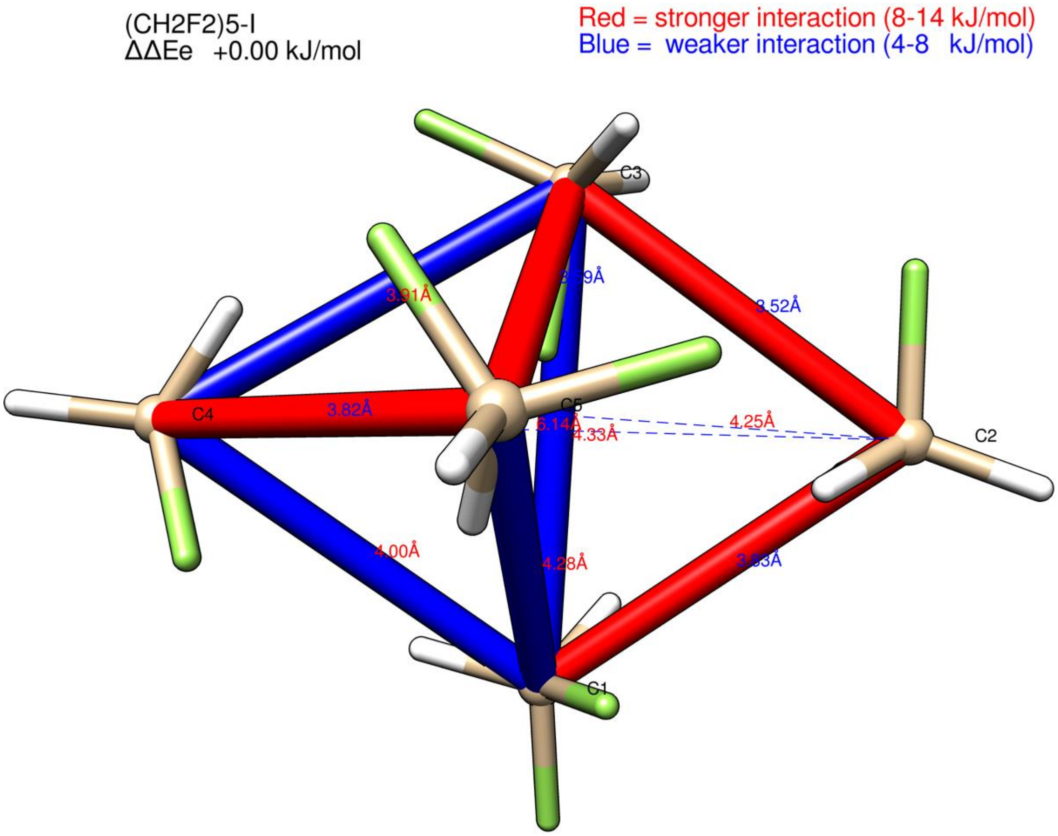


Figure S5.The lowest energy isomers of $(CH_2F_2)_5$. The carbon labels (C1,C2,C3) correspond to the monomer/fragment numbers in the table below.

Table S15. Many-body decomposition of the MP2-F12/VTZ-F12//MP2/aVDZ binding energy of $(CH_2F_2)_5$-I

| | $(CH_2F_2)_5$-I |
|---|---|
| Individual 1-body contributions: | |
| Fragment 1(1) : | 1.4 |
| Fragment 2(2) : | 1.0 |
| Fragment 3(3) : | 1.2 |
| Fragment 4(4) : | 1.0 |
| Fragment 5(5) : | 1.2 |
| Individual 2-body contributions: | |
| Fragment 1(1-2) : | -10.6 |
| Fragment 2(1-3) : | -5.4 |
| Fragment 3(1-4) : | -5.8 |
| Fragment 4(1-5) : | -4.3 |
| Fragment 5(2-3) : | -10.8 |
| Fragment 6(2-4) : | -0.8 |
| Fragment 7(2-5) : | -1.8 |
| Fragment 8(3-4) : | -5.1 |
| Fragment 9(3-5) : | -10.9 |
| Fragment 10(4-5) : | -10.8 |
| Individual 3-body contributions: | |
| Fragment 1(1-2-3) : | -0.2 |
| Fragment 2(1-2-4) : | 0.0 |
| Fragment 3(1-2-5) : | 0.0 |
| Fragment 4(1-3-4) : | 0.2 |
| Fragment 5(1-3-5) : | -0.5 |
| Fragment 6(1-4-5) : | -0.4 |
| Fragment 7(2-3-4) : | -0.3 |
| Fragment 8(2-3-5) : | 0.4 |
| Fragment 9(2-4-5) : | 0.1 |
| Fragment 10(3-4-5) : | -0.2 |
| Individual 4-body contributions: | 0.0 |
| Individual 5-body contributions: | 0.0 |
| | |
| Many-body energies: | |
| 1-body = | 5.8 |
| 2-body = | -66.2 |
| 3-body = | -0.9 |
| 4-body = | 0.0 |
| 5-body = | 0.0 |
| | |
| Interaction Energy = | -67.1 |
| **Binding Energy** = | **-61.3** |

### 2.3.7 $(CH_2F_2)_6$

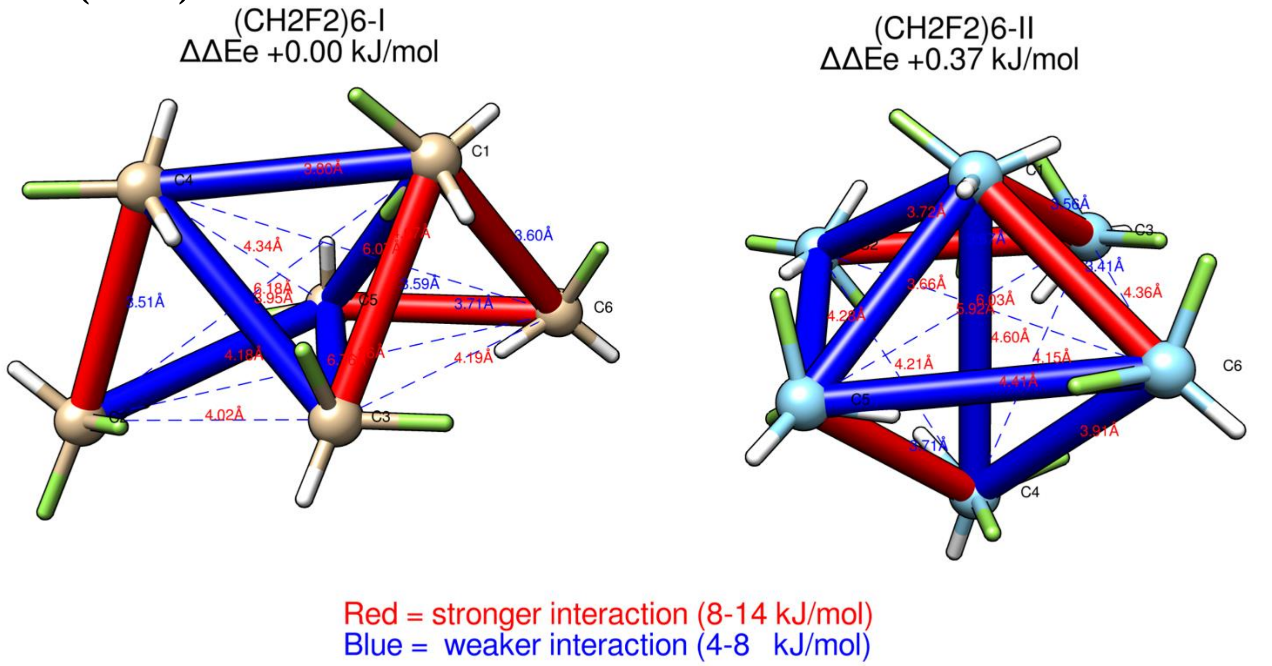


Figure S6.The two lowest energy isomers of $(CH_2F_2)_6$. The carbon labels (C1,C2,C3) correspond to the monomer/fragment numbers in the table below.

Table S16. Many-body decomposition of the MP2-F12/VTZ-F12//MP2/aVDZ binding energy of $(CH_2F_2)_6$-I and $(CH_2F_2)_6$-II

| | $(CH_2F_2)_6$-I | $(CH_2F_2)_6$-II |
|---|---|---|
| Individual 1-body contributions: | | |
| Fragment 1(1) : | 1.2 | 1.7 |
| Fragment 2(2) : | 1.2 | 1.6 |
| Fragment 3(3) : | 1.4 | 0.9 |
| Fragment 4(4) : | 1.2 | 1.3 |
| Fragment 5(5) : | 1.2 | 1.0 |
| Fragment 6(6) : | 0.9 | 1.0 |
| Individual 2-body contributions: | | |
| Fragment 1(1, 2) | -1.2 | -6.9 |
| Fragment 2(1, 3) | -11.4 | -10.9 |
| Fragment 3(1, 4) | -5.8 | -5.9 |
| Fragment 4(1, 5) | -4.2 | -4.2 |
| Fragment 5(1, 6) | -9.3 | -11.7 |
| Fragment 6(2, 3) | -4.1 | -10.7 |
| Fragment 7(2, 4) | -13.3 | -3.5 |
| Fragment 8(2, 5) | -5.6 | -6.2 |
| Fragment 9(2, 6) | -0.7 | -1.5 |
| Fragment 10(3, 4) | -7.6 | -1.8 |
| Fragment 11(3, 5) | -5.6 | -0.6 |
| Fragment 12(3, 6) | -2.5 | -1.2 |
| Fragment 13(4, 5) | -2.5 | -10.4 |
| Fragment 14(4, 6) | -0.4 | -6.4 |
| Fragment 15(5, 6) | -11.6 | -4.3 |
| Individual 3-body contributions: | -1.0 | -0.6 |
| Individual 4-body contributions: | 0.0 | 0.0 |
| Individual 5-body contributions: | 0.0 | 0.0 |
| Individual 6-body contributions: | 0.0 | 0.0 |
| Many-body energies: | | |
| 1-body = | 7.1 | 7.5 |
| 2-body = | -85.7 | -86.1 |
| 3-body = | -1.0 | -0.6 |
| 4-body = | 0.0 | 0.0 |
| 5-body = | 0.0 | 0.0 |
| 6-body = | 0.0 | 0.0 |
| Interaction Energy = | -86.7 | -86.7 |
| **Binding Energy =** | **-79.6** | **-79.2** |

### 2.3.8 $(CH_2F_2)_7$

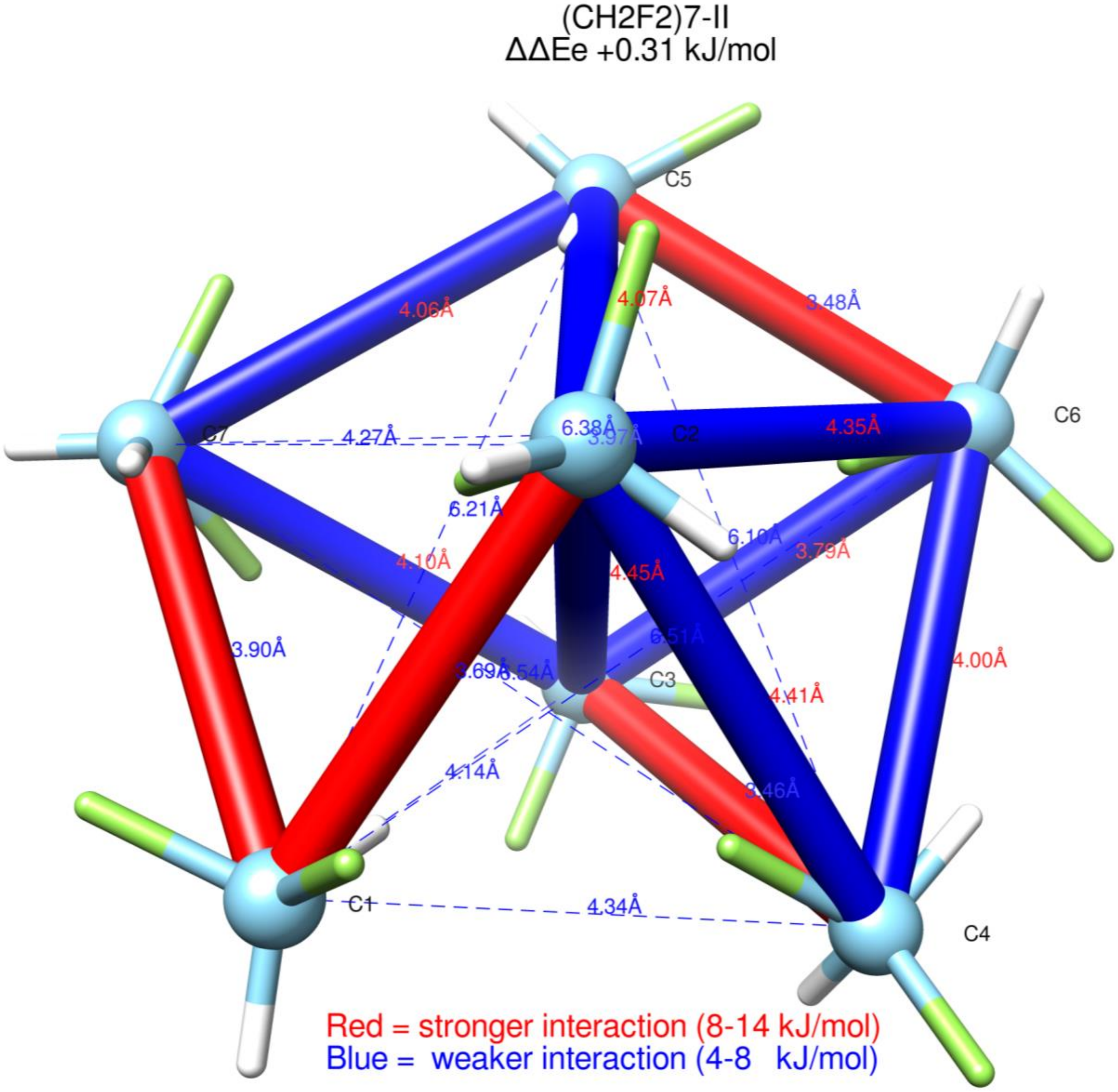


Figure S7.The lowest energy isomers of $(CH_2F_2)_7$. The carbon labels (C1,C2,C3) correspond to the monomer/fragment numbers in the table below.

Table S17. Many-body decomposition of the MP2-F12/VTZ-F12//MP2/aVDZ binding energy of $(CH_2F_2)_7$-II

| | $(CH_2F_2)_7$-II |
|---|---|
| Individual 1-body contributions: | |
| Fragment 1(1) : | 1.0 |
| Fragment 2(2) : | 1.5 |
| Fragment 3(3) : | 1.5 |
| Fragment 4(4) : | 1.2 |
| Fragment 5(5) : | 1.3 |
| Fragment 6(6) : | 1.1 |
| Fragment 7(7) : | 0.7 |
| Individual 2-body contributions: | |
| Fragment 1(1, 2) | -9.7 |
| Fragment 2(1, 3) | -3.7 |
| Fragment 3(1, 4) | -2.5 |

| | |
|---|---|
| Fragment 4(1, 5) | -1.0 |
| Fragment 5(1, 6) | -0.8 |
| Fragment 6(1, 7) | -9.9 |
| Fragment 7(2, 3) | -6.2 |
| Fragment 8(2, 4) | -5.1 |
| Fragment 9(2, 5) | -6.8 |
| Fragment 10(2, 6) | -5.2 |
| Fragment 11(2, 7) | -1.5 |
| Fragment 12(3, 4) | -11.6 |
| Fragment 13(3, 5) | -2.8 |
| Fragment 14(3, 6) | -6.4 |
| Fragment 15(3, 7) | -6.4 |
| Fragment 16(4, 5) | -1.9 |
| Fragment 17(4, 6) | -4.2 |
| Fragment 18(4, 7) | -1.0 |
| Fragment 19(5, 6) | -13.2 |
| Fragment 20(5, 7) | -4.9 |
| Fragment 21(6, 7) | -0.7 |
| | |
| Individual 3-body contributions: | -1.8 |
| Individual 4-body contributions: | -0.1 |
| Individual 5-body contributions: | 0.0 |
| Individual 6-body contributions: | 0.0 |
| Individual 7-body contributions: | 0.0 |
| | |
| Many-body energies: | |
| 1-body = | 7.1 |
| 2-body = | -85.7 |
| 3-body = | -1.0 |
| 4-body = | 0.0 |
| 5-body = | 0.0 |
| 6-body = | 0.0 |
| 7-body = | 0.0 |
| | |
| Interaction Energy = | -107.5 |
| **Binding Energy =** | **-99.1** |

### 2.3.9 Comparison of MBD of $(CH_2F_2)_n$ and $(H_2O)_n$

The table below summarizes the breakdown of the total interaction energy into its many body components for all the observed clusters. **Figure S7** shows that virtually all the interaction energy in these clusters comes from pair-wise (two-body) interactions. Higher many-body interactions are essentially zero. These findings suggest that pairwise interactions between the different monomers in the clusters dictate the overall structure of the cluster. There are not higher-order effects that give the clusters distinct structural features.

Table S18. N-body contribution to the interaction energy as a fraction of the total interaction energy (E_nb/E_total)

| | $(CH_2F_2)_{2\text{-}7}$ clusters | | | | | | | | |
|---|---|---|---|---|---|---|---|---|---|
| **nb** | **2-I** | **3-I** | **4-III** | **4-I** | **5-I** | **6-I** | **6-II** | **7-II** | **7-I** |
| 1b | -0.08 | -0.09 | -0.09 | -0.09 | -0.09 | -0.09 | -0.10 | -0.08 | -0.08 |
| 2b | 1.08 | 1.07 | 1.07 | 1.09 | 1.08 | 1.08 | 1.09 | 1.07 | 1.07 |
| 3b | | 0.02 | 0.03 | 0.00 | 0.01 | 0.01 | 0.01 | 0.02 | 0.01 |
| 4b | | | 0.00 | 0.00 | 0.00 | 0.00 | 0.00 | 0.00 | 0.00 |
| 5b | | | | | 0.00 | 0.00 | 0.00 | 0.00 | 0.00 |
| 6b | | | | | | 0.00 | 0.00 | 0.00 | 0.00 |
| 7b | | | | | | | | 0.00 | 0.00 |

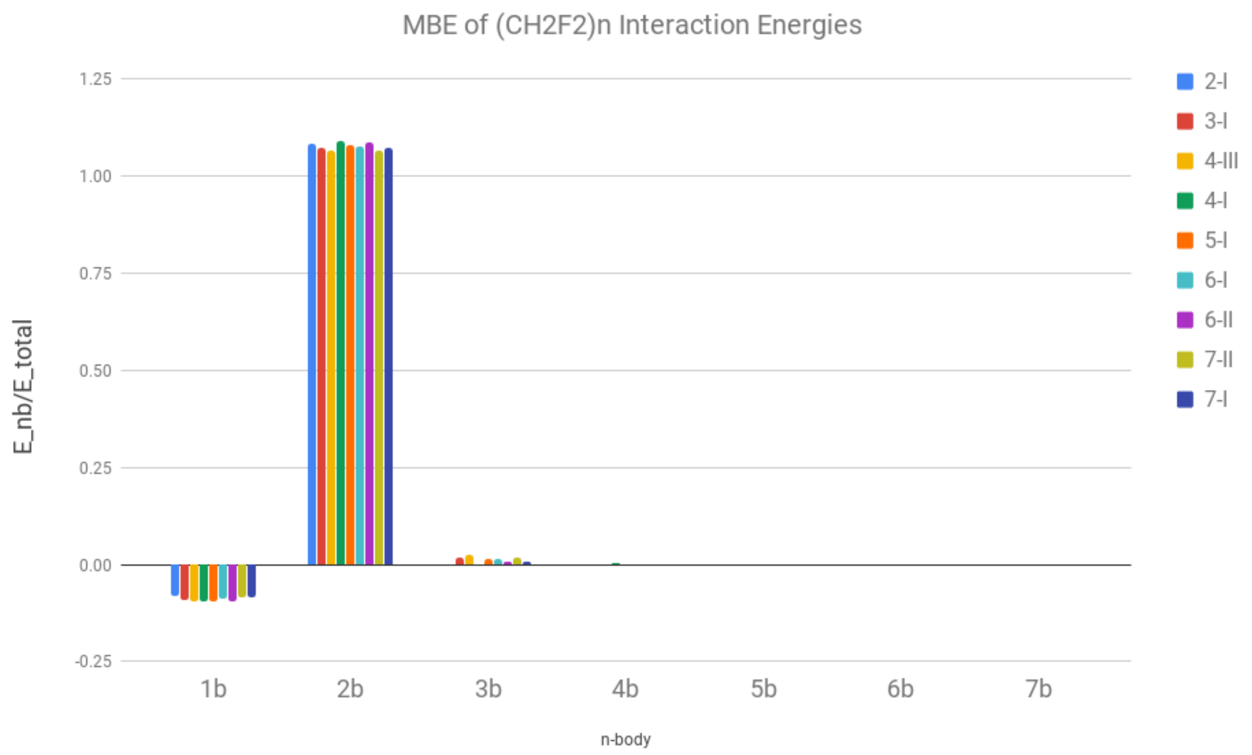


Figure S8. N-body contributions to the total interaction of $(CH_2F_2)_n$, n=2-7. 2-body interactions are dominant while larger N-body contributions are essentially zero.

The dominance of two-body interactions are in contrast to other systems like water clusters where three-body effects give the clusters their marked structural features.

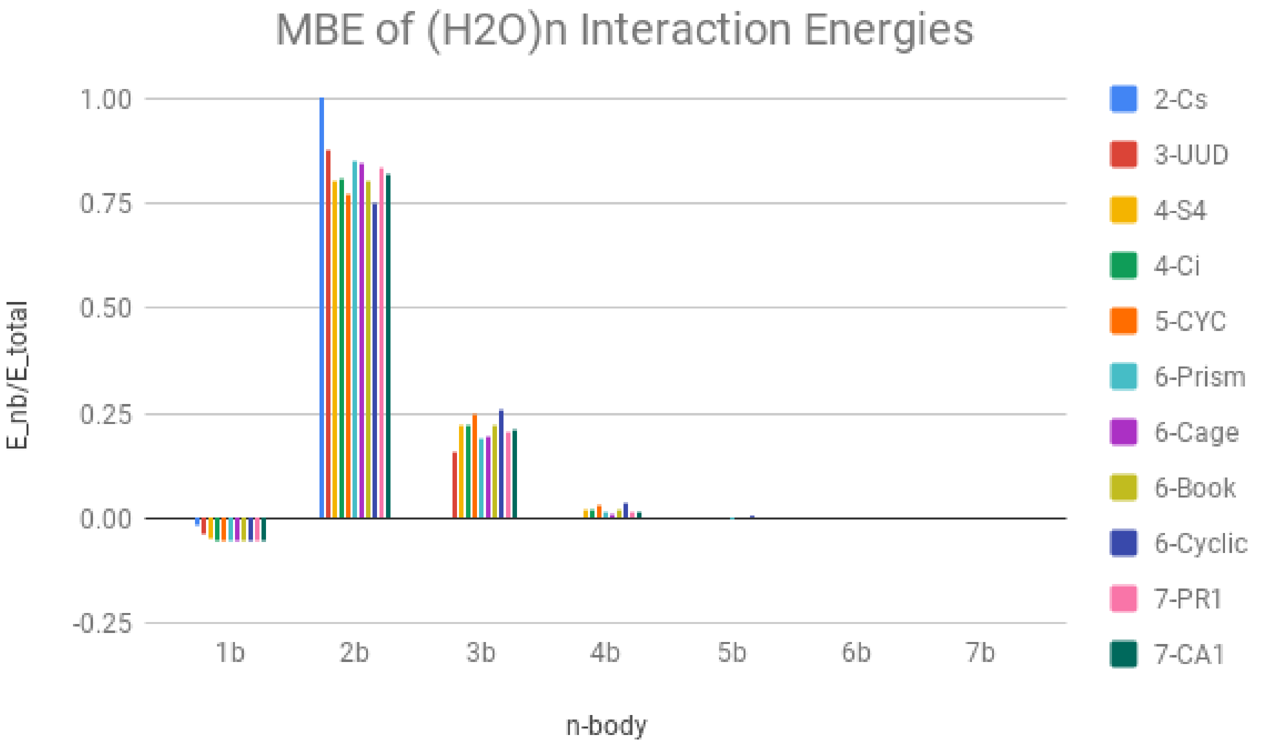


Figure S9. N-body contributions to the total interaction of $(H_2O)_n$, n=2-7. While 2-body interactions account for about 75% of the total interaction energy, 3-body interactions are significant (~20%) as well.

### 2.3.10 Distribution of two-body interaction energies and comparison with $(CH_2F_2)_2$

We have demonstrated that the interaction energy of difluoromethane clusters is largely dominated by pairwise (two-body) interactions. We can safely think of the clusters as a collection of dimers that distort to optimize their pair-wise interactions.

These constituent dimers span a large range in terms of geometry and binding energy. Some are very strongly bound, with structures and energies resembling that of an isolated dimer. These have two-body interaction energies ($E_{2b}$) close to that of the most stable isolated dimer (about -13 kJ/mol) or that of low energy stable (minima) and meta-stable ($1^{st}$-order transition states) (-8 to -13 kJ/mol). Others look like less stable dimers with $E_{2b}$ of -4 to -8 kJ/mol, while the remaining have small attractive (0 to -4 kJ/mol) or even repulsive ($E_{2b} > 0$) interactions.

**Figure S9** shows a histogram of the distribution of these two-body interaction energies in the $(CH_2F_2)_{n=2-7}$ clusters investigated in this study. It demonstrates that there are a set of strong ( -8 > $E_{2b}$ > -13 ), weak (-4 > $E_{2b}$ > -8) and very weak ($E_{2b}$ > -4) dimers.

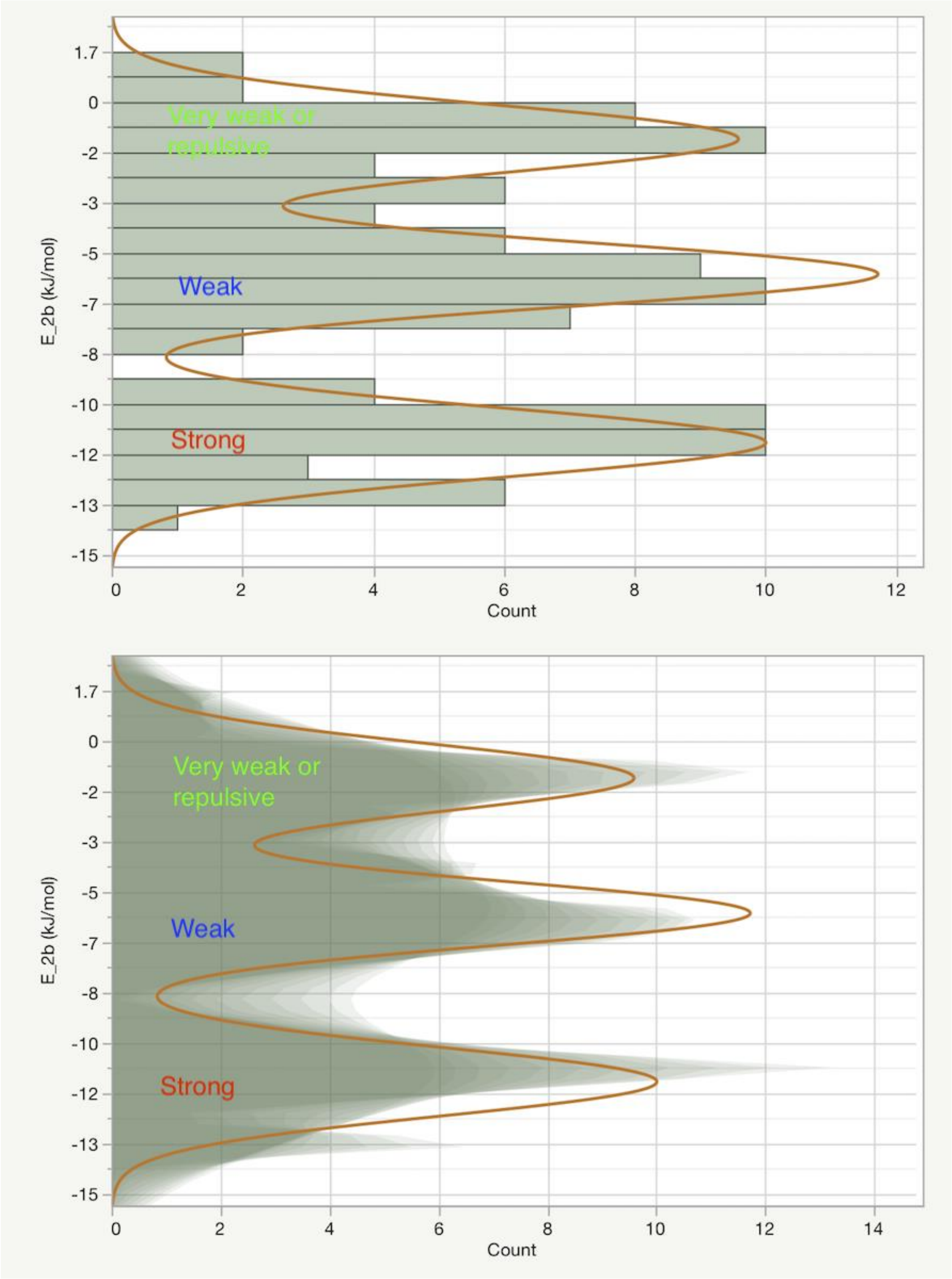


Figure S10. The distribution of the two-body interaction energies in the $(CH_2F_2)_{n=2-7}$ clusters investigated in this study. The figures show a histogram (top) or shadow histogram fitted to a sum of three normal distribution functions (bottom).

The two-body interaction energies of clusters are similar to those of isolated dimers, as shown in the figure below. Most of the isolated dimer isomers shown below are strongly bound although it is possible to find more weakly bound dimers if we search for higher-order transition states. Nevertheless, the presence of so many stable or metastable dimer building blocks leads to a vast number of conformers in larger clusters.

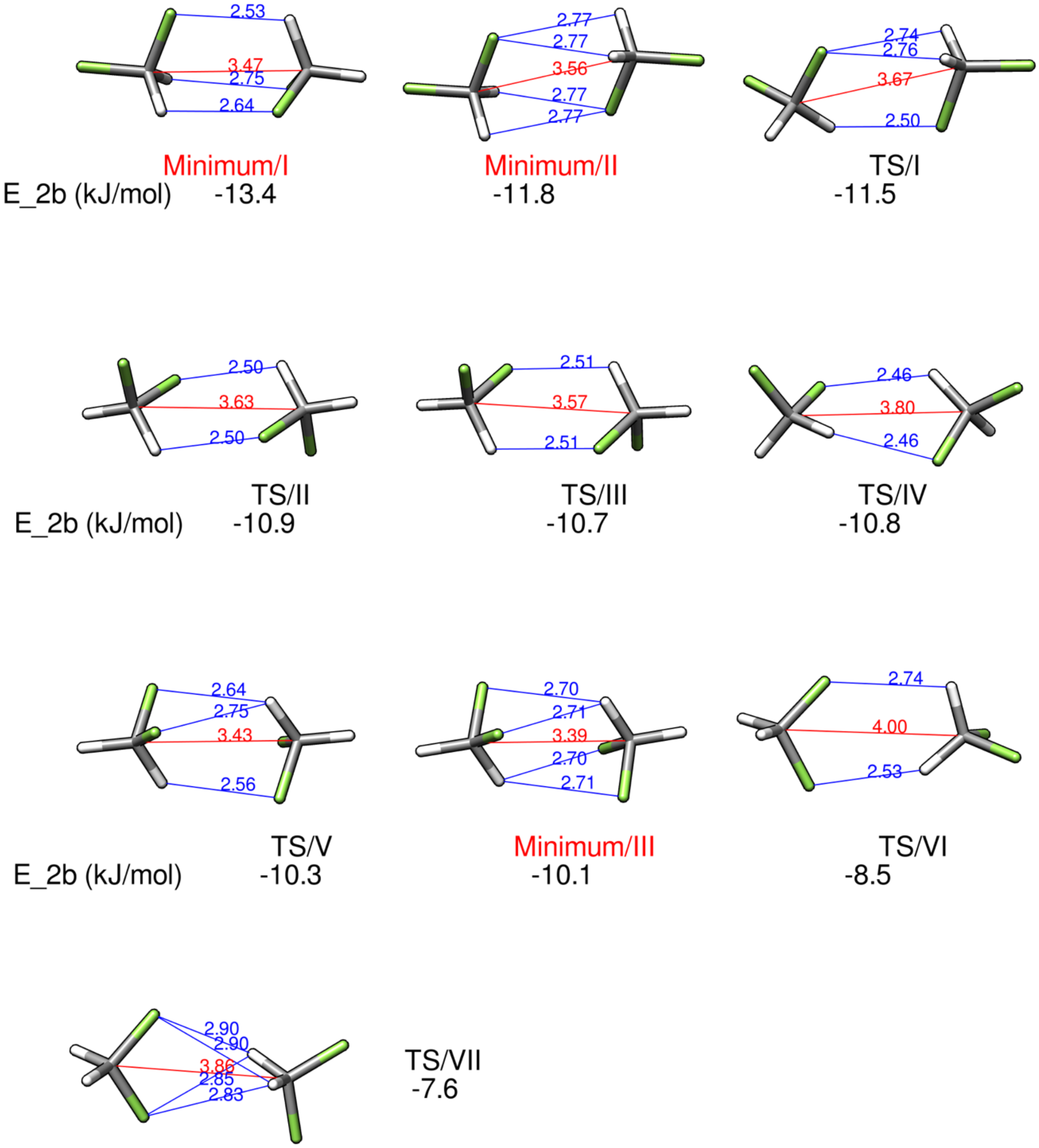


Figure S11. The interaction energy of many low energy minima and first-order transition states of difluoromethane dimer.

Therefore, we can think of these clusters as a set of monomers held together by pair-wise interactions. There are little higher-order, non-additive contributions that lead to particular structural motifs.

### 2.3.11 Takeaways from MBD Analysis

The many-body decomposition of the binding energy reported above leads to the following conclusions.

- **Monomer distortion:** Due to the limited degrees of freedom available to the DFM monomers, they undergo very small distortions relative to their isolated analogs. The distortion energy per monomer is usually 1-2 kJ/mol, which is comparable to water clusters.
- **Two-body interaction:** As mentioned in the previous section, these 2B interactions are what hold the clusters together. Each monomer distorts to optimize its pairwise interactions with its neighbors. The fact that the isolated dimer itself has a large number of low-energy isomers (see **Figure S10**) confers vast conformational flexibility to the larger clusters.
- **Three- and higher-body interactions:** Many-body interactions past two-body ones are negligible and they have little to no effect on the structures of the clusters.
- **Cooperativity:** the small three- and higher-body interaction energy contributions suggest that cooperative effects are present, but very small.

Therefore, we can think of these clusters as a set of monomers held together by pair-wise interactions. Higher-order, non-additive contributions are too small to lead to particular structural motifs.

## 2.4 Using SAPT to understand MBD Findings

One reason many-body contributions to the interaction energy of DFM clusters are very small relative to water clusters is due to the increased importance of dispersion in DFM clusters compared to prototypical hydrogen bonded systems like water clusters. Dispersion is less directional, therefore it allows for more disordered structures.

We can decompose the interaction energy of any dimer into its physically meaningful constituents (electrostatics, exchange repulsion, induction and dispersion) using symmetry-adapted perturbation theory (SAPT)[13,14] as implemented in PSI4[15] package. To understand the importance of these different constituents, we have chosen to compare DFM dimer with water dimer and methane dimer.

Table S19. SAPT2+3/aVDZ interaction energy components of three prototypical dimers

| Dimers | Electrostatics | Exchange | Induction | Dispersion | Total |
|---|---|---|---|---|---|
| $(H_2O)_2$ | -34.1 | 34.73 | -10.38 | -9.46 | -19.25 |
| $(CH_2F_2)_2$ | -15.15 | 15.23 | -2.43 | -10.67 | -13.01 |
| $(CH_4)_2$ | -1.26 | 4.39 | -0.21 | -4.81 | -1.88 |

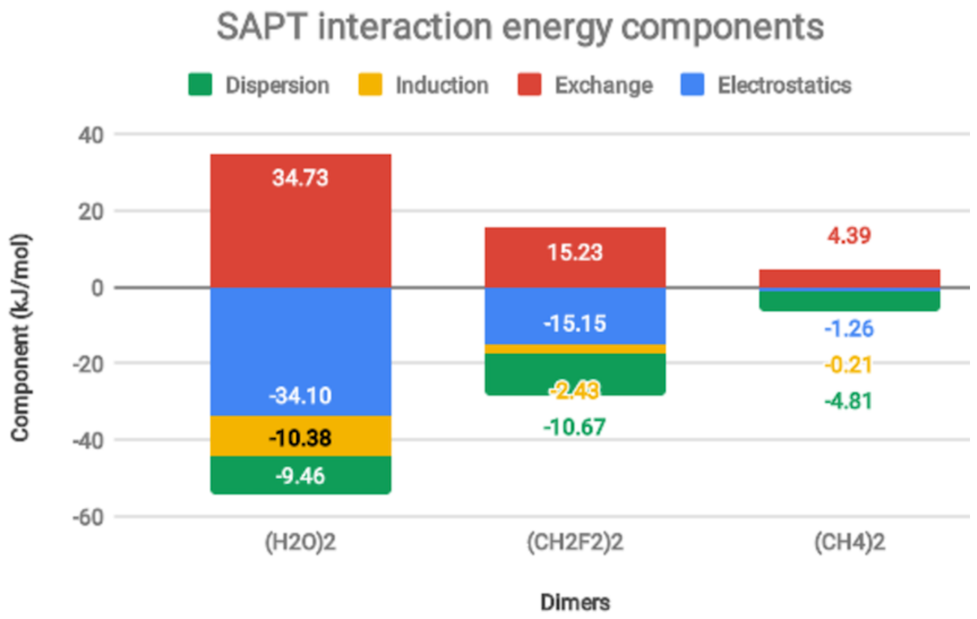


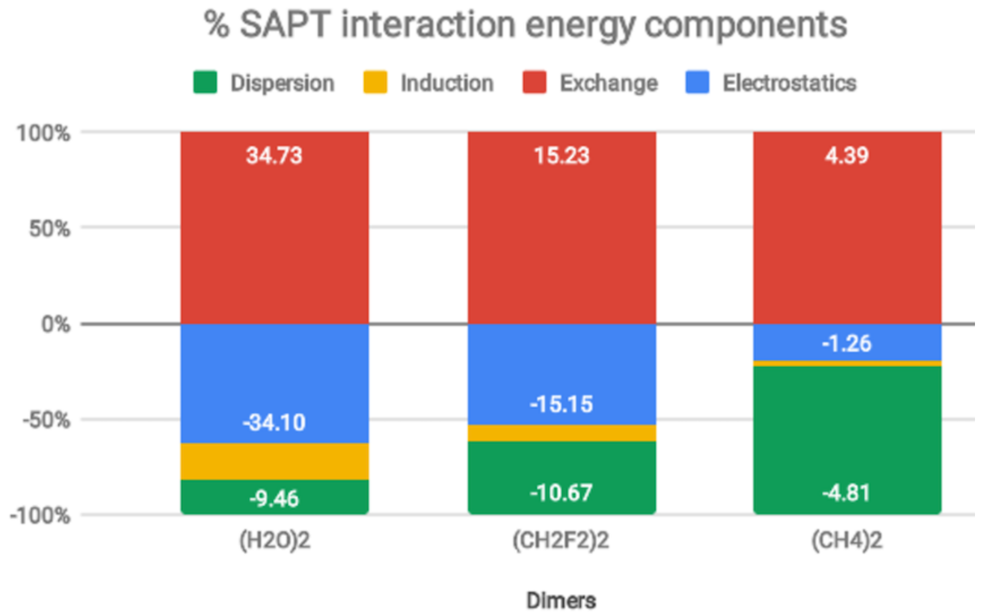


Figure S12. SAPT2+3/aVDZ interaction energy components (top) as a percentage of the total interaction energy (bottom)

As **Table S19** and **Figure S13** show, the total magnitude and constituent components of the interaction energy differ a lot in these three dimers.

- Water dimer is held together by a strong hydrogen bond (~20 kJ $mol^{-1}$). Due to water monomer's permanent dipole moment and the lone pairs on the oxygens, all three attractive components (electrostatics, induction and dispersion) of the SAPT interaction energy are important. The electrostatic component cancels out exchange repulsion and that leaves induction and dispersion as the remaining attractive components.
- DFM dimer is held together by a number of weak hydrogen bonds (two ~6 kJ $mol^{-1}$ or three ~4 kJ $mol^{-1}$ or four ~3 kJ $mol^{-1}$). While it has a permanent dipole and large electron density around the fluorine, the electrostatic component in DFM dimer is about half that of water dimer. The

electrostatic component here is cancelled out by exchange repulsion of the same magnitude, leaving induction and dispersion as the main forces holding the dimer together. The dispersion component in DFM dimer is in fact larger than that of water dimer. The increased importance of dispersion leads to less directional interaction and more disordered structures.

- Methane dimer is largely held together by dispersion interactions because the monomers do not have permanent dipole moment. The absence of a strongly directional interaction means the shapes they form are mainly a function of steric repulsion and dispersive attraction.

Therefore, the progressively larger role dispersion plays in the total interaction energy as one goes from water dimer to DFM dimer to methane dimer correlates with the increasingly disordered structure of the larger clusters.

## 2.5 Cartesian Coordinates

### 2.5.1 $CH_2F_2$ monomer

Cartesian coordinates of the DFM monomer and observed clusters is provided below in Angstrom units.

Table S20. MP2/aVDZ optimized Cartesian coordinates of DFM ($CH_2F_2$) monomer

| | | | |
|---|---|---|---|
| 5 | | | |
| Monomer | | | |
| F | 2.2235 | 1.1410 | -1.0907 |
| F | 3.4390 | 0.9691 | 0.7724 |
| C | 3.1619 | 0.3727 | -0.4378 |
| H | 4.0805 | 0.3584 | -1.0385 |
| H | 2.7304 | -0.6186 | -0.2479 |

### 2.5.2 $(CH_2F_2)_2$

Table S21. MP2/aVDZ optimized Cartesian coordinates of $(CH_2F_2)_2$

| | | | |
|---|---|---|---|
| 10 | | | |
| Dimer-I(observed) | | | |
| C | -1.49129 | -0.52627 | 0.06876 |
| H | -1.39502 | -0.85984 | 1.10855 |
| H | -1.05131 | -1.20707 | -0.66905 |
| F | -0.87114 | 0.70524 | -0.05183 |
| F | -2.82640 | -0.35406 | -0.22205 |
| C | 1.97096 | -0.47371 | 0.16271 |
| H | 3.05028 | -0.62115 | 0.29030 |
| H | 1.65465 | 0.57136 | 0.07312 |
| F | 1.32254 | -1.03762 | 1.24418 |
| F | 1.56635 | -1.15487 | -0.96698 |

### 2.5.3 $(CH_2F_2)_3$

Table S22. MP2/aVDZ optimized Cartesian coordinates of $(CH_2F_2)_3$

| | | | |
|---|---|---|---|
| 15 | | | |
| Trimer-I(observed) | | | |
| F | 0.346241 | 2.310068 | -0.035071 |
| F | -1.250138 | 3.727727 | -0.676258 |
| C | -0.957754 | 2.392970 | -0.497220 |
| H | -1.624152 | 1.981371 | 0.267631 |
| H | -1.011727 | 1.885838 | -1.467333 |
| F | -1.011318 | 0.625365 | 2.306962 |
| F | 0.262312 | -1.189537 | 2.516957 |
| C | 0.299178 | 0.181745 | 2.349952 |
| H | 0.784149 | 0.626044 | 3.227324 |
| H | 0.774403 | 0.411213 | 1.391777 |
| F | -0.709021 | -0.674775 | -0.441241 |
| F | -2.907288 | -0.349489 | -0.281818 |
| C | -1.897991 | -1.257444 | -0.038444 |
| H | -1.844301 | -1.448729 | 1.038319 |
| H | -2.072032 | -2.146279 | -0.657024 |

### 2.5.4 $(CH_2F_2)_4$

Table S23. MP2/aVDZ optimized Cartesian coordinates of $(CH_2F_2)_4$

| | | | |
|---|---|---|---|
| 20 | | | |
| Tetramer-III(observed) | | | |
| C | -0.11610 | 1.97410 | 0.32100 |
| H | -0.83260 | 2.64210 | 0.81200 |
| H | -0.53550 | 1.37590 | -0.49260 |
| F | 0.41190 | 1.12010 | 1.27420 |
| F | 0.93030 | 2.73050 | -0.17270 |
| C | 2.86360 | 0.04030 | -0.15170 |
| H | 3.05820 | 0.90500 | 0.49170 |
| H | 2.04130 | 0.17830 | -0.86270 |
| F | 2.56610 | -1.04750 | 0.65300 |
| F | 4.00880 | -0.26420 | -0.85380 |
| C | -3.23670 | -0.03830 | -0.32760 |
| H | -2.89670 | -0.97700 | -0.77630 |
| H | -4.30580 | -0.00190 | -0.08590 |
| F | -2.94800 | 1.00060 | -1.19090 |
| F | -2.51980 | 0.17860 | 0.83530 |
| C | -0.23260 | -1.93130 | 0.26970 |
| H | -0.22350 | -1.31680 | 1.17500 |
| H | 0.61030 | -2.62190 | 0.16870 |
| F | -0.23860 | -1.08480 | -0.83080 |
| F | -1.40870 | -2.65710 | 0.22860 |

### 2.5.5 $(CH_2F_2)_5$

Table S24. MP2/aVDZ optimized Cartesian coordinates of $(CH_2F_2)_5$-I

| | | | |
|---|---|---|---|
| 25 | | | |
| Pentamer-I(observed) | | | |
| F | 8.74518 | -11.41164 | -0.01849 |
| F | 7.49198 | -9.74584 | -0.81100 |
| C | 7.86381 | -10.42039 | 0.34921 |
| H | 8.37567 | -9.71110 | 1.00703 |
| H | 6.96567 | -10.88135 | 0.77325 |
| F | 4.61106 | -11.17463 | 0.11047 |
| F | 3.21436 | -9.47115 | -0.22722 |
| C | 4.22388 | -10.21759 | -0.80889 |
| H | 5.07330 | -9.56018 | -1.01311 |
| H | 3.81067 | -10.72916 | -1.68632 |
| F | 5.06227 | -7.19180 | 2.93084 |
| F | 5.76452 | -8.53165 | 1.29413 |
| C | 4.63422 | -7.97904 | 1.88111 |
| H | 4.02176 | -8.79547 | 2.27815 |
| H | 4.13674 | -7.34605 | 1.14014 |
| F | 8.49929 | -8.21144 | 3.00157 |
| F | 8.85555 | -7.18242 | 1.05821 |
| C | 8.39219 | -6.99903 | 2.35239 |
| H | 9.05096 | -6.27816 | 2.85139 |
| H | 7.33812 | -6.71138 | 2.30919 |
| F | 5.23404 | -7.11250 | -1.35479 |
| F | 6.14985 | -5.82442 | 0.21730 |
| C | 6.43276 | -6.59008 | -0.90038 |
| H | 7.08736 | -7.41463 | -0.60834 |
| H | 6.83581 | -5.92799 | -1.67585 |

### 2.5.6 $(CH_2F_2)_6$

Table S25. MP2/aVDZ optimized Cartesian coordinates of $(CH_2F_2)_6$-I

| | | | |
|---|---|---|---|
| 30 | | | |
| Hexamer-I(observed) | | | |
| F | 1.34150 | 0.83650 | -0.59240 |
| F | 1.26840 | 3.03510 | -0.93840 |
| C | 2.05290 | 2.01750 | -0.43500 |
| H | 2.96820 | 1.94880 | -1.03200 |
| H | 2.21010 | 2.18500 | 0.63550 |
| F | -2.72930 | 0.08960 | 1.15770 |
| F | -4.15390 | -1.62450 | 1.20380 |
| C | -3.34880 | -0.87640 | 0.37340 |
| H | -2.57660 | -1.52340 | -0.05500 |
| H | -3.97570 | -0.36180 | -0.36330 |
| F | 1.31430 | 0.36830 | 2.47640 |
| F | -0.16950 | 1.87120 | 1.76960 |
| C | -0.01270 | 0.53950 | 2.10750 |
| H | -0.64480 | 0.32380 | 2.97530 |
| H | -0.21200 | -0.07690 | 1.22830 |
| F | -1.41090 | 0.33150 | -1.50650 |
| F | -2.98120 | 1.90880 | -1.51500 |
| C | -1.63540 | 1.68630 | -1.30550 |
| H | -1.06420 | 2.24470 | -2.05470 |
| H | -1.39090 | 1.93110 | -0.26770 |
| F | 0.27320 | -2.07700 | 0.12890 |
| F | -1.00440 | -2.62210 | -1.61220 |
| C | 0.26330 | -2.21620 | -1.25360 |
| H | 0.97460 | -3.00420 | -1.52230 |
| H | 0.46630 | -1.23800 | -1.69950 |
| F | 4.08410 | -0.00340 | 0.29650 |
| F | 3.18490 | -1.70460 | -0.83220 |
| C | 3.40710 | -1.20110 | 0.43640 |
| H | 2.44320 | -1.00490 | 0.91310 |
| H | 2.44320 | -1.00490 | 0.91310 |
| H | 4.05680 | -1.90110 | 0.97530 |

Table S26. MP2/aVDZ optimized Cartesian coordinates of $(CH_2F_2)_6$-II

| | | | |
|---|---|---|---|
| 30 | | | |
| Hexamer-II(observed) | | | |
| F | 0.19430 | -0.25060 | 0.57220 |
| F | -0.14720 | -0.64050 | 2.74200 |
| C | 0.80360 | -0.68710 | 1.74950 |
| H | 1.61080 | 0.01160 | 1.98860 |
| H | 1.11470 | -1.72610 | 1.60150 |
| F | -3.85060 | 1.16210 | 1.54980 |
| F | -2.23370 | 1.51780 | 0.05670 |
| C | -2.55490 | 0.82970 | 1.22580 |
| H | -2.50140 | -0.24330 | 1.01550 |
| H | -1.88490 | 1.17690 | 2.01870 |
| F | 2.01450 | 2.10650 | 0.47630 |
| F | 0.38800 | 2.38330 | 1.97590 |
| C | 0.78850 | 2.70770 | 0.69190 |
| H | 0.06440 | 2.29570 | -0.01510 |
| H | 0.92300 | 3.79460 | 0.63770 |
| F | 0.11400 | -0.70950 | -2.83220 |
| F | 0.31450 | 1.43460 | -2.26080 |
| C | -0.60350 | 0.42150 | -2.48500 |
| H | -1.14800 | 0.22810 | -1.55800 |
| H | -1.23140 | 0.70810 | -3.33660 |
| F | -2.13570 | -1.86300 | -0.85110 |
| F | -1.03020 | -2.95090 | 0.75170 |
| C | -1.09450 | -2.74700 | -0.61130 |
| H | -0.16030 | -2.28810 | -0.94700 |
| H | -1.34060 | -3.70030 | -1.09410 |
| F | 3.50080 | -0.59250 | 0.18870 |
| F | 2.17400 | -1.98760 | -0.92680 |
| C | 2.81940 | -0.76260 | -0.99920 |
| H | 3.54740 | -0.80840 | -1.81750 |
| H | 2.07040 | 0.02850 | -1.09400 |

### 2.5.7 $(CH_2F_2)_7$

Table S27. MP2/aVDZ optimized Cartesian coordinates of $(CH_2F_2)_7$-II

| | | | |
|---|---|---|---|
| 35 | | | |
| Heptamer-II(observed) | | | |
| F | -0.28813 | -3.25292 | 1.40896 |
| F | -2.06494 | -3.39518 | 0.07282 |
| C | -0.68255 | -3.30769 | 0.08631 |
| H | -0.27571 | -4.22428 | -0.35659 |
| H | -0.38117 | -2.38561 | -0.41696 |
| F | -0.96994 | -0.40566 | 1.10835 |
| F | -0.64856 | 0.77516 | 2.96807 |
| C | -0.33453 | -0.41466 | 2.34645 |
| H | 0.74444 | -0.46125 | 2.17408 |
| H | -0.74761 | -1.24178 | 2.93252 |
| F | 0.51387 | -1.21592 | -2.42158 |
| F | 1.56984 | 0.73006 | -2.17236 |
| C | 0.34307 | 0.09984 | -2.02330 |
| H | 0.06874 | 0.11805 | -0.96679 |
| H | -0.37722 | 0.57693 | -2.69407 |
| F | 1.84333 | -1.44107 | 0.24513 |
| F | 4.00510 | -1.98213 | 0.30530 |
| C | 3.08038 | -1.18579 | -0.33416 |
| H | 3.32995 | -0.13425 | -0.16077 |
| H | 3.02777 | -1.47846 | -1.38848 |
| F | -0.70997 | 3.86928 | 0.74012 |
| F | -0.96322 | 2.60646 | -1.07579 |
| C | -1.46565 | 2.84716 | 0.19348 |
| H | -2.49988 | 3.19343 | 0.09422 |
| H | -1.33591 | 1.94771 | 0.80021 |
| F | 3.33273 | 2.24893 | 0.20541 |
| F | 1.29285 | 1.50823 | 0.70909 |
| C | 2.00331 | 2.61694 | 0.27626 |
| H | 1.65367 | 2.87955 | -0.72690 |
| H | 1.89567 | 3.40806 | 1.02574 |
| F | -3.57877 | 0.70713 | -0.24024 |
| F | -2.38514 | -0.70634 | -1.48458 |
| C | -3.42892 | -0.62389 | -0.58422 |
| H | -4.34521 | -0.95631 | -1.08639 |
| H | -3.16348 | -1.19345 | 0.31071 |

## 2.6 Quantitative analysis for difluoromethane tetramer

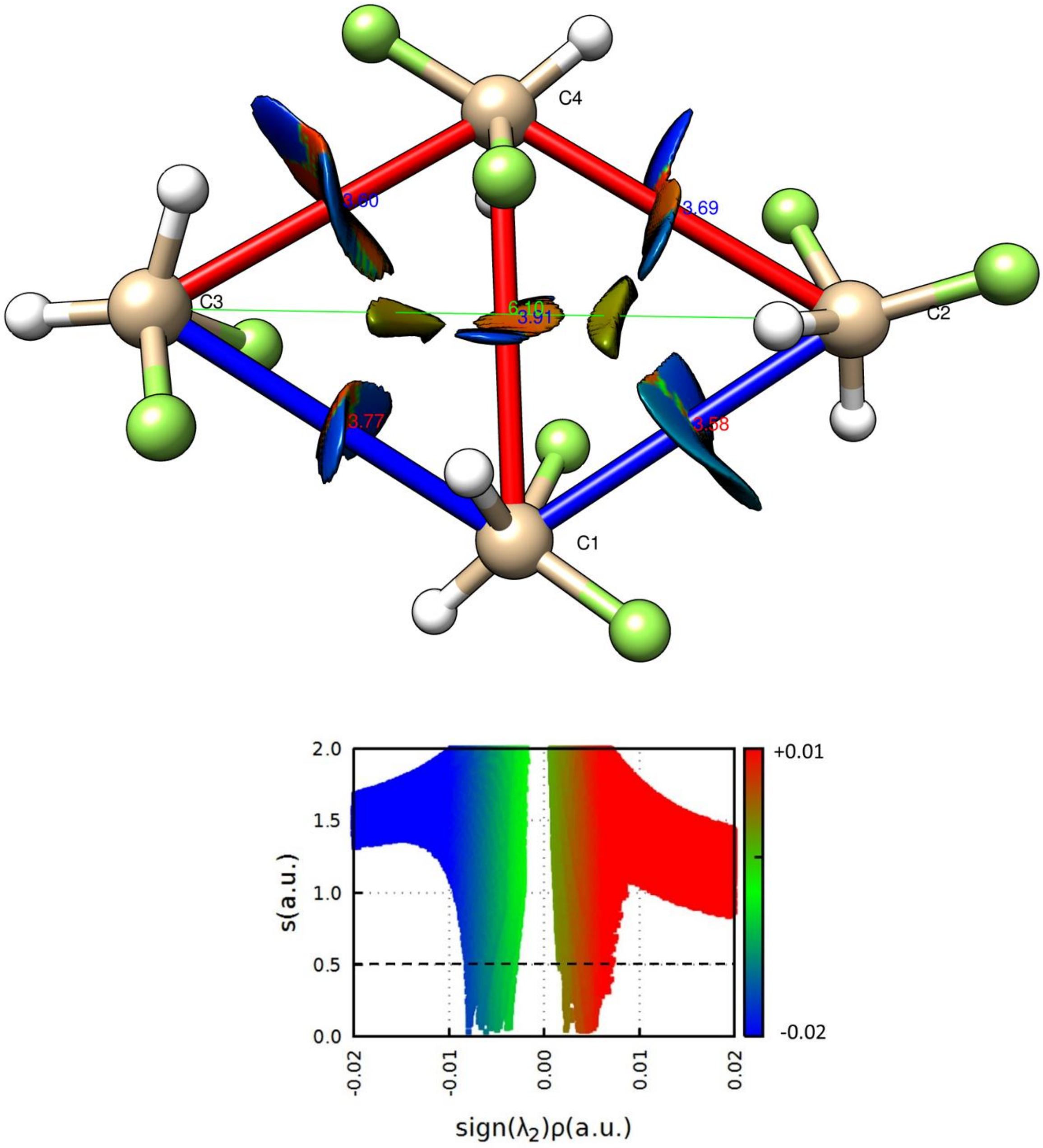


Figure S13. Quantitative analysis of attractive and repulsive interactions using the QTAIM procedure and NCI iso-surfaces. The carbon-carbon interaction distances are also reported. Blue- and green-colored areas indicate the presence of strong and weak attractive interactions, respectively. Red color signifies repulsive interactions. Bond-critical-point (BCP) paths are represented as blue lines. The iso-surface of the NCI analysis was build using s=0.5 and a blue-green-red color scale from -0.02 < sign($\lambda_2$ ) $\rho$(r) < +0.01 a.u.

# References


[1] G. G. Brown, B. C. Dian, K. O. Douglass, S. M. Geyer, S. T. Shipman, B. H. Pate, *Rev. Sci. Instrum.* **2008**, *79*, 053103.

[2] C. Pérez, S. Lobsiger, N. A. Seifert, D. P. Zaleski, B. Temelso, G. C. Shields, Z. Kisiel, B. H. Pate, *Chem. Phys. Lett.* **2013**, *571*, 1–15.

[3] I. Uriarte, C. Pérez, E. Caballero-Mancebo, F. J. Basterretxea, A. Lesarri, J. A. Fernández, E. J. Cocinero, *Chem. - A Eur. J.* **2017**, *23*, 7156.

[4] J. K. G. Watson, *Vibrational Spectra and Structure*, Elsevier Science Publishers, New York, Amsterdam, **1977**.

[5] T. T. Odbadrakh, A. G. Gale, B. T. Ball, B. Temelso, G. C. Shields, *J. Vis. Exp.* **2020**, *158*, e60964.

[6] D. E. Goldberg, *Genetic Algorithms in Search, Optimization and Machine Learning*, Addison-Wesley Longman Publishing Co., Inc., USA, **1989**.

[7] J. M. Dieterich, B. Hartke, *Mol. Phys.* **2010**, *108*, 279–291.

[8] J. J. P. Stewart, *J. Mol. Model.* **2013**, *19*, 1–32.

[9] J. J. P. Stewart, *MOPAC* **2008**.

[10] R. Sure, S. Grimme, *J. Comput. Chem.* **2013**, *34*, 1672–1685.

[11] F. Neese, *WIREs Comput. Mol. Sci.* **2018**, *8*, e1327.

[12] G. Prampolini, L. Carbonaro, G. Feng, L. Evangelisti, W. Caminati, I. Cacelli, *J. Chem. Theory Comput.* **2014**, *10*, 2204–2211.

[13] K. Szalewicz, *Wiley Interdiscip. Rev. Comput. Mol. Sci.* **2012**, *2*, 254–272.

[14] T. M. Parker, L. A. Burns, R. M. Parrish, A. G. Ryno, C. D. Sherrill, *J. Chem. Phys.* **2014**, *140*, 16.

[15] J. M. Turney, A. C. Simmonett, R. M. Parrish, E. G. Hohenstein, F. A. Evangelista, J. T. Fermann, B. J. Mintz, L. A. Burns, J. J. Wilke, M. L. Abrams, N. J. Russ, M. L. Leininger, C. L. Janssen, E. T. Seidl, W. D. Allen, H. F. Schaefer, R. A. King, E. F. Valeev, C. D. Sherrill, T. D. Crawford, *Wiley Interdiscip. Rev. Comput. Mol. Sci.* **2012**, *2*, 556–565.

Pentamer - 5 I

| | | | | | | | obs | o-c | error | blends o-c | wt | Notes |
|---|---|---|---|---|---|---|---|---|---|---|---|---|
| 1: | 3 | 1 | 2 | 2 | 0 | 2 | 2261.5707 | -0.0013 | 0.025 | | | |
| 2: | 4 | 0 | 4 | 3 | 1 | 3 | 2352.8413 | -0.0010 | 0.025 | | | |
| 3: | 4 | 1 | 4 | 3 | 1 | 3 | 2355.0441 | -0.0011 | 0.025 | | | |
| 4: | 4 | 0 | 4 | 3 | 0 | 3 | 2361.7401 | 0.0001 | 0.025 | | | |
| 5: | 4 | 1 | 4 | 3 | 0 | 3 | 2363.9425 | -0.0004 | 0.025 | | | |
| 6: | 3 | 3 | 1 | 2 | 2 | 0 | 2434.9927 | -0.0003 | 0.025 | | | |
| 7: | 3 | 3 | 0 | 2 | 2 | 0 | 2448.6892 | -0.0017 | 0.025 | | | |
| 8: | 3 | 3 | 1 | 2 | 2 | 1 | 2473.1529 | -0.0013 | 0.025 | | | |
| 9: | 4 | 1 | 3 | 3 | 2 | 2 | 2473.9902 | -0.0004 | 0.025 | | | |
| 10: | 3 | 3 | 0 | 2 | 2 | 1 | 2486.8511 | -0.0010 | 0.025 | | | |
| 11: | 4 | 2 | 3 | 3 | 2 | 2 | 2528.3062 | -0.0048 | 0.025 | | | |
| 12: | 4 | 1 | 3 | 3 | 1 | 2 | 2597.0208 | -0.0007 | 0.025 | | | |
| 13: | 4 | 3 | 2 | 3 | 3 | 1 | 2610.9127 | -0.0158 | 0.025 | | | |
| 14: | 4 | 2 | 3 | 3 | 1 | 2 | 2651.3416 | -0.0003 | 0.025 | | | |
| 15: | 4 | 2 | 2 | 3 | 2 | 1 | 2722.4107 | 0.0109 | 0.025 | | | |
| 16: | 5 | 0 | 5 | 4 | 1 | 4 | 2919.9260 | -0.0024 | 0.025 | | | |
| 17: | 5 | 1 | 5 | 4 | 1 | 4 | 2920.4186 | 0.0000 | 0.025 | | | |
| 18: | 5 | 0 | 5 | 4 | 0 | 4 | 2922.1294 | -0.0019 | 0.025 | | | |
| 19: | 5 | 1 | 5 | 4 | 0 | 4 | 2922.6201 | -0.0013 | 0.025 | | | |
| 20: | 4 | 2 | 2 | 3 | 1 | 2 | 2998.4237 | -0.0015 | 0.025 | | | |
| 21: | 4 | 3 | 2 | 3 | 2 | 1 | 3011.1796 | 0.0004 | 0.025 | | | |
| 22: | 4 | 1 | 3 | 3 | 0 | 3 | 3053.5157 | -0.0041 | 0.025 | | | |
| 23: | 5 | 2 | 3 | 4 | 3 | 2 | 3078.0233 | 0.0054 | 0.025 | | | |
| 24: | 4 | 3 | 1 | 3 | 2 | 1 | 3090.4225 | -0.0021 | 0.025 | | | |
| 25: | 5 | 1 | 4 | 4 | 2 | 3 | 3098.7966 | -0.0015 | 0.025 | | | |
| 26: | 5 | 1 | 4 | 4 | 1 | 3 | 3153.1164 | -0.0021 | 0.025 | | | |
| 27: | 4 | 3 | 2 | 3 | 2 | 2 | 3164.1718 | -0.0020 | 0.025 | | | |
| 28: | 5 | 2 | 4 | 4 | 1 | 3 | 3171.2179 | -0.0010 | 0.025 | | | |
| 29: | 4 | 3 | 1 | 3 | 2 | 2 | 3243.4176 | -0.0016 | 0.025 | | | |
| 30: | 5 | 3 | 3 | 4 | 3 | 2 | 3247.1477 | -0.0012 | 0.025 | | | |
| 31: | 4 | 4 | 1 | 3 | 3 | 0 | 3304.0698 | -0.0019 | 0.025 | | | |
| 32: | 4 | 4 | 0 | 3 | 3 | 0 | 3308.1062 | -0.0016 | 0.025 | | | |
| 33: | 4 | 4 | 1 | 3 | 3 | 1 | 3317.7690 | -0.0007 | 0.025 | | | |
| 34: | 4 | 4 | 0 | 3 | 3 | 1 | 3321.8045 | -0.0013 | 0.025 | | | |
| 35: | 5 | 2 | 3 | 4 | 2 | 2 | 3366.7963 | -0.0008 | 0.025 | | | |
| 36: | 5 | 3 | 2 | 4 | 3 | 1 | 3397.7755 | -0.0023 | 0.025 | | | |
| 37: | 4 | 2 | 2 | 3 | 1 | 3 | 3446.0384 | 0.0124 | 0.025 | | | |
| 38: | 6 | 0 | 6 | 5 | 1 | 5 | 3483.9435 | -0.0019 | 0.025 | | | |
| 39: | 6 | 1 | 6 | 5 | 1 | 5 | 3484.0450 | -0.0026 | 0.025 | | | |
| 40: | 6 | 0 | 6 | 5 | 0 | 5 | 3484.4341 | -0.0014 | 0.025 | | | |
| 41: | 6 | 1 | 6 | 5 | 0 | 5 | 3484.5371 | -0.0006 | 0.025 | | | |
| 42: | 5 | 3 | 3 | 4 | 2 | 2 | 3535.9281 | -0.0001 | 0.025 | | | |
| 43: | 6 | 1 | 5 | 5 | 2 | 4 | 3686.0680 | -0.0024 | 0.025 | | | |
| 44: | 6 | 2 | 5 | 5 | 2 | 4 | 3691.1004 | -0.0019 | 0.025 | | | |
| 45: | 6 | 1 | 5 | 5 | 1 | 4 | 3704.1679 | -0.0029 | 0.025 | | | |
| 46: | 6 | 2 | 5 | 5 | 1 | 4 | 3709.2005 | -0.0022 | 0.025 | | | |
| 47: | 5 | 3 | 2 | 4 | 2 | 2 | 3765.7999 | -0.0027 | 0.025 | | | |
| 48: | 5 | 2 | 3 | 4 | 1 | 3 | 3768.1980 | -0.0029 | 0.025 | | | |
| 49: | 6 | 2 | 4 | 5 | 3 | 3 | 3785.1008 | -0.0014 | 0.025 | | | |
| 50: | 5 | 1 | 4 | 4 | 0 | 4 | 3844.8968 | -0.0016 | 0.025 | | | |
| 51: | 5 | 2 | 4 | 4 | 1 | 4 | 3860.7929 | -0.0030 | 0.025 | | | |
| 52: | 6 | 3 | 4 | 5 | 3 | 3 | 3861.1271 | -0.0028 | 0.025 | | | |
| 53: | 5 | 3 | 3 | 4 | 2 | 3 | 3883.0091 | -0.0025 | 0.025 | | | |
| 54: | 5 | 4 | 2 | 4 | 3 | 1 | 3910.3346 | -0.0030 | 0.025 | | | |
| 55: | 5 | 4 | 1 | 4 | 3 | 1 | 3942.1546 | -0.0016 | 0.025 | | | |
| 56: | 6 | 2 | 4 | 5 | 2 | 3 | 3954.2314 | -0.0018 | 0.025 | | | |
| 57: | 5 | 4 | 2 | 4 | 3 | 2 | 3989.5799 | -0.0032 | 0.025 | | | |
| 58: | 6 | 3 | 4 | 5 | 2 | 3 | 4030.2596 | -0.0013 | 0.025 | | | |
| 59: | 7 | 0 | 7 | 6 | 1 | 6 | 4047.1874 | -0.0045 | 0.025 | -0.0147 | 0.50 | |
| 60: | 7 | 1 | 7 | 6 | 1 | 6 | 4047.1874 | -0.0249 | 0.025 | -0.0147 | 0.50 | |
| 61: | 7 | 0 | 7 | 6 | 0 | 6 | 4047.3165 | 0.0224 | 0.025 | 0.0122 | 0.50 | |
| 62: | 7 | 1 | 7 | 6 | 0 | 6 | 4047.3165 | 0.0020 | 0.025 | 0.0122 | 0.50 | |
| 63: | 6 | 3 | 3 | 5 | 3 | 2 | 4092.7159 | -0.0032 | 0.025 | | | |
| 64: | 5 | 3 | 2 | 4 | 2 | 3 | 4112.8819 | -0.0042 | 0.025 | | | |

| | | | | | | | | | | | |
|---|---|---|---|---|---|---|---|---|---|---|---|
| 65: | 5 | 5 | 1 | 4 | 4 | 0 | 4163.7786 | -0.0032 | 0.025 | | |
| 66: | 5 | 5 | 0 | 4 | 4 | 0 | 4164.8456 | -0.0025 | 0.025 | | |
| 67: | 5 | 5 | 1 | 4 | 4 | 1 | 4167.8142 | -0.0037 | 0.025 | | |
| 68: | 5 | 5 | 0 | 4 | 4 | 1 | 4168.8821 | -0.0021 | 0.025 | | |
| 69: | 7 | 1 | 6 | 6 | 2 | 5 | 4256.6230 | -0.0027 | 0.025 | | |
| 70: | 7 | 2 | 6 | 6 | 2 | 5 | 4257.8759 | -0.0032 | 0.025 | | |
| 71: | 7 | 2 | 6 | 6 | 1 | 5 | 4262.9092 | -0.0020 | 0.025 | | |
| 72: | 7 | 3 | 4 | 6 | 4 | 3 | 4372.9873 | -0.0020 | 0.025 | | |
| 73: | 7 | 2 | 5 | 6 | 3 | 4 | 4426.3287 | -0.0032 | 0.025 | | |
| 74: | 6 | 4 | 3 | 5 | 3 | 2 | 4453.7361 | -0.0026 | 0.025 | | |
| 75: | 6 | 3 | 3 | 5 | 2 | 3 | 4491.7215 | -0.0031 | 0.025 | | |
| 76: | 7 | 2 | 5 | 6 | 2 | 4 | 4502.3574 | -0.0023 | 0.025 | | |
| 77: | 7 | 3 | 5 | 6 | 2 | 4 | 4529.1697 | 0.0003 | 0.025 | | |
| 78: | 6 | 2 | 4 | 5 | 1 | 4 | 4569.3116 | -0.0041 | 0.025 | | |
| 79: | 6 | 4 | 2 | 5 | 3 | 2 | 4577.4714 | -0.0040 | 0.025 | | |
| 80: | 7 | 4 | 4 | 6 | 4 | 3 | 4581.8891 | -0.0038 | 0.025 | | |
| 81: | 8 | 0 | 8 | 7 | 1 | 7 | 4610.2748 | 0.0083 | 0.025 | -0.0038 | 0.25 |
| 82: | 8 | 1 | 8 | 7 | 1 | 7 | 4610.2748 | 0.0044 | 0.025 | -0.0038 | 0.25 |
| 83: | 8 | 0 | 8 | 7 | 0 | 7 | 4610.2748 | -0.0120 | 0.025 | -0.0038 | 0.25 |
| 84: | 8 | 1 | 8 | 7 | 0 | 7 | 4610.2748 | -0.0159 | 0.025 | -0.0038 | 0.25 |
| 85: | 7 | 5 | 3 | 6 | 5 | 2 | 4615.2599 | -0.0047 | 0.025 | | |
| 86: | 6 | 1 | 5 | 5 | 0 | 5 | 4626.9476 | 0.0097 | 0.025 | | |
| 87: | 6 | 3 | 4 | 5 | 2 | 4 | 4627.2404 | -0.0026 | 0.025 | | |
| 88: | 6 | 2 | 5 | 5 | 1 | 5 | 4631.4781 | -0.0016 | 0.025 | | |
| 89: | 7 | 5 | 2 | 6 | 5 | 1 | 4658.0165 | 0.0098 | 0.025 | | |
| 90: | 6 | 4 | 3 | 5 | 3 | 3 | 4683.6089 | -0.0043 | 0.025 | | |
| 91: | 7 | 3 | 4 | 6 | 3 | 3 | 4734.0063 | -0.0026 | 0.025 | | |
| 92: | 7 | 4 | 3 | 6 | 4 | 2 | 4766.8946 | -0.0032 | 0.025 | | |
| 93: | 6 | 5 | 2 | 5 | 4 | 1 | 4796.7219 | -0.0045 | 0.025 | | |
| 94: | 8 | 1 | 7 | 7 | 2 | 6 | 4821.5434 | -0.0033 | 0.025 | | |
| 95: | 8 | 2 | 7 | 7 | 2 | 6 | 4821.8344 | -0.0026 | 0.025 | | |
| 96: | 8 | 1 | 7 | 7 | 1 | 6 | 4822.7972 | -0.0030 | 0.025 | | |
| 97: | 8 | 2 | 7 | 7 | 1 | 6 | 4823.0883 | -0.0022 | 0.025 | | |
| 98: | 6 | 5 | 2 | 5 | 4 | 2 | 4828.5401 | -0.0049 | 0.025 | | |
| 99: | 6 | 5 | 1 | 5 | 4 | 2 | 4839.1548 | -0.0041 | 0.025 | | |
| 100: | 8 | 4 | 4 | 7 | 5 | 3 | 4853.7125 | -0.0033 | 0.025 | | |
| 101: | 7 | 4 | 4 | 6 | 3 | 3 | 4942.9094 | -0.0030 | 0.025 | | |
| 102: | 6 | 6 | 0 | 5 | 5 | 0 | 5019.2639 | -0.0022 | 0.025 | | |
| 103: | 6 | 6 | 1 | 5 | 5 | 1 | 5020.0661 | -0.0027 | 0.025 | | |
| 104: | 6 | 6 | 0 | 5 | 5 | 1 | 5020.3302 | -0.0021 | 0.025 | | |
| 105: | 8 | 2 | 6 | 7 | 3 | 5 | 5021.1136 | -0.0051 | 0.025 | | |
| 106: | 8 | 3 | 6 | 7 | 3 | 5 | 5029.1307 | -0.0028 | 0.025 | | |
| 107: | 8 | 3 | 6 | 7 | 2 | 5 | 5055.9393 | -0.0039 | 0.025 | | |
| 108: | 6 | 3 | 3 | 5 | 2 | 4 | 5088.7014 | -0.0052 | 0.025 | | |
| 109: | 8 | 3 | 5 | 7 | 4 | 4 | 5103.5952 | -0.0030 | 0.025 | | |
| 110: | 8 | 4 | 5 | 7 | 4 | 4 | 5198.6964 | -0.0034 | 0.025 | | |
| 111: | 7 | 4 | 3 | 6 | 3 | 3 | 5251.6495 | -0.0047 | 0.025 | | |
| 112: | 9 | 5 | 4 | 8 | 6 | 3 | 5252.1732 | 0.0056 | 0.025 | | |
| 113: | 7 | 3 | 4 | 6 | 2 | 4 | 5271.4964 | -0.0039 | 0.025 | | |
| 114: | 8 | 6 | 3 | 7 | 6 | 2 | 5276.7852 | -0.0024 | 0.025 | | |
| 115: | 8 | 5 | 4 | 7 | 5 | 3 | 5278.0520 | -0.0041 | 0.025 | | |
| 116: | 8 | 3 | 5 | 7 | 3 | 4 | 5312.4980 | -0.0038 | 0.025 | | |
| 117: | 7 | 2 | 5 | 6 | 1 | 5 | 5367.5008 | -0.0038 | 0.025 | | |
| 118: | 7 | 5 | 3 | 6 | 4 | 2 | 5378.8896 | -0.0044 | 0.025 | | |
| 119: | 9 | 1 | 8 | 8 | 2 | 7 | 5384.9049 | -0.0033 | 0.025 | | |
| 120: | 9 | 2 | 8 | 8 | 2 | 7 | 5384.9744 | 0.0023 | 0.025 | | |
| 121: | 9 | 1 | 8 | 8 | 1 | 7 | 5385.1827 | -0.0159 | 0.025 | | |
| 122: | 9 | 2 | 8 | 8 | 1 | 7 | 5385.2613 | -0.0011 | 0.025 | | |
| 123: | 7 | 3 | 5 | 6 | 2 | 5 | 5389.2782 | -0.0041 | 0.025 | | |
| 124: | 8 | 5 | 3 | 7 | 5 | 2 | 5396.3142 | -0.0031 | 0.025 | | |
| 125: | 7 | 1 | 6 | 6 | 0 | 6 | 5404.1575 | -0.0025 | 0.025 | | |
| 126: | 7 | 4 | 4 | 6 | 3 | 4 | 5404.3714 | -0.0047 | 0.025 | | |
| 127: | 7 | 2 | 6 | 6 | 1 | 6 | 5405.3073 | -0.0040 | 0.025 | | |
| 128: | 8 | 4 | 5 | 7 | 3 | 4 | 5407.6001 | -0.0032 | 0.025 | | |
| 129: | 7 | 5 | 2 | 6 | 4 | 2 | 5432.2455 | -0.0044 | 0.025 | | |
| 130: | 8 | 4 | 4 | 7 | 4 | 3 | 5465.7078 | -0.0041 | 0.025 | | |
| 131: | 6 | 4 | 3 | 5 | 1 | 4 | 5467.8211 | -0.0055 | 0.025 | | |
| 132: | 7 | 5 | 3 | 6 | 4 | 3 | 5502.6255 | -0.0053 | 0.025 | | |
| 133: | 7 | 5 | 2 | 6 | 4 | 3 | 5555.9826 | -0.0040 | 0.025 | | |
| 134: | 9 | 2 | 7 | 8 | 3 | 6 | 5594.3653 | -0.0038 | 0.025 | | |

| | | | | | | | | | | | |
|---|---|---|---|---|---|---|---|---|---|---|---|
| 135: | 9 | 2 | 7 | 8 | 2 | 6 | 5602.3798 | -0.0042 | 0.025 | | |
| 136: | 9 | 3 | 7 | 8 | 2 | 6 | 5604.5355 | -0.0029 | 0.025 | | |
| 137: | 7 | 6 | 2 | 6 | 5 | 1 | 5665.2754 | -0.0054 | 0.025 | | |
| 138: | 7 | 6 | 1 | 6 | 5 | 1 | 5668.4287 | -0.0050 | 0.025 | | |
| 139: | 7 | 6 | 2 | 6 | 5 | 2 | 5675.8900 | -0.0047 | 0.025 | | |
| 140: | 9 | 4 | 5 | 8 | 5 | 4 | 5677.8208 | -0.0034 | 0.025 | | |
| 141: | 7 | 6 | 1 | 6 | 5 | 2 | 5679.0431 | -0.0045 | 0.025 | | |
| 142: | 7 | 4 | 3 | 6 | 3 | 4 | 5713.1117 | -0.0061 | 0.025 | | |
| 143: | 10 | 0 | 10 | 9 | 1 | 9 | 5736.3250 | -0.0018 | 0.025 | -0.0023 | 0.25 |
| 144: | 10 | 1 | 10 | 9 | 0 | 9 | 5736.3250 | -0.0027 | 0.025 | -0.0023 | 0.25 |
| 145: | 10 | 0 | 10 | 9 | 0 | 9 | 5736.3250 | -0.0026 | 0.025 | -0.0023 | 0.25 |
| 146: | 10 | 1 | 10 | 9 | 1 | 9 | 5736.3250 | -0.0020 | 0.025 | -0.0023 | 0.25 |
| 147: | 9 | 3 | 6 | 8 | 4 | 5 | 5757.4735 | -0.0036 | 0.025 | | |
| 148: | 9 | 4 | 6 | 8 | 4 | 5 | 5792.2963 | -0.0036 | 0.025 | | |
| 149: | 9 | 3 | 6 | 8 | 3 | 5 | 5852.5729 | -0.0058 | 0.025 | | |
| 150: | 7 | 7 | 1 | 6 | 6 | 0 | 5872.7486 | -0.0044 | 0.025 | | |
| 151: | 7 | 7 | 0 | 6 | 6 | 0 | 5872.8084 | -0.0069 | 0.025 | -0.0069 | 0.50 |
| 152: | 7 | 7 | 0 | 6 | 6 | 0 | 5872.8084 | -0.0069 | 0.025 | -0.0069 | 0.50 |
| 153: | 7 | 7 | 1 | 6 | 6 | 1 | 5873.0091 | -0.0075 | 0.025 | | |
| 154: | 7 | 7 | 0 | 6 | 6 | 1 | 5873.0760 | -0.0029 | 0.025 | | |
| 155: | 9 | 4 | 6 | 8 | 3 | 5 | 5887.3981 | -0.0033 | 0.025 | | |
| 156: | 8 | 5 | 4 | 7 | 4 | 3 | 5890.0486 | -0.0037 | 0.025 | | |
| 157: | 9 | 5 | 5 | 8 | 5 | 4 | 5921.4181 | -0.0035 | 0.025 | | |
| 158: | 9 | 7 | 3 | 8 | 7 | 2 | 5932.2269 | -0.0049 | 0.025 | | |
| 159: | 9 | 7 | 2 | 8 | 7 | 1 | 5937.5861 | -0.0048 | 0.025 | | |
| 160: | 10 | 1 | 9 | 9 | 2 | 8 | 5947.8956 | -0.0147 | 0.025 | -0.0215 | 0.50 |
| 161: | 10 | 2 | 9 | 9 | 2 | 8 | 5947.8956 | -0.0282 | 0.025 | -0.0215 | 0.50 |
| 162: | 10 | 1 | 9 | 9 | 1 | 8 | 5947.9897 | 0.0155 | 0.025 | 0.0088 | 0.50 |
| 163: | 10 | 2 | 9 | 9 | 1 | 8 | 5947.9897 | 0.0020 | 0.025 | 0.0088 | 0.50 |
| 164: | 9 | 6 | 4 | 8 | 6 | 3 | 5953.5293 | 0.0062 | 0.025 | | |
| 165: | 8 | 4 | 4 | 7 | 3 | 4 | 5983.3531 | -0.0042 | 0.025 | | |
| 166: | 9 | 6 | 3 | 8 | 6 | 2 | 6012.1784 | -0.0053 | 0.025 | | |
| 167: | 8 | 5 | 3 | 7 | 4 | 3 | 6061.6641 | -0.0053 | 0.025 | | |
| 168: | 8 | 3 | 5 | 7 | 2 | 5 | 6081.6380 | -0.0043 | 0.025 | | |
| 169: | 9 | 4 | 5 | 8 | 4 | 4 | 6102.1615 | -0.0030 | 0.025 | | |
| 170: | 7 | 3 | 4 | 6 | 2 | 5 | 6131.6017 | -0.0114 | 0.025 | | |
| 171: | 10 | 5 | 5 | 9 | 6 | 4 | 6138.9954 | -0.0018 | 0.025 | | |
| 172: | 9 | 5 | 4 | 8 | 5 | 3 | 6139.9086 | -0.0035 | 0.025 | | |
| 173: | 8 | 4 | 5 | 7 | 3 | 5 | 6149.9299 | -0.0044 | 0.025 | | |
| 174: | 8 | 2 | 6 | 7 | 1 | 6 | 6153.7716 | -0.0036 | 0.025 | | |
| 175: | 8 | 3 | 6 | 7 | 2 | 6 | 6160.5318 | -0.0048 | 0.025 | | |
| 176: | 10 | 2 | 8 | 9 | 2 | 7 | 6162.0801 | -0.0042 | 0.025 | | |
| 177: | 10 | 3 | 8 | 9 | 2 | 7 | 6162.6203 | -0.0019 | 0.025 | | |
| 178: | 8 | 1 | 7 | 7 | 0 | 7 | 6179.6668 | 0.0005 | 0.025 | | |
| 179: | 8 | 2 | 7 | 7 | 1 | 7 | 6179.9298 | -0.0063 | 0.025 | | |
| 180: | 8 | 5 | 4 | 7 | 4 | 4 | 6198.7887 | -0.0054 | 0.025 | | |
| 181: | 8 | 6 | 3 | 7 | 5 | 2 | 6284.0568 | -0.0050 | 0.025 | | |
| 182: | 11 | 0 | 11 | 10 | 1 | 10 | 6299.3366 | -0.0041 | 0.025 | -0.0042 | 0.25 |
| 183: | 11 | 1 | 11 | 10 | 1 | 10 | 6299.3366 | -0.0041 | 0.025 | -0.0042 | 0.25 |
| 184: | 11 | 0 | 11 | 10 | 0 | 10 | 6299.3366 | -0.0042 | 0.025 | -0.0042 | 0.25 |
| 185: | 11 | 1 | 11 | 10 | 0 | 10 | 6299.3366 | -0.0042 | 0.025 | -0.0042 | 0.25 |
| 186: | 8 | 6 | 2 | 7 | 5 | 2 | 6303.3772 | -0.0054 | 0.025 | | |
| 187: | 8 | 6 | 3 | 7 | 5 | 3 | 6337.4122 | -0.0054 | 0.025 | | |
| 188: | 7 | 4 | 4 | 6 | 1 | 5 | 6345.5454 | -0.0033 | 0.025 | | |
| 189: | 8 | 6 | 2 | 7 | 5 | 3 | 6356.7334 | -0.0050 | 0.025 | | |
| 190: | 10 | 3 | 7 | 9 | 4 | 6 | 6358.0235 | -0.0038 | 0.025 | | |
| 191: | 10 | 4 | 7 | 9 | 4 | 6 | 6368.9766 | -0.0039 | 0.025 | | |
| 192: | 8 | 5 | 3 | 7 | 4 | 4 | 6370.4062 | -0.0049 | 0.025 | | |
| 193: | 7 | 5 | 3 | 6 | 2 | 4 | 6401.1334 | -0.0083 | 0.025 | | |
| 194: | 11 | 1 | 10 | 10 | 2 | 9 | 6510.8532 | 0.0038 | 0.025 | -0.0043 | 0.25 |
| 195: | 11 | 2 | 10 | 10 | 2 | 9 | 6510.8532 | 0.0010 | 0.025 | -0.0043 | 0.25 |
| 196: | 11 | 1 | 10 | 10 | 1 | 9 | 6510.8532 | -0.0096 | 0.025 | -0.0043 | 0.25 |
| 197: | 11 | 2 | 10 | 10 | 1 | 9 | 6510.8532 | -0.0124 | 0.025 | -0.0043 | 0.25 |
| 198: | 8 | 7 | 2 | 7 | 6 | 1 | 6524.0135 | -0.0063 | 0.025 | | |
| 199: | 8 | 7 | 1 | 7 | 6 | 1 | 6524.8835 | -0.0044 | 0.025 | | |
| 200: | 8 | 7 | 2 | 7 | 6 | 2 | 6527.1667 | -0.0059 | 0.025 | | |
| 201: | 8 | 7 | 1 | 7 | 6 | 2 | 6528.0373 | -0.0033 | 0.025 | | |
| 202: | 10 | 5 | 6 | 9 | 5 | 5 | 6539.5813 | -0.0036 | 0.025 | | |
| 203: | 10 | 8 | 2 | 9 | 8 | 1 | 6587.0903 | -0.0094 | 0.025 | | |
| 204: | 10 | 7 | 4 | 9 | 7 | 3 | 6615.2324 | -0.0026 | 0.025 | | |

| | | | | | | | | | | | |
|---|---|---|---|---|---|---|---|---|---|---|---|
| 205: | 10 | 6 | 5 | 9 | 6 | 4 | 6619.4130 | -0.0015 | 0.025 | | |
| 206: | 10 | 7 | 3 | 9 | 7 | 2 | 6639.0082 | -0.0050 | 0.025 | | |
| 207: | 10 | 4 | 6 | 9 | 4 | 5 | 6671.2970 | -0.0039 | 0.025 | | |
| 208: | 11 | 2 | 9 | 10 | 3 | 8 | 6723.2430 | -0.0054 | 0.025 | | |
| 209: | 11 | 2 | 9 | 10 | 2 | 8 | 6723.7809 | -0.0053 | 0.025 | | |
| 210: | 11 | 3 | 9 | 10 | 2 | 8 | 6723.9086 | -0.0048 | 0.025 | | |
| 211: | 8 | 4 | 4 | 7 | 3 | 5 | 6725.6866 | -0.0016 | 0.025 | | |
| 212: | 8 | 8 | 1 | 7 | 7 | 1 | 6726.1487 | 0.0123 | 0.025 | 0.0052 | 0.50 |
| 213: | 8 | 8 | 0 | 7 | 7 | 1 | 6726.1487 | -0.0018 | 0.025 | 0.0052 | 0.50 |
| 214: | 9 | 5 | 4 | 8 | 4 | 4 | 6735.8651 | -0.0043 | 0.025 | | |
| 215: | 10 | 6 | 4 | 9 | 6 | 3 | 6764.2919 | -0.0038 | 0.025 | | |
| 216: | 9 | 4 | 5 | 8 | 3 | 5 | 6773.0160 | -0.0040 | 0.025 | | |
| 217: | 10 | 5 | 6 | 9 | 4 | 5 | 6783.1781 | -0.0044 | 0.025 | | |
| 218: | 10 | 5 | 5 | 9 | 5 | 4 | 6840.3475 | -0.0052 | 0.025 | | |
| 219: | 9 | 6 | 4 | 8 | 5 | 3 | 6841.2619 | -0.0055 | 0.025 | | |
| 220: | 12 | 0 | 12 | 11 | 1 | 11 | 6862.3393 | -0.0041 | 0.025 | -0.0041 | 0.25 |
| 221: | 12 | 1 | 12 | 11 | 1 | 11 | 6862.3393 | -0.0041 | 0.025 | -0.0041 | 0.25 |
| 222: | 12 | 0 | 12 | 11 | 0 | 11 | 6862.3393 | -0.0041 | 0.025 | -0.0041 | 0.25 |
| 223: | 12 | 1 | 12 | 11 | 0 | 11 | 6862.3393 | -0.0041 | 0.025 | -0.0041 | 0.25 |
| 224: | 9 | 3 | 6 | 8 | 2 | 6 | 6886.2872 | -0.0055 | 0.025 | | |
| 225: | 9 | 4 | 6 | 8 | 3 | 6 | 6913.0952 | -0.0054 | 0.025 | | |
| 226: | 9 | 6 | 3 | 8 | 5 | 3 | 6919.2432 | -0.0057 | 0.025 | | |
| 227: | 9 | 5 | 5 | 8 | 4 | 5 | 6921.5101 | -0.0057 | 0.025 | | |
| 228: | 11 | 3 | 8 | 10 | 4 | 7 | 6933.2907 | -0.0047 | 0.025 | | |
| 229: | 9 | 3 | 7 | 8 | 2 | 7 | 6935.2163 | -0.0069 | 0.025 | | |
| 230: | 11 | 4 | 8 | 10 | 4 | 7 | 6936.4079 | -0.0005 | 0.025 | | |
| 231: | 11 | 3 | 8 | 10 | 3 | 7 | 6944.2428 | -0.0060 | 0.025 | | |
| 232: | 11 | 4 | 8 | 10 | 3 | 7 | 6947.3566 | -0.0052 | 0.025 | | |
| 233: | 11 | 5 | 6 | 10 | 6 | 5 | 6990.3115 | -0.0027 | 0.025 | | |
| 234: | 9 | 6 | 4 | 8 | 5 | 4 | 7012.8793 | -0.0053 | 0.025 | | |
| 235: | 12 | 1 | 11 | 11 | 2 | 10 | 7073.7816 | -0.0023 | 0.025 | -0.0040 | 0.25 |
| 236: | 12 | 2 | 11 | 11 | 2 | 10 | 7073.7816 | -0.0028 | 0.025 | -0.0040 | 0.25 |
| 237: | 12 | 1 | 11 | 11 | 1 | 10 | 7073.7816 | -0.0051 | 0.025 | -0.0040 | 0.25 |
| 238: | 12 | 2 | 11 | 11 | 1 | 10 | 7073.7816 | -0.0056 | 0.025 | -0.0040 | 0.25 |
| 239: | 9 | 6 | 3 | 8 | 5 | 4 | 7090.8604 | -0.0055 | 0.025 | | |
| 240: | 11 | 4 | 7 | 10 | 5 | 6 | 7091.6088 | -0.0037 | 0.025 | | |
| 241: | 11 | 5 | 7 | 10 | 5 | 6 | 7133.7002 | -0.0057 | 0.025 | | |
| 242: | 9 | 7 | 3 | 8 | 6 | 2 | 7163.2897 | -0.0064 | 0.025 | | |
| 243: | 9 | 7 | 2 | 8 | 6 | 2 | 7169.5170 | -0.0064 | 0.025 | | |
| 244: | 8 | 5 | 4 | 7 | 2 | 5 | 7176.8344 | -0.0037 | 0.025 | | |
| 245: | 9 | 7 | 3 | 8 | 6 | 3 | 7182.6099 | -0.0068 | 0.025 | | |
| 246: | 9 | 7 | 2 | 8 | 6 | 3 | 7188.8374 | -0.0067 | 0.025 | | |
| 247: | 11 | 5 | 7 | 10 | 4 | 6 | 7245.5820 | -0.0054 | 0.025 | | |
| 248: | 11 | 8 | 4 | 10 | 8 | 3 | 7269.8760 | -0.0073 | 0.025 | | |
| 249: | 11 | 8 | 3 | 10 | 8 | 2 | 7278.3070 | -0.0088 | 0.025 | | |
| 250: | 8 | 4 | 5 | 7 | 1 | 6 | 7282.5827 | -0.0081 | 0.025 | | |
| 251: | 11 | 7 | 5 | 10 | 7 | 4 | 7295.9154 | -0.0065 | 0.025 | | |
| 252: | 9 | 5 | 4 | 8 | 4 | 5 | 7311.6158 | -0.0075 | 0.025 | | |
| 253: | 10 | 6 | 5 | 9 | 5 | 4 | 7320.7640 | -0.0059 | 0.025 | | |
| 254: | 11 | 7 | 4 | 10 | 7 | 3 | 7371.4576 | -0.0208 | 0.025 | | |
| 255: | 9 | 8 | 2 | 8 | 7 | 2 | 7379.8738 | -0.0091 | 0.025 | | |
| 256: | 9 | 8 | 1 | 8 | 7 | 2 | 7380.1056 | -0.0038 | 0.025 | | |
| 257: | 13 | 0 | 13 | 12 | 0 | 12 | 7425.3269 | -0.0065 | 0.025 | -0.0065 | 0.25 |
| 258: | 13 | 0 | 13 | 12 | 1 | 12 | 7425.3269 | -0.0065 | 0.025 | -0.0065 | 0.25 |
| 259: | 13 | 1 | 13 | 12 | 0 | 12 | 7425.3269 | -0.0065 | 0.025 | -0.0065 | 0.25 |
| 260: | 13 | 1 | 13 | 12 | 1 | 12 | 7425.3269 | -0.0065 | 0.025 | -0.0065 | 0.25 |
| 261: | 11 | 5 | 6 | 10 | 5 | 5 | 7470.7288 | -0.0027 | 0.025 | | |
| 262: | 10 | 5 | 5 | 9 | 4 | 5 | 7474.0531 | -0.0046 | 0.025 | | |
| 263: | 12 | 3 | 9 | 11 | 4 | 8 | 7499.2066 | -0.0070 | 0.025 | | |
| 264: | 12 | 4 | 9 | 11 | 4 | 8 | 7500.0264 | -0.0100 | 0.025 | | |
| 265: | 12 | 3 | 9 | 11 | 3 | 8 | 7502.3188 | -0.0078 | 0.025 | | |
| 266: | 12 | 4 | 9 | 11 | 3 | 8 | 7503.1460 | -0.0033 | 0.025 | | |
| 267: | 10 | 6 | 4 | 9 | 5 | 4 | 7543.6259 | -0.0067 | 0.025 | | |
| 268: | 9 | 9 | 1 | 8 | 8 | 0 | 7579.2639 | 0.0027 | 0.025 | -0.0059 | 0.25 |
| 269: | 9 | 9 | 0 | 8 | 8 | 0 | 7579.2639 | -0.0004 | 0.025 | -0.0059 | 0.25 |
| 270: | 9 | 9 | 1 | 8 | 8 | 1 | 7579.2639 | -0.0114 | 0.025 | -0.0059 | 0.25 |
| 271: | 9 | 9 | 0 | 8 | 8 | 1 | 7579.2639 | -0.0146 | 0.025 | -0.0059 | 0.25 |
| 272: | 10 | 4 | 6 | 9 | 3 | 6 | 7591.7374 | -0.0048 | 0.025 | | |
| 273: | 13 | 1 | 12 | 12 | 2 | 11 | 7636.7140 | -0.0055 | 0.025 | -0.0059 | 0.25 |
| 274: | 13 | 2 | 12 | 12 | 2 | 11 | 7636.7140 | -0.0056 | 0.025 | -0.0059 | 0.25 |

| | | | | | | | | | | | |
|---|---|---|---|---|---|---|---|---|---|---|---|
| 275: | 13 | 1 | 12 | 12 | 1 | 11 | 7636.7140 | -0.0061 | 0.025 | -0.0059 | 0.25 |
| 276: | 13 | 2 | 12 | 12 | 1 | 11 | 7636.7140 | -0.0062 | 0.025 | -0.0059 | 0.25 |
| 277: | 10 | 5 | 6 | 9 | 4 | 6 | 7668.7939 | -0.0071 | 0.025 | | |
| 278: | 10 | 3 | 7 | 9 | 2 | 7 | 7676.7544 | -0.0043 | 0.025 | | |
| 279: | 10 | 4 | 7 | 9 | 3 | 7 | 7685.5514 | -0.0062 | 0.025 | | |
| 280: | 10 | 2 | 8 | 9 | 1 | 8 | 7710.2447 | -0.0001 | 0.025 | | |
| 281: | 12 | 5 | 8 | 11 | 5 | 7 | 7710.3708 | -0.0057 | 0.025 | | |
| 282: | 10 | 3 | 8 | 9 | 2 | 8 | 7710.7110 | -0.0079 | 0.025 | | |
| 283: | 10 | 6 | 5 | 9 | 5 | 5 | 7710.8701 | -0.0073 | 0.025 | | |
| 284: | 10 | 1 | 9 | 9 | 0 | 9 | 7729.2511 | 0.0059 | 0.025 | -0.0004 | 0.50 |
| 285: | 10 | 2 | 9 | 9 | 1 | 9 | 7729.2511 | -0.0067 | 0.025 | -0.0004 | 0.50 |
| 286: | 12 | 4 | 8 | 11 | 4 | 7 | 7738.7192 | 0.0027 | 0.025 | | |
| 287: | 11 | 6 | 6 | 10 | 5 | 5 | 7744.7282 | -0.0055 | 0.025 | | |
| 288: | 12 | 5 | 8 | 11 | 4 | 7 | 7752.4651 | -0.0047 | 0.025 | | |
| 289: | 12 | 5 | 7 | 11 | 6 | 6 | 7756.2934 | -0.0011 | 0.025 | | |
| 290: | 10 | 7 | 4 | 9 | 6 | 3 | 7766.3427 | -0.0047 | 0.025 | | |
| 291: | 10 | 7 | 3 | 9 | 6 | 3 | 7796.3463 | -0.0065 | 0.025 | | |
| 292: | 10 | 7 | 4 | 9 | 6 | 4 | 7844.3226 | -0.0062 | 0.025 | | |
| 293: | 13 | 2 | 11 | 12 | 3 | 10 | 7848.7963 | 0.0120 | 0.025 | -0.0055 | 0.25 |
| 294: | 13 | 3 | 11 | 12 | 3 | 10 | 7848.7963 | 0.0057 | 0.025 | -0.0055 | 0.25 |
| 295: | 13 | 2 | 11 | 12 | 2 | 10 | 7848.7963 | -0.0167 | 0.025 | -0.0055 | 0.25 |
| 296: | 13 | 3 | 11 | 12 | 2 | 10 | 7848.7963 | -0.0230 | 0.025 | -0.0055 | 0.25 |
| 297: | 10 | 7 | 3 | 9 | 6 | 4 | 7874.3284 | -0.0059 | 0.025 | | |
| 298: | 10 | 6 | 4 | 9 | 5 | 5 | 7933.7358 | -0.0043 | 0.025 | | |
| 299: | 12 | 7 | 6 | 11 | 7 | 5 | 7964.0394 | 0.0003 | 0.025 | | |
| 300: | 14 | 0 | 14 | 13 | 0 | 13 | 7988.3016 | -0.0073 | 0.025 | -0.0074 | 0.25 |
| 301: | 14 | 1 | 14 | 13 | 1 | 13 | 7988.3016 | -0.0073 | 0.025 | -0.0074 | 0.25 |
| 302: | 14 | 1 | 14 | 13 | 0 | 13 | 7988.3016 | -0.0073 | 0.025 | -0.0074 | 0.25 |
| 303: | 14 | 0 | 14 | 13 | 1 | 13 | 7988.3016 | -0.0073 | 0.025 | -0.0074 | 0.25 |
| 304: | 10 | 8 | 3 | 9 | 7 | 2 | 8026.9011 | 0.0053 | 0.025 | | |
| 305: | 10 | 8 | 2 | 9 | 7 | 2 | 8028.7548 | 0.0046 | 0.025 | | |
| 306: | 10 | 8 | 3 | 9 | 7 | 3 | 8033.1292 | 0.0061 | 0.025 | | |
| 307: | 10 | 8 | 2 | 9 | 7 | 3 | 8034.9842 | 0.0066 | 0.025 | | |
| 308: | 9 | 5 | 5 | 8 | 2 | 6 | 8050.3362 | 0.0046 | 0.025 | | |
| 309: | 13 | 3 | 10 | 12 | 4 | 9 | 8062.3204 | 0.0002 | 0.025 | | |
| 310: | 13 | 4 | 10 | 12 | 3 | 9 | 8063.3557 | 0.0071 | 0.025 | | |
| 311: | 9 | 6 | 4 | 8 | 3 | 5 | 8108.0802 | -0.0002 | 0.025 | | |
| 312: | 12 | 6 | 7 | 11 | 5 | 6 | 8156.9324 | 0.0048 | 0.025 | | |
| 313: | 14 | 1 | 13 | 13 | 2 | 12 | 8199.6557 | 0.0039 | 0.025 | 0.0039 | 0.25 |
| 314: | 14 | 2 | 13 | 13 | 1 | 12 | 8199.6557 | 0.0038 | 0.025 | 0.0039 | 0.25 |
| 315: | 14 | 1 | 13 | 13 | 1 | 12 | 8199.6557 | 0.0038 | 0.025 | 0.0039 | 0.25 |
| 316: | 14 | 2 | 13 | 13 | 2 | 12 | 8199.6557 | 0.0039 | 0.025 | 0.0039 | 0.25 |
| 317: | 11 | 6 | 5 | 10 | 5 | 5 | 8218.9525 | 0.0065 | 0.025 | | |
| 318: | 10 | 9 | 2 | 9 | 8 | 1 | 8232.7809 | 0.0122 | 0.025 | -0.0161 | 0.50 |
| 319: | 10 | 9 | 1 | 9 | 8 | 1 | 8232.7809 | -0.0445 | 0.025 | -0.0161 | 0.50 |
| 320: | 10 | 9 | 2 | 9 | 8 | 2 | 8233.0488 | 0.0537 | 0.025 | 0.0253 | 0.50 |
| 321: | 10 | 9 | 1 | 9 | 8 | 2 | 8233.0488 | -0.0030 | 0.025 | 0.0253 | 0.50 |
| 322: | 13 | 4 | 9 | 12 | 5 | 8 | 8273.3549 | 0.0014 | 0.025 | | |
| 323: | 11 | 5 | 6 | 10 | 4 | 6 | 8273.4980 | 0.0097 | 0.025 | | |
| 324: | 13 | 5 | 9 | 12 | 5 | 8 | 8277.4400 | 0.0061 | 0.025 | | |
| 325: | 13 | 4 | 9 | 12 | 4 | 8 | 8287.1129 | 0.0061 | 0.025 | | |
| 326: | 13 | 5 | 9 | 12 | 4 | 8 | 8291.1953 | 0.0080 | 0.025 | | |
| 327: | 11 | 7 | 5 | 10 | 6 | 4 | 8297.9777 | 0.0040 | 0.025 | | |
| 328: | 13 | 6 | 7 | 12 | 7 | 6 | 8309.0489 | 0.0122 | 0.025 | | |
| 329: | 10 | 5 | 5 | 9 | 4 | 6 | 8359.6838 | 0.0075 | 0.025 | | |
| 330: | 11 | 4 | 7 | 10 | 3 | 7 | 8402.3933 | 0.0070 | 0.025 | | |
| 331: | 11 | 7 | 4 | 10 | 6 | 4 | 8403.5346 | -0.0009 | 0.025 | | |
| 332: | 14 | 2 | 12 | 13 | 3 | 11 | 8411.5557 | 0.0116 | 0.025 | 0.0078 | 0.25 |
| 333: | 14 | 3 | 12 | 13 | 3 | 11 | 8411.5557 | 0.0102 | 0.025 | 0.0078 | 0.25 |
| 334: | 14 | 2 | 12 | 13 | 2 | 11 | 8411.5557 | 0.0053 | 0.025 | 0.0078 | 0.25 |
| 335: | 14 | 3 | 12 | 13 | 2 | 11 | 8411.5557 | 0.0039 | 0.025 | 0.0078 | 0.25 |
| 336: | 13 | 5 | 8 | 12 | 6 | 7 | 8428.2521 | 0.0044 | 0.025 | | |
| 337: | 10 | 10 | 1 | 9 | 9 | 0 | 8432.3990 | 0.0086 | 0.025 | 0.0067 | 0.25 |
| 338: | 10 | 10 | 0 | 9 | 9 | 1 | 8432.3990 | 0.0047 | 0.025 | 0.0067 | 0.25 |
| 339: | 10 | 10 | 0 | 9 | 9 | 0 | 8432.3990 | 0.0079 | 0.025 | 0.0067 | 0.25 |
| 340: | 10 | 10 | 1 | 9 | 9 | 1 | 8432.3990 | 0.0054 | 0.025 | 0.0067 | 0.25 |
| 341: | 11 | 6 | 6 | 10 | 5 | 6 | 8435.6148 | 0.0058 | 0.025 | | |
| 342: | 11 | 3 | 8 | 10 | 2 | 8 | 8458.9290 | 0.0057 | 0.025 | | |
| 343: | 13 | 6 | 8 | 12 | 6 | 7 | 8476.9001 | 0.0200 | 0.025 | | |
| 344: | 11 | 1 | 10 | 10 | 0 | 10 | 8503.7790 | -0.0012 | 0.025 | -0.0026 | 0.50 |

| | | | | | | | | | | | | |
|---|---|---|---|---|---|---|---|---|---|---|---|---|
| 345: | 11 | 2 | 10 | 10 | 1 | 10 | 8503.7790 | -0.0038 | 0.025 | -0.0026 | 0.50 |
| 346: | 11 | 7 | 5 | 10 | 6 | 5 | 8520.8389 | 0.0026 | 0.025 | | |
| 347: | 13 | 6 | 8 | 12 | 5 | 7 | 8603.5171 | 0.0064 | 0.025 | | |
| 348: | 11 | 7 | 4 | 10 | 6 | 5 | 8626.4041 | 0.0058 | 0.025 | | |
| 349: | 11 | 8 | 4 | 10 | 7 | 3 | 8657.7714 | 0.0055 | 0.025 | | |
| 350: | 11 | 8 | 3 | 10 | 7 | 3 | 8668.0542 | 0.0014 | 0.025 | | |
| 351: | 11 | 8 | 3 | 10 | 7 | 4 | 8698.0632 | 0.0049 | 0.025 | | |
| 352: | 16 | 10 | 7 | 15 | 11 | 4 | 8711.2059 | 0.0175 | 0.025 | | |
| 353: | 12 | 7 | 6 | 11 | 6 | 5 | 8746.3512 | 0.0045 | 0.025 | | |
| 354: | 15 | 1 | 14 | 14 | 2 | 13 | 8762.5825 | 0.0064 | 0.025 | 0.0065 | 0.25 |
| 355: | 15 | 2 | 14 | 14 | 2 | 13 | 8762.5825 | 0.0064 | 0.025 | 0.0065 | 0.25 |
| 356: | 15 | 1 | 14 | 14 | 1 | 13 | 8762.5825 | 0.0064 | 0.025 | 0.0065 | 0.25 |
| 357: | 15 | 2 | 14 | 14 | 1 | 13 | 8762.5825 | 0.0064 | 0.025 | 0.0065 | 0.25 |
| 358: | 14 | 4 | 10 | 13 | 5 | 9 | 8839.3841 | 0.0043 | 0.025 | | |
| 359: | 14 | 5 | 10 | 13 | 4 | 9 | 8844.5983 | 0.0099 | 0.025 | | |
| 360: | 11 | 9 | 3 | 10 | 8 | 2 | 8883.8838 | 0.0043 | 0.025 | | |
| 361: | 11 | 9 | 2 | 10 | 8 | 2 | 8884.4049 | 0.0042 | 0.025 | | |
| 362: | 11 | 9 | 3 | 10 | 8 | 3 | 8885.7389 | 0.0050 | 0.025 | | |
| 363: | 11 | 9 | 2 | 10 | 8 | 3 | 8886.2611 | 0.0060 | 0.025 | | |
| 364: | 11 | 6 | 5 | 10 | 5 | 6 | 8909.8286 | 0.0074 | 0.025 | | |
| 365: | 16 | 9 | 8 | 15 | 10 | 5 | 8934.8240 | 0.0263 | 0.025 | | |
| 366: | 12 | 6 | 6 | 11 | 5 | 6 | 8964.2407 | 0.0056 | 0.025 | | |
| 367: | 15 | 2 | 13 | 14 | 3 | 12 | 8974.3371 | 0.0047 | 0.025 | 0.0040 | 0.25 |
| 368: | 15 | 3 | 13 | 14 | 3 | 12 | 8974.3371 | 0.0045 | 0.025 | 0.0040 | 0.25 |
| 369: | 15 | 2 | 13 | 14 | 2 | 12 | 8974.3371 | 0.0034 | 0.025 | 0.0040 | 0.25 |
| 370: | 15 | 3 | 13 | 14 | 2 | 12 | 8974.3371 | 0.0031 | 0.025 | 0.0040 | 0.25 |
| 371: | 10 | 5 | 6 | 9 | 2 | 7 | 8987.5377 | 0.0052 | 0.025 | | |
| 372: | 12 | 7 | 5 | 11 | 6 | 5 | 9023.8066 | 0.0060 | 0.025 | | |
| 373: | 14 | 5 | 9 | 13 | 6 | 8 | 9036.7075 | 0.0088 | 0.025 | | |
| 374: | 14 | 6 | 8 | 13 | 7 | 7 | 9088.5933 | 0.0096 | 0.025 | | |
| 375: | 12 | 5 | 7 | 11 | 4 | 7 | 9100.2970 | 0.0060 | 0.025 | | |
| 376: | 14 | 6 | 9 | 13 | 5 | 8 | 9101.7075 | 0.0067 | 0.025 | | |
| 377: | 16 | 0 | 16 | 15 | 0 | 15 | 9114.2177 | 0.0064 | 0.025 | 0.0064 | 0.25 |
| 378: | 16 | 0 | 16 | 15 | 1 | 15 | 9114.2177 | 0.0064 | 0.025 | 0.0064 | 0.25 |
| 379: | 16 | 1 | 16 | 15 | 0 | 15 | 9114.2177 | 0.0064 | 0.025 | 0.0064 | 0.25 |
| 380: | 16 | 1 | 16 | 15 | 1 | 15 | 9114.2177 | 0.0064 | 0.025 | 0.0064 | 0.25 |
| 381: | 10 | 7 | 4 | 9 | 4 | 5 | 9179.3873 | -0.0019 | 0.025 | | |
| 382: | 12 | 6 | 7 | 11 | 5 | 7 | 9184.8325 | 0.0041 | 0.025 | | |
| 383: | 12 | 4 | 8 | 11 | 3 | 8 | 9196.8589 | 0.0048 | 0.025 | | |
| 384: | 12 | 7 | 6 | 11 | 6 | 6 | 9220.5617 | 0.0028 | 0.025 | | |
| 385: | 10 | 4 | 7 | 9 | 1 | 8 | 9235.8733 | 0.0007 | 0.025 | | |
| 386: | 12 | 3 | 9 | 11 | 2 | 9 | 9237.4682 | 0.0046 | 0.025 | | |
| 387: | 12 | 4 | 9 | 11 | 3 | 9 | 9238.1565 | -0.0027 | 0.025 | | |
| 388: | 12 | 8 | 5 | 11 | 7 | 4 | 9243.8652 | 0.0016 | 0.025 | | |
| 389: | 12 | 1 | 11 | 11 | 0 | 11 | 9278.2283 | 0.0020 | 0.025 | 0.0018 | 0.50 |
| 390: | 12 | 2 | 11 | 11 | 1 | 11 | 9278.2283 | 0.0015 | 0.025 | 0.0018 | 0.50 |
| 391: | 11 | 11 | 1 | 10 | 10 | 0 | 9285.4851 | 0.0051 | 0.025 | 0.0047 | 0.25 |
| 392: | 11 | 11 | 0 | 10 | 10 | 1 | 9285.4851 | 0.0042 | 0.025 | 0.0047 | 0.25 |
| 393: | 11 | 11 | 1 | 10 | 10 | 1 | 9285.4851 | 0.0044 | 0.025 | 0.0047 | 0.25 |
| 394: | 11 | 11 | 0 | 10 | 10 | 0 | 9285.4851 | 0.0049 | 0.025 | 0.0047 | 0.25 |
| 395: | 12 | 8 | 4 | 11 | 7 | 4 | 9286.4807 | 0.0011 | 0.025 | | |
| 396: | 16 | 1 | 15 | 15 | 1 | 14 | 9325.4936 | 0.0048 | 0.025 | 0.0049 | 0.25 |
| 397: | 16 | 1 | 15 | 15 | 2 | 14 | 9325.4936 | 0.0048 | 0.025 | 0.0049 | 0.25 |
| 398: | 16 | 2 | 15 | 15 | 1 | 14 | 9325.4936 | 0.0048 | 0.025 | 0.0049 | 0.25 |
| 399: | 16 | 2 | 15 | 15 | 2 | 14 | 9325.4936 | 0.0048 | 0.025 | 0.0049 | 0.25 |
| 400: | 12 | 8 | 5 | 11 | 7 | 5 | 9349.4387 | 0.0131 | 0.025 | | |
| 401: | 16 | 9 | 7 | 15 | 10 | 6 | 9352.8517 | 0.0236 | 0.025 | | |
| 402: | 14 | 6 | 8 | 13 | 6 | 7 | 9389.2782 | 0.0132 | 0.025 | | |
| 403: | 12 | 8 | 4 | 11 | 7 | 5 | 9392.0471 | 0.0056 | 0.025 | | |
| 404: | 15 | 4 | 11 | 14 | 5 | 10 | 9402.1257 | 0.0017 | 0.025 | | |
| 405: | 15 | 5 | 11 | 14 | 4 | 10 | 9403.5499 | 0.0026 | 0.025 | | |
| 406: | 11 | 5 | 6 | 10 | 4 | 7 | 9461.4359 | 0.0087 | 0.025 | | |
| 407: | 12 | 7 | 5 | 11 | 6 | 6 | 9498.0188 | 0.0061 | 0.025 | | |
| 408: | 12 | 9 | 4 | 11 | 8 | 3 | 9527.4150 | 0.0028 | 0.025 | | |
| 409: | 14 | 7 | 8 | 13 | 6 | 7 | 9528.8366 | 0.0041 | 0.025 | | |
| 410: | 12 | 9 | 3 | 11 | 8 | 3 | 9530.6726 | 0.0055 | 0.025 | | |
| 411: | 16 | 2 | 14 | 15 | 3 | 13 | 9537.1398 | 0.0018 | 0.025 | 0.0016 | 0.25 |
| 412: | 16 | 3 | 14 | 15 | 3 | 13 | 9537.1398 | 0.0017 | 0.025 | 0.0016 | 0.25 |
| 413: | 16 | 2 | 14 | 15 | 2 | 13 | 9537.1398 | 0.0015 | 0.025 | 0.0016 | 0.25 |
| 414: | 16 | 3 | 14 | 15 | 2 | 13 | 9537.1398 | 0.0014 | 0.025 | 0.0016 | 0.25 |

| | | | | | | | | | | | |
|---|---|---|---|---|---|---|---|---|---|---|---|
| 415: | 17 | 9 | 9 | 16 | 10 | 6 | 9537.7039 | 0.0321 | 0.025 | 0.0185 | 0.50 |
| 416: | 12 | 9 | 4 | 11 | 8 | 4 | 9537.7039 | 0.0047 | 0.025 | 0.0185 | 0.50 |
| 417: | 12 | 9 | 3 | 11 | 8 | 4 | 9540.9581 | 0.0041 | 0.025 | | |
| 418: | 15 | 5 | 10 | 14 | 6 | 9 | 9614.4422 | 0.0066 | 0.025 | | |
| 419: | 15 | 6 | 10 | 14 | 5 | 9 | 9635.8384 | 0.0064 | 0.025 | | |
| 420: | 17 | 0 | 17 | 16 | 0 | 16 | 9677.1388 | 0.0033 | 0.025 | 0.0034 | 0.25 |
| 421: | 17 | 0 | 17 | 16 | 1 | 16 | 9677.1388 | 0.0033 | 0.025 | 0.0034 | 0.25 |
| 422: | 17 | 1 | 17 | 16 | 0 | 16 | 9677.1388 | 0.0033 | 0.025 | 0.0034 | 0.25 |
| 423: | 17 | 1 | 17 | 16 | 1 | 16 | 9677.1388 | 0.0033 | 0.025 | 0.0034 | 0.25 |
| 424: | 13 | 7 | 6 | 12 | 6 | 6 | 9701.2174 | 0.0050 | 0.025 | | |
| 425: | 12 | 10 | 3 | 11 | 9 | 2 | 9738.4504 | 0.0016 | 0.025 | | |
| 426: | 12 | 10 | 2 | 11 | 9 | 2 | 9738.6033 | 0.0143 | 0.025 | | |
| 427: | 12 | 10 | 3 | 11 | 9 | 3 | 9738.9604 | -0.0095 | 0.025 | | |
| 428: | 12 | 10 | 2 | 11 | 9 | 3 | 9739.1148 | 0.0046 | 0.025 | | |
| 429: | 13 | 8 | 6 | 12 | 7 | 5 | 9749.4108 | 0.0110 | 0.025 | | |
| 430: | 16 | 3 | 13 | 15 | 4 | 12 | 9749.7106 | 0.0105 | 0.025 | 0.0036 | 0.25 |
| 431: | 16 | 4 | 13 | 15 | 4 | 12 | 9749.7106 | 0.0080 | 0.025 | 0.0036 | 0.25 |
| 432: | 16 | 3 | 13 | 15 | 3 | 12 | 9749.7106 | -0.0008 | 0.025 | 0.0036 | 0.25 |
| 433: | 16 | 4 | 13 | 15 | 3 | 12 | 9749.7106 | -0.0033 | 0.025 | 0.0036 | 0.25 |
| 434: | 13 | 6 | 7 | 12 | 5 | 7 | 9773.3139 | 0.0130 | 0.025 | | |
| 435: | 17 | 1 | 16 | 16 | 1 | 15 | 9888.3936 | 0.0067 | 0.025 | 0.0067 | 0.25 |
| 436: | 17 | 1 | 16 | 16 | 2 | 15 | 9888.3936 | 0.0067 | 0.025 | 0.0067 | 0.25 |
| 437: | 17 | 2 | 16 | 16 | 1 | 15 | 9888.3936 | 0.0067 | 0.025 | 0.0067 | 0.25 |
| 438: | 17 | 2 | 16 | 16 | 2 | 15 | 9888.3936 | 0.0067 | 0.025 | 0.0067 | 0.25 |
| 439: | 13 | 7 | 7 | 12 | 6 | 7 | 9947.3340 | -0.0174 | 0.025 | | |
| 440: | 13 | 6 | 8 | 12 | 5 | 8 | 9951.2953 | -0.0365 | 0.025 | | |
| 441: | 13 | 8 | 6 | 12 | 7 | 6 | 10026.8533 | -0.0003 | 0.025 | | |
| 442: | 13 | 9 | 5 | 12 | 8 | 4 | 10148.7579 | 0.0077 | 0.025 | | |
| 443: | 13 | 8 | 5 | 12 | 7 | 6 | 10162.9031 | 0.0079 | 0.025 | | |
| 444: | 13 | 9 | 4 | 12 | 8 | 4 | 10164.1112 | 0.0127 | 0.025 | | |
| 445: | 14 | 8 | 7 | 13 | 7 | 6 | 10167.1263 | 0.0039 | 0.025 | | |
| 446: | 16 | 5 | 11 | 15 | 6 | 10 | 10180.3620 | 0.0026 | 0.025 | | |
| 447: | 18 | 0 | 18 | 17 | 0 | 17 | 10240.0671 | 0.0272 | 0.025 | 0.0272 | 0.25 |
| 448: | 18 | 0 | 18 | 17 | 1 | 17 | 10240.0671 | 0.0272 | 0.025 | 0.0272 | 0.25 |
| 449: | 18 | 1 | 18 | 17 | 0 | 17 | 10240.0671 | 0.0272 | 0.025 | 0.0272 | 0.25 |
| 450: | 18 | 1 | 18 | 17 | 1 | 17 | 10240.0671 | 0.0272 | 0.025 | 0.0272 | 0.25 |
| 451: | 17 | 3 | 14 | 16 | 4 | 13 | 10312.2723 | 0.0076 | 0.025 | 0.0061 | 0.25 |
| 452: | 17 | 4 | 14 | 16 | 4 | 13 | 10312.2723 | 0.0071 | 0.025 | 0.0061 | 0.25 |
| 453: | 17 | 3 | 14 | 16 | 3 | 13 | 10312.2723 | 0.0051 | 0.025 | 0.0061 | 0.25 |
| 454: | 17 | 4 | 14 | 16 | 3 | 13 | 10312.2723 | 0.0045 | 0.025 | 0.0061 | 0.25 |
| 455: | 13 | 10 | 4 | 12 | 9 | 3 | 10387.1070 | 0.0067 | 0.025 | | |
| 456: | 13 | 10 | 3 | 12 | 9 | 3 | 10388.0687 | -0.0019 | 0.025 | | |
| 457: | 13 | 10 | 4 | 12 | 9 | 4 | 10390.3632 | 0.0080 | 0.025 | | |
| 458: | 13 | 10 | 3 | 12 | 9 | 4 | 10391.3365 | 0.0110 | 0.025 | | |
| 459: | 18 | 1 | 17 | 17 | 1 | 16 | 10451.2674 | -0.0007 | 0.025 | -0.0097 | 0.20 |
| 460: | 18 | 1 | 17 | 17 | 2 | 16 | 10451.2674 | -0.0007 | 0.025 | -0.0097 | 0.20 |
| 461: | 18 | 2 | 17 | 17 | 1 | 16 | 10451.2674 | -0.0007 | 0.025 | -0.0097 | 0.20 |
| 462: | 18 | 2 | 17 | 17 | 2 | 16 | 10451.2674 | -0.0007 | 0.025 | -0.0097 | 0.20 |
| 463: | 16 | 7 | 10 | 15 | 6 | 9 | 10451.2674 | -0.0457 | 0.025 | -0.0097 | 0.20 |
| 464: | 14 | 8 | 6 | 13 | 7 | 6 | 10502.5017 | 0.0085 | 0.025 | | |
| 465: | 13 | 7 | 6 | 12 | 6 | 7 | 10508.5262 | 0.0063 | 0.025 | | |
| 466: | 18 | 2 | 16 | 17 | 2 | 15 | 10662.7676 | 0.0062 | 0.025 | 0.0063 | 0.25 |
| 467: | 18 | 2 | 16 | 17 | 3 | 15 | 10662.7676 | 0.0063 | 0.025 | 0.0063 | 0.25 |
| 468: | 18 | 3 | 16 | 17 | 2 | 15 | 10662.7676 | 0.0062 | 0.025 | 0.0063 | 0.25 |
| 469: | 18 | 3 | 16 | 17 | 3 | 15 | 10662.7676 | 0.0063 | 0.025 | 0.0063 | 0.25 |
| 470: | 12 | 6 | 7 | 11 | 3 | 8 | 10685.0735 | 0.0143 | 0.025 | | |
| 471: | 14 | 7 | 8 | 13 | 6 | 8 | 10698.6059 | -0.0167 | 0.025 | | |
| 472: | 14 | 5 | 9 | 13 | 4 | 9 | 10714.6869 | 0.0098 | 0.025 | | |
| 473: | 14 | 9 | 6 | 13 | 8 | 5 | 10716.8434 | 0.0047 | 0.025 | | |
| 474: | 14 | 6 | 9 | 13 | 5 | 9 | 10726.9744 | 0.0081 | 0.025 | | |
| 475: | 14 | 8 | 7 | 13 | 7 | 7 | 10728.3003 | 0.0095 | 0.025 | | |
| 476: | 17 | 6 | 12 | 16 | 5 | 11 | 10744.4335 | -0.0148 | 0.025 | | |
| 477: | 14 | 4 | 10 | 13 | 3 | 10 | 10761.9687 | 0.0170 | 0.025 | | |
| 478: | 14 | 5 | 10 | 13 | 4 | 10 | 10762.8799 | 0.0059 | 0.025 | | |
| 479: | 14 | 9 | 5 | 13 | 8 | 5 | 10773.9857 | 0.0005 | 0.025 | | |
| 480: | 13 | 12 | 2 | 12 | 11 | 1 | 10792.4053 | 0.0097 | 0.025 | 0.0077 | 0.25 |
| 481: | 13 | 12 | 1 | 12 | 11 | 1 | 10792.4053 | 0.0089 | 0.025 | 0.0077 | 0.25 |
| 482: | 13 | 12 | 2 | 12 | 11 | 2 | 10792.4053 | 0.0064 | 0.025 | 0.0077 | 0.25 |
| 483: | 13 | 12 | 1 | 12 | 11 | 2 | 10792.4053 | 0.0057 | 0.025 | 0.0077 | 0.25 |
| 484: | 19 | 0 | 19 | 18 | 0 | 18 | 10802.9286 | 0.0052 | 0.025 | 0.0052 | 0.25 |

| | | | | | | | | | | | | |
|---|---|---|---|---|---|---|---|---|---|---|---|---|
| 485: | 19 | 0 | 19 | 18 | 1 | 18 | 10802.9286 | 0.0052 | 0.025 | 0.0052 | 0.25 |
| 486: | 19 | 1 | 19 | 18 | 0 | 18 | 10802.9286 | 0.0052 | 0.025 | 0.0052 | 0.25 |
| 487: | 19 | 1 | 19 | 18 | 1 | 18 | 10802.9286 | 0.0052 | 0.025 | 0.0052 | 0.25 |
| 488: | 14 | 2 | 12 | 13 | 1 | 12 | 10811.2336 | 0.0065 | 0.025 | 0.0059 | 0.50 |
| 489: | 14 | 3 | 12 | 13 | 2 | 12 | 10811.2336 | 0.0053 | 0.025 | 0.0059 | 0.50 |
| 490: | 14 | 9 | 6 | 13 | 8 | 6 | 10852.8872 | 0.0070 | 0.025 | | |
| 491: | 18 | 3 | 15 | 17 | 4 | 14 | 10874.8879 | 0.0078 | 0.025 | 0.0075 | 0.25 |
| 492: | 18 | 4 | 15 | 17 | 4 | 14 | 10874.8879 | 0.0077 | 0.025 | 0.0075 | 0.25 |
| 493: | 18 | 3 | 15 | 17 | 3 | 14 | 10874.8879 | 0.0073 | 0.025 | 0.0075 | 0.25 |
| 494: | 18 | 4 | 15 | 17 | 3 | 14 | 10874.8879 | 0.0071 | 0.025 | 0.0075 | 0.25 |
| 495: | 14 | 9 | 5 | 13 | 8 | 6 | 10910.0253 | -0.0013 | 0.025 | | |
| 496: | 17 | 6 | 11 | 16 | 6 | 10 | 10975.1897 | -0.0145 | 0.025 | | |
| 497: | 13 | 13 | 0 | 12 | 12 | 0 | 10991.5603 | 0.0133 | 0.025 | 0.0134 | 0.25 |
| 498: | 13 | 13 | 0 | 12 | 12 | 1 | 10991.5603 | 0.0133 | 0.025 | 0.0134 | 0.25 |
| 499: | 13 | 13 | 1 | 12 | 12 | 0 | 10991.5603 | 0.0133 | 0.025 | 0.0134 | 0.25 |
| 500: | 13 | 13 | 1 | 12 | 12 | 1 | 10991.5603 | 0.0133 | 0.025 | 0.0134 | 0.25 |
| 501: | 19 | 1 | 18 | 18 | 1 | 17 | 11014.1400 | 0.0096 | 0.025 | 0.0097 | 0.25 |
| 502: | 19 | 1 | 18 | 18 | 2 | 17 | 11014.1400 | 0.0096 | 0.025 | 0.0097 | 0.25 |
| 503: | 19 | 2 | 18 | 18 | 1 | 17 | 11014.1400 | 0.0096 | 0.025 | 0.0097 | 0.25 |
| 504: | 19 | 2 | 18 | 18 | 2 | 17 | 11014.1400 | 0.0096 | 0.025 | 0.0097 | 0.25 |
| 505: | 14 | 10 | 4 | 13 | 9 | 5 | 11045.9652 | 0.0083 | 0.025 | | |
| 506: | 15 | 8 | 7 | 14 | 7 | 7 | 11182.9091 | 0.0067 | 0.025 | | |
| 507: | 15 | 9 | 7 | 14 | 8 | 6 | 11195.8291 | -0.0104 | 0.025 | | |
| 508: | 14 | 11 | 4 | 13 | 10 | 3 | 11242.8940 | 0.0108 | 0.025 | | |
| 509: | 14 | 11 | 3 | 13 | 10 | 3 | 11243.1759 | 0.0165 | 0.025 | | |
| 510: | 14 | 11 | 4 | 13 | 10 | 4 | 11243.8532 | -0.0004 | 0.025 | | |
| 511: | 14 | 11 | 3 | 13 | 10 | 4 | 11244.1332 | 0.0035 | 0.025 | | |
| 512: | 15 | 7 | 8 | 14 | 6 | 8 | 11272.6910 | 0.0022 | 0.025 | | |
| 513: | 20 | 0 | 20 | 19 | 0 | 19 | 11365.7912 | 0.0065 | 0.025 | 0.0066 | 0.25 |
| 514: | 20 | 0 | 20 | 19 | 1 | 19 | 11365.7912 | 0.0065 | 0.025 | 0.0066 | 0.25 |
| 515: | 20 | 1 | 20 | 19 | 0 | 19 | 11365.7912 | 0.0065 | 0.025 | 0.0066 | 0.25 |
| 516: | 20 | 1 | 20 | 19 | 1 | 19 | 11365.7912 | 0.0065 | 0.025 | 0.0066 | 0.25 |
| 517: | 14 | 12 | 3 | 13 | 11 | 2 | 11445.5516 | 0.0316 | 0.025 | 0.0089 | 0.25 |
| 518: | 14 | 12 | 2 | 13 | 11 | 2 | 11445.5516 | 0.0224 | 0.025 | 0.0089 | 0.25 |
| 519: | 14 | 12 | 3 | 13 | 11 | 3 | 11445.5516 | -0.0047 | 0.025 | 0.0089 | 0.25 |
| 520: | 14 | 12 | 2 | 13 | 11 | 3 | 11445.5516 | -0.0138 | 0.025 | 0.0089 | 0.25 |
| 521: | 15 | 8 | 8 | 14 | 7 | 8 | 11457.2021 | 0.0104 | 0.025 | | |
| 522: | 17 | 10 | 7 | 16 | 10 | 6 | 11471.5080 | -0.0002 | 0.025 | | |
| 523: | 15 | 6 | 10 | 14 | 5 | 10 | 11505.9246 | 0.0038 | 0.025 | | |
| 524: | 18 | 6 | 12 | 17 | 7 | 11 | 11522.0852 | 0.0187 | 0.025 | | |
| 525: | 20 | 1 | 19 | 19 | 1 | 18 | 11576.9737 | 0.0017 | 0.025 | 0.0017 | 0.25 |
| 526: | 20 | 1 | 19 | 19 | 2 | 18 | 11576.9737 | 0.0017 | 0.025 | 0.0017 | 0.25 |
| 527: | 20 | 2 | 19 | 19 | 1 | 18 | 11576.9737 | 0.0017 | 0.025 | 0.0017 | 0.25 |
| 528: | 20 | 2 | 19 | 19 | 2 | 18 | 11576.9737 | 0.0017 | 0.025 | 0.0017 | 0.25 |
| 529: | 15 | 10 | 6 | 14 | 9 | 5 | 11636.3440 | 0.0156 | 0.025 | | |
| 530: | 14 | 13 | 2 | 13 | 12 | 1 | 11645.4468 | 0.0132 | 0.025 | 0.0127 | 0.25 |
| 531: | 14 | 13 | 1 | 13 | 12 | 1 | 11645.4468 | 0.0130 | 0.025 | 0.0127 | 0.25 |
| 532: | 14 | 13 | 2 | 13 | 12 | 2 | 11645.4468 | 0.0124 | 0.025 | 0.0127 | 0.25 |
| 533: | 14 | 13 | 1 | 13 | 12 | 2 | 11645.4468 | 0.0122 | 0.025 | 0.0127 | 0.25 |
| 534: | 19 | 4 | 15 | 18 | 5 | 14 | 11650.5544 | 0.0038 | 0.025 | 0.0013 | 0.25 |
| 535: | 19 | 5 | 15 | 18 | 5 | 14 | 11650.5544 | 0.0029 | 0.025 | 0.0013 | 0.25 |
| 536: | 19 | 4 | 15 | 18 | 4 | 14 | 11650.5544 | -0.0002 | 0.025 | 0.0013 | 0.25 |
| 537: | 19 | 5 | 15 | 18 | 4 | 14 | 11650.5544 | -0.0012 | 0.025 | 0.0013 | 0.25 |
| 538: | 15 | 10 | 6 | 14 | 9 | 6 | 11693.4792 | 0.0043 | 0.025 | | |
| 539: | 15 | 9 | 6 | 14 | 8 | 7 | 11700.6269 | 0.0169 | 0.025 | | |
| 540: | 15 | 10 | 5 | 14 | 9 | 6 | 11714.9293 | 0.0143 | 0.025 | | |
| 541: | 18 | 7 | 11 | 17 | 8 | 10 | 11720.5122 | -0.0075 | 0.025 | | |
| 542: | 19 | 9 | 10 | 18 | 10 | 8 | 11832.5134 | -0.0368 | 0.025 | | |
| 543: | 14 | 14 | 0 | 13 | 13 | 0 | 11844.5314 | 0.0104 | 0.025 | 0.0104 | 0.25 |
| 544: | 14 | 14 | 0 | 13 | 13 | 1 | 11844.5314 | 0.0104 | 0.025 | 0.0104 | 0.25 |
| 545: | 14 | 14 | 1 | 13 | 13 | 0 | 11844.5314 | 0.0104 | 0.025 | 0.0104 | 0.25 |
| 546: | 14 | 14 | 1 | 13 | 13 | 1 | 11844.5314 | 0.0104 | 0.025 | 0.0104 | 0.25 |
| 547: | 15 | 11 | 5 | 14 | 10 | 4 | 11888.5891 | 0.0144 | 0.025 | | |
| 548: | 15 | 11 | 4 | 14 | 10 | 5 | 11895.2833 | 0.0126 | 0.025 | | |
| 549: | 21 | 0 | 21 | 20 | 0 | 20 | 11928.6304 | 0.0077 | 0.025 | 0.0078 | 0.25 |
| 550: | 21 | 0 | 21 | 20 | 1 | 20 | 11928.6304 | 0.0077 | 0.025 | 0.0078 | 0.25 |
| 551: | 21 | 1 | 21 | 20 | 0 | 20 | 11928.6304 | 0.0077 | 0.025 | 0.0078 | 0.25 |
| 552: | 21 | 1 | 21 | 20 | 1 | 20 | 11928.6304 | 0.0077 | 0.025 | 0.0078 | 0.25 |
| 553: | 21 | 1 | 20 | 20 | 1 | 19 | 12139.7949 | 0.0036 | 0.025 | 0.0036 | 0.25 |
| 554: | 21 | 1 | 20 | 20 | 2 | 19 | 12139.7949 | 0.0036 | 0.025 | 0.0036 | 0.25 |

| | | | | | | | | | | | | | |
|---|---|---|---|---|---|---|---|---|---|---|---|---|---|
| 555: | 21 | 2 | 20 | 20 | 1 | 19 | 12139.7949 | 0.0036 | 0.025 | 0.0036 | 0.25 |
| 556: | 21 | 2 | 20 | 20 | 2 | 19 | 12139.7949 | 0.0036 | 0.025 | 0.0036 | 0.25 |
| 557: | 20 | 4 | 16 | 19 | 5 | 15 | 12212.9167 | 0.0046 | 0.025 | 0.0040 | 0.25 |
| 558: | 20 | 5 | 16 | 19 | 5 | 15 | 12212.9167 | 0.0044 | 0.025 | 0.0040 | 0.25 |
| 559: | 20 | 4 | 16 | 19 | 4 | 15 | 12212.9167 | 0.0036 | 0.025 | 0.0040 | 0.25 |
| 560: | 20 | 5 | 16 | 19 | 4 | 15 | 12212.9167 | 0.0034 | 0.025 | 0.0040 | 0.25 |
| 561: | 15 | 13 | 3 | 14 | 12 | 2 | 12298.7285 | 0.0137 | 0.025 | 0.0081 | 0.25 |
| 562: | 15 | 13 | 2 | 14 | 12 | 2 | 12298.7285 | 0.0115 | 0.025 | 0.0081 | 0.25 |
| 563: | 15 | 13 | 3 | 14 | 12 | 3 | 12298.7285 | 0.0046 | 0.025 | 0.0081 | 0.25 |
| 564: | 15 | 13 | 2 | 14 | 12 | 3 | 12298.7285 | 0.0023 | 0.025 | 0.0081 | 0.25 |
| 565: | 22 | 0 | 22 | 21 | 0 | 21 | 12491.4369 | 0.0008 | 0.025 | 0.0008 | 0.25 |
| 566: | 22 | 0 | 22 | 21 | 1 | 21 | 12491.4369 | 0.0008 | 0.025 | 0.0008 | 0.25 |
| 567: | 22 | 1 | 22 | 21 | 0 | 21 | 12491.4369 | 0.0008 | 0.025 | 0.0008 | 0.25 |
| 568: | 22 | 1 | 22 | 21 | 1 | 21 | 12491.4369 | 0.0008 | 0.025 | 0.0008 | 0.25 |
| 569: | 15 | 14 | 2 | 14 | 13 | 1 | 12498.4233 | 0.0065 | 0.025 | 0.0065 | 0.25 |
| 570: | 15 | 14 | 1 | 14 | 13 | 1 | 12498.4233 | 0.0065 | 0.025 | 0.0065 | 0.25 |
| 571: | 15 | 14 | 1 | 14 | 13 | 2 | 12498.4233 | 0.0063 | 0.025 | 0.0065 | 0.25 |
| 572: | 15 | 14 | 2 | 14 | 13 | 2 | 12498.4233 | 0.0064 | 0.025 | 0.0065 | 0.25 |
| 573: | 15 | 15 | 0 | 14 | 14 | 0 | 12697.4643 | 0.0119 | 0.025 | 0.0120 | 0.25 |
| 574: | 15 | 15 | 0 | 14 | 14 | 1 | 12697.4643 | 0.0119 | 0.025 | 0.0120 | 0.25 |
| 575: | 15 | 15 | 1 | 14 | 14 | 0 | 12697.4643 | 0.0119 | 0.025 | 0.0120 | 0.25 |
| 576: | 15 | 15 | 1 | 14 | 14 | 1 | 12697.4643 | 0.0119 | 0.025 | 0.0120 | 0.25 |
| 577: | 22 | 1 | 21 | 21 | 1 | 20 | 12702.5822 | -0.0044 | 0.025 | -0.0044 | 0.25 |
| 578: | 22 | 1 | 21 | 21 | 2 | 20 | 12702.5822 | -0.0044 | 0.025 | -0.0044 | 0.25 |
| 579: | 22 | 2 | 21 | 21 | 1 | 20 | 12702.5822 | -0.0044 | 0.025 | -0.0044 | 0.25 |
| 580: | 22 | 2 | 21 | 21 | 2 | 20 | 12702.5822 | -0.0044 | 0.025 | -0.0044 | 0.25 |
| 581: | 16 | 12 | 5 | 15 | 11 | 4 | 12745.9574 | -0.0023 | 0.025 | | |
| 582: | 16 | 12 | 4 | 15 | 11 | 4 | 12746.4386 | 0.0028 | 0.025 | | |
| 583: | 16 | 12 | 5 | 15 | 11 | 5 | 12747.5739 | 0.0185 | 0.025 | | |
| 584: | 16 | 12 | 4 | 15 | 11 | 5 | 12748.0361 | 0.0046 | 0.025 | | |
| 585: | 21 | 5 | 16 | 20 | 6 | 15 | 12989.2027 | 0.0094 | 0.025 | 0.0056 | 0.25 |
| 586: | 21 | 6 | 16 | 20 | 6 | 15 | 12989.2027 | 0.0079 | 0.025 | 0.0056 | 0.25 |
| 587: | 21 | 5 | 16 | 20 | 5 | 15 | 12989.2027 | 0.0032 | 0.025 | 0.0056 | 0.25 |
| 588: | 21 | 6 | 16 | 20 | 5 | 15 | 12989.2027 | 0.0017 | 0.025 | 0.0056 | 0.25 |
| 589: | 23 | 0 | 23 | 22 | 0 | 22 | 13054.2249 | 0.0011 | 0.025 | 0.0011 | 0.25 |
| 590: | 23 | 0 | 23 | 22 | 1 | 22 | 13054.2249 | 0.0011 | 0.025 | 0.0011 | 0.25 |
| 591: | 23 | 1 | 23 | 22 | 0 | 22 | 13054.2249 | 0.0011 | 0.025 | 0.0011 | 0.25 |
| 592: | 23 | 1 | 23 | 22 | 1 | 22 | 13054.2249 | 0.0011 | 0.025 | 0.0011 | 0.25 |
| 593: | 15 | 8 | 8 | 14 | 5 | 9 | 13119.0981 | -0.0175 | 0.025 | | |
| 594: | 17 | 11 | 7 | 16 | 10 | 6 | 13120.5860 | 0.0147 | 0.025 | | |
| 595: | 22 | 3 | 19 | 21 | 3 | 18 | 13125.5209 | -0.0031 | 0.025 | -0.0032 | 0.25 |
| 596: | 22 | 3 | 19 | 21 | 4 | 18 | 13125.5209 | -0.0031 | 0.025 | -0.0032 | 0.25 |
| 597: | 22 | 4 | 19 | 21 | 3 | 18 | 13125.5209 | -0.0031 | 0.025 | -0.0032 | 0.25 |
| 598: | 22 | 4 | 19 | 21 | 4 | 18 | 13125.5209 | -0.0031 | 0.025 | -0.0032 | 0.25 |
| 599: | 16 | 14 | 3 | 15 | 13 | 2 | 13151.8052 | 0.0059 | 0.025 | 0.0046 | 0.25 |
| 600: | 16 | 14 | 2 | 15 | 13 | 2 | 13151.8052 | 0.0054 | 0.025 | 0.0046 | 0.25 |
| 601: | 16 | 14 | 3 | 15 | 13 | 3 | 13151.8052 | 0.0037 | 0.025 | 0.0046 | 0.25 |
| 602: | 16 | 14 | 2 | 15 | 13 | 3 | 13151.8052 | 0.0031 | 0.025 | 0.0046 | 0.25 |
| 603: | 23 | 1 | 22 | 22 | 1 | 21 | 13265.3551 | -0.0017 | 0.025 | -0.0018 | 0.25 |
| 604: | 23 | 1 | 22 | 22 | 2 | 21 | 13265.3551 | -0.0017 | 0.025 | -0.0018 | 0.25 |
| 605: | 23 | 2 | 22 | 22 | 1 | 21 | 13265.3551 | -0.0017 | 0.025 | -0.0018 | 0.25 |
| 606: | 23 | 2 | 22 | 22 | 2 | 21 | 13265.3551 | -0.0017 | 0.025 | -0.0018 | 0.25 |
| 607: | 16 | 15 | 1 | 15 | 14 | 1 | 13351.3542 | 0.0076 | 0.025 | 0.0076 | 0.25 |
| 608: | 16 | 15 | 1 | 15 | 14 | 2 | 13351.3542 | 0.0075 | 0.025 | 0.0076 | 0.25 |
| 609: | 16 | 15 | 2 | 15 | 14 | 1 | 13351.3542 | 0.0076 | 0.025 | 0.0076 | 0.25 |
| 610: | 16 | 15 | 2 | 15 | 14 | 2 | 13351.3542 | 0.0075 | 0.025 | 0.0076 | 0.25 |
| 611: | 17 | 12 | 5 | 16 | 11 | 5 | 13390.6853 | 0.0038 | 0.025 | | |
| 612: | 17 | 12 | 6 | 16 | 11 | 6 | 13395.6255 | -0.0596 | 0.025 | | |
| 613: | 17 | 12 | 5 | 16 | 11 | 6 | 13398.1039 | 0.0005 | 0.025 | | |
| 614: | 23 | 2 | 21 | 22 | 2 | 20 | 13476.6199 | -0.0011 | 0.025 | -0.0011 | 0.25 |
| 615: | 23 | 2 | 21 | 22 | 3 | 20 | 13476.6199 | -0.0011 | 0.025 | -0.0011 | 0.25 |
| 616: | 23 | 3 | 21 | 22 | 2 | 20 | 13476.6199 | -0.0011 | 0.025 | -0.0011 | 0.25 |
| 617: | 23 | 3 | 21 | 22 | 3 | 20 | 13476.6199 | -0.0011 | 0.025 | -0.0011 | 0.25 |
| 618: | 16 | 16 | 0 | 15 | 15 | 0 | 13550.3484 | 0.0104 | 0.025 | 0.0104 | 0.25 |
| 619: | 16 | 16 | 0 | 15 | 15 | 1 | 13550.3484 | 0.0104 | 0.025 | 0.0104 | 0.25 |
| 620: | 16 | 16 | 1 | 15 | 15 | 0 | 13550.3484 | 0.0104 | 0.025 | 0.0104 | 0.25 |
| 621: | 16 | 16 | 1 | 15 | 15 | 1 | 13550.3484 | 0.0104 | 0.025 | 0.0104 | 0.25 |
| 622: | 22 | 5 | 17 | 21 | 6 | 16 | 13551.2428 | 0.0012 | 0.025 | 0.0003 | 0.25 |
| 623: | 22 | 6 | 17 | 21 | 6 | 16 | 13551.2428 | 0.0009 | 0.025 | 0.0003 | 0.25 |
| 624: | 22 | 5 | 17 | 21 | 5 | 16 | 13551.2428 | -0.0002 | 0.025 | 0.0003 | 0.25 |

| | | | | | | | | | | | |
|---|---|---|---|---|---|---|---|---|---|---|---|
| 625: | 22 | 6 | 17 | 21 | 5 | 16 | 13551.2428 | -0.0005 | 0.025 | 0.0003 | 0.25 |
| 626: | 24 | 0 | 24 | 23 | 0 | 23 | 13616.9877 | 0.0031 | 0.025 | 0.0031 | 0.25 |
| 627: | 24 | 0 | 24 | 23 | 1 | 23 | 13616.9877 | 0.0031 | 0.025 | 0.0031 | 0.25 |
| 628: | 24 | 1 | 24 | 23 | 0 | 23 | 13616.9877 | 0.0031 | 0.025 | 0.0031 | 0.25 |
| 629: | 24 | 1 | 24 | 23 | 1 | 23 | 13616.9877 | 0.0031 | 0.025 | 0.0031 | 0.25 |
| 630: | 20 | 10 | 11 | 19 | 9 | 10 | 13633.9119 | -0.0076 | 0.025 | | |
| 631: | 23 | 3 | 20 | 22 | 3 | 19 | 13688.1752 | -0.0057 | 0.025 | -0.0057 | 0.25 |
| 632: | 23 | 3 | 20 | 22 | 4 | 19 | 13688.1752 | -0.0057 | 0.025 | -0.0057 | 0.25 |
| 633: | 23 | 4 | 20 | 22 | 3 | 19 | 13688.1752 | -0.0057 | 0.025 | -0.0057 | 0.25 |
| 634: | 23 | 4 | 20 | 22 | 4 | 19 | 13688.1752 | -0.0057 | 0.025 | -0.0057 | 0.25 |
| 635: | 24 | 1 | 23 | 23 | 1 | 22 | 13828.1009 | 0.0003 | 0.025 | 0.0004 | 0.25 |
| 636: | 24 | 1 | 23 | 23 | 2 | 22 | 13828.1009 | 0.0003 | 0.025 | 0.0004 | 0.25 |
| 637: | 24 | 2 | 23 | 23 | 1 | 22 | 13828.1009 | 0.0003 | 0.025 | 0.0004 | 0.25 |
| 638: | 24 | 2 | 23 | 23 | 2 | 22 | 13828.1009 | 0.0003 | 0.025 | 0.0004 | 0.25 |
| 639: | 23 | 4 | 19 | 22 | 4 | 18 | 13900.2979 | 0.0002 | 0.025 | 0.0002 | 0.25 |
| 640: | 23 | 4 | 19 | 22 | 5 | 18 | 13900.2979 | 0.0002 | 0.025 | 0.0002 | 0.25 |
| 641: | 23 | 5 | 19 | 22 | 4 | 18 | 13900.2979 | 0.0002 | 0.025 | 0.0002 | 0.25 |
| 642: | 23 | 5 | 19 | 22 | 5 | 18 | 13900.2979 | 0.0002 | 0.025 | 0.0002 | 0.25 |
| 643: | 17 | 15 | 3 | 16 | 14 | 2 | 14004.7962 | -0.0001 | 0.025 | -0.0005 | 0.25 |
| 644: | 17 | 15 | 2 | 16 | 14 | 2 | 14004.7962 | -0.0002 | 0.025 | -0.0005 | 0.25 |
| 645: | 17 | 15 | 3 | 16 | 14 | 3 | 14004.7962 | -0.0006 | 0.025 | -0.0005 | 0.25 |
| 646: | 17 | 15 | 2 | 16 | 14 | 3 | 14004.7962 | -0.0008 | 0.025 | -0.0005 | 0.25 |
| 647: | 24 | 2 | 22 | 23 | 2 | 21 | 14039.3241 | -0.0077 | 0.025 | -0.0077 | 0.25 |
| 648: | 24 | 2 | 22 | 23 | 3 | 21 | 14039.3241 | -0.0077 | 0.025 | -0.0077 | 0.25 |
| 649: | 24 | 3 | 22 | 23 | 2 | 21 | 14039.3241 | -0.0077 | 0.025 | -0.0077 | 0.25 |
| 650: | 24 | 3 | 22 | 23 | 3 | 21 | 14039.3241 | -0.0077 | 0.025 | -0.0077 | 0.25 |
| 651: | 23 | 5 | 18 | 22 | 6 | 17 | 14113.4001 | 0.0053 | 0.025 | 0.0052 | 0.25 |
| 652: | 23 | 6 | 18 | 22 | 6 | 17 | 14113.4001 | 0.0053 | 0.025 | 0.0052 | 0.25 |
| 653: | 23 | 5 | 18 | 22 | 5 | 17 | 14113.4001 | 0.0050 | 0.025 | 0.0052 | 0.25 |
| 654: | 23 | 6 | 18 | 22 | 5 | 17 | 14113.4001 | 0.0049 | 0.025 | 0.0052 | 0.25 |
| 655: | 17 | 16 | 1 | 16 | 15 | 1 | 14204.2213 | -0.0014 | 0.025 | -0.0015 | 0.25 |
| 656: | 17 | 16 | 1 | 16 | 15 | 2 | 14204.2213 | -0.0014 | 0.025 | -0.0015 | 0.25 |
| 657: | 17 | 16 | 2 | 16 | 15 | 1 | 14204.2213 | -0.0014 | 0.025 | -0.0015 | 0.25 |
| 658: | 17 | 16 | 2 | 16 | 15 | 2 | 14204.2213 | -0.0014 | 0.025 | -0.0015 | 0.25 |
| 659: | 25 | 1 | 24 | 24 | 1 | 23 | 14390.8153 | -0.0009 | 0.025 | -0.0009 | 0.25 |
| 660: | 25 | 1 | 24 | 24 | 2 | 23 | 14390.8153 | -0.0009 | 0.025 | -0.0009 | 0.25 |
| 661: | 25 | 2 | 24 | 24 | 1 | 23 | 14390.8153 | -0.0009 | 0.025 | -0.0009 | 0.25 |
| 662: | 25 | 2 | 24 | 24 | 2 | 23 | 14390.8153 | -0.0009 | 0.025 | -0.0009 | 0.25 |
| 663: | 17 | 17 | 0 | 16 | 16 | 0 | 14403.1790 | 0.0038 | 0.025 | 0.0039 | 0.25 |
| 664: | 17 | 17 | 0 | 16 | 16 | 1 | 14403.1790 | 0.0038 | 0.025 | 0.0039 | 0.25 |
| 665: | 17 | 17 | 1 | 16 | 16 | 0 | 14403.1790 | 0.0038 | 0.025 | 0.0039 | 0.25 |
| 666: | 17 | 17 | 1 | 16 | 16 | 1 | 14403.1790 | 0.0038 | 0.025 | 0.0039 | 0.25 |
| 667: | 17 | 8 | 9 | 16 | 7 | 10 | 14434.6339 | -0.0226 | 0.025 | | |
| 668: | 24 | 4 | 20 | 23 | 4 | 19 | 14462.7970 | -0.0043 | 0.025 | -0.0044 | 0.25 |
| 669: | 24 | 4 | 20 | 23 | 5 | 19 | 14462.7970 | -0.0043 | 0.025 | -0.0044 | 0.25 |
| 670: | 24 | 5 | 20 | 23 | 4 | 19 | 14462.7970 | -0.0043 | 0.025 | -0.0044 | 0.25 |
| 671: | 24 | 5 | 20 | 23 | 5 | 19 | 14462.7970 | -0.0043 | 0.025 | -0.0044 | 0.25 |
| 672: | 19 | 10 | 10 | 18 | 9 | 10 | 14472.3032 | 0.0010 | 0.025 | | |
| 673: | 25 | 2 | 23 | 24 | 2 | 22 | 14602.0146 | -0.0024 | 0.025 | -0.0025 | 0.25 |
| 674: | 25 | 2 | 23 | 24 | 3 | 22 | 14602.0146 | -0.0024 | 0.025 | -0.0025 | 0.25 |
| 675: | 25 | 3 | 23 | 24 | 2 | 22 | 14602.0146 | -0.0024 | 0.025 | -0.0025 | 0.25 |
| 676: | 25 | 3 | 23 | 24 | 3 | 22 | 14602.0146 | -0.0024 | 0.025 | -0.0025 | 0.25 |
| 677: | 18 | 16 | 3 | 17 | 15 | 2 | 14857.6978 | -0.0186 | 0.025 | -0.0187 | 0.25 |
| 678: | 18 | 16 | 2 | 17 | 15 | 2 | 14857.6978 | -0.0186 | 0.025 | -0.0187 | 0.25 |
| 679: | 18 | 16 | 2 | 17 | 15 | 3 | 14857.6978 | -0.0187 | 0.025 | -0.0187 | 0.25 |
| 680: | 18 | 16 | 3 | 17 | 15 | 3 | 14857.6978 | -0.0187 | 0.025 | -0.0187 | 0.25 |
| 681: | 26 | 1 | 25 | 25 | 1 | 24 | 14953.4934 | -0.0093 | 0.025 | -0.0093 | 0.25 |
| 682: | 26 | 1 | 25 | 25 | 2 | 24 | 14953.4934 | -0.0093 | 0.025 | -0.0093 | 0.25 |
| 683: | 26 | 2 | 25 | 25 | 1 | 24 | 14953.4934 | -0.0093 | 0.025 | -0.0093 | 0.25 |
| 684: | 26 | 2 | 25 | 25 | 2 | 24 | 14953.4934 | -0.0093 | 0.025 | -0.0093 | 0.25 |
| 685: | 18 | 17 | 1 | 17 | 16 | 1 | 15057.0467 | 0.0025 | 0.025 | 0.0026 | 0.25 |
| 686: | 18 | 17 | 1 | 17 | 16 | 2 | 15057.0467 | 0.0025 | 0.025 | 0.0026 | 0.25 |
| 687: | 18 | 17 | 2 | 17 | 16 | 1 | 15057.0467 | 0.0025 | 0.025 | 0.0026 | 0.25 |
| 688: | 18 | 17 | 2 | 17 | 16 | 2 | 15057.0467 | 0.0025 | 0.025 | 0.0026 | 0.25 |
| 689: | 20 | 9 | 11 | 19 | 8 | 11 | 15124.9872 | -0.0338 | 0.025 | | |
| 690: | 19 | 16 | 4 | 18 | 15 | 3 | 15510.5233 | -0.0143 | 0.025 | -0.0151 | 0.25 |
| 691: | 19 | 16 | 3 | 18 | 15 | 3 | 15510.5233 | -0.0146 | 0.025 | -0.0151 | 0.25 |
| 692: | 19 | 16 | 4 | 18 | 15 | 4 | 15510.5233 | -0.0156 | 0.025 | -0.0151 | 0.25 |
| 693: | 19 | 16 | 3 | 18 | 15 | 4 | 15510.5233 | -0.0159 | 0.025 | -0.0151 | 0.25 |
| 694: | 27 | 1 | 26 | 26 | 1 | 25 | 15516.1526 | -0.0061 | 0.025 | -0.0062 | 0.25 |

```
 695: 27  1 26   26  2 25                         15516.1526  -0.0061  0.025  -0.0062 0.25
 696: 27  2 26   26  1 25                         15516.1526  -0.0061  0.025  -0.0062 0.25
 697: 27  2 26   26  2 25                         15516.1526  -0.0061  0.025  -0.0062 0.25
 698: 26  4 22   25  4 21                         15587.7997  -0.0071  0.025  -0.0071 0.25
 699: 26  4 22   25  5 21                         15587.7997  -0.0071  0.025  -0.0071 0.25
 700: 26  5 22   25  4 21                         15587.7997  -0.0071  0.025  -0.0071 0.25
 701: 26  5 22   25  5 21                         15587.7997  -0.0071  0.025  -0.0071 0.25
 702: 19 17  2   18 16  2                         15710.5553  -0.0087  0.025  -0.0087 0.25
 703: 19 17  3   18 16  2                         15710.5553  -0.0087  0.025  -0.0087 0.25
 704: 19 17  2   18 16  3                         15710.5553  -0.0087  0.025  -0.0087 0.25
 705: 19 17  3   18 16  3                         15710.5553  -0.0087  0.025  -0.0087 0.25
 706: 27  2 25   26  2 24                         15727.3018  -0.0018  0.025  -0.0019 0.25
 707: 27  2 25   26  3 24                         15727.3018  -0.0018  0.025  -0.0019 0.25
 708: 27  3 25   26  2 24                         15727.3018  -0.0018  0.025  -0.0019 0.25
 709: 27  3 25   26  3 24                         15727.3018  -0.0018  0.025  -0.0019 0.25
 710: 19 18  1   18 17  1                         15909.7990  -0.0097  0.025  -0.0097 0.25
 711: 19 18  1   18 17  2                         15909.7990  -0.0097  0.025  -0.0097 0.25
 712: 19 18  2   18 17  1                         15909.7990  -0.0097  0.025  -0.0097 0.25
 713: 19 18  2   18 17  2                         15909.7990  -0.0097  0.025  -0.0097 0.25
 714: 20 16  5   19 15  4                         16162.3329  -0.0042  0.025  -0.0107 0.25
 715: 20 16  4   19 15  4                         16162.3329  -0.0069  0.025  -0.0107 0.25
 716: 20 16  5   19 15  5                         16162.3329  -0.0144  0.025  -0.0107 0.25
 717: 20 16  4   19 15  5                         16162.3329  -0.0171  0.025  -0.0107 0.25
 718: 20 19  1   19 18  1                         16762.5087  -0.0058  0.025  -0.0058 0.25
 719: 20 19  1   19 18  2                         16762.5087  -0.0058  0.025  -0.0058 0.25
 720: 20 19  2   19 18  1                         16762.5087  -0.0058  0.025  -0.0058 0.25
 721: 20 19  2   19 18  2                         16762.5087  -0.0058  0.025  -0.0058 0.25
 722: 20 20  0   19 19  0                         16961.3623  -0.0044  0.025  -0.0044 0.25
 723: 20 20  0   19 19  1                         16961.3623  -0.0044  0.025  -0.0044 0.25
 724: 20 20  1   19 19  0                         16961.3623  -0.0044  0.025  -0.0044 0.25
 725: 20 20  1   19 19  1                         16961.3623  -0.0044  0.025  -0.0044 0.25
--------------------------------------------------------------------------------

PARAMETERS IN FIT:

        10000          A  /MHz        426.6195(2)                         1
        20000          B  /MHz        358.4285(1)                         2
        30000          C  /MHz        281.5441(1)                         3
          200         DJ  /kHz          0.0961(3)                         4
         1100        DJK  /kHz         -0.040(2)                          5
         2000         DK  /kHz          0.070(2)                          6
        40100         d1  /kHz         -0.0269(1)                         7
        50000         d2  /kHz         -0.0030(1)                         8

 MICROWAVE AVG =       -0.000534 MHz, IR AVG =        0.00000
 MICROWAVE RMS =        0.008096 MHz, IR RMS =        0.00000
 END OF ITERATION  1 OLD, NEW RMS ERROR=        0.32383        0.32383

  distinct frequency lines in fit:   474
       distinct parameters of fit:     8

 for standard errors previous errors are multiplied by:   0.326598

 PARAMETERS IN FIT WITH STANDARD ERRORS ON THOSE THAT ARE FITTED:

        10000          A  /MHz        426.61950(7)                        1
        20000          B  /MHz        358.42855(5)                        2
        30000          C  /MHz        281.54415(5)                        3
          200         DJ  /kHz          0.0961(1)                         4
         1100        DJK  /kHz         -0.0405(7)                         5
         2000         DK  /kHz          0.0706(6)                         6
        40100         d1  /kHz         -0.02690(5)                        7
        50000         d2  /kHz         -0.00306(3)                        8

 CORRELATION COEFFICIENTS, C.ij:

           A        B        C       -DJ      -DJK     -DK      d1       d2

A           1.0000
B          -0.0988   1.0000
```

```
C          -0.0971   0.1216   1.0000
-DJ         0.3808  -0.7049  -0.3739   1.0000
-DJK       -0.6606   0.2761   0.0909  -0.7229   1.0000
-DK         0.5004  -0.1543  -0.0116   0.5882  -0.9538   1.0000
d1          0.1751  -0.7880   0.1969   0.7193  -0.4189   0.3062   1.0000
d2         -0.2420  -0.1389   0.3216  -0.3711   0.5130  -0.5146   0.1057   1.0000

 Mean value of |C.ij|, i.ne.j =   0.3767
 Mean value of  C.ij,  i.ne.j =  -0.0698


 Worst fitting lines (obs-calc/error):

   612:   -2.4        542:   -1.5        440:   -1.5        689:   -1.4
   447:    1.1        365:    1.1        320:    1.0        401:    0.9
   667:   -0.9        160:   -0.9        254:   -0.8        343:    0.8
   524:    0.7        677:   -0.7        583:    0.7        415:    0.7
   593:   -0.7        352:    0.7        439:   -0.7        477:    0.7
   539:    0.7        471:   -0.7        509:    0.7        318:   -0.6
   121:   -0.6         13:   -0.6        529:    0.6        690:   -0.6
   476:   -0.6         59:   -0.6        594:    0.6        496:   -0.6
   547:    0.6        470:    0.6        540:    0.6        426:    0.6
   497:    0.5        402:    0.5        400:    0.5        434:    0.5
   530:    0.5        444:    0.5        548:    0.5         37:    0.5
    61:    0.5        328:    0.5        573:    0.5        170:   -0.5
   429:    0.4        458:    0.4
```

/ SPFIT output reformatted with PIFORM

Hexamer - 6 I

--------------------------------------------------------------------------------====
=====

| | | | | | | | obs | o-c | error | blends o-c | wt | Notes |
|---|---|---|---|---|---|---|---|---|---|---|---|---|
| 1: | 5 | 2 | 3 | 4 | 2 | 2 | 2030.5646 | 0.0027 | 0.025 | | | |
| 2: | 5 | 1 | 4 | 4 | 1 | 3 | 2032.4118 | 0.0032 | 0.025 | | | |
| 3: | 4 | 2 | 2 | 3 | 0 | 3 | 2277.4760 | -0.0084 | 0.025 | | | |
| 4: | 6 | 1 | 6 | 5 | 1 | 5 | 2281.7088 | -0.0016 | 0.025 | | | |
| 5: | 6 | 0 | 6 | 5 | 0 | 5 | 2298.4214 | -0.0001 | 0.025 | | | |
| 6: | 6 | 2 | 5 | 5 | 2 | 4 | 2365.9806 | 0.0011 | 0.025 | | | |
| 7: | 6 | 5 | 2 | 5 | 5 | 1 | 2388.7696 | 0.0068 | 0.025 | 0.0020 | 0.50 | |
| 8: | 6 | 5 | 1 | 5 | 5 | 0 | 2388.7696 | -0.0028 | 0.025 | 0.0020 | 0.50 | |
| 9: | 6 | 4 | 3 | 5 | 4 | 2 | 2391.4746 | 0.0056 | 0.025 | | | |
| 10: | 6 | 4 | 2 | 5 | 4 | 1 | 2392.0602 | 0.0021 | 0.025 | | | |
| 11: | 6 | 3 | 4 | 5 | 3 | 3 | 2392.2590 | 0.0019 | 0.025 | | | |
| 12: | 6 | 3 | 3 | 5 | 3 | 2 | 2405.5904 | 0.0015 | 0.025 | | | |
| 13: | 6 | 1 | 5 | 5 | 1 | 4 | 2426.1214 | 0.0042 | 0.025 | | | |
| 14: | 6 | 2 | 4 | 5 | 2 | 3 | 2447.5079 | 0.0037 | 0.025 | | | |
| 15: | 7 | 0 | 7 | 6 | 1 | 6 | 2641.8145 | 0.0028 | 0.025 | | | |
| 16: | 7 | 1 | 7 | 6 | 1 | 6 | 2655.5649 | -0.0025 | 0.025 | | | |
| 17: | 7 | 0 | 7 | 6 | 0 | 6 | 2666.2267 | -0.0017 | 0.025 | | | |
| 18: | 7 | 1 | 7 | 6 | 0 | 6 | 2679.9725 | -0.0116 | 0.025 | | | |
| 19: | 7 | 2 | 6 | 6 | 2 | 5 | 2752.5044 | -0.0014 | 0.025 | | | |
| 20: | 7 | 6 | 2 | 6 | 6 | 1 | 2786.5041 | 0.0041 | 0.025 | 0.0038 | 0.50 | |
| 21: | 7 | 6 | 1 | 6 | 6 | 0 | 2786.5041 | 0.0034 | 0.025 | 0.0038 | 0.50 | |
| 22: | 7 | 5 | 3 | 6 | 5 | 2 | 2788.9848 | 0.0304 | 0.025 | 0.0039 | 0.50 | |
| 23: | 7 | 5 | 2 | 6 | 5 | 1 | 2788.9848 | -0.0227 | 0.025 | 0.0039 | 0.50 | |
| 24: | 7 | 3 | 5 | 6 | 3 | 4 | 2791.1462 | 0.0012 | 0.025 | | | |
| 25: | 7 | 4 | 4 | 6 | 4 | 3 | 2792.8409 | 0.0010 | 0.025 | | | |
| 26: | 7 | 4 | 3 | 6 | 4 | 2 | 2794.7657 | 0.0014 | 0.025 | | | |
| 27: | 7 | 1 | 6 | 6 | 1 | 5 | 2811.6678 | 0.0038 | 0.025 | | | |
| 28: | 7 | 3 | 4 | 6 | 3 | 3 | 2819.0355 | 0.0006 | 0.025 | | | |
| 29: | 7 | 2 | 5 | 6 | 2 | 4 | 2861.6388 | 0.0034 | 0.025 | | | |
| 30: | 14 | 4 | 10 | 13 | 6 | 7 | 2885.6978 | 0.0302 | 0.025 | | | |
| 31: | 8 | 0 | 8 | 7 | 1 | 7 | 3020.6902 | -0.0036 | 0.025 | | | |
| 32: | 8 | 1 | 8 | 7 | 1 | 7 | 3028.1500 | -0.0029 | 0.025 | | | |
| 33: | 8 | 0 | 8 | 7 | 0 | 7 | 3034.4471 | -0.0023 | 0.025 | | | |
| 34: | 8 | 1 | 8 | 7 | 0 | 7 | 3041.9055 | -0.0031 | 0.025 | | | |
| 35: | 8 | 2 | 7 | 7 | 2 | 6 | 3135.9590 | 0.0002 | 0.025 | | | |
| 36: | 8 | 7 | 2 | 7 | 7 | 1 | 3184.2687 | 0.0030 | 0.025 | 0.0030 | 0.50 | |
| 37: | 8 | 7 | 1 | 7 | 7 | 0 | 3184.2687 | 0.0030 | 0.025 | 0.0030 | 0.50 | |
| 38: | 8 | 6 | 3 | 7 | 6 | 2 | 3186.4525 | 0.0044 | 0.025 | | | |
| 39: | 8 | 3 | 6 | 7 | 3 | 5 | 3188.4440 | -0.0004 | 0.025 | | | |
| 40: | 8 | 1 | 7 | 7 | 1 | 6 | 3188.5453 | 0.0041 | 0.025 | | | |
| 41: | 8 | 5 | 4 | 7 | 5 | 3 | 3190.0761 | 0.0030 | 0.025 | | | |
| 42: | 8 | 5 | 3 | 7 | 5 | 2 | 3190.2837 | 0.0008 | 0.025 | | | |
| 43: | 8 | 4 | 5 | 7 | 4 | 4 | 3194.9702 | 0.0025 | 0.025 | | | |
| 44: | 8 | 4 | 4 | 7 | 4 | 3 | 3200.0943 | 0.0010 | 0.025 | | | |
| 45: | 8 | 3 | 5 | 7 | 3 | 4 | 3238.1793 | 0.0019 | 0.025 | | | |
| 46: | 8 | 2 | 6 | 7 | 2 | 5 | 3270.4119 | 0.0054 | 0.025 | | | |
| 47: | 9 | 1 | 9 | 8 | 1 | 8 | 3399.8595 | -0.0028 | 0.025 | | | |
| 48: | 9 | 0 | 9 | 8 | 0 | 8 | 3403.3868 | -0.0027 | 0.025 | | | |
| 49: | 9 | 1 | 9 | 8 | 0 | 8 | 3407.3241 | 0.0025 | 0.025 | | | |
| 50: | 9 | 2 | 8 | 8 | 2 | 7 | 3516.4068 | 0.0003 | 0.025 | | | |
| 51: | 9 | 1 | 8 | 8 | 1 | 7 | 3558.3686 | 0.0037 | 0.025 | | | |
| 52: | 9 | 8 | 1 | 8 | 8 | 0 | 3582.0503 | 0.0030 | 0.025 | 0.0031 | 0.50 | |
| 53: | 9 | 8 | 2 | 8 | 8 | 1 | 3582.0503 | 0.0030 | 0.025 | 0.0031 | 0.50 | |
| 54: | 9 | 3 | 7 | 8 | 3 | 6 | 3583.4084 | 0.0020 | 0.025 | | | |
| 55: | 9 | 7 | 3 | 8 | 7 | 2 | 3584.0167 | -0.0228 | 0.025 | -0.0230 | 0.50 | |
| 56: | 9 | 7 | 2 | 8 | 7 | 1 | 3584.0167 | -0.0231 | 0.025 | -0.0230 | 0.50 | |
| 57: | 9 | 6 | 4 | 8 | 6 | 3 | 3587.1630 | 0.0101 | 0.025 | 0.0005 | 0.50 | |
| 58: | 9 | 6 | 3 | 8 | 6 | 2 | 3587.1630 | -0.0091 | 0.025 | 0.0005 | 0.50 | |
| 59: | 9 | 5 | 5 | 8 | 5 | 4 | 3592.1964 | 0.0023 | 0.025 | | | |
| 60: | 9 | 5 | 4 | 8 | 5 | 3 | 3592.8642 | 0.0011 | 0.025 | | | |
| 61: | 9 | 4 | 6 | 8 | 4 | 5 | 3597.4514 | 0.0021 | 0.025 | | | |

| | | | | | | | | | | | | |
|---|---|---|---|---|---|---|---|---|---|---|---|---|
| 62: | 9 | 4 | 5 | 8 | 4 | 4 | 3609.1635 | 0.0013 | 0.025 | | |
| 63: | 9 | 3 | 6 | 8 | 3 | 5 | 3660.8597 | 0.0028 | 0.025 | | |
| 64: | 9 | 2 | 7 | 8 | 2 | 6 | 3672.0575 | 0.0063 | 0.025 | | |
| 65: | 10 | 0 | 10 | 9 | 1 | 9 | 3768.9715 | -0.0119 | 0.025 | | |
| 66: | 10 | 1 | 10 | 9 | 1 | 9 | 3771.0086 | -0.0031 | 0.025 | | |
| 67: | 10 | 0 | 10 | 9 | 0 | 9 | 3772.9126 | -0.0028 | 0.025 | | |
| 68: | 10 | 1 | 10 | 9 | 0 | 9 | 3774.9364 | -0.0072 | 0.025 | | |
| 69: | 10 | 2 | 9 | 9 | 2 | 8 | 3894.1285 | -0.0010 | 0.025 | | |
| 70: | 10 | 1 | 9 | 9 | 1 | 8 | 3924.3632 | 0.0010 | 0.025 | | |
| 71: | 10 | 3 | 8 | 9 | 3 | 7 | 3975.4336 | 0.0014 | 0.025 | | |
| 72: | 10 | 9 | 1 | 9 | 9 | 0 | 3979.8365 | 0.0003 | 0.025 | 0.0004 | 0.50 |
| 73: | 10 | 9 | 2 | 9 | 9 | 1 | 3979.8365 | 0.0003 | 0.025 | 0.0004 | 0.50 |
| 74: | 10 | 8 | 2 | 9 | 8 | 1 | 3981.6975 | 0.0029 | 0.025 | 0.0029 | 0.50 |
| 75: | 10 | 8 | 3 | 9 | 8 | 2 | 3981.6975 | 0.0029 | 0.025 | 0.0029 | 0.50 |
| 76: | 10 | 7 | 4 | 9 | 7 | 3 | 3984.4370 | 0.0027 | 0.025 | 0.0019 | 0.50 |
| 77: | 10 | 7 | 3 | 9 | 7 | 2 | 3984.4370 | 0.0011 | 0.025 | 0.0019 | 0.50 |
| 78: | 10 | 6 | 5 | 9 | 6 | 4 | 3988.7118 | 0.0013 | 0.025 | | |
| 79: | 10 | 6 | 4 | 9 | 6 | 3 | 3988.7829 | 0.0012 | 0.025 | | |
| 80: | 10 | 5 | 6 | 9 | 5 | 5 | 3995.3204 | 0.0017 | 0.025 | | |
| 81: | 10 | 5 | 5 | 9 | 5 | 4 | 3997.1432 | 0.0012 | 0.025 | | |
| 82: | 10 | 4 | 7 | 9 | 4 | 6 | 3999.6708 | 0.0015 | 0.025 | | |
| 83: | 10 | 4 | 6 | 9 | 4 | 5 | 4023.2511 | 0.0004 | 0.025 | | |
| 84: | 10 | 2 | 8 | 9 | 2 | 7 | 4065.0693 | 0.0068 | 0.025 | | |
| 85: | 10 | 3 | 7 | 9 | 3 | 6 | 4083.1733 | 0.0026 | 0.025 | | |
| 86: | 11 | 0 | 11 | 10 | 1 | 10 | 4140.8002 | 0.0040 | 0.025 | | |
| 87: | 11 | 1 | 11 | 10 | 1 | 10 | 4141.8206 | -0.0041 | 0.025 | | |
| 88: | 11 | 0 | 11 | 10 | 0 | 10 | 4142.8205 | -0.0039 | 0.025 | | |
| 89: | 11 | 2 | 10 | 10 | 2 | 9 | 4269.5670 | -0.0019 | 0.025 | | |
| 90: | 11 | 1 | 10 | 10 | 1 | 9 | 4289.5694 | 0.0010 | 0.025 | | |
| 91: | 11 | 3 | 9 | 10 | 3 | 8 | 4364.1317 | -0.0003 | 0.025 | | |
| 92: | 11 | 10 | 1 | 10 | 10 | 0 | 4377.6327 | 0.0051 | 0.025 | 0.0051 | 0.50 |
| 93: | 11 | 10 | 2 | 10 | 10 | 1 | 4377.6327 | 0.0051 | 0.025 | 0.0051 | 0.50 |
| 94: | 11 | 9 | 2 | 10 | 9 | 1 | 4379.3883 | 0.0012 | 0.025 | 0.0013 | 0.50 |
| 95: | 11 | 9 | 3 | 10 | 9 | 2 | 4379.3883 | 0.0012 | 0.025 | 0.0013 | 0.50 |
| 96: | 11 | 8 | 4 | 10 | 8 | 3 | 4381.8665 | 0.0013 | 0.025 | 0.0013 | 0.50 |
| 97: | 11 | 8 | 3 | 10 | 8 | 2 | 4381.8665 | 0.0012 | 0.025 | 0.0013 | 0.50 |
| 98: | 11 | 7 | 5 | 10 | 7 | 4 | 4385.5280 | 0.0032 | 0.025 | | |
| 99: | 11 | 7 | 4 | 10 | 7 | 3 | 4385.5282 | -0.0031 | 0.025 | | |
| 100: | 11 | 6 | 6 | 10 | 6 | 5 | 4391.2101 | 0.0021 | 0.025 | | |
| 101: | 11 | 6 | 5 | 10 | 6 | 4 | 4391.4313 | -0.0003 | 0.025 | | |
| 102: | 11 | 5 | 7 | 10 | 5 | 6 | 4399.3309 | 0.0017 | 0.025 | | |
| 103: | 11 | 4 | 8 | 10 | 4 | 7 | 4400.8603 | 0.0012 | 0.025 | | |
| 104: | 11 | 5 | 6 | 10 | 5 | 5 | 4403.7191 | 0.0006 | 0.025 | | |
| 105: | 11 | 4 | 7 | 10 | 4 | 6 | 4443.2276 | 0.0000 | 0.025 | | |
| 106: | 11 | 2 | 9 | 10 | 2 | 8 | 4448.3898 | 0.0076 | 0.025 | | |
| 107: | 11 | 3 | 8 | 10 | 3 | 7 | 4501.2026 | 0.0050 | 0.025 | | |
| 108: | 12 | 0 | 12 | 11 | 1 | 11 | 4511.9239 | -0.0065 | 0.025 | | |
| 109: | 12 | 1 | 12 | 11 | 1 | 11 | 4512.4413 | -0.0036 | 0.025 | | |
| 110: | 12 | 0 | 12 | 11 | 0 | 11 | 4512.9545 | -0.0045 | 0.025 | | |
| 111: | 12 | 1 | 12 | 11 | 0 | 11 | 4513.4976 | 0.0241 | 0.025 | | |
| 112: | 12 | 2 | 11 | 11 | 2 | 10 | 4643.2351 | -0.0022 | 0.025 | | |
| 113: | 12 | 1 | 11 | 11 | 1 | 10 | 4655.6256 | -0.0014 | 0.025 | | |
| 114: | 12 | 3 | 10 | 11 | 3 | 9 | 4749.3569 | 0.0000 | 0.025 | | |
| 115: | 12 | 11 | 1 | 11 | 11 | 0 | 4775.4199 | 0.0017 | 0.025 | 0.0017 | 0.50 |
| 116: | 12 | 11 | 2 | 11 | 11 | 1 | 4775.4199 | 0.0017 | 0.025 | 0.0017 | 0.50 |
| 117: | 12 | 10 | 2 | 11 | 10 | 1 | 4777.1033 | 0.0014 | 0.025 | 0.0014 | 0.50 |
| 118: | 12 | 10 | 3 | 11 | 10 | 2 | 4777.1033 | 0.0014 | 0.025 | 0.0014 | 0.50 |
| 119: | 12 | 9 | 3 | 11 | 9 | 2 | 4779.3913 | 0.0020 | 0.025 | 0.0021 | 0.50 |
| 120: | 12 | 9 | 4 | 11 | 9 | 3 | 4779.3913 | 0.0020 | 0.025 | 0.0021 | 0.50 |
| 121: | 12 | 8 | 5 | 11 | 8 | 4 | 4782.6166 | 0.0009 | 0.025 | 0.0007 | 0.50 |
| 122: | 12 | 8 | 4 | 11 | 8 | 3 | 4782.6166 | 0.0004 | 0.025 | 0.0007 | 0.50 |
| 123: | 12 | 7 | 6 | 11 | 7 | 5 | 4787.3997 | 0.0114 | 0.025 | -0.0003 | 0.50 |
| 124: | 12 | 7 | 5 | 11 | 7 | 4 | 4787.3997 | -0.0120 | 0.025 | -0.0003 | 0.50 |
| 125: | 12 | 6 | 6 | 11 | 6 | 5 | 4795.3281 | -0.0007 | 0.025 | | |
| 126: | 12 | 4 | 9 | 11 | 4 | 8 | 4800.1989 | 0.0000 | 0.025 | | |
| 127: | 12 | 5 | 8 | 11 | 5 | 7 | 4803.9438 | 0.0009 | 0.025 | | |
| 128: | 9 | 3 | 6 | 8 | 1 | 7 | 4812.2167 | 0.0164 | 0.025 | | |
| 129: | 12 | 5 | 7 | 11 | 5 | 6 | 4813.4670 | -0.0002 | 0.025 | | |
| 130: | 12 | 2 | 10 | 11 | 2 | 9 | 4822.2707 | 0.0073 | 0.025 | | |
| 131: | 12 | 4 | 8 | 11 | 4 | 7 | 4868.5481 | 0.0005 | 0.025 | | |

```
 132: 13  1 13   12  1 12                  4882.9540  -0.0041  0.025
 133: 13  0 13   12  0 12                  4883.2139  -0.0042  0.025
 134: 12  3  9   11  3  8                  4912.1304   0.0070  0.025
 135: 13  2 12   12  2 11                  5015.6275  -0.0035  0.025
 136: 13  3 11   12  3 10                  5131.2041   0.0000  0.025
 137: 13 10  3   12 10  2                  5176.9741   0.0018  0.025   0.0018 0.50
 138: 13 10  4   12 10  3                  5176.9741   0.0018  0.025   0.0018 0.50
 139: 13  9  5   12  9  4                  5179.8879   0.0012  0.025   0.0012 0.50
 140: 13  9  4   12  9  3                  5179.8879   0.0012  0.025   0.0012 0.50
 141: 13  8  6   12  8  5                  5184.0091   0.0039  0.025   0.0029 0.50
 142: 13  8  5   12  8  4                  5184.0091   0.0017  0.025   0.0029 0.50
 143: 13  2 11   12  2 10                  5188.9443   0.0071  0.025
 144: 13  7  7   12  7  6                  5190.1037   0.0007  0.025
 145: 13  7  6   12  7  5                  5190.1795   0.0036  0.025
 146: 13  4 10   12  4  9                  5196.9295   0.0013  0.025
 147: 13  6  8   12  6  7                  5199.2352   0.0008  0.025
 148: 13  6  7   12  6  6                  5200.7770   0.0012  0.025
 149: 13  5  9   12  5  8                  5208.6875   0.0009  0.025
 150: 13  5  8   12  5  7                  5227.5313  -0.0002  0.025
 151: 14  1 14   13  1 13                  5253.4106  -0.0021  0.025
 152: 14  0 14   13  0 13                  5253.5402  -0.0022  0.025
 153: 13  4  9   12  4  8                  5296.5305   0.0004  0.025
 154: 13  3 10   12  3  9                  5314.0989   0.0089  0.025
 155: 14  2 13   13  2 12                  5387.1636  -0.0052  0.025
 156: 14  1 13   13  1 12                  5391.3257  -0.0033  0.025
 157:  9  3  7    8  1  8                  5485.0661  -0.0005  0.025
 158: 14  3 12   13  3 11                  5509.9902  -0.0006  0.025
 159: 14  2 12   13  2 11                  5551.9680   0.0059  0.025
 160: 14 12  2   13 12  1                  5572.5654   0.0016  0.025   0.0016 0.50
 161: 14 12  3   13 12  2                  5572.5654   0.0016  0.025   0.0016 0.50
 162: 14 11  3   13 11  2                  5574.5958   0.0024  0.025   0.0024 0.50
 163: 14 11  4   13 11  3                  5574.5958   0.0024  0.025   0.0024 0.50
 164: 14 10  4   13 10  3                  5577.2725  -0.0014  0.025  -0.0015 0.50
 165: 14 10  5   13 10  4                  5577.2725  -0.0014  0.025  -0.0015 0.50
 166: 14  9  6   13  9  5                  5580.9273   0.0023  0.025   0.0022 0.50
 167: 14  9  5   13  9  4                  5580.9273   0.0021  0.025   0.0022 0.50
 168: 14  8  7   13  8  6                  5586.0999   0.0040  0.025   0.0003 0.50
 169: 14  8  6   13  8  5                  5586.0999  -0.0035  0.025   0.0003 0.50
 170: 14  4 11   13  4 10                  5590.4433   0.0015  0.025
 171: 14  7  8   13  7  7                  5593.7457   0.0022  0.025
 172: 14  7  7   13  7  6                  5593.9475   0.0004  0.025
 173: 14  6  8   13  6  7                  5608.2297  -0.0004  0.025
 174: 14  5 10   13  5  9                  5612.9062   0.0006  0.025
 175: 15  1 15   14  1 14                  5623.8327  -0.0017  0.025
 176: 15  0 15   14  0 14                  5623.8917  -0.0067  0.025
 177: 14  5  9   13  5  8                  5647.0809  -0.0030  0.025
 178: 14  3 11   13  3 10                  5705.7823   0.0108  0.025
 179: 14  4 10   13  4  9                  5722.9673   0.0022  0.025
 180: 15  2 14   14  2 13                  5758.1606  -0.0057  0.025
 181: 15  1 14   14  1 13                  5760.4642  -0.0044  0.025
 182: 15  3 13   14  3 12                  5886.1912  -0.0019  0.025
 183: 11  3  8   10  1  9                  5913.8428   0.0013  0.025
 184: 15  2 13   14  2 12                  5914.5402   0.0018  0.025
 185: 15 13  2   14 13  1                  5970.2982  -0.0019  0.025  -0.0019 0.50
 186: 15 13  3   14 13  2                  5970.2982  -0.0019  0.025  -0.0019 0.50
 187: 15 12  3   14 12  2                  5972.2397   0.0005  0.025   0.0006 0.50
 188: 15 12  4   14 12  3                  5972.2397   0.0005  0.025   0.0006 0.50
 189: 15 11  4   14 11  3                  5974.7454   0.0069  0.025   0.0069 0.50
 190: 15 11  5   14 11  4                  5974.7454   0.0069  0.025   0.0069 0.50
 191: 15 10  6   14 10  5                  5978.0452   0.0021  0.025   0.0022 0.50
 192: 15 10  5   14 10  4                  5978.0452   0.0021  0.025   0.0022 0.50
 193: 15  4 12   14  4 11                  5980.3547   0.0009  0.025
 194: 15  9  7   14  9  6                  5982.5533   0.0013  0.025   0.0010 0.50
 195: 15  9  6   14  9  5                  5982.5533   0.0006  0.025   0.0010 0.50
 196: 15  8  8   14  8  7                  5988.9639   0.0108  0.025  -0.0008 0.50
 197: 15  8  7   14  8  6                  5988.9639  -0.0123  0.025  -0.0008 0.50
 198: 16  1 16   15  1 15                  5994.2183  -0.0182  0.025
 199: 16  0 16   15  0 15                  5994.2735   0.0056  0.025
 200: 15  7  9   14  7  8                  5998.3735   0.0020  0.025
 201: 15  7  8   14  7  7                  5998.8896  -0.0012  0.025
```

```
202: 15  6 10   14  6  9        6010.9740  -0.0002  0.025
203: 15  5 11   14  5 10        6015.8189   0.0001  0.025
204: 15  6  9   14  6  8        6018.3716  -0.0023  0.025
205: 15  5 10   14  5  9        6072.6701  -0.0039  0.025
206: 15  3 12   14  3 11        6086.5664   0.0124  0.025
207: 16  2 15   15  2 14        6128.8337  -0.0078  0.025
208: 16  1 15   15  1 14        6130.0808  -0.0068  0.025
209: 15  4 11   14  4 10        6143.7466   0.0051  0.025
210: 16  3 14   15  3 13        6260.3572  -0.0037  0.025
211: 17  1 17   16  1 16        6364.6299   0.0046  0.025  -0.0029 0.50
212: 17  0 17   16  0 16        6364.6299  -0.0105  0.025  -0.0029 0.50
213: 16  4 13   15  4 12        6366.5315  -0.0001  0.025  -0.0009 0.25
214: 16 15  1   15 15  0        6366.5315  -0.0011  0.025  -0.0009 0.25
215: 16 15  1   15 15  0        6366.5315  -0.0011  0.025  -0.0009 0.25
216: 16 15  2   15 15  1        6366.5315  -0.0011  0.025  -0.0009 0.25
217: 16 13  3   15 13  2        6369.9036   0.0032  0.025   0.0032 0.50
218: 16 13  4   15 13  3        6369.9036   0.0032  0.025   0.0032 0.50
219: 16 12  4   15 12  3        6372.2570   0.0015  0.025   0.0015 0.50
220: 16 12  5   15 12  4        6372.2570   0.0015  0.025   0.0015 0.50
221: 16 11  5   15 11  4        6375.2974   0.0032  0.025   0.0032 0.50
222: 16 11  6   15 11  5        6375.2974   0.0032  0.025   0.0032 0.50
223: 16 10  7   15 10  6        6379.3173   0.0001  0.025   0.0001 0.50
224: 16 10  6   15 10  5        6379.3173   0.0001  0.025   0.0001 0.50
225: 16  9  8   15  9  7        6384.8185   0.0003  0.025  -0.0008 0.50
226: 16  9  7   15  9  6        6384.8185  -0.0020  0.025  -0.0008 0.50
227: 16  8  9   15  8  8        6392.6515   0.0073  0.025
228: 16  8  8   15  8  7        6392.6856  -0.0240  0.025
229: 16  7 10   15  7  9        6404.0188  -0.0004  0.025
230: 16  7  9   15  7  8        6405.2416  -0.0033  0.025
231: 16  5 12   15  5 11        6416.6091  -0.0003  0.025
232: 16  6 11   15  6 10        6417.6588  -0.0013  0.025
233: 16  6 10   15  6  9        6432.1562  -0.0054  0.025
234: 16  3 13   15  3 12        6457.3030   0.0130  0.025
235: 17  2 16   16  2 15        6499.3270  -0.0067  0.025
236: 17  1 16   16  1 15        6499.9874  -0.0087  0.025
237: 16  5 11   15  5 10        6503.2584  -0.0046  0.025
238: 16  4 12   15  4 11        6555.9055   0.0075  0.025
239: 17  3 15   16  3 14        6633.0277  -0.0063  0.025
240: 17  2 15   16  2 14        6644.0239  -0.0046  0.025
241: 18  1 18   17  1 17        6735.0049   0.0014  0.025  -0.0022 0.50
242: 18  0 18   17  0 17        6735.0049  -0.0058  0.025  -0.0022 0.50
243: 17  4 14   16  4 13        6749.0954  -0.0010  0.025
244: 17 13  4   16 13  3        6769.8161   0.0076  0.025   0.0076 0.50
245: 17 13  5   16 13  4        6769.8161   0.0076  0.025   0.0076 0.50
246: 17 12  5   16 12  4        6772.6373   0.0000  0.025  -0.0001 0.50
247: 17 12  6   16 12  5        6772.6373   0.0000  0.025  -0.0001 0.50
248: 17 11  7   16 11  6        6776.2907  -0.0001  0.025  -0.0001 0.50
249: 17 11  6   16 11  5        6776.2907  -0.0001  0.025  -0.0001 0.50
250: 17 10  8   16 10  7        6781.1334  -0.0023  0.025  -0.0024 0.50
251: 17 10  7   16 10  6        6781.1334  -0.0025  0.025  -0.0024 0.50
252: 17  9  9   16  9  8        6787.7803   0.0031  0.025  -0.0005 0.50
253: 17  9  8   16  9  7        6787.7803  -0.0041  0.025  -0.0005 0.50
254: 17  8 10   16  8  9        6797.2356  -0.0007  0.025
255: 17  8  9   16  8  8        6797.4024  -0.0040  0.025
256: 17  7 11   16  7 10        6810.6628  -0.0018  0.025
257: 17  7 10   16  7  9        6813.3579  -0.0050  0.025
258: 17  5 13   16  5 12        6814.5275  -0.0006  0.025
259: 17  3 14   16  3 13        6820.7609   0.0126  0.025
260: 17  6 12   16  6 11        6824.2533   0.0000  0.025
261: 17  6 11   16  6 10        6850.7556  -0.0078  0.025
262: 18  2 17   17  2 16        6869.7184  -0.0081  0.025
263: 18  1 17   17  1 16        6870.0646  -0.0088  0.025
264: 17  5 12   16  5 11        6935.6854  -0.0051  0.025
265: 17  4 13   16  4 12        6957.5515   0.0111  0.025
266: 18  3 16   17  3 15        7004.6724  -0.0068  0.025
267: 18  2 16   17  2 15        7011.1420  -0.0057  0.025
268: 19  1 19   18  1 18        7105.3749   0.0031  0.025   0.0014 0.50
269: 19  0 19   18  0 18        7105.3749  -0.0003  0.025   0.0014 0.50
270: 18  4 15   17  4 14        7128.3879  -0.0020  0.025
271: 18 13  6   17 13  5        7170.0403  -0.0050  0.025
```

| | | | | | | | | | | | |
|---|---|---|---|---|---|---|---|---|---|---|---|
| 272: | 18 | 12 | 6 | 17 | 12 | 5 | 7173.4056 | -0.0040 | 0.025 | -0.0040 | 0.50 |
| 273: | 18 | 12 | 7 | 17 | 12 | 6 | 7173.4056 | -0.0040 | 0.025 | -0.0040 | 0.50 |
| 274: | 18 | 11 | 8 | 17 | 11 | 7 | 7177.7554 | -0.0046 | 0.025 | -0.0046 | 0.50 |
| 275: | 18 | 11 | 7 | 17 | 11 | 6 | 7177.7554 | -0.0046 | 0.025 | -0.0046 | 0.50 |
| 276: | 18 | 3 | 15 | 17 | 3 | 14 | 7180.8125 | 0.0096 | 0.025 | | |
| 277: | 18 | 10 | 9 | 17 | 10 | 8 | 7183.5391 | -0.0012 | 0.025 | -0.0016 | 0.50 |
| 278: | 18 | 10 | 8 | 17 | 10 | 7 | 7183.5391 | -0.0019 | 0.025 | -0.0016 | 0.50 |
| 279: | 18 | 9 | 10 | 17 | 9 | 9 | 7191.4934 | 0.0073 | 0.025 | -0.0030 | 0.50 |
| 280: | 18 | 9 | 9 | 17 | 9 | 8 | 7191.4934 | -0.0134 | 0.025 | -0.0030 | 0.50 |
| 281: | 18 | 8 | 11 | 17 | 8 | 10 | 7202.7872 | -0.0030 | 0.025 | | |
| 282: | 18 | 8 | 10 | 17 | 8 | 9 | 7203.1911 | -0.0112 | 0.025 | | |
| 283: | 18 | 5 | 14 | 17 | 5 | 13 | 7208.9839 | -0.0006 | 0.025 | | |
| 284: | 18 | 7 | 12 | 17 | 7 | 11 | 7218.1945 | -0.0023 | 0.025 | | |
| 285: | 18 | 7 | 11 | 17 | 7 | 10 | 7223.7648 | -0.0087 | 0.025 | | |
| 286: | 18 | 6 | 13 | 17 | 6 | 12 | 7230.0662 | -0.0035 | 0.025 | | |
| 287: | 19 | 2 | 18 | 18 | 2 | 17 | 7240.0604 | -0.0069 | 0.025 | | |
| 288: | 19 | 1 | 18 | 18 | 1 | 17 | 7240.2363 | -0.0103 | 0.025 | | |
| 289: | 18 | 6 | 12 | 17 | 6 | 11 | 7275.2353 | -0.0113 | 0.025 | | |
| 290: | 18 | 4 | 14 | 17 | 4 | 13 | 7347.5294 | 0.0256 | 0.025 | | |
| 291: | 18 | 5 | 13 | 17 | 5 | 12 | 7365.4366 | -0.0012 | 0.025 | | |
| 292: | 19 | 3 | 17 | 18 | 3 | 16 | 7375.6512 | -0.0097 | 0.025 | | |
| 293: | 19 | 2 | 17 | 18 | 2 | 16 | 7379.3570 | -0.0077 | 0.025 | | |
| 294: | 20 | 1 | 20 | 19 | 1 | 19 | 7475.7346 | 0.0041 | 0.025 | 0.0033 | 0.50 |
| 295: | 20 | 0 | 20 | 19 | 0 | 19 | 7475.7346 | 0.0024 | 0.025 | 0.0033 | 0.50 |
| 296: | 19 | 4 | 16 | 18 | 4 | 15 | 7504.9068 | -0.0047 | 0.025 | | |
| 297: | 19 | 3 | 16 | 18 | 3 | 15 | 7540.7963 | 0.0060 | 0.025 | | |
| 298: | 19 | 13 | 6 | 18 | 13 | 5 | 7570.6350 | 0.0032 | 0.025 | 0.0032 | 0.50 |
| 299: | 19 | 13 | 7 | 18 | 13 | 6 | 7570.6350 | 0.0032 | 0.025 | 0.0032 | 0.50 |
| 300: | 19 | 12 | 7 | 18 | 12 | 6 | 7574.5880 | -0.0101 | 0.025 | -0.0102 | 0.50 |
| 301: | 19 | 12 | 8 | 18 | 12 | 7 | 7574.5880 | -0.0101 | 0.025 | -0.0102 | 0.50 |
| 302: | 19 | 11 | 8 | 18 | 11 | 7 | 7579.7301 | -0.0048 | 0.025 | -0.0048 | 0.50 |
| 303: | 19 | 11 | 9 | 18 | 11 | 8 | 7579.7301 | -0.0047 | 0.025 | -0.0048 | 0.50 |
| 304: | 19 | 10 | 10 | 18 | 10 | 9 | 7586.5731 | -0.0023 | 0.025 | -0.0035 | 0.50 |
| 305: | 19 | 10 | 9 | 18 | 10 | 8 | 7586.5731 | -0.0046 | 0.025 | -0.0035 | 0.50 |
| 306: | 19 | 5 | 15 | 18 | 5 | 14 | 7599.6128 | -0.0001 | 0.025 | | |
| 307: | 19 | 8 | 12 | 18 | 8 | 11 | 7609.3421 | -0.0066 | 0.025 | | |
| 308: | 19 | 8 | 11 | 18 | 8 | 10 | 7610.2793 | -0.0073 | 0.025 | | |
| 309: | 20 | 2 | 19 | 19 | 2 | 18 | 7610.3723 | -0.0088 | 0.025 | | |
| 310: | 20 | 1 | 19 | 19 | 1 | 18 | 7610.4626 | -0.0102 | 0.025 | | |
| 311: | 19 | 7 | 13 | 18 | 7 | 12 | 7626.3735 | -0.0061 | 0.025 | | |
| 312: | 19 | 6 | 14 | 18 | 6 | 13 | 7634.3140 | -0.0040 | 0.025 | | |
| 313: | 19 | 7 | 12 | 18 | 7 | 11 | 7637.2327 | -0.0102 | 0.025 | | |
| 314: | 19 | 6 | 13 | 18 | 6 | 12 | 7705.8449 | -0.0138 | 0.025 | | |
| 315: | 13 | 4 | 10 | 12 | 2 | 11 | 7708.9239 | 0.0087 | 0.025 | | |
| 316: | 19 | 4 | 15 | 18 | 4 | 14 | 7725.5772 | 0.0178 | 0.025 | | |
| 317: | 20 | 3 | 18 | 19 | 3 | 17 | 7746.2285 | -0.0114 | 0.025 | | |
| 318: | 20 | 2 | 18 | 19 | 2 | 17 | 7748.3035 | -0.0112 | 0.025 | | |
| 319: | 19 | 5 | 14 | 18 | 5 | 13 | 7788.2295 | 0.0028 | 0.025 | | |
| 320: | 21 | 1 | 21 | 20 | 1 | 20 | 7846.0954 | 0.0163 | 0.025 | 0.0159 | 0.50 |
| 321: | 21 | 0 | 21 | 20 | 0 | 20 | 7846.0954 | 0.0155 | 0.025 | 0.0159 | 0.50 |
| 322: | 20 | 4 | 17 | 19 | 4 | 16 | 7879.2298 | -0.0049 | 0.025 | | |
| 323: | 20 | 3 | 17 | 19 | 3 | 16 | 7902.5149 | 0.0010 | 0.025 | | |
| 324: | 20 | 11 | 10 | 19 | 11 | 9 | 7982.2378 | -0.0123 | 0.025 | -0.0125 | 0.50 |
| 325: | 20 | 11 | 9 | 19 | 11 | 8 | 7982.2378 | -0.0125 | 0.025 | -0.0125 | 0.50 |
| 326: | 20 | 5 | 16 | 19 | 5 | 15 | 7986.3032 | -0.0013 | 0.025 | | |
| 327: | 20 | 10 | 11 | 19 | 10 | 10 | 7990.2825 | -0.0062 | 0.025 | -0.0095 | 0.50 |
| 328: | 20 | 10 | 10 | 19 | 10 | 9 | 7990.2825 | -0.0126 | 0.025 | -0.0095 | 0.50 |
| 329: | 20 | 8 | 13 | 19 | 8 | 12 | 8016.9212 | 0.0021 | 0.025 | | |
| 330: | 20 | 8 | 12 | 19 | 8 | 11 | 8018.9257 | -0.0097 | 0.025 | | |
| 331: | 20 | 6 | 15 | 19 | 6 | 14 | 8036.1907 | 0.0049 | 0.025 | | |
| 332: | 21 | 3 | 19 | 20 | 3 | 18 | 8116.5901 | 0.0001 | 0.025 | | |
| 333: | 21 | 2 | 19 | 20 | 2 | 18 | 8117.7251 | -0.0060 | 0.025 | | |
| 334: | 20 | 6 | 14 | 19 | 6 | 13 | 8141.0932 | -0.0051 | 0.025 | | |
| 335: | 22 | 1 | 22 | 21 | 1 | 21 | 8216.4363 | 0.0192 | 0.025 | 0.0190 | 0.50 |
| 336: | 22 | 0 | 22 | 21 | 0 | 21 | 8216.4363 | 0.0188 | 0.025 | 0.0190 | 0.50 |
| 337: | 21 | 4 | 18 | 20 | 4 | 17 | 8251.9291 | 0.0039 | 0.025 | | |
| 338: | 21 | 3 | 18 | 20 | 3 | 17 | 8266.4201 | 0.0094 | 0.025 | | |
| 339: | 22 | 2 | 21 | 21 | 2 | 20 | 8350.9823 | 0.0107 | 0.025 | -0.0009 | 0.50 |
| 340: | 22 | 1 | 21 | 21 | 1 | 20 | 8350.9823 | -0.0126 | 0.025 | -0.0009 | 0.50 |
| 341: | 21 | 14 | 8 | 20 | 14 | 7 | 8368.6923 | -0.0002 | 0.025 | | |

```
 342: 21  5 17   20  5 16                     8369.2131   0.0076  0.025
 343: 21  9 13   20  9 12                     8407.7372   0.0178  0.025
 344: 21  9 12   20  9 11                     8408.0331  -0.0015  0.025
 345: 21  6 16   20  6 15                     8434.9379   0.0003  0.025
 346: 21  7 14   20  7 13                     8477.6177  -0.0104  0.025
 347: 22  3 20   21  3 19                     8486.8143  -0.0039  0.025
 348: 22  2 20   21  2 19                     8487.4331  -0.0032  0.025
 349: 21  6 15   20  6 14                     8577.3494  -0.0053  0.025
 350: 21  5 16   20  5 15                     8601.8915   0.0245  0.025
 351: 22  4 19   21  4 18                     8623.4765  -0.0025  0.025
 352: 22  3 19   21  3 18                     8632.2004  -0.0026  0.025
 353: 23  2 22   22  2 21                     8721.2661   0.0106  0.025   0.0048 0.50
 354: 23  1 22   22  1 21                     8721.2661  -0.0009  0.025   0.0048 0.50
 355: 22  5 18   21  5 17                     8748.6864   0.0043  0.025
 356: 22 14  8   21 14  7                     8769.8162   0.0068  0.025   0.0068 0.50
 357: 22 14  9   21 14  8                     8769.8162   0.0068  0.025   0.0068 0.50
 358: 22 13  9   21 13  8                     8774.7072  -0.0012  0.025  -0.0012 0.50
 359: 22 13 10   21 13  9                     8774.7072  -0.0012  0.025  -0.0012 0.50
 360: 22 12 10   21 12  9                     8780.9362  -0.0009  0.025  -0.0010 0.50
 361: 22 12 11   21 12 10                     8780.9362  -0.0009  0.025  -0.0010 0.50
 362: 22 11 12   21 11 11                     8789.0531  -0.0002  0.025  -0.0013 0.50
 363: 22 11 11   21 11 10                     8789.0531  -0.0022  0.025  -0.0013 0.50
 364: 22  4 18   21  4 17                     8810.5194   0.0233  0.025
 365: 22  9 14   21  9 13                     8815.0261   0.0025  0.025
 366: 22  9 13   21  9 12                     8815.7216  -0.0009  0.025
 367: 22  6 17   21  6 16                     8830.0134   0.0099  0.025
 368: 22  8 15   21  8 14                     8834.7797   0.0008  0.025
 369: 23  3 21   22  3 20                     8856.9808  -0.0071  0.025
 370: 23  2 21   22  2 20                     8857.3086  -0.0096  0.025
 371: 22  7 15   21  7 14                     8906.6612  -0.0168  0.025
 372: 24  1 24   23  1 23                     8957.0834   0.0246  0.025   0.0246 0.50
 373: 24  0 24   23  0 23                     8957.0834   0.0245  0.025   0.0246 0.50
 374: 22  5 17   21  5 16                     8989.8618   0.0336  0.025
 375: 23  4 20   22  4 19                     8994.2857  -0.0058  0.025
 376: 23  3 20   22  3 19                     8999.4037  -0.0068  0.025
 377: 22  6 16   21  6 15                     9009.8121  -0.0065  0.025
 378: 23  5 19   22  5 18                     9125.2588   0.0045  0.025
 379: 23  4 19   22  4 18                     9167.9886   0.0165  0.025
 380: 23 14  9   22 14  8                     9171.2916  -0.0070  0.025  -0.0070 0.50
 381: 23 14 10   22 14  9                     9171.2916  -0.0070  0.025  -0.0070 0.50
 382: 23 13 10   22 13  9                     9176.9615   0.0442  0.025   0.0443 0.50
 383: 23 13 11   22 13 10                     9176.9615   0.0443  0.025   0.0443 0.50
 384: 23 12 12   22 12 11                     9184.0674  -0.0062  0.025  -0.0063 0.50
 385: 23 12 11   22 12 10                     9184.0674  -0.0064  0.025  -0.0063 0.50
 386: 23 11 13   22 11 12                     9193.4162  -0.0078  0.025  -0.0105 0.50
 387: 23 11 12   22 11 11                     9193.4162  -0.0132  0.025  -0.0105 0.50
 388: 23  7 17   22  7 16                     9255.3841  -0.0004  0.025
 389: 23  8 15   22  8 14                     9259.3631  -0.0130  0.025
 390: 25  0 25   24  0 24                     9327.3847   0.0233  0.025   0.0233 0.50
 391: 25  1 25   24  1 24                     9327.3847   0.0233  0.025   0.0233 0.50
 392: 23  7 16   22  7 15                     9341.8685  -0.0223  0.025
 393: 24  3 21   23  3 20                     9367.5773  -0.0148  0.025
 394: 23  6 17   22  6 16                     9434.0861   0.0165  0.025
 395: 26  0 26   25  0 25                     9697.6845   0.0338  0.025   0.0338 0.50
 396: 26  1 26   25  1 25                     9697.6845   0.0338  0.025   0.0338 0.50
 397: 24  5 19   23  5 18                     9729.3487   0.0257  0.025
 398: 25  4 22   24  4 21                     9734.7424  -0.0144  0.025
 399: 25  3 22   24  3 21                     9736.3992  -0.0164  0.025
 400: 24  7 17   23  7 16                     9781.2996  -0.0176  0.025
 401: 29  0 29   28  0 28                    10808.4179  -0.0161  0.025  -0.0162 0.50
 402: 29  1 29   28  1 28                    10808.4179  -0.0161  0.025  -0.0162 0.50
 403: 29  2 28   28  2 27                    10942.7843   0.0000  0.025   0.0000 0.50
 404: 29  1 28   28  1 27                    10942.7843  -0.0001  0.025   0.0000 0.50
-------------------------------------------------------------------------------

PARAMETERS IN FIT (values truncated and Nlines statistics):

       10000           A  /MHz          356.111(2)                            1
       20000           B  /MHz          211.5252(1)                           2
       30000           C  /MHz          185.2338(1)                           3
```

```
         200          DJ  /kHz          0.0211(2)                          4
        1100         DJK  /kHz          0.013(1)                           5
        2000          DK  /kHz          0.32(5)                            6
       40100          d1  /kHz        [ 0.]                                7
       50000          d2  /kHz         -0.00075(8)                         8

 MICROWAVE AVG =        0.000125 MHz, IR AVG =       0.00000
 MICROWAVE RMS =        0.008229 MHz, IR RMS =       0.00000
 END OF ITERATION  1 OLD, NEW RMS ERROR=       0.32915         0.32915

  distinct frequency lines in fit:   328
       distinct parameters of fit:     7

                                        upper state  lower state         overall
     limits of quantum number  1:       4   29       3   28            3   29
     limits of quantum number  2:       0   15       0   15            0   15
     limits of quantum number  3:       1   29       0   28            0   29

                 frequency range:        2030        10942


 PARAMETERS IN FIT WITH STANDARD ERRORS ON THOSE THAT ARE FITTED:
 (values rounded and degrees of freedom, Ndegf=Nlines-Nconst, statistics)

       10000           A  /MHz        356.1116(7)                          1
       20000           B  /MHz        211.52526(4)                         2
       30000           C  /MHz        185.23390(5)                         3
         200          DJ  /kHz          0.02118(5)                         4
        1100         DJK  /kHz          0.0135(4)                          5
        2000          DK  /kHz          0.33(2)                            6
       40100          d1  /kHz        [ 0.]                                7
       50000          d2  /kHz         -0.00076(3)                         8

 CORRELATION COEFFICIENTS, C.ij:

           A        B        C        -DJ      -DJK     -DK      d2

A           1.0000
B           0.1489   1.0000
C          -0.2098   0.2800   1.0000
-DJ         0.4020  -0.4272  -0.7790   1.0000
-DJK       -0.2712  -0.3802   0.0283  -0.2931   1.0000
-DK        -0.9046  -0.0253   0.2122  -0.4851   0.2381   1.0000
d2         -0.2951  -0.0195   0.2382  -0.3967   0.2872   0.4412   1.0000

 Mean value of |C.ij|, i.ne.j =   0.3220
 Mean value of  C.ij,  i.ne.j =  -0.1053


 No correlations with absolute value greater than 0.9950


 Worst fitted lines (obs-calc/error):

   382:    1.8       395:    1.4       374:    1.3        30:    1.2
   397:    1.0       290:    1.0       372:    1.0       350:    1.0
   111:    1.0       228:   -1.0       390:    0.9       364:    0.9
    55:   -0.9       392:   -0.9       335:    0.8       198:   -0.7
   343:    0.7       316:    0.7       400:   -0.7       371:   -0.7
   379:    0.7       394:    0.7       399:   -0.7       128:    0.7
   401:   -0.6       320:    0.6       393:   -0.6       398:   -0.6
   314:   -0.6       234:    0.5       389:   -0.5       259:    0.5
   324:   -0.5       206:    0.5        65:   -0.5        18:   -0.5
   317:   -0.5       289:   -0.5       318:   -0.4       282:   -0.4
   265:    0.4       178:    0.4       386:   -0.4       346:   -0.4
   288:   -0.4       310:   -0.4       313:   -0.4       300:   -0.4
   367:    0.4       292:   -0.4

   382: 23 13 10   22 13  9                    9176.9615   0.0442  0.025   0.0443 0.50
   395: 26  0 26   25  0 25                    9697.6845   0.0338  0.025   0.0338 0.50
```

```
   374: 22  5 17   21  5 16                        8989.8618   0.0336  0.025
    30: 14  4 10   13  6  7                        2885.6978   0.0302  0.025
   397: 24  5 19   23  5 18                        9729.3487   0.0257  0.025
   290: 18  4 14   17  4 13                        7347.5294   0.0256  0.025
   372: 24  1 24   23  1 23                        8957.0834   0.0246  0.025   0.0246 0.50
   350: 21  5 16   20  5 15                        8601.8915   0.0245  0.025
   111: 12  1 12   11  0 11                        4513.4976   0.0241  0.025
   228: 16  8  8   15  8  7                        6392.6856  -0.0240  0.025
                                               _____________________________________
__________________________________________/ SPFIT output reformatted with PIFORM
```

Hexamer - 6 II

| | | | | | | | obs | o-c | error | blends o-c | blends wt | Notes |
|---|---|---|---|---|---|---|---|---|---|---|---|---|
| 1: | 5 | 0 | 5 | 4 | 0 | 4 | 2320.1607 | 0.0006 | 0.025 | | | |
| 2: | 5 | 1 | 5 | 4 | 1 | 4 | 2319.0224 | 0.0008 | 0.025 | | | |
| 3: | 4 | 4 | 0 | 3 | 3 | 0 | 2380.2291 | 0.0013 | 0.025 | | | |
| 4: | 4 | 4 | 1 | 3 | 3 | 1 | 2384.8246 | 0.0016 | 0.025 | | | |
| 5: | 5 | 2 | 4 | 4 | 2 | 3 | 2421.6726 | 0.0023 | 0.025 | | | |
| 6: | 5 | 1 | 4 | 4 | 1 | 3 | 2443.4540 | 0.0014 | 0.025 | | | |
| 7: | 5 | 3 | 3 | 4 | 3 | 2 | 2487.3030 | 0.0004 | 0.025 | | | |
| 8: | 5 | 2 | 3 | 4 | 2 | 2 | 2551.8423 | 0.0017 | 0.025 | | | |
| 9: | 5 | 3 | 2 | 4 | 3 | 1 | 2561.5284 | 0.0022 | 0.025 | | | |
| 10: | 5 | 3 | 2 | 4 | 2 | 2 | 2766.4299 | 0.0007 | 0.025 | | | |
| 11: | 6 | 1 | 6 | 5 | 1 | 5 | 2772.1027 | 0.0012 | 0.025 | | | |
| 12: | 6 | 0 | 6 | 5 | 0 | 5 | 2772.3761 | -0.0006 | 0.025 | | | |
| 13: | 5 | 2 | 4 | 4 | 1 | 4 | 2816.1894 | -0.0052 | 0.025 | | | |
| 14: | 5 | 3 | 3 | 4 | 2 | 3 | 2830.6010 | 0.0016 | 0.025 | | | |
| 15: | 5 | 4 | 1 | 4 | 3 | 1 | 2869.3147 | 0.0010 | 0.025 | | | |
| 16: | 6 | 2 | 5 | 5 | 2 | 4 | 2881.0088 | -0.0040 | 0.025 | | | |
| 17: | 5 | 4 | 2 | 4 | 3 | 2 | 2892.7518 | 0.0002 | 0.025 | | | |
| 18: | 6 | 3 | 4 | 5 | 3 | 3 | 2967.9987 | 0.0004 | 0.025 | | | |
| 19: | 5 | 5 | 0 | 4 | 4 | 0 | 2991.4022 | 0.0021 | 0.025 | | | |
| 20: | 5 | 5 | 1 | 4 | 4 | 1 | 2992.7231 | 0.0009 | 0.025 | | | |
| 21: | 6 | 4 | 3 | 5 | 4 | 2 | 3006.3459 | 0.0020 | 0.025 | | | |
| 22: | 6 | 2 | 4 | 5 | 2 | 3 | 3021.3158 | 0.0009 | 0.025 | | | |
| 23: | 6 | 4 | 2 | 5 | 4 | 1 | 3048.7648 | 0.0007 | 0.025 | | | |
| 24: | 6 | 3 | 3 | 5 | 3 | 2 | 3085.5278 | 0.0004 | 0.025 | | | |
| 25: | 7 | 1 | 7 | 6 | 1 | 6 | 3224.8897 | 0.0047 | 0.025 | | | |
| 26: | 7 | 0 | 7 | 6 | 0 | 6 | 3224.9504 | 0.0036 | 0.025 | | | |
| 27: | 6 | 3 | 3 | 5 | 2 | 3 | 3300.1179 | 0.0017 | 0.025 | | | |
| 28: | 7 | 2 | 6 | 6 | 2 | 5 | 3336.1150 | 0.0018 | 0.025 | | | |
| 29: | 7 | 1 | 6 | 6 | 1 | 5 | 3338.7539 | 0.0007 | 0.025 | | | |
| 30: | 6 | 2 | 4 | 5 | 1 | 4 | 3339.5122 | -0.0020 | 0.025 | | | |
| 31: | 6 | 4 | 2 | 5 | 3 | 2 | 3356.5537 | 0.0021 | 0.025 | | | |
| 32: | 6 | 1 | 5 | 5 | 0 | 5 | 3374.9585 | 0.0145 | 0.025 | | | |
| 33: | 6 | 3 | 4 | 5 | 2 | 4 | 3376.9246 | -0.0026 | 0.025 | | | |
| 34: | 6 | 2 | 5 | 5 | 1 | 5 | 3378.1931 | 0.0073 | 0.025 | | | |
| 35: | 6 | 4 | 3 | 5 | 3 | 3 | 3411.7944 | 0.0016 | 0.025 | | | |
| 36: | 7 | 3 | 5 | 6 | 3 | 4 | 3437.4611 | 0.0015 | 0.025 | | | |
| 37: | 7 | 2 | 5 | 6 | 2 | 4 | 3468.1691 | 0.0010 | 0.025 | | | |
| 38: | 6 | 5 | 1 | 5 | 4 | 1 | 3485.3921 | 0.0017 | 0.025 | | | |
| 39: | 6 | 5 | 2 | 5 | 4 | 2 | 3495.0874 | 0.0005 | 0.025 | | | |
| 40: | 7 | 4 | 4 | 6 | 4 | 3 | 3500.9234 | 0.0010 | 0.025 | | | |
| 41: | 7 | 5 | 3 | 6 | 5 | 2 | 3514.9466 | 0.0014 | 0.025 | | | |
| 42: | 7 | 5 | 2 | 6 | 5 | 1 | 3533.2162 | 0.0009 | 0.025 | | | |
| 43: | 7 | 3 | 4 | 6 | 3 | 3 | 3584.0155 | -0.0088 | 0.025 | | | |
| 44: | 7 | 4 | 3 | 6 | 4 | 2 | 3590.5206 | 0.0012 | 0.025 | | | |
| 45: | 6 | 6 | 0 | 5 | 5 | 0 | 3601.3896 | 0.0033 | 0.025 | | | |
| 46: | 6 | 6 | 1 | 5 | 5 | 1 | 3601.7217 | 0.0000 | 0.025 | | | |
| 47: | 8 | 1 | 8 | 7 | 1 | 7 | 3677.6040 | 0.0079 | 0.025 | 0.0013 | 0.50 | |
| 48: | 8 | 0 | 8 | 7 | 0 | 7 | 3677.6040 | -0.0052 | 0.025 | 0.0013 | 0.50 | |
| 49: | 8 | 2 | 7 | 7 | 2 | 6 | 3789.4704 | 0.0007 | 0.025 | | | |
| 50: | 8 | 1 | 7 | 7 | 1 | 6 | 3790.1890 | -0.0003 | 0.025 | | | |
| 51: | 7 | 4 | 3 | 6 | 3 | 3 | 3861.5444 | 0.0008 | 0.025 | | | |
| 52: | 7 | 3 | 4 | 6 | 2 | 4 | 3862.8250 | -0.0005 | 0.025 | | | |
| 53: | 8 | 3 | 6 | 7 | 3 | 5 | 3898.2066 | 0.0003 | 0.025 | | | |
| 54: | 8 | 2 | 6 | 7 | 2 | 5 | 3910.9794 | -0.0007 | 0.025 | | | |
| 55: | 7 | 2 | 5 | 6 | 1 | 5 | 3918.1705 | 0.0016 | 0.025 | | | |
| 56: | 7 | 3 | 5 | 6 | 2 | 5 | 3933.3743 | 0.0003 | 0.025 | | | |
| 57: | 7 | 1 | 6 | 6 | 0 | 6 | 3941.3211 | 0.0007 | 0.025 | | | |
| 58: | 7 | 4 | 4 | 6 | 3 | 4 | 3944.7194 | 0.0024 | 0.025 | | | |
| 59: | 7 | 5 | 2 | 6 | 4 | 2 | 3969.8431 | 0.0015 | 0.025 | | | |
| 60: | 8 | 4 | 5 | 7 | 4 | 4 | 3983.8753 | -0.0003 | 0.025 | | | |
| 61: | 7 | 5 | 3 | 6 | 4 | 3 | 4003.6912 | 0.0030 | 0.025 | | | |
| 62: | 8 | 7 | 2 | 7 | 7 | 1 | 4005.4857 | 0.0051 | 0.025 | | | |
| 63: | 8 | 7 | 1 | 7 | 7 | 0 | 4005.7703 | -0.0054 | 0.025 | | | |
| 64: | 8 | 6 | 3 | 7 | 6 | 2 | 4017.4005 | 0.0036 | 0.025 | | | |

| | | | | | | | | | | | |
|---|---|---|---|---|---|---|---|---|---|---|---|
| 65: | 8 | 5 | 4 | 7 | 5 | 3 | 4020.3803 | 0.0009 | 0.025 | | |
| 66: | 8 | 6 | 2 | 7 | 6 | 1 | 4023.8291 | 0.0014 | 0.025 | | |
| 67: | 8 | 3 | 5 | 7 | 3 | 4 | 4050.7207 | 0.0001 | 0.025 | | |
| 68: | 8 | 5 | 3 | 7 | 5 | 2 | 4073.5178 | -0.0009 | 0.025 | | |
| 69: | 7 | 6 | 1 | 6 | 5 | 1 | 4098.6263 | 0.0022 | 0.025 | | |
| 70: | 7 | 6 | 2 | 6 | 5 | 2 | 4101.8023 | 0.0015 | 0.025 | | |
| 71: | 8 | 4 | 4 | 7 | 4 | 3 | 4118.5662 | 0.0002 | 0.025 | | |
| 72: | 9 | 1 | 9 | 8 | 1 | 8 | 4130.2941 | 0.0033 | 0.025 | 0.0020 | 0.50 |
| 73: | 9 | 0 | 9 | 8 | 0 | 8 | 4130.2941 | 0.0006 | 0.025 | 0.0020 | 0.50 |
| 74: | 7 | 7 | 0 | 6 | 6 | 0 | 4210.9715 | 0.0040 | 0.025 | | |
| 75: | 7 | 7 | 1 | 6 | 6 | 1 | 4211.0468 | 0.0003 | 0.025 | | |
| 76: | 9 | 2 | 8 | 8 | 2 | 7 | 4242.2736 | 0.0031 | 0.025 | | |
| 77: | 9 | 1 | 8 | 8 | 1 | 7 | 4242.4520 | 0.0009 | 0.025 | | |
| 78: | 9 | 3 | 7 | 8 | 3 | 6 | 4353.8897 | 0.0002 | 0.025 | | |
| 79: | 9 | 2 | 7 | 8 | 2 | 6 | 4358.1846 | -0.0004 | 0.025 | | |
| 80: | 8 | 4 | 4 | 7 | 3 | 4 | 4396.0872 | 0.0020 | 0.025 | | |
| 81: | 8 | 3 | 5 | 7 | 2 | 5 | 4445.3789 | 0.0008 | 0.025 | | |
| 82: | 8 | 5 | 3 | 7 | 4 | 3 | 4452.8397 | -0.0012 | 0.025 | | |
| 83: | 9 | 4 | 6 | 8 | 4 | 5 | 4454.8550 | 0.0000 | 0.025 | | |
| 84: | 8 | 2 | 6 | 7 | 1 | 6 | 4490.3971 | 0.0012 | 0.025 | | |
| 85: | 8 | 4 | 5 | 7 | 3 | 5 | 4491.1334 | 0.0003 | 0.025 | | |
| 86: | 9 | 3 | 6 | 8 | 3 | 5 | 4493.8325 | 0.0004 | 0.025 | | |
| 87: | 8 | 3 | 6 | 7 | 2 | 6 | 4495.4676 | 0.0004 | 0.025 | | |
| 88: | 8 | 1 | 7 | 7 | 0 | 7 | 4506.5685 | 0.0055 | 0.025 | | |
| 89: | 8 | 2 | 7 | 7 | 1 | 7 | 4506.7792 | -0.0029 | 0.025 | | |
| 90: | 9 | 5 | 5 | 8 | 5 | 4 | 4517.0882 | 0.0015 | 0.025 | | |
| 91: | 8 | 5 | 4 | 7 | 4 | 4 | 4523.1460 | 0.0007 | 0.025 | | |
| 92: | 9 | 6 | 3 | 8 | 6 | 2 | 4553.3473 | -0.0006 | 0.025 | | |
| 93: | 10 | 1 | 10 | 9 | 1 | 9 | 4582.9814 | 0.0010 | 0.025 | 0.0008 | 0.50 |
| 94: | 10 | 0 | 10 | 9 | 0 | 9 | 4582.9814 | 0.0004 | 0.025 | 0.0008 | 0.50 |
| 95: | 8 | 6 | 2 | 7 | 5 | 2 | 4589.2366 | 0.0001 | 0.025 | | |
| 96: | 8 | 6 | 3 | 7 | 5 | 3 | 4604.2528 | 0.0004 | 0.025 | | |
| 97: | 9 | 4 | 5 | 8 | 4 | 4 | 4617.1250 | -0.0008 | 0.025 | | |
| 98: | 9 | 5 | 4 | 8 | 5 | 3 | 4621.3470 | 0.0006 | 0.025 | | |
| 99: | 10 | 2 | 9 | 9 | 2 | 8 | 4694.9414 | 0.0082 | 0.025 | | |
| 100: | 10 | 1 | 9 | 9 | 1 | 8 | 4694.9748 | -0.0012 | 0.025 | | |
| 101: | 8 | 7 | 1 | 7 | 6 | 1 | 4709.5067 | 0.0011 | 0.025 | | |
| 102: | 8 | 7 | 2 | 7 | 6 | 2 | 4710.4175 | 0.0015 | 0.025 | | |
| 103: | 8 | 5 | 3 | 7 | 3 | 4 | 4730.3574 | -0.0028 | 0.025 | | |
| 104: | 10 | 3 | 8 | 9 | 3 | 7 | 4807.3441 | -0.0051 | 0.025 | | |
| 105: | 10 | 2 | 8 | 9 | 2 | 7 | 4808.6180 | -0.0014 | 0.025 | | |
| 106: | 8 | 8 | 0 | 7 | 7 | 0 | 4820.4389 | 0.0098 | 0.025 | 0.0010 | 0.50 |
| 107: | 8 | 8 | 1 | 7 | 7 | 1 | 4820.4389 | -0.0078 | 0.025 | 0.0010 | 0.50 |
| 108: | 10 | 4 | 7 | 9 | 4 | 6 | 4916.4417 | -0.0004 | 0.025 | | |
| 109: | 10 | 3 | 7 | 9 | 3 | 6 | 4933.3681 | -0.0009 | 0.025 | | |
| 110: | 9 | 5 | 4 | 8 | 4 | 4 | 4955.6221 | 0.0007 | 0.025 | | |
| 111: | 9 | 4 | 5 | 8 | 3 | 5 | 4962.4940 | 0.0034 | 0.025 | | |
| 112: | 10 | 5 | 6 | 9 | 5 | 5 | 5001.5710 | -0.0012 | 0.025 | | |
| 113: | 10 | 8 | 3 | 9 | 8 | 2 | 5015.9749 | 0.0005 | 0.025 | | |
| 114: | 10 | 8 | 2 | 9 | 8 | 1 | 5016.5391 | -0.0034 | 0.025 | | |
| 115: | 9 | 3 | 6 | 8 | 2 | 6 | 5028.2363 | 0.0063 | 0.025 | | |
| 116: | 10 | 7 | 4 | 9 | 7 | 3 | 5031.3323 | 0.0120 | 0.025 | | |
| 117: | 11 | 1 | 11 | 10 | 1 | 10 | 5035.6688 | 0.0024 | 0.025 | 0.0024 | 0.50 |
| 118: | 11 | 0 | 11 | 10 | 0 | 10 | 5035.6688 | 0.0023 | 0.025 | 0.0024 | 0.50 |
| 119: | 10 | 6 | 5 | 9 | 6 | 4 | 5036.7621 | 0.0000 | 0.025 | | |
| 120: | 10 | 7 | 3 | 9 | 7 | 2 | 5040.4025 | 0.0039 | 0.025 | | |
| 121: | 9 | 4 | 6 | 8 | 3 | 6 | 5047.7834 | 0.0015 | 0.025 | | |
| 122: | 9 | 5 | 5 | 8 | 4 | 5 | 5056.3559 | -0.0002 | 0.025 | | |
| 123: | 9 | 2 | 7 | 8 | 1 | 7 | 5058.3921 | 0.0004 | 0.025 | | |
| 124: | 9 | 3 | 7 | 8 | 2 | 7 | 5059.8856 | -0.0014 | 0.025 | | |
| 125: | 9 | 6 | 3 | 8 | 5 | 3 | 5069.0652 | -0.0003 | 0.025 | | |
| 126: | 10 | 4 | 6 | 9 | 4 | 5 | 5080.9131 | -0.0031 | 0.025 | | |
| 127: | 10 | 6 | 4 | 9 | 6 | 3 | 5100.3243 | -0.0004 | 0.025 | | |
| 128: | 9 | 6 | 4 | 8 | 5 | 4 | 5113.0120 | 0.0006 | 0.025 | | |
| 129: | 11 | 2 | 10 | 10 | 2 | 9 | 5147.5806 | 0.0067 | 0.025 | 0.0019 | 0.50 |
| 130: | 11 | 1 | 10 | 10 | 1 | 9 | 5147.5806 | -0.0030 | 0.025 | 0.0019 | 0.50 |
| 131: | 10 | 5 | 5 | 9 | 5 | 4 | 5152.6339 | 0.0000 | 0.025 | | |
| 132: | 9 | 7 | 2 | 8 | 6 | 2 | 5204.7952 | 0.0010 | 0.025 | | |
| 133: | 9 | 7 | 3 | 8 | 6 | 3 | 5210.1421 | 0.0007 | 0.025 | | |
| 134: | 11 | 3 | 9 | 10 | 3 | 8 | 5260.0647 | 0.0019 | 0.025 | | |

| | | | | | | | | | | | |
|---|---|---|---|---|---|---|---|---|---|---|---|
| 135: | 11 | 2 | 9 | 10 | 2 | 8 | 5260.4068 | -0.0009 | 0.025 | | |
| 136: | 9 | 5 | 4 | 8 | 3 | 5 | 5300.9849 | -0.0011 | 0.025 | | |
| 137: | 9 | 8 | 1 | 8 | 7 | 1 | 5319.4331 | 0.0040 | 0.025 | | |
| 138: | 9 | 8 | 2 | 8 | 7 | 2 | 5319.6671 | -0.0018 | 0.025 | | |
| 139: | 11 | 4 | 8 | 10 | 4 | 7 | 5372.4103 | -0.0002 | 0.025 | | |
| 140: | 11 | 3 | 8 | 10 | 3 | 7 | 5378.4292 | -0.0012 | 0.025 | | |
| 141: | 9 | 9 | 0 | 8 | 8 | 0 | 5429.8556 | 0.0039 | 0.025 | 0.0021 | 0.50 |
| 142: | 9 | 9 | 1 | 8 | 8 | 1 | 5429.8556 | 0.0001 | 0.025 | 0.0021 | 0.50 |
| 143: | 11 | 5 | 7 | 10 | 5 | 6 | 5473.5297 | -0.0020 | 0.025 | | |
| 144: | 12 | 0 | 12 | 11 | 0 | 11 | 5488.3518 | 0.0039 | 0.025 | 0.0039 | 0.50 |
| 145: | 12 | 1 | 12 | 11 | 1 | 11 | 5488.3518 | 0.0039 | 0.025 | 0.0039 | 0.50 |
| 146: | 10 | 5 | 5 | 9 | 4 | 5 | 5491.1316 | 0.0022 | 0.025 | | |
| 147: | 11 | 4 | 7 | 10 | 4 | 6 | 5520.2642 | -0.0032 | 0.025 | | |
| 148: | 11 | 8 | 4 | 10 | 8 | 3 | 5530.4543 | 0.0015 | 0.025 | | |
| 149: | 11 | 8 | 3 | 10 | 8 | 2 | 5533.4544 | 0.0001 | 0.025 | | |
| 150: | 11 | 7 | 5 | 10 | 7 | 4 | 5545.4505 | -0.0023 | 0.025 | | |
| 151: | 10 | 6 | 4 | 9 | 5 | 4 | 5548.0445 | 0.0005 | 0.025 | | |
| 152: | 10 | 4 | 6 | 9 | 3 | 6 | 5549.5757 | 0.0009 | 0.025 | | |
| 153: | 11 | 7 | 4 | 10 | 7 | 3 | 5575.6416 | -0.0004 | 0.025 | | |
| 154: | 12 | 2 | 11 | 11 | 2 | 10 | 5600.2198 | 0.0030 | 0.025 | 0.0020 | 0.50 |
| 155: | 12 | 1 | 11 | 11 | 1 | 10 | 5600.2198 | 0.0009 | 0.025 | 0.0020 | 0.50 |
| 156: | 10 | 5 | 6 | 9 | 4 | 6 | 5603.0738 | 0.0003 | 0.025 | | |
| 157: | 10 | 3 | 7 | 9 | 2 | 7 | 5603.4145 | 0.0005 | 0.025 | | |
| 158: | 10 | 4 | 7 | 9 | 3 | 7 | 5610.3349 | 0.0002 | 0.025 | | |
| 159: | 10 | 2 | 8 | 9 | 1 | 8 | 5624.5629 | 0.0029 | 0.025 | | |
| 160: | 10 | 3 | 8 | 9 | 2 | 8 | 5624.9617 | -0.0042 | 0.025 | | |
| 161: | 10 | 6 | 5 | 9 | 5 | 5 | 5632.6866 | -0.0001 | 0.025 | | |
| 162: | 10 | 1 | 9 | 9 | 0 | 9 | 5636.0950 | 0.0077 | 0.025 | 0.0018 | 0.50 |
| 163: | 10 | 2 | 9 | 9 | 1 | 9 | 5636.0950 | -0.0040 | 0.025 | 0.0018 | 0.50 |
| 164: | 11 | 5 | 6 | 10 | 5 | 5 | 5650.8918 | -0.0001 | 0.025 | | |
| 165: | 11 | 6 | 5 | 10 | 6 | 4 | 5653.4929 | -0.0008 | 0.025 | | |
| 166: | 10 | 7 | 3 | 9 | 6 | 3 | 5691.8454 | 0.0005 | 0.025 | | |
| 167: | 10 | 7 | 4 | 9 | 6 | 4 | 5712.3229 | -0.0002 | 0.025 | | |
| 168: | 12 | 3 | 10 | 11 | 3 | 9 | 5712.6003 | 0.0032 | 0.025 | | |
| 169: | 12 | 2 | 10 | 11 | 2 | 9 | 5712.6876 | 0.0024 | 0.025 | | |
| 170: | 10 | 8 | 2 | 9 | 7 | 2 | 5816.8558 | 0.0005 | 0.025 | | |
| 171: | 10 | 8 | 3 | 9 | 7 | 3 | 5818.5209 | -0.0001 | 0.025 | | |
| 172: | 12 | 4 | 9 | 11 | 4 | 8 | 5825.8135 | -0.0009 | 0.025 | | |
| 173: | 12 | 3 | 9 | 11 | 3 | 8 | 5827.6996 | -0.0039 | 0.025 | | |
| 174: | 10 | 6 | 4 | 9 | 4 | 5 | 5886.5344 | -0.0050 | 0.025 | | |
| 175: | 10 | 9 | 1 | 9 | 8 | 1 | 5929.0417 | 0.0178 | 0.025 | -0.0119 | 0.50 |
| 176: | 10 | 9 | 2 | 9 | 8 | 2 | 5929.0417 | -0.0417 | 0.025 | -0.0119 | 0.50 |
| 177: | 12 | 5 | 8 | 11 | 5 | 7 | 5935.5990 | -0.0006 | 0.025 | | |
| 178: | 13 | 0 | 13 | 12 | 0 | 12 | 5941.0288 | 0.0045 | 0.025 | 0.0045 | 0.50 |
| 179: | 13 | 1 | 13 | 12 | 1 | 12 | 5941.0288 | 0.0045 | 0.025 | 0.0045 | 0.50 |
| 180: | 12 | 4 | 8 | 11 | 4 | 7 | 5956.5449 | -0.0016 | 0.025 | | |
| 181: | 10 | 5 | 5 | 9 | 3 | 6 | 5959.7955 | 0.0076 | 0.025 | | |
| 182: | 12 | 10 | 3 | 11 | 10 | 2 | 6013.4735 | 0.0053 | 0.025 | -0.0141 | 0.50 |
| 183: | 12 | 10 | 2 | 11 | 10 | 1 | 6013.4735 | -0.0335 | 0.025 | -0.0141 | 0.50 |
| 184: | 12 | 6 | 7 | 11 | 6 | 6 | 6020.6576 | 0.0002 | 0.025 | | |
| 185: | 12 | 9 | 4 | 11 | 9 | 3 | 6028.5967 | 0.0012 | 0.025 | | |
| 186: | 12 | 9 | 3 | 11 | 9 | 2 | 6029.5045 | -0.0018 | 0.025 | | |
| 187: | 10 | 10 | 0 | 9 | 9 | 0 | 6039.2583 | 0.0025 | 0.025 | 0.0021 | 0.50 |
| 188: | 10 | 10 | 1 | 9 | 9 | 1 | 6039.2583 | 0.0017 | 0.025 | 0.0021 | 0.50 |
| 189: | 12 | 8 | 5 | 11 | 8 | 4 | 6047.1929 | -0.0015 | 0.025 | | |
| 190: | 11 | 6 | 5 | 10 | 5 | 5 | 6048.9041 | 0.0003 | 0.025 | | |
| 191: | 13 | 2 | 12 | 12 | 2 | 11 | 6052.8662 | 0.0029 | 0.025 | 0.0027 | 0.50 |
| 192: | 13 | 1 | 12 | 12 | 1 | 11 | 6052.8662 | 0.0024 | 0.025 | 0.0027 | 0.50 |
| 193: | 12 | 7 | 6 | 11 | 7 | 5 | 6054.8837 | -0.0002 | 0.025 | | |
| 194: | 12 | 8 | 4 | 11 | 8 | 3 | 6059.0696 | -0.0013 | 0.025 | | |
| 195: | 11 | 5 | 6 | 10 | 4 | 6 | 6061.1051 | 0.0000 | 0.025 | | |
| 196: | 12 | 5 | 7 | 11 | 5 | 6 | 6111.7065 | -0.0021 | 0.025 | | |
| 197: | 12 | 7 | 5 | 11 | 7 | 4 | 6128.6890 | 0.0037 | 0.025 | | |
| 198: | 11 | 4 | 7 | 10 | 3 | 7 | 6136.4740 | 0.0007 | 0.025 | | |
| 199: | 11 | 5 | 7 | 10 | 4 | 7 | 6160.1627 | -0.0003 | 0.025 | | |
| 200: | 13 | 3 | 11 | 12 | 3 | 10 | 6165.1313 | 0.0115 | 0.025 | 0.0008 | 0.50 |
| 201: | 13 | 2 | 11 | 12 | 2 | 10 | 6165.1313 | -0.0099 | 0.025 | 0.0008 | 0.50 |
| 202: | 11 | 6 | 6 | 10 | 5 | 6 | 6166.1935 | -0.0001 | 0.025 | | |
| 203: | 11 | 7 | 4 | 10 | 6 | 4 | 6167.1615 | -0.0006 | 0.025 | | |
| 204: | 11 | 3 | 8 | 10 | 2 | 8 | 6173.2250 | 0.0000 | 0.025 | | |

| | | | | | | | | | | | | |
|---|---|---|---|---|---|---|---|---|---|---|---|---|
| 205: | 11 | 4 | 8 | 10 | 3 | 8 | 6175.3950 | -0.0008 | 0.025 | | |
| 206: | 12 | 6 | 6 | 11 | 6 | 5 | 6187.4685 | -0.0007 | 0.025 | | |
| 207: | 11 | 2 | 9 | 10 | 1 | 9 | 6190.0000 | 0.0082 | 0.025 | | |
| 208: | 11 | 3 | 9 | 10 | 2 | 9 | 6190.0836 | -0.0118 | 0.025 | | |
| 209: | 11 | 1 | 10 | 10 | 0 | 10 | 6200.6950 | 0.0050 | 0.025 | 0.0038 | 0.50 |
| 210: | 11 | 2 | 10 | 10 | 1 | 10 | 6200.6950 | 0.0025 | 0.025 | 0.0038 | 0.50 |
| 211: | 11 | 7 | 5 | 10 | 6 | 5 | 6221.0133 | -0.0008 | 0.025 | | |
| 212: | 13 | 4 | 10 | 12 | 4 | 9 | 6278.3325 | -0.0031 | 0.025 | | |
| 213: | 13 | 3 | 10 | 12 | 3 | 9 | 6278.8756 | -0.0048 | 0.025 | | |
| 214: | 11 | 8 | 3 | 10 | 7 | 3 | 6309.9112 | 0.0002 | 0.025 | | |
| 215: | 11 | 8 | 4 | 10 | 7 | 4 | 6317.6515 | -0.0020 | 0.025 | | |
| 216: | 13 | 5 | 9 | 12 | 5 | 8 | 6391.6375 | -0.0014 | 0.025 | | |
| 217: | 14 | 0 | 14 | 13 | 0 | 13 | 6393.6992 | 0.0044 | 0.025 | 0.0044 | 0.50 |
| 218: | 14 | 1 | 14 | 13 | 1 | 13 | 6393.6992 | 0.0044 | 0.025 | 0.0044 | 0.50 |
| 219: | 11 | 9 | 2 | 10 | 8 | 2 | 6427.3194 | 0.0001 | 0.025 | | |
| 220: | 11 | 9 | 3 | 10 | 8 | 3 | 6427.7946 | -0.0001 | 0.025 | | |
| 221: | 11 | 6 | 5 | 10 | 4 | 6 | 6459.1185 | 0.0015 | 0.025 | | |
| 222: | 13 | 6 | 8 | 12 | 6 | 7 | 6493.2501 | -0.0026 | 0.025 | | |
| 223: | 14 | 2 | 13 | 13 | 2 | 12 | 6505.5126 | 0.0017 | 0.025 | 0.0017 | 0.50 |
| 224: | 14 | 1 | 13 | 13 | 1 | 12 | 6505.5126 | 0.0016 | 0.025 | 0.0017 | 0.50 |
| 225: | 13 | 11 | 3 | 12 | 11 | 2 | 6512.3558 | 0.0037 | 0.025 | -0.0010 | 0.50 |
| 226: | 13 | 11 | 2 | 12 | 11 | 1 | 6512.3558 | -0.0058 | 0.025 | -0.0010 | 0.50 |
| 227: | 13 | 10 | 4 | 12 | 10 | 3 | 6526.6295 | 0.0055 | 0.025 | | |
| 228: | 13 | 10 | 3 | 12 | 10 | 2 | 6526.8797 | -0.0038 | 0.025 | | |
| 229: | 11 | 10 | 1 | 10 | 9 | 1 | 6538.5101 | 0.0084 | 0.025 | 0.0013 | 0.50 |
| 230: | 11 | 10 | 2 | 10 | 9 | 2 | 6538.5101 | -0.0057 | 0.025 | 0.0013 | 0.50 |
| 231: | 13 | 9 | 5 | 12 | 9 | 4 | 6545.6095 | -0.0011 | 0.025 | | |
| 232: | 13 | 5 | 8 | 12 | 5 | 7 | 6547.3236 | -0.0031 | 0.025 | | |
| 233: | 13 | 9 | 4 | 12 | 9 | 3 | 6549.7336 | -0.0037 | 0.025 | | |
| 234: | 13 | 7 | 7 | 12 | 7 | 6 | 6554.4669 | -0.0029 | 0.025 | | |
| 235: | 13 | 8 | 6 | 12 | 8 | 5 | 6563.3956 | -0.0033 | 0.025 | | |
| 236: | 12 | 6 | 6 | 11 | 5 | 6 | 6585.4848 | 0.0036 | 0.025 | | |
| 237: | 13 | 8 | 5 | 12 | 8 | 4 | 6599.6202 | -0.0030 | 0.025 | | |
| 238: | 14 | 3 | 12 | 13 | 3 | 11 | 6617.6682 | 0.0030 | 0.025 | 0.0006 | 0.50 |
| 239: | 14 | 2 | 12 | 13 | 2 | 11 | 6617.6682 | -0.0019 | 0.025 | 0.0006 | 0.50 |
| 240: | 12 | 7 | 5 | 11 | 6 | 5 | 6642.3549 | 0.0012 | 0.025 | | |
| 241: | 11 | 11 | 0 | 10 | 10 | 0 | 6648.6463 | -0.0001 | 0.025 | -0.0002 | 0.50 |
| 242: | 11 | 11 | 1 | 10 | 10 | 1 | 6648.6463 | -0.0002 | 0.025 | -0.0002 | 0.50 |
| 243: | 12 | 5 | 7 | 11 | 4 | 7 | 6652.5470 | 0.0008 | 0.025 | | |
| 244: | 13 | 6 | 7 | 12 | 6 | 6 | 6685.1621 | 0.0018 | 0.025 | | |
| 245: | 12 | 6 | 7 | 11 | 5 | 7 | 6713.3186 | -0.0006 | 0.025 | | |
| 246: | 12 | 4 | 8 | 11 | 3 | 8 | 6714.5902 | 0.0008 | 0.025 | | |
| 247: | 14 | 4 | 11 | 13 | 4 | 10 | 6730.6650 | 0.0055 | 0.025 | | |
| 248: | 14 | 3 | 11 | 13 | 3 | 10 | 6730.8030 | -0.0040 | 0.025 | | |
| 249: | 12 | 3 | 9 | 11 | 2 | 9 | 6740.5195 | -0.0014 | 0.025 | | |
| 250: | 12 | 7 | 6 | 11 | 6 | 6 | 6740.8170 | -0.0019 | 0.025 | | |
| 251: | 12 | 4 | 9 | 11 | 3 | 9 | 6741.1435 | -0.0039 | 0.025 | | |
| 252: | 12 | 2 | 10 | 11 | 1 | 10 | 6755.1028 | 0.0095 | 0.025 | -0.0031 | 0.50 |
| 253: | 12 | 3 | 10 | 11 | 2 | 10 | 6755.1028 | -0.0157 | 0.025 | -0.0031 | 0.50 |
| 254: | 12 | 1 | 11 | 11 | 0 | 11 | 6765.2378 | -0.0046 | 0.025 | -0.0049 | 0.50 |
| 255: | 12 | 2 | 11 | 11 | 1 | 11 | 6765.2378 | -0.0051 | 0.025 | -0.0049 | 0.50 |
| 256: | 12 | 8 | 4 | 11 | 7 | 4 | 6793.3394 | -0.0005 | 0.025 | | |
| 257: | 12 | 8 | 5 | 11 | 7 | 5 | 6819.3931 | -0.0019 | 0.025 | | |
| 258: | 14 | 5 | 10 | 13 | 5 | 9 | 6844.8573 | -0.0017 | 0.025 | | |
| 259: | 15 | 0 | 15 | 14 | 0 | 14 | 6846.3628 | 0.0040 | 0.025 | 0.0041 | 0.50 |
| 260: | 15 | 1 | 15 | 14 | 1 | 14 | 6846.3628 | 0.0040 | 0.025 | 0.0041 | 0.50 |
| 261: | 14 | 4 | 10 | 13 | 4 | 9 | 6847.4067 | -0.0057 | 0.025 | | |
| 262: | 12 | 9 | 3 | 11 | 8 | 3 | 6923.3720 | 0.0006 | 0.025 | | |
| 263: | 12 | 9 | 4 | 11 | 8 | 4 | 6925.9303 | -0.0072 | 0.025 | | |
| 264: | 14 | 6 | 9 | 13 | 6 | 8 | 6955.5510 | -0.0059 | 0.025 | | |
| 265: | 15 | 1 | 14 | 14 | 1 | 13 | 6958.1455 | -0.0115 | 0.025 | -0.0116 | 0.50 |
| 266: | 15 | 2 | 14 | 14 | 2 | 13 | 6958.1455 | -0.0115 | 0.025 | -0.0116 | 0.50 |
| 267: | 14 | 5 | 9 | 13 | 5 | 8 | 6980.3858 | -0.0044 | 0.025 | | |
| 268: | 12 | 10 | 2 | 11 | 9 | 2 | 7037.1438 | -0.0269 | 0.025 | | |
| 269: | 12 | 10 | 3 | 11 | 9 | 3 | 7037.2981 | -0.0001 | 0.025 | | |
| 270: | 14 | 7 | 8 | 13 | 7 | 7 | 7040.8519 | 0.0049 | 0.025 | | |
| 271: | 14 | 10 | 5 | 13 | 10 | 4 | 7042.9360 | 0.0000 | 0.025 | | |
| 272: | 14 | 10 | 4 | 13 | 10 | 3 | 7044.2443 | -0.0081 | 0.025 | | |
| 273: | 14 | 9 | 6 | 13 | 9 | 5 | 7064.6176 | 0.0019 | 0.025 | | |
| 274: | 15 | 3 | 13 | 14 | 3 | 12 | 7070.2325 | 0.0004 | 0.025 | -0.0001 | 0.50 |

| | | | | | | | | | | | |
|---|---|---|---|---|---|---|---|---|---|---|---|
| 275: | 15 | 2 | 13 | 14 | 2 | 12 | 7070.2325 | -0.0006 | 0.025 | -0.0001 | 0.50 |
| 276: | 14 | 8 | 7 | 13 | 8 | 6 | 7074.3610 | -0.0019 | 0.025 | | |
| 277: | 14 | 9 | 5 | 13 | 9 | 4 | 7079.4231 | 0.0007 | 0.025 | | |
| 278: | 13 | 7 | 6 | 12 | 6 | 6 | 7141.5423 | 0.0004 | 0.025 | | |
| 279: | 14 | 6 | 8 | 13 | 6 | 7 | 7142.9588 | -0.0036 | 0.025 | | |
| 280: | 12 | 11 | 1 | 11 | 10 | 1 | 7147.9300 | 0.0014 | 0.025 | -0.0002 | 0.50 |
| 281: | 12 | 11 | 2 | 11 | 10 | 2 | 7147.9300 | -0.0018 | 0.025 | -0.0002 | 0.50 |
| 282: | 14 | 8 | 6 | 13 | 8 | 5 | 7158.2806 | -0.0023 | 0.025 | | |
| 283: | 14 | 7 | 7 | 13 | 7 | 6 | 7222.9021 | -0.0011 | 0.025 | | |
| 284: | 13 | 5 | 8 | 12 | 4 | 8 | 7243.3256 | -0.0007 | 0.025 | | |
| 285: | 12 | 12 | 0 | 11 | 11 | 0 | 7258.0248 | 0.0004 | 0.025 | 0.0004 | 0.50 |
| 286: | 12 | 12 | 1 | 11 | 11 | 1 | 7258.0248 | 0.0004 | 0.025 | 0.0004 | 0.50 |
| 287: | 13 | 8 | 5 | 12 | 7 | 5 | 7264.2770 | -0.0009 | 0.025 | | |
| 288: | 13 | 6 | 8 | 12 | 5 | 8 | 7270.9703 | -0.0020 | 0.025 | | |
| 289: | 13 | 7 | 7 | 12 | 6 | 7 | 7274.6296 | -0.0018 | 0.025 | | |
| 290: | 13 | 4 | 9 | 12 | 3 | 9 | 7286.2950 | 0.0002 | 0.025 | | |
| 291: | 13 | 5 | 9 | 12 | 4 | 9 | 7289.1758 | -0.0008 | 0.025 | | |
| 292: | 15 | 5 | 11 | 14 | 5 | 10 | 7297.0941 | -0.0042 | 0.025 | | |
| 293: | 15 | 4 | 11 | 14 | 4 | 10 | 7297.8541 | -0.0162 | 0.025 | | |
| 294: | 16 | 0 | 16 | 15 | 0 | 15 | 7299.0191 | 0.0036 | 0.025 | 0.0036 | 0.50 |
| 295: | 16 | 1 | 16 | 15 | 1 | 15 | 7299.0191 | 0.0036 | 0.025 | 0.0036 | 0.50 |
| 296: | 13 | 3 | 10 | 12 | 2 | 10 | 7306.7267 | 0.0105 | 0.025 | | |
| 297: | 13 | 4 | 10 | 12 | 3 | 10 | 7306.8820 | -0.0041 | 0.025 | | |
| 298: | 13 | 2 | 11 | 12 | 1 | 11 | 7320.0222 | 0.0065 | 0.025 | 0.0036 | 0.50 |
| 299: | 13 | 3 | 11 | 12 | 2 | 11 | 7320.0222 | 0.0006 | 0.025 | 0.0036 | 0.50 |
| 300: | 13 | 8 | 6 | 12 | 7 | 6 | 7327.9075 | -0.0024 | 0.025 | | |
| 301: | 13 | 1 | 12 | 12 | 0 | 12 | 7329.7536 | -0.0047 | 0.025 | -0.0048 | 0.50 |
| 302: | 13 | 2 | 12 | 12 | 1 | 12 | 7329.7536 | -0.0048 | 0.025 | -0.0048 | 0.50 |
| 303: | 16 | 1 | 15 | 15 | 1 | 14 | 7410.7998 | 0.0002 | 0.025 | 0.0002 | 0.50 |
| 304: | 16 | 2 | 15 | 15 | 2 | 14 | 7410.7998 | 0.0002 | 0.025 | 0.0002 | 0.50 |
| 305: | 15 | 6 | 10 | 14 | 6 | 9 | 7411.5068 | -0.0042 | 0.025 | | |
| 306: | 13 | 9 | 4 | 12 | 8 | 4 | 7414.0352 | -0.0024 | 0.025 | | |
| 307: | 15 | 5 | 10 | 14 | 5 | 9 | 7421.0248 | -0.0086 | 0.025 | | |
| 308: | 13 | 9 | 5 | 12 | 8 | 5 | 7424.3484 | -0.0052 | 0.025 | | |
| 309: | 15 | 7 | 9 | 14 | 7 | 8 | 7513.8336 | -0.0076 | 0.025 | | |
| 310: | 16 | 3 | 14 | 15 | 3 | 13 | 7522.8116 | -0.0019 | 0.025 | -0.0021 | 0.50 |
| 311: | 16 | 2 | 14 | 15 | 2 | 13 | 7522.8116 | -0.0022 | 0.025 | -0.0021 | 0.50 |
| 312: | 13 | 10 | 3 | 12 | 9 | 3 | 7534.5437 | -0.0041 | 0.025 | | |
| 313: | 15 | 10 | 6 | 14 | 10 | 5 | 7562.2526 | 0.0006 | 0.025 | | |
| 314: | 15 | 10 | 5 | 14 | 10 | 4 | 7567.5995 | -0.0078 | 0.025 | | |
| 315: | 15 | 9 | 7 | 14 | 9 | 6 | 7582.6461 | -0.0033 | 0.025 | | |
| 316: | 15 | 9 | 6 | 14 | 9 | 5 | 7624.9719 | -0.0019 | 0.025 | | |
| 317: | 16 | 4 | 13 | 15 | 4 | 12 | 7635.3907 | 0.0011 | 0.025 | -0.0036 | 0.50 |
| 318: | 16 | 3 | 13 | 15 | 3 | 12 | 7635.3907 | -0.0083 | 0.025 | -0.0036 | 0.50 |
| 319: | 13 | 11 | 2 | 12 | 10 | 2 | 7646.7885 | 0.0053 | 0.025 | -0.0110 | 0.50 |
| 320: | 13 | 11 | 3 | 12 | 10 | 3 | 7646.7885 | -0.0273 | 0.025 | -0.0110 | 0.50 |
| 321: | 14 | 7 | 7 | 13 | 6 | 7 | 7679.2835 | -0.0014 | 0.025 | | |
| 322: | 15 | 7 | 8 | 14 | 7 | 7 | 7719.8130 | -0.0055 | 0.025 | | |
| 323: | 15 | 8 | 7 | 14 | 8 | 6 | 7720.6351 | -0.0028 | 0.025 | | |
| 324: | 14 | 8 | 6 | 13 | 7 | 6 | 7735.9018 | -0.0015 | 0.025 | | |
| 325: | 16 | 5 | 12 | 15 | 5 | 11 | 7749.1441 | 0.0051 | 0.025 | | |
| 326: | 16 | 4 | 12 | 15 | 4 | 11 | 7749.3449 | -0.0134 | 0.025 | | |
| 327: | 17 | 0 | 17 | 16 | 0 | 16 | 7751.6657 | 0.0011 | 0.025 | 0.0012 | 0.50 |
| 328: | 17 | 1 | 17 | 16 | 1 | 16 | 7751.6657 | 0.0011 | 0.025 | 0.0012 | 0.50 |
| 329: | 14 | 6 | 8 | 13 | 5 | 8 | 7754.5669 | -0.0014 | 0.025 | | |
| 330: | 13 | 12 | 1 | 12 | 11 | 1 | 7757.3254 | 0.0001 | 0.025 | -0.0002 | 0.50 |
| 331: | 13 | 12 | 2 | 12 | 11 | 2 | 7757.3254 | -0.0005 | 0.025 | -0.0002 | 0.50 |
| 332: | 14 | 7 | 8 | 13 | 6 | 8 | 7822.2220 | -0.0036 | 0.025 | | |
| 333: | 14 | 5 | 9 | 13 | 4 | 9 | 7824.3089 | 0.0012 | 0.025 | | |
| 334: | 14 | 6 | 9 | 13 | 5 | 9 | 7834.8867 | -0.0037 | 0.025 | | |
| 335: | 14 | 8 | 7 | 13 | 7 | 7 | 7847.8005 | -0.0026 | 0.025 | | |
| 336: | 14 | 4 | 10 | 13 | 3 | 10 | 7854.8241 | -0.0027 | 0.025 | | |
| 337: | 14 | 5 | 10 | 13 | 4 | 10 | 7855.6986 | -0.0013 | 0.025 | | |
| 338: | 17 | 1 | 16 | 16 | 1 | 15 | 7863.4321 | -0.0049 | 0.025 | -0.0049 | 0.50 |
| 339: | 17 | 2 | 16 | 16 | 2 | 15 | 7863.4321 | -0.0049 | 0.025 | -0.0049 | 0.50 |
| 340: | 16 | 6 | 11 | 15 | 6 | 10 | 7864.4446 | -0.0029 | 0.025 | | |
| 341: | 13 | 13 | 0 | 12 | 12 | 0 | 7867.3892 | 0.0000 | 0.025 | 0.0000 | 0.50 |
| 342: | 13 | 13 | 1 | 12 | 12 | 1 | 7867.3892 | 0.0000 | 0.025 | 0.0000 | 0.50 |
| 343: | 16 | 5 | 11 | 15 | 5 | 10 | 7867.6866 | -0.0083 | 0.025 | | |
| 344: | 14 | 2 | 12 | 13 | 1 | 12 | 7884.8148 | -0.0072 | 0.025 | -0.0080 | 0.50 |

| | | | | | | | | | | | |
|---|---|---|---|---|---|---|---|---|---|---|---|
| 345: | 14 | 3 | 12 | 13 | 2 | 12 | 7884.8148 | -0.0086 | 0.025 | -0.0080 | 0.50 |
| 346: | 14 | 9 | 5 | 13 | 8 | 5 | 7893.8325 | -0.0042 | 0.025 | | |
| 347: | 14 | 9 | 6 | 13 | 8 | 6 | 7925.5667 | -0.0038 | 0.025 | | |
| 348: | 17 | 3 | 15 | 16 | 3 | 14 | 7975.4054 | 0.0022 | 0.025 | 0.0023 | 0.50 |
| 349: | 17 | 2 | 15 | 16 | 2 | 14 | 7975.4054 | 0.0022 | 0.025 | 0.0023 | 0.50 |
| 350: | 16 | 7 | 10 | 15 | 7 | 9 | 7976.2153 | 0.0076 | 0.025 | | |
| 351: | 16 | 6 | 10 | 15 | 6 | 9 | 8004.7972 | 0.0007 | 0.025 | | |
| 352: | 14 | 10 | 5 | 13 | 9 | 5 | 8032.6533 | 0.0010 | 0.025 | | |
| 353: | 16 | 10 | 7 | 15 | 10 | 6 | 8083.3055 | 0.0045 | 0.025 | | |
| 354: | 17 | 4 | 14 | 16 | 4 | 13 | 8087.8421 | 0.0104 | 0.025 | 0.0093 | 0.50 |
| 355: | 17 | 3 | 14 | 16 | 3 | 13 | 8087.8421 | 0.0081 | 0.025 | 0.0093 | 0.50 |
| 356: | 16 | 9 | 8 | 15 | 9 | 7 | 8094.9449 | 0.0066 | 0.025 | | |
| 357: | 16 | 10 | 6 | 15 | 10 | 5 | 8101.1607 | 0.0033 | 0.025 | | |
| 358: | 14 | 11 | 3 | 13 | 10 | 3 | 8144.7810 | 0.0056 | 0.025 | | |
| 359: | 14 | 11 | 4 | 13 | 10 | 4 | 8144.9950 | -0.0021 | 0.025 | | |
| 360: | 17 | 5 | 13 | 16 | 5 | 12 | 8201.2679 | 0.0382 | 0.025 | 0.0086 | 0.50 |
| 361: | 17 | 4 | 13 | 16 | 4 | 12 | 8201.2679 | -0.0210 | 0.025 | 0.0086 | 0.50 |
| 362: | 18 | 0 | 18 | 17 | 0 | 17 | 8204.3187 | 0.0133 | 0.025 | 0.0134 | 0.50 |
| 363: | 18 | 1 | 18 | 17 | 1 | 17 | 8204.3187 | 0.0133 | 0.025 | 0.0134 | 0.50 |
| 364: | 15 | 8 | 7 | 14 | 7 | 7 | 8233.6495 | 0.0115 | 0.025 | | |
| 365: | 15 | 7 | 8 | 14 | 6 | 8 | 8256.1508 | 0.0097 | 0.025 | | |
| 366: | 14 | 12 | 2 | 13 | 11 | 2 | 8256.2987 | 0.0118 | 0.025 | 0.0078 | 0.50 |
| 367: | 14 | 12 | 3 | 13 | 11 | 3 | 8256.2987 | 0.0037 | 0.025 | 0.0078 | 0.50 |
| 368: | 18 | 1 | 17 | 17 | 1 | 16 | 8316.0768 | 0.0086 | 0.025 | 0.0086 | 0.50 |
| 369: | 18 | 2 | 17 | 17 | 2 | 16 | 8316.0768 | 0.0086 | 0.025 | 0.0086 | 0.50 |
| 370: | 17 | 6 | 12 | 16 | 6 | 11 | 8316.3317 | -0.0003 | 0.025 | | |
| 371: | 17 | 5 | 12 | 16 | 5 | 11 | 8317.3512 | -0.0013 | 0.025 | | |
| 372: | 15 | 6 | 9 | 14 | 5 | 9 | 8349.0793 | 0.0083 | 0.025 | | |
| 373: | 15 | 9 | 6 | 14 | 8 | 6 | 8360.5109 | -0.0167 | 0.025 | | |
| 374: | 14 | 13 | 1 | 13 | 12 | 1 | 8366.7030 | 0.0045 | 0.025 | 0.0045 | 0.50 |
| 375: | 14 | 13 | 2 | 13 | 12 | 2 | 8366.7030 | 0.0044 | 0.025 | 0.0045 | 0.50 |
| 376: | 15 | 7 | 9 | 14 | 6 | 9 | 8380.5146 | 0.0048 | 0.025 | | |
| 377: | 15 | 8 | 8 | 14 | 7 | 8 | 8381.9391 | 0.0081 | 0.025 | | |
| 378: | 15 | 5 | 10 | 14 | 4 | 10 | 8397.9352 | 0.0066 | 0.025 | | |
| 379: | 15 | 6 | 10 | 14 | 5 | 10 | 8401.5421 | -0.0004 | 0.025 | | |
| 380: | 17 | 7 | 11 | 16 | 7 | 10 | 8431.9546 | -0.0054 | 0.025 | | |
| 381: | 15 | 9 | 7 | 14 | 8 | 7 | 8433.8642 | 0.0072 | 0.025 | | |
| 382: | 15 | 3 | 12 | 14 | 2 | 12 | 8437.7563 | 0.0096 | 0.025 | 0.0042 | 0.50 |
| 383: | 15 | 4 | 12 | 14 | 3 | 12 | 8437.7563 | -0.0012 | 0.025 | 0.0042 | 0.50 |
| 384: | 17 | 6 | 11 | 16 | 6 | 10 | 8443.2262 | 0.0040 | 0.025 | | |
| 385: | 15 | 2 | 13 | 14 | 1 | 13 | 8449.5441 | -0.0001 | 0.025 | -0.0003 | 0.50 |
| 386: | 15 | 3 | 13 | 14 | 2 | 13 | 8449.5441 | -0.0004 | 0.025 | -0.0003 | 0.50 |
| 387: | 15 | 10 | 5 | 14 | 9 | 5 | 8517.2550 | 0.0069 | 0.025 | | |
| 388: | 17 | 14 | 4 | 16 | 14 | 3 | 8520.3672 | 0.0067 | 0.025 | 0.0062 | 0.50 |
| 389: | 17 | 14 | 3 | 16 | 14 | 2 | 8520.3672 | 0.0056 | 0.025 | 0.0062 | 0.50 |
| 390: | 17 | 11 | 6 | 16 | 11 | 5 | 8586.7616 | -0.0425 | 0.025 | | |
| 391: | 17 | 9 | 9 | 16 | 9 | 8 | 8596.3881 | -0.0056 | 0.025 | | |
| 392: | 17 | 7 | 10 | 16 | 7 | 9 | 8602.8826 | 0.0095 | 0.025 | | |
| 393: | 15 | 11 | 4 | 14 | 10 | 4 | 8641.0991 | 0.0058 | 0.025 | | |
| 394: | 15 | 11 | 5 | 14 | 10 | 5 | 8642.2387 | 0.0013 | 0.025 | | |
| 395: | 17 | 10 | 7 | 16 | 10 | 6 | 8651.4667 | -0.0016 | 0.025 | | |
| 396: | 17 | 8 | 9 | 16 | 8 | 8 | 8754.7884 | 0.0047 | 0.025 | | |
| 397: | 17 | 9 | 8 | 16 | 9 | 7 | 8755.2959 | 0.0019 | 0.025 | | |
| 398: | 18 | 5 | 13 | 17 | 5 | 12 | 8768.3451 | 0.0151 | 0.025 | | |
| 399: | 19 | 1 | 18 | 18 | 1 | 17 | 8768.6891 | -0.0027 | 0.025 | -0.0027 | 0.50 |
| 400: | 19 | 2 | 18 | 18 | 2 | 17 | 8768.6891 | -0.0027 | 0.025 | -0.0027 | 0.50 |
| 401: | 16 | 9 | 7 | 15 | 8 | 7 | 8828.7923 | 0.0028 | 0.025 | | |
| 402: | 15 | 13 | 2 | 14 | 12 | 2 | 8865.7337 | 0.0055 | 0.025 | 0.0046 | 0.50 |
| 403: | 15 | 13 | 3 | 14 | 12 | 3 | 8865.7337 | 0.0036 | 0.025 | 0.0046 | 0.50 |
| 404: | 19 | 2 | 17 | 18 | 2 | 16 | 8880.5845 | -0.0042 | 0.025 | -0.0043 | 0.50 |
| 405: | 19 | 3 | 17 | 18 | 3 | 16 | 8880.5845 | -0.0042 | 0.025 | -0.0043 | 0.50 |
| 406: | 18 | 6 | 12 | 17 | 6 | 11 | 8888.5001 | 0.0048 | 0.025 | | |
| 407: | 16 | 7 | 10 | 15 | 6 | 10 | 8945.2231 | 0.0167 | 0.025 | | |
| 408: | 16 | 5 | 11 | 15 | 4 | 11 | 8967.7507 | -0.0024 | 0.025 | | |
| 409: | 16 | 6 | 11 | 15 | 5 | 11 | 8968.8906 | -0.0011 | 0.025 | | |
| 410: | 15 | 14 | 1 | 14 | 13 | 1 | 8976.0499 | -0.0010 | 0.025 | -0.0011 | 0.50 |
| 411: | 15 | 14 | 2 | 14 | 13 | 2 | 8976.0499 | -0.0010 | 0.025 | -0.0011 | 0.50 |
| 412: | 19 | 4 | 16 | 18 | 4 | 15 | 8992.8123 | 0.0006 | 0.025 | 0.0006 | 0.50 |
| 413: | 19 | 3 | 16 | 18 | 3 | 15 | 8992.8123 | 0.0004 | 0.025 | 0.0006 | 0.50 |
| 414: | 16 | 10 | 6 | 15 | 9 | 6 | 8993.4321 | 0.0004 | 0.025 | | |

```
 415: 16  3 13   15  2 13                          9002.9134   0.0010  0.025  -0.0003 0.50
 416: 16  4 13   15  3 13                          9002.9134  -0.0015  0.025  -0.0003 0.50
 417: 16  2 14   15  1 14                          9014.1955  -0.0055  0.025  -0.0056 0.50
 418: 16  3 14   15  2 14                          9014.1955  -0.0056  0.025  -0.0056 0.50
 419: 18 15  4   17 15  3                          9019.1110   0.0046  0.025   0.0045 0.50
 420: 18 15  3   17 15  2                          9019.1110   0.0043  0.025   0.0045 0.50
 421: 18  9 10   17  9  9                          9083.7951   0.0050  0.025
 422: 18 11  8   17 11  7                          9103.0441   0.0020  0.025
 423: 19  5 15   18  5 14                          9105.7295   0.0391  0.025   0.0372 0.50
 424: 19  4 15   18  4 14                          9105.7295   0.0353  0.025   0.0372 0.50
 425: 20  0 20   19  0 19                          9109.5754   0.0156  0.025   0.0157 0.50
 426: 20  1 20   19  1 19                          9109.5754   0.0156  0.025   0.0157 0.50
 427: 18 10  9   17 10  8                          9116.4310   0.0089  0.025
 428: 16 11  5   15 10  5                          9133.9706   0.0061  0.025
 429: 16 11  6   15 10  6                          9138.6815  -0.0029  0.025
 430: 20  1 19   19  1 18                          9221.3122   0.0048  0.025   0.0049 0.50
 431: 20  2 19   19  2 18                          9221.3122   0.0048  0.025   0.0049 0.50
 432: 18  9  9   17  9  8                          9295.1198  -0.0080  0.025
 433: 20  2 18   19  2 17                          9333.1832   0.0046  0.025   0.0047 0.50
 434: 20  3 18   19  3 17                          9333.1832   0.0046  0.025   0.0047 0.50
 435: 16 13  3   15 12  3                          9364.2475   0.0023  0.025  -0.0054 0.50
 436: 16 13  4   15 12  4                          9364.2475  -0.0132  0.025  -0.0054 0.50
 437: 16 14  2   15 13  2                          9475.1324   0.0067  0.025   0.0065 0.50
 438: 16 14  3   15 13  3                          9475.1324   0.0063  0.025   0.0065 0.50
 439: 17 10  8   16  9  8                          9538.9812   0.0070  0.025
 440: 20  5 16   19  5 15                          9558.0305   0.0031  0.025   0.0027 0.50
 441: 20  4 16   19  4 15                          9558.0305   0.0022  0.025   0.0027 0.50
 442: 21  0 21   20  0 20                          9562.1170  -0.0554  0.025  -0.0554 0.50
 443: 21  1 21   20  1 20                          9562.1170  -0.0554  0.025  -0.0554 0.50
 444: 16 15  1   15 14  1                          9585.3835  -0.0004  0.025  -0.0004 0.50
 445: 16 15  2   15 14  2                          9585.3835  -0.0004  0.025  -0.0004 0.50
 446: 17 11  6   16 10  6                          9619.6154   0.0042  0.025
 447: 16 16  0   15 15  0                          9695.3994   0.0009  0.025   0.0009 0.50
 448: 16 16  1   15 15  1                          9695.3994   0.0009  0.025   0.0009 0.50
 449: 21  2 19   20  2 18                          9785.7718   0.0083  0.025   0.0084 0.50
 450: 21  3 19   20  3 18                          9785.7718   0.0083  0.025   0.0084 0.50
 451: 17 16  1   16 15  1                         10194.6963  -0.0010  0.025  -0.0010 0.50
 452: 17 16  2   16 15  2                         10194.6963  -0.0010  0.025  -0.0010 0.50
 453: 17 17  0   16 16  0                         10304.7035  -0.0001  0.025  -0.0001 0.50
 454: 17 17  1   16 16  1                         10304.7035  -0.0001  0.025  -0.0001 0.50
 455: 23  0 23   22  0 22                         10467.3838   0.0177  0.025   0.0178 0.50
 456: 23  1 23   22  1 22                         10467.3838   0.0177  0.025   0.0178 0.50
 457: 23  2 21   22  2 20                         10690.8926  -0.0197  0.025  -0.0198 0.50
 458: 23  3 21   22  3 20                         10690.8926  -0.0197  0.025  -0.0198 0.50
 459: 18 16  2   17 15  2                         10693.8140  -0.0056  0.025  -0.0056 0.50
 460: 18 16  3   17 15  3                         10693.8140  -0.0056  0.025  -0.0056 0.50
 461: 19  3 16   18  2 16                         10697.6512  -0.0040  0.025  -0.0040 0.50
 462: 19  4 16   18  3 16                         10697.6512  -0.0040  0.025  -0.0040 0.50
--------------------------------------------------------------------------------

PARAMETERS IN FIT:

        10000           A  /MHz          304.7204(2)                         1
        20000           B  /MHz          266.1777(2)                         2
        30000           C  /MHz          226.3591(2)                         3
          200          DJ  /kHz            0.0207(4)                         4
         1100         DJK  /kHz            0.058(2)                          5
         2000          DK  /kHz           -0.037(2)                          6
        40100          d1  /kHz           -0.0017(2)                         7
        50000          d2  /kHz           -0.0017(1)                         8

 MICROWAVE AVG =        0.000085 MHz, IR AVG =        0.00000
 MICROWAVE RMS =        0.006228 MHz, IR RMS =        0.00000
 END OF ITERATION  1 OLD, NEW RMS ERROR=        0.24911         0.24911

  distinct frequency lines in fit:   385
       distinct parameters of fit:     8

 for standard errors previous errors are multiplied by:   0.251739
```

PARAMETERS IN FIT WITH STANDARD ERRORS ON THOSE THAT ARE FITTED:

| | | | | |
|---|---|---|---|---|
| 10000 | A | /MHz | 304.72043(7) | 1 |
| 20000 | B | /MHz | 266.17778(5) | 2 |
| 30000 | C | /MHz | 226.35912(5) | 3 |
| 200 | DJ | /kHz | 0.0207(1) | 4 |
| 1100 | DJK | /kHz | 0.0580(5) | 5 |
| 2000 | DK | /kHz | -0.0374(5) | 6 |
| 40100 | d1 | /kHz | -0.00175(5) | 7 |
| 50000 | d2 | /kHz | -0.00173(3) | 8 |

CORRELATION COEFFICIENTS, C.ij:

| | A | B | C | -DJ | -DJK | -DK | d1 | d2 |
|---|---|---|---|---|---|---|---|---|
| A | 1.0000 | | | | | | | |
| B | -0.1511 | 1.0000 | | | | | | |
| C | 0.0782 | 0.0941 | 1.0000 | | | | | |
| -DJ | 0.1991 | -0.6955 | -0.4348 | 1.0000 | | | | |
| -DJK | -0.4324 | 0.1924 | -0.0262 | -0.6331 | 1.0000 | | | |
| -DK | 0.0540 | 0.0233 | 0.1043 | 0.4072 | -0.8698 | 1.0000 | | |
| d1 | 0.2397 | -0.7212 | 0.3443 | 0.5480 | -0.4402 | 0.2657 | 1.0000 | |
| d2 | 0.1050 | -0.1430 | 0.1475 | -0.1581 | 0.2747 | -0.3326 | 0.1543 | 1.0000 |

Mean value of |C.ij|, i.ne.j = 0.2954
Mean value of C.ij, i.ne.j = -0.0645

Worst fitting lines (obs-calc/error):

| | | | | | | | |
|---|---|---|---|---|---|---|---|
| 442: | -2.2 | 390: | -1.7 | 423: | 1.5 | 268: | -1.1 |
| 457: | -0.8 | 455: | 0.7 | 373: | -0.7 | 407: | 0.7 |
| 293: | -0.6 | 425: | 0.6 | 398: | 0.6 | 32: | 0.6 |
| 182: | -0.6 | 326: | -0.5 | 362: | 0.5 | 116: | 0.5 |
| 175: | -0.5 | 208: | -0.5 | 265: | -0.5 | 364: | 0.5 |
| 319: | -0.4 | 296: | 0.4 | 365: | 0.4 | 392: | 0.4 |
| 354: | 0.4 | 427: | 0.4 | 43: | -0.4 | 368: | 0.3 |
| 307: | -0.3 | 360: | 0.3 | 449: | 0.3 | 372: | 0.3 |
| 343: | -0.3 | 99: | 0.3 | 207: | 0.3 | 377: | 0.3 |
| 272: | -0.3 | 432: | -0.3 | 344: | -0.3 | 366: | 0.3 |
| 314: | -0.3 | 350: | 0.3 | 181: | 0.3 | 309: | -0.3 |
| 34: | 0.3 | 263: | -0.3 | 381: | 0.3 | 439: | 0.3 |
| 387: | 0.3 | 356: | 0.3 | | | | |

/ SPFIT output reformatted with PIFORM

Heptamer - 7 II

| | | | | | | | obs | o-c | error | blends o-c | blends wt | Notes |
|---|---|---|---|---|---|---|---|---|---|---|---|---|
| 1: | 6 | 0 | 6 | 5 | 1 | 5 | 2018.8362 | 0.0004 | 0.025 | -0.0010 | 0.25 | |
| 2: | 6 | 1 | 6 | 5 | 0 | 5 | 2018.8362 | -0.0023 | 0.025 | -0.0010 | 0.25 | |
| 3: | 6 | 0 | 6 | 5 | 0 | 5 | 2018.8362 | -0.0021 | 0.025 | -0.0010 | 0.25 | |
| 4: | 6 | 1 | 6 | 5 | 1 | 5 | 2018.8362 | 0.0002 | 0.025 | -0.0010 | 0.25 | |
| 5: | 6 | 1 | 5 | 5 | 2 | 4 | 2135.7497 | 0.0017 | 0.025 | -0.0124 | 0.50 | |
| 6: | 6 | 2 | 5 | 5 | 2 | 4 | 2135.7497 | -0.0265 | 0.025 | -0.0124 | 0.50 | |
| 7: | 6 | 1 | 5 | 5 | 1 | 4 | 2136.0344 | 0.0212 | 0.025 | 0.0071 | 0.50 | |
| 8: | 6 | 2 | 5 | 5 | 1 | 4 | 2136.0344 | -0.0070 | 0.025 | 0.0071 | 0.50 | |
| 9: | 6 | 2 | 4 | 5 | 3 | 3 | 2249.2964 | 0.0146 | 0.025 | | | |
| 10: | 6 | 3 | 4 | 5 | 3 | 3 | 2250.5780 | 0.0000 | 0.025 | | | |
| 11: | 6 | 2 | 4 | 5 | 2 | 3 | 2257.4442 | -0.0045 | 0.025 | | | |
| 12: | 6 | 3 | 4 | 5 | 2 | 3 | 2258.7442 | -0.0008 | 0.025 | | | |
| 13: | 7 | 0 | 7 | 6 | 1 | 6 | 2345.5582 | -0.0011 | 0.025 | -0.0013 | 0.25 | |
| 14: | 7 | 1 | 7 | 6 | 0 | 6 | 2345.5582 | -0.0014 | 0.025 | -0.0013 | 0.25 | |
| 15: | 7 | 0 | 7 | 6 | 0 | 6 | 2345.5582 | -0.0014 | 0.025 | -0.0013 | 0.25 | |
| 16: | 7 | 1 | 7 | 6 | 1 | 6 | 2345.5582 | -0.0012 | 0.025 | -0.0013 | 0.25 | |
| 17: | 7 | 1 | 6 | 6 | 2 | 5 | 2462.5296 | -0.0042 | 0.025 | -0.0056 | 0.50 | |
| 18: | 7 | 2 | 6 | 6 | 2 | 5 | 2462.5296 | -0.0069 | 0.025 | -0.0056 | 0.50 | |
| 19: | 7 | 1 | 6 | 6 | 1 | 5 | 2462.5689 | 0.0068 | 0.025 | 0.0054 | 0.50 | |
| 20: | 7 | 2 | 6 | 6 | 1 | 5 | 2462.5689 | 0.0040 | 0.025 | 0.0054 | 0.50 | |
| 21: | 7 | 2 | 5 | 6 | 3 | 4 | 2579.3122 | -0.0012 | 0.025 | | | |
| 22: | 7 | 3 | 5 | 6 | 3 | 4 | 2579.5149 | 0.0345 | 0.025 | | | |
| 23: | 7 | 2 | 5 | 6 | 2 | 4 | 2580.6085 | -0.0011 | 0.025 | | | |
| 24: | 7 | 3 | 5 | 6 | 2 | 4 | 2580.7760 | -0.0006 | 0.025 | | | |
| 25: | 6 | 5 | 2 | 5 | 4 | 1 | 2595.9318 | 0.0034 | 0.025 | | | |
| 26: | 8 | 0 | 8 | 7 | 0 | 7 | 2672.2806 | -0.0013 | 0.025 | -0.0013 | 0.25 | |
| 27: | 8 | 1 | 8 | 7 | 1 | 7 | 2672.2806 | -0.0013 | 0.025 | -0.0013 | 0.25 | |
| 28: | 8 | 1 | 8 | 7 | 0 | 7 | 2672.2806 | -0.0013 | 0.025 | -0.0013 | 0.25 | |
| 29: | 8 | 0 | 8 | 7 | 1 | 7 | 2672.2806 | -0.0013 | 0.025 | -0.0013 | 0.25 | |
| 30/ | 7 | 3 | 4 | 6 | 4 | 3 | 2686.2940 | 0.1353 | 0.025 | | | |
| 31: | 7 | 4 | 4 | 6 | 3 | 3 | 2713.5181 | 0.0011 | 0.025 | | | |
| 32: | 6 | 6 | 1 | 5 | 5 | 0 | 2716.9813 | 0.0007 | 0.025 | | | |
| 33: | 6 | 6 | 0 | 5 | 5 | 1 | 2729.0612 | 0.0006 | 0.025 | | | |
| 34: | 8 | 2 | 6 | 7 | 3 | 5 | 2906.3732 | -0.0095 | 0.025 | | | |
| 35: | 8 | 2 | 6 | 7 | 2 | 5 | 2906.5760 | 0.0262 | 0.025 | 0.0168 | 0.50 | |
| 36: | 8 | 3 | 6 | 7 | 2 | 5 | 2906.5760 | 0.0073 | 0.025 | 0.0168 | 0.50 | |
| 37: | 9 | 0 | 9 | 8 | 0 | 8 | 2999.0018 | -0.0016 | 0.025 | -0.0016 | 0.25 | |
| 38: | 9 | 1 | 9 | 8 | 1 | 8 | 2999.0018 | -0.0016 | 0.025 | -0.0016 | 0.25 | |
| 39: | 9 | 1 | 9 | 8 | 0 | 8 | 2999.0018 | -0.0016 | 0.025 | -0.0016 | 0.25 | |
| 40: | 9 | 0 | 9 | 8 | 1 | 8 | 2999.0018 | -0.0016 | 0.025 | -0.0016 | 0.25 | |
| 41: | 8 | 3 | 5 | 7 | 4 | 4 | 3022.2839 | -0.0013 | 0.025 | | | |
| 42: | 8 | 4 | 5 | 7 | 4 | 4 | 3022.9894 | -0.0020 | 0.025 | | | |
| 43: | 8 | 3 | 5 | 7 | 3 | 4 | 3026.8604 | -0.0016 | 0.025 | | | |
| 44: | 8 | 4 | 5 | 7 | 3 | 4 | 3027.5674 | -0.0008 | 0.025 | | | |
| 45: | 8 | 4 | 4 | 7 | 5 | 3 | 3114.6685 | -0.0019 | 0.025 | | | |
| 46: | 9 | 1 | 8 | 8 | 2 | 7 | 3115.9399 | -0.0008 | 0.025 | -0.0010 | 0.25 | |
| 47: | 9 | 2 | 8 | 8 | 1 | 7 | 3115.9399 | -0.0011 | 0.025 | -0.0010 | 0.25 | |
| 48: | 9 | 1 | 8 | 8 | 1 | 7 | 3115.9399 | -0.0010 | 0.025 | -0.0010 | 0.25 | |
| 49: | 9 | 2 | 8 | 8 | 2 | 7 | 3115.9399 | -0.0008 | 0.025 | -0.0010 | 0.25 | |
| 50: | 8 | 5 | 4 | 7 | 5 | 3 | 3127.5988 | -0.0033 | 0.025 | | | |
| 51: | 8 | 4 | 4 | 7 | 4 | 3 | 3165.4011 | 0.0010 | 0.025 | | | |
| 52: | 7 | 7 | 1 | 6 | 6 | 0 | 3179.2110 | -0.0003 | 0.025 | | | |
| 53: | 7 | 7 | 0 | 6 | 6 | 1 | 3185.0026 | 0.0012 | 0.025 | | | |
| 54: | 9 | 2 | 7 | 8 | 3 | 6 | 3233.0541 | 0.0087 | 0.025 | -0.0017 | 0.25 | |
| 55: | 9 | 3 | 7 | 8 | 3 | 6 | 3233.0541 | 0.0068 | 0.025 | -0.0017 | 0.25 | |
| 56: | 9 | 2 | 7 | 8 | 2 | 6 | 3233.0541 | -0.0101 | 0.025 | -0.0017 | 0.25 | |
| 57: | 9 | 3 | 7 | 8 | 2 | 6 | 3233.0541 | -0.0120 | 0.025 | -0.0017 | 0.25 | |
| 58: | 8 | 5 | 3 | 7 | 5 | 2 | 3285.2962 | -0.0010 | 0.025 | | | |
| 59: | 10 | 0 | 10 | 9 | 1 | 9 | 3325.7223 | -0.0009 | 0.025 | -0.0010 | 0.25 | |
| 60: | 10 | 0 | 10 | 9 | 0 | 9 | 3325.7223 | -0.0009 | 0.025 | -0.0010 | 0.25 | |
| 61: | 10 | 1 | 10 | 9 | 1 | 9 | 3325.7223 | -0.0009 | 0.025 | -0.0010 | 0.25 | |
| 62: | 10 | 1 | 10 | 9 | 0 | 9 | 3325.7223 | -0.0009 | 0.025 | -0.0010 | 0.25 | |
| 63: | 9 | 3 | 6 | 8 | 4 | 5 | 3350.4100 | -0.0011 | 0.025 | | | |
| 64: | 9 | 4 | 6 | 8 | 4 | 5 | 3350.5018 | -0.0020 | 0.025 | | | |

| | | | | | | | | | | | |
|---|---|---|---|---|---|---|---|---|---|---|---|
| 65: | 9 | 3 | 6 | 8 | 3 | 5 | 3351.1148 | -0.0025 | 0.025 | | |
| 66: | 9 | 4 | 6 | 8 | 3 | 5 | 3351.2100 | 0.0000 | 0.025 | | |
| 67: | 8 | 6 | 3 | 7 | 5 | 2 | 3380.3109 | 0.0053 | 0.025 | | |
| 68: | 10 | 1 | 9 | 9 | 2 | 8 | 3442.6477 | -0.0009 | 0.025 | -0.0009 | 0.25 |
| 69: | 10 | 2 | 9 | 9 | 1 | 8 | 3442.6477 | -0.0009 | 0.025 | -0.0009 | 0.25 |
| 70: | 10 | 1 | 9 | 9 | 1 | 8 | 3442.6477 | -0.0009 | 0.025 | -0.0009 | 0.25 |
| 71: | 10 | 2 | 9 | 9 | 2 | 8 | 3442.6477 | -0.0009 | 0.025 | -0.0009 | 0.25 |
| 72: | 9 | 4 | 5 | 8 | 5 | 4 | 3463.3505 | -0.0011 | 0.025 | | |
| 73: | 9 | 5 | 5 | 8 | 5 | 4 | 3465.7351 | -0.0003 | 0.025 | | |
| 74: | 9 | 4 | 5 | 8 | 4 | 4 | 3476.2823 | -0.0010 | 0.025 | | |
| 75: | 9 | 5 | 5 | 8 | 4 | 4 | 3478.6669 | -0.0001 | 0.025 | | |
| 76: | 9 | 5 | 4 | 8 | 6 | 3 | 3529.2244 | -0.0016 | 0.025 | | |
| 77: | 8 | 7 | 2 | 7 | 6 | 1 | 3550.5384 | 0.0002 | 0.025 | | |
| 78: | 10 | 2 | 8 | 9 | 3 | 7 | 3559.6946 | 0.0000 | 0.025 | -0.0011 | 0.25 |
| 79: | 10 | 3 | 8 | 9 | 2 | 7 | 3559.6946 | -0.0021 | 0.025 | -0.0011 | 0.25 |
| 80: | 10 | 2 | 8 | 9 | 2 | 7 | 3559.6946 | -0.0020 | 0.025 | -0.0011 | 0.25 |
| 81: | 10 | 3 | 8 | 9 | 3 | 7 | 3559.6946 | -0.0002 | 0.025 | -0.0011 | 0.25 |
| 82: | 9 | 6 | 4 | 8 | 6 | 3 | 3559.8985 | -0.0016 | 0.025 | | |
| 83: | 8 | 7 | 1 | 7 | 6 | 2 | 3605.3621 | -0.0014 | 0.025 | | |
| 84: | 9 | 5 | 4 | 8 | 5 | 3 | 3624.2353 | 0.0009 | 0.025 | | |
| 85: | 8 | 8 | 1 | 7 | 7 | 0 | 3639.8759 | 0.0002 | 0.025 | | |
| 86: | 8 | 8 | 0 | 7 | 7 | 1 | 3642.5285 | 0.0011 | 0.025 | | |
| 87: | 11 | 0 | 11 | 10 | 0 | 10 | 3652.4393 | -0.0018 | 0.025 | -0.0019 | 0.25 |
| 88: | 11 | 1 | 11 | 10 | 1 | 10 | 3652.4393 | -0.0018 | 0.025 | -0.0019 | 0.25 |
| 89: | 11 | 1 | 11 | 10 | 0 | 10 | 3652.4393 | -0.0018 | 0.025 | -0.0019 | 0.25 |
| 90: | 11 | 0 | 11 | 10 | 1 | 10 | 3652.4393 | -0.0018 | 0.025 | -0.0019 | 0.25 |
| 91: | 9 | 6 | 4 | 8 | 5 | 3 | 3654.9031 | -0.0053 | 0.025 | | |
| 92: | 9 | 6 | 3 | 8 | 6 | 2 | 3713.6545 | -0.0005 | 0.025 | | |
| 93: | 11 | 1 | 10 | 10 | 1 | 9 | 3769.3567 | -0.0014 | 0.025 | -0.0015 | 0.25 |
| 94: | 11 | 2 | 10 | 10 | 2 | 9 | 3769.3567 | -0.0014 | 0.025 | -0.0015 | 0.25 |
| 95: | 11 | 2 | 10 | 10 | 1 | 9 | 3769.3567 | -0.0014 | 0.025 | -0.0015 | 0.25 |
| 96: | 11 | 1 | 10 | 10 | 2 | 9 | 3769.3567 | -0.0014 | 0.025 | -0.0015 | 0.25 |
| 97: | 10 | 4 | 6 | 9 | 5 | 5 | 3794.4448 | -0.0023 | 0.025 | | |
| 98: | 10 | 5 | 6 | 9 | 5 | 5 | 3794.8061 | -0.0025 | 0.025 | | |
| 99: | 10 | 4 | 6 | 9 | 4 | 5 | 3796.8296 | -0.0013 | 0.025 | | |
| 100: | 10 | 5 | 6 | 9 | 4 | 5 | 3797.1907 | -0.0016 | 0.025 | | |
| 101: | 9 | 7 | 3 | 8 | 6 | 2 | 3869.3975 | 0.0012 | 0.025 | | |
| 102: | 11 | 2 | 9 | 10 | 3 | 8 | 3886.3620 | -0.0013 | 0.025 | -0.0015 | 0.25 |
| 103: | 11 | 3 | 9 | 10 | 2 | 8 | 3886.3620 | -0.0015 | 0.025 | -0.0015 | 0.25 |
| 104: | 11 | 2 | 9 | 10 | 2 | 8 | 3886.3620 | -0.0015 | 0.025 | -0.0015 | 0.25 |
| 105: | 11 | 3 | 9 | 10 | 3 | 8 | 3886.3620 | -0.0013 | 0.025 | -0.0015 | 0.25 |
| 106: | 10 | 5 | 5 | 9 | 6 | 4 | 3899.9601 | -0.0009 | 0.025 | | |
| 107: | 10 | 6 | 5 | 9 | 6 | 4 | 3906.7479 | -0.0019 | 0.025 | | |
| 108: | 10 | 5 | 5 | 9 | 5 | 4 | 3930.6333 | -0.0018 | 0.025 | | |
| 109: | 10 | 6 | 5 | 9 | 5 | 4 | 3937.4257 | 0.0017 | 0.025 | | |
| 110: | 12 | 0 | 12 | 11 | 0 | 11 | 3979.1543 | -0.0025 | 0.025 | -0.0025 | 0.25 |
| 111: | 12 | 1 | 12 | 11 | 1 | 11 | 3979.1543 | -0.0025 | 0.025 | -0.0025 | 0.25 |
| 112: | 12 | 1 | 12 | 11 | 0 | 11 | 3979.1543 | -0.0025 | 0.025 | -0.0025 | 0.25 |
| 113: | 12 | 0 | 12 | 11 | 1 | 11 | 3979.1543 | -0.0025 | 0.025 | -0.0025 | 0.25 |
| 114: | 9 | 8 | 2 | 8 | 7 | 1 | 4021.7862 | 0.0021 | 0.025 | | |
| 115: | 9 | 8 | 1 | 8 | 7 | 2 | 4051.9875 | 0.0006 | 0.025 | | |
| 116: | 10 | 6 | 4 | 9 | 6 | 3 | 4079.7353 | 0.0001 | 0.025 | | |
| 117: | 9 | 7 | 2 | 8 | 6 | 3 | 4089.1366 | -0.0029 | 0.025 | | |
| 118: | 12 | 1 | 11 | 11 | 1 | 10 | 4096.0659 | -0.0019 | 0.025 | -0.0019 | 0.25 |
| 119: | 12 | 2 | 11 | 11 | 2 | 10 | 4096.0659 | -0.0019 | 0.025 | -0.0019 | 0.25 |
| 120: | 12 | 2 | 11 | 11 | 1 | 10 | 4096.0659 | -0.0019 | 0.025 | -0.0019 | 0.25 |
| 121: | 12 | 1 | 11 | 11 | 2 | 10 | 4096.0659 | -0.0019 | 0.025 | -0.0019 | 0.25 |
| 122: | 9 | 9 | 1 | 8 | 8 | 0 | 4099.6879 | 0.0007 | 0.025 | | |
| 123: | 9 | 9 | 0 | 8 | 8 | 1 | 4100.8643 | 0.0019 | 0.025 | | |
| 124: | 11 | 4 | 7 | 10 | 5 | 6 | 4121.4174 | 0.0009 | 0.025 | | |
| 125: | 11 | 5 | 7 | 10 | 5 | 6 | 4121.4590 | -0.0055 | 0.025 | | |
| 126: | 11 | 4 | 7 | 10 | 4 | 6 | 4121.7742 | -0.0037 | 0.025 | | |
| 127: | 11 | 5 | 7 | 10 | 4 | 6 | 4121.8195 | -0.0064 | 0.025 | | |
| 128: | 10 | 7 | 3 | 9 | 7 | 2 | 4127.8863 | -0.0020 | 0.025 | | |
| 129: | 10 | 7 | 4 | 9 | 6 | 3 | 4142.3695 | 0.0015 | 0.025 | | |
| 130: | 12 | 2 | 10 | 11 | 2 | 9 | 4213.0443 | -0.0009 | 0.025 | -0.0009 | 0.25 |
| 131: | 12 | 3 | 10 | 11 | 3 | 9 | 4213.0443 | -0.0009 | 0.025 | -0.0009 | 0.25 |
| 132: | 12 | 3 | 10 | 11 | 2 | 9 | 4213.0443 | -0.0009 | 0.025 | -0.0009 | 0.25 |
| 133: | 12 | 2 | 10 | 11 | 3 | 9 | 4213.0443 | -0.0009 | 0.025 | -0.0009 | 0.25 |
| 134: | 11 | 5 | 6 | 10 | 6 | 5 | 4237.8771 | -0.0037 | 0.025 | | |

| | | | | | | | | | | |
|---|---|---|---|---|---|---|---|---|---|---|
| 135: | 11 | 6 | 6 | 10 | 6 | 5 | 4239.0626 | -0.0044 | 0.025 | | |
| 136: | 11 | 5 | 6 | 10 | 5 | 5 | 4244.6693 | -0.0003 | 0.025 | | |
| 137: | 11 | 6 | 6 | 10 | 5 | 5 | 4245.8559 | 0.0000 | 0.025 | | |
| 138: | 9 | 6 | 3 | 8 | 5 | 4 | 4265.2762 | -0.0070 | 0.025 | | |
| 139: | 13 | 0 | 13 | 12 | 0 | 12 | 4305.8670 | -0.0029 | 0.025 | -0.0029 | 0.25 |
| 140: | 13 | 1 | 13 | 12 | 1 | 12 | 4305.8670 | -0.0029 | 0.025 | -0.0029 | 0.25 |
| 141: | 13 | 1 | 13 | 12 | 0 | 12 | 4305.8670 | -0.0029 | 0.025 | -0.0029 | 0.25 |
| 142: | 13 | 0 | 13 | 12 | 1 | 12 | 4305.8670 | -0.0029 | 0.025 | -0.0029 | 0.25 |
| 143: | 12 | 3 | 9 | 11 | 4 | 8 | 4330.2061 | -0.0007 | 0.025 | -0.0014 | 0.25 |
| 144: | 12 | 4 | 9 | 11 | 3 | 8 | 4330.2061 | -0.0020 | 0.025 | -0.0014 | 0.25 |
| 145: | 12 | 3 | 9 | 11 | 3 | 8 | 4330.2061 | -0.0018 | 0.025 | -0.0014 | 0.25 |
| 146: | 12 | 4 | 9 | 11 | 4 | 8 | 4330.2061 | -0.0008 | 0.025 | -0.0014 | 0.25 |
| 147: | 11 | 7 | 5 | 10 | 7 | 4 | 4344.7836 | -0.0016 | 0.025 | | |
| 148: | 10 | 8 | 3 | 9 | 7 | 2 | 4358.6185 | -0.0007 | 0.025 | | |
| 149: | 11 | 6 | 5 | 10 | 6 | 4 | 4390.6044 | -0.0005 | 0.025 | | |
| 150: | 11 | 8 | 4 | 10 | 8 | 3 | 4407.2382 | -0.0008 | 0.025 | | |
| 151: | 11 | 7 | 5 | 10 | 6 | 4 | 4407.4192 | 0.0011 | 0.025 | | |
| 152: | 13 | 1 | 12 | 12 | 1 | 11 | 4422.7739 | -0.0023 | 0.025 | -0.0023 | 0.25 |
| 153: | 13 | 2 | 12 | 12 | 2 | 11 | 4422.7739 | -0.0023 | 0.025 | -0.0023 | 0.25 |
| 154: | 13 | 2 | 12 | 12 | 1 | 11 | 4422.7739 | -0.0023 | 0.025 | -0.0023 | 0.25 |
| 155: | 13 | 1 | 12 | 12 | 2 | 11 | 4422.7739 | -0.0023 | 0.025 | -0.0023 | 0.25 |
| 156: | 12 | 4 | 8 | 11 | 5 | 7 | 4447.8435 | 0.0295 | 0.025 | 0.0027 | 0.25 |
| 157: | 12 | 5 | 8 | 11 | 5 | 7 | 4447.8435 | 0.0237 | 0.025 | 0.0027 | 0.25 |
| 158: | 12 | 4 | 8 | 11 | 4 | 7 | 4447.8435 | -0.0184 | 0.025 | 0.0027 | 0.25 |
| 159: | 12 | 5 | 8 | 11 | 4 | 7 | 4447.8435 | -0.0242 | 0.025 | 0.0027 | 0.25 |
| 160: | 10 | 9 | 2 | 9 | 8 | 1 | 4488.3920 | 0.0001 | 0.025 | | |
| 161: | 10 | 9 | 1 | 9 | 8 | 2 | 4504.0005 | 0.0007 | 0.025 | | |
| 162: | 10 | 8 | 2 | 9 | 7 | 3 | 4507.6269 | 0.0094 | 0.025 | | |
| 163: | 11 | 7 | 4 | 10 | 7 | 3 | 4525.8409 | 0.0005 | 0.025 | | |
| 164: | 11 | 8 | 3 | 10 | 8 | 2 | 4527.7085 | -0.0022 | 0.025 | | |
| 165: | 13 | 2 | 11 | 12 | 2 | 10 | 4539.7321 | -0.0019 | 0.025 | -0.0019 | 0.25 |
| 166: | 13 | 2 | 11 | 12 | 3 | 10 | 4539.7321 | -0.0019 | 0.025 | -0.0019 | 0.25 |
| 167: | 13 | 3 | 11 | 12 | 2 | 10 | 4539.7321 | -0.0019 | 0.025 | -0.0019 | 0.25 |
| 168: | 13 | 3 | 11 | 12 | 3 | 10 | 4539.7321 | -0.0019 | 0.025 | -0.0019 | 0.25 |
| 169: | 10 | 10 | 1 | 9 | 9 | 0 | 4559.0746 | 0.0001 | 0.025 | | |
| 170: | 10 | 10 | 0 | 9 | 9 | 1 | 4559.5832 | 0.0006 | 0.025 | | |
| 171: | 12 | 5 | 7 | 11 | 6 | 6 | 4566.0427 | -0.0014 | 0.025 | | |
| 172: | 12 | 6 | 7 | 11 | 6 | 6 | 4566.2180 | -0.0038 | 0.025 | | |
| 173: | 12 | 5 | 7 | 11 | 5 | 6 | 4567.2301 | -0.0002 | 0.025 | | |
| 174: | 12 | 6 | 7 | 11 | 5 | 6 | 4567.4066 | -0.0014 | 0.025 | | |
| 175: | 14 | 0 | 14 | 13 | 0 | 13 | 4632.5781 | -0.0021 | 0.025 | -0.0022 | 0.25 |
| 176: | 14 | 1 | 14 | 13 | 1 | 13 | 4632.5781 | -0.0021 | 0.025 | -0.0022 | 0.25 |
| 177: | 14 | 1 | 14 | 13 | 0 | 13 | 4632.5781 | -0.0021 | 0.025 | -0.0022 | 0.25 |
| 178: | 14 | 0 | 14 | 13 | 1 | 13 | 4632.5781 | -0.0021 | 0.025 | -0.0022 | 0.25 |
| 179: | 13 | 3 | 10 | 12 | 4 | 9 | 4656.8276 | -0.0016 | 0.025 | -0.0017 | 0.25 |
| 180: | 13 | 4 | 10 | 12 | 4 | 9 | 4656.8276 | -0.0016 | 0.025 | -0.0017 | 0.25 |
| 181: | 13 | 3 | 10 | 12 | 3 | 9 | 4656.8276 | -0.0017 | 0.025 | -0.0017 | 0.25 |
| 182: | 13 | 4 | 10 | 12 | 3 | 9 | 4656.8276 | -0.0017 | 0.025 | -0.0017 | 0.25 |
| 183: | 12 | 7 | 6 | 11 | 7 | 5 | 4682.7167 | 0.0051 | 0.025 | | |
| 184: | 12 | 6 | 6 | 11 | 6 | 5 | 4696.1133 | -0.0172 | 0.025 | | |
| 185: | 12 | 7 | 6 | 11 | 6 | 5 | 4699.5233 | -0.0013 | 0.025 | | |
| 186: | 13 | 4 | 9 | 12 | 5 | 8 | 4774.2509 | 0.0018 | 0.025 | -0.0014 | 0.25 |
| 187: | 13 | 5 | 9 | 12 | 5 | 8 | 4774.2509 | 0.0011 | 0.025 | -0.0014 | 0.25 |
| 188: | 13 | 4 | 9 | 12 | 4 | 8 | 4774.2509 | -0.0040 | 0.025 | -0.0014 | 0.25 |
| 189: | 13 | 5 | 9 | 12 | 4 | 8 | 4774.2509 | -0.0046 | 0.025 | -0.0014 | 0.25 |
| 190: | 12 | 8 | 5 | 11 | 8 | 4 | 4778.5272 | -0.0019 | 0.025 | | |
| 191: | 11 | 9 | 3 | 10 | 8 | 2 | 4843.8739 | -0.0120 | 0.025 | | |
| 192: | 14 | 2 | 12 | 13 | 2 | 11 | 4866.4245 | -0.0016 | 0.025 | -0.0016 | 0.25 |
| 193: | 14 | 3 | 12 | 13 | 3 | 11 | 4866.4245 | -0.0016 | 0.025 | -0.0016 | 0.25 |
| 194: | 14 | 3 | 12 | 13 | 2 | 11 | 4866.4245 | -0.0016 | 0.025 | -0.0016 | 0.25 |
| 195: | 14 | 2 | 12 | 13 | 3 | 11 | 4866.4245 | -0.0016 | 0.025 | -0.0016 | 0.25 |
| 196: | 11 | 9 | 2 | 10 | 8 | 3 | 4936.8967 | -0.0015 | 0.025 | | |
| 197: | 10 | 6 | 4 | 9 | 5 | 5 | 4879.2797 | -0.0032 | 0.025 | | |
| 198: | 11 | 10 | 2 | 10 | 9 | 1 | 4951.6522 | -0.0013 | 0.025 | | |
| 199: | 15 | 0 | 15 | 14 | 0 | 14 | 4959.2858 | -0.0016 | 0.025 | -0.0017 | 0.25 |
| 200: | 15 | 1 | 15 | 14 | 1 | 14 | 4959.2858 | -0.0016 | 0.025 | -0.0017 | 0.25 |
| 201: | 15 | 1 | 15 | 14 | 0 | 14 | 4959.2858 | -0.0016 | 0.025 | -0.0017 | 0.25 |
| 202: | 15 | 0 | 15 | 14 | 1 | 14 | 4959.2858 | -0.0016 | 0.025 | -0.0017 | 0.25 |
| 203: | 11 | 10 | 1 | 10 | 9 | 2 | 4959.3435 | 0.0008 | 0.025 | | |
| 204: | 14 | 3 | 11 | 13 | 3 | 10 | 4983.4743 | -0.0009 | 0.025 | -0.0009 | 0.25 |

```
205: 14  4 11   13  4 10        4983.4743  -0.0009  0.025  -0.0009 0.25
206: 14  4 11   13  3 10        4983.4743  -0.0009  0.025  -0.0009 0.25
207: 14  3 11   13  4 10        4983.4743  -0.0009  0.025  -0.0009 0.25
208: 13  6  7   12  7  6        5010.7332  -0.0001  0.025
209: 13  7  7   12  7  6        5011.3084   0.0028  0.025
210: 13  6  7   12  6  6        5014.1241  -0.0033  0.025
211: 13  7  7   12  6  6        5014.6958  -0.0039  0.025
212: 11 11  1   10 10  0        5018.2620   0.0022  0.025
213: 15  1 14   14  1 13        5076.1849  -0.0019  0.025  -0.0019 0.25
214: 15  2 14   14  2 13        5076.1849  -0.0019  0.025  -0.0019 0.25
215: 15  2 14   14  1 13        5076.1849  -0.0019  0.025  -0.0019 0.25
216: 15  1 14   14  2 13        5076.1849  -0.0019  0.025  -0.0019 0.25
217: 14  4 10   13  5  9        5100.7627  -0.0009  0.025  -0.0013 0.25
218: 14  5 10   13  4  9        5100.7627  -0.0016  0.025  -0.0013 0.25
219: 14  4 10   13  4  9        5100.7627  -0.0015  0.025  -0.0013 0.25
220: 14  5 10   13  5  9        5100.7627  -0.0010  0.025  -0.0013 0.25
221: 13  7  6   12  8  5        5116.1645   0.0012  0.025
222: 13  8  6   12  8  5        5124.8082  -0.0037  0.025
223: 12  9  4   11  8  3        5137.9158   0.0032  0.025
224: 13  8  6   12  7  5        5161.6046   0.0037  0.025
225: 15  2 13   14  2 12        5193.1180  -0.0011  0.025  -0.0011 0.25
226: 15  3 13   14  3 12        5193.1180  -0.0011  0.025  -0.0011 0.25
227: 15  3 13   14  2 12        5193.1180  -0.0011  0.025  -0.0011 0.25
228: 15  2 13   14  3 12        5193.1180  -0.0011  0.025  -0.0011 0.25
229: 13  9  5   12  9  4        5206.8664  -0.0020  0.025
230: 16  0 16   15  0 15        5285.9895  -0.0020  0.025  -0.0020 0.25
231: 16  1 16   15  1 15        5285.9895  -0.0020  0.025  -0.0020 0.25
232: 16  1 16   15  0 15        5285.9895  -0.0020  0.025  -0.0020 0.25
233: 16  0 16   15  1 15        5285.9895  -0.0020  0.025  -0.0020 0.25
234: 15  3 12   14  3 11        5310.1344  -0.0005  0.025  -0.0005 0.25
235: 15  4 12   14  4 11        5310.1344  -0.0005  0.025  -0.0005 0.25
236: 15  4 12   14  3 11        5310.1344  -0.0005  0.025  -0.0005 0.25
237: 15  3 12   14  4 11        5310.1344  -0.0005  0.025  -0.0005 0.25
238: 13  8  5   12  8  4        5314.7081   0.0020  0.025
239: 12 10  3   11  9  2        5322.5882   0.0018  0.025
240: 14  6  8   13  7  7        5337.3122   0.0010  0.025
241: 14  7  8   13  7  7        5337.4066   0.0104  0.025
242: 14  6  8   13  6  7        5337.8857   0.0022  0.025
243: 14  7  8   13  6  7        5337.9672  -0.0012  0.025
244: 12 10  2   11  9  3        5376.4347  -0.0005  0.025
245: 16  1 15   15  1 14        5402.8873  -0.0006  0.025  -0.0007 0.25
246: 16  2 15   15  2 14        5402.8873  -0.0006  0.025  -0.0007 0.25
247: 16  2 15   15  1 14        5402.8873  -0.0006  0.025  -0.0007 0.25
248: 16  1 15   15  2 14        5402.8873  -0.0006  0.025  -0.0007 0.25
249: 12 11  2   11 10  1        5412.8918   0.0003  0.025
250: 12 11  1   11 10  2        5416.5459   0.0007  0.025
251: 15  4 11   14  4 10        5427.3330   0.0003  0.025   0.0004 0.25
252: 15  5 11   14  4 10        5427.3330   0.0003  0.025   0.0004 0.25
253: 15  5 11   14  5 10        5427.3330   0.0004  0.025   0.0004 0.25
254: 15  4 11   14  5 10        5427.3330   0.0004  0.025   0.0004 0.25
255: 12  9  3   11  8  4        5445.2436   0.0012  0.025
256: 14  8  7   13  8  6        5456.4518  -0.0035  0.025
257: 14  7  7   13  7  6        5463.4606  -0.0010  0.025
258: 14  8  7   13  7  6        5465.1035  -0.0004  0.025
259: 11  6  5   10  5  6        5475.0801   0.0008  0.025
260: 12 12  1   11 11  0        5477.3521   0.0021  0.025
261: 12 12  0   11 11  1        5477.4423   0.0021  0.025
262: 16  2 14   15  2 13        5519.8113  -0.0001  0.025  -0.0002 0.25
263: 16  3 14   15  3 13        5519.8113  -0.0001  0.025  -0.0002 0.25
264: 16  3 14   15  2 13        5519.8113  -0.0001  0.025  -0.0002 0.25
265: 16  2 14   15  3 13        5519.8113  -0.0001  0.025  -0.0002 0.25
266: 15  5 10   14  6  9        5544.9123   0.0019  0.025   0.0003 0.25
267: 15  6 10   14  6  9        5544.9123   0.0015  0.025   0.0003 0.25
268: 15  5 10   14  5  9        5544.9123  -0.0010  0.025   0.0003 0.25
269: 15  6 10   14  5  9        5544.9123  -0.0013  0.025   0.0003 0.25
270: 14  9  6   13  9  5        5564.1433  -0.0022  0.025
271: 14  8  6   13  8  5        5615.9652   0.0043  0.025
272: 16  3 13   15  3 12        5636.8024   0.0001  0.025   0.0001 0.25
273: 16  4 13   15  4 12        5636.8024   0.0001  0.025   0.0001 0.25
274: 16  4 13   15  3 12        5636.8024   0.0001  0.025   0.0001 0.25
```

| | | | | | | | | | | | | |
|---|---|---|---|---|---|---|---|---|---|---|---|---|
| 275: | 16 | 3 | 13 | 15 | 4 | 12 | 5636.8024 | 0.0001 | 0.025 | 0.0001 | 0.25 |
| 276: | 17 | 1 | 16 | 16 | 1 | 15 | 5729.5842 | -0.0017 | 0.025 | -0.0018 | 0.25 |
| 277: | 17 | 2 | 16 | 16 | 2 | 15 | 5729.5842 | -0.0017 | 0.025 | -0.0018 | 0.25 |
| 278: | 17 | 2 | 16 | 16 | 1 | 15 | 5729.5842 | -0.0017 | 0.025 | -0.0018 | 0.25 |
| 279: | 17 | 1 | 16 | 16 | 2 | 15 | 5729.5842 | -0.0017 | 0.025 | -0.0018 | 0.25 |
| 280: | 16 | 4 | 12 | 15 | 4 | 11 | 5753.9347 | -0.0004 | 0.025 | -0.0005 | 0.25 |
| 281: | 16 | 5 | 12 | 15 | 5 | 11 | 5753.9347 | -0.0004 | 0.025 | -0.0005 | 0.25 |
| 282: | 16 | 5 | 12 | 15 | 4 | 11 | 5753.9347 | -0.0004 | 0.025 | -0.0005 | 0.25 |
| 283: | 16 | 4 | 12 | 15 | 5 | 11 | 5753.9347 | -0.0004 | 0.025 | -0.0005 | 0.25 |
| 284: | 15 | 7 | 8 | 14 | 8 | 7 | 5782.6347 | 0.0020 | 0.025 | | |
| 285: | 15 | 8 | 8 | 14 | 8 | 7 | 5782.8993 | -0.0032 | 0.025 | | |
| 286: | 15 | 7 | 8 | 14 | 7 | 7 | 5784.2711 | -0.0037 | 0.025 | | |
| 287: | 15 | 8 | 8 | 14 | 7 | 7 | 5784.5449 | 0.0000 | 0.025 | | |
| 288: | 13 | 11 | 3 | 12 | 10 | 2 | 5794.5464 | 0.0004 | 0.025 | | |
| 289: | 13 | 11 | 2 | 12 | 10 | 3 | 5823.8486 | 0.0020 | 0.025 | | |
| 290: | 17 | 2 | 15 | 16 | 2 | 14 | 5846.5022 | 0.0000 | 0.025 | -0.0001 | 0.25 |
| 291: | 17 | 3 | 15 | 16 | 3 | 14 | 5846.5022 | 0.0000 | 0.025 | -0.0001 | 0.25 |
| 292: | 17 | 3 | 15 | 16 | 2 | 14 | 5846.5022 | 0.0000 | 0.025 | -0.0001 | 0.25 |
| 293: | 17 | 2 | 15 | 16 | 3 | 14 | 5846.5022 | 0.0000 | 0.025 | -0.0001 | 0.25 |
| 294: | 13 | 10 | 3 | 12 | 9 | 4 | 5851.5865 | 0.0023 | 0.025 | | |
| 295: | 16 | 5 | 11 | 15 | 6 | 10 | 5871.3545 | 0.0008 | 0.025 | 0.0006 | 0.25 |
| 296: | 16 | 6 | 11 | 15 | 5 | 10 | 5871.3545 | 0.0004 | 0.025 | 0.0006 | 0.25 |
| 297: | 16 | 5 | 11 | 15 | 5 | 10 | 5871.3545 | 0.0004 | 0.025 | 0.0006 | 0.25 |
| 298: | 16 | 6 | 11 | 15 | 6 | 10 | 5871.3545 | 0.0007 | 0.025 | 0.0006 | 0.25 |
| 299: | 13 | 12 | 1 | 12 | 11 | 2 | 5874.7146 | 0.0030 | 0.025 | | |
| 300: | 18 | 0 | 18 | 17 | 0 | 17 | 5939.3873 | -0.0013 | 0.025 | -0.0014 | 0.25 |
| 301: | 18 | 1 | 18 | 17 | 1 | 17 | 5939.3873 | -0.0013 | 0.025 | -0.0014 | 0.25 |
| 302: | 18 | 1 | 18 | 17 | 0 | 17 | 5939.3873 | -0.0013 | 0.025 | -0.0014 | 0.25 |
| 303: | 18 | 0 | 18 | 17 | 1 | 17 | 5939.3873 | -0.0013 | 0.025 | -0.0014 | 0.25 |
| 304: | 17 | 3 | 14 | 16 | 3 | 13 | 5963.4738 | 0.0001 | 0.025 | 0.0002 | 0.25 |
| 305: | 17 | 4 | 14 | 16 | 4 | 13 | 5963.4738 | 0.0001 | 0.025 | 0.0002 | 0.25 |
| 306: | 17 | 4 | 14 | 16 | 3 | 13 | 5963.4738 | 0.0001 | 0.025 | 0.0002 | 0.25 |
| 307: | 17 | 3 | 14 | 16 | 4 | 13 | 5963.4738 | 0.0001 | 0.025 | 0.0002 | 0.25 |
| 308: | 16 | 6 | 10 | 15 | 7 | 9 | 5989.3475 | 0.0064 | 0.025 | 0.0000 | 0.25 |
| 309: | 16 | 7 | 10 | 15 | 7 | 9 | 5989.3475 | 0.0050 | 0.025 | 0.0000 | 0.25 |
| 310: | 16 | 6 | 10 | 15 | 6 | 9 | 5989.3475 | -0.0050 | 0.025 | 0.0000 | 0.25 |
| 311: | 16 | 7 | 10 | 15 | 6 | 9 | 5989.3475 | -0.0064 | 0.025 | 0.0000 | 0.25 |
| 312: | 18 | 1 | 17 | 17 | 1 | 16 | 6056.2793 | -0.0010 | 0.025 | -0.0011 | 0.25 |
| 313: | 18 | 1 | 17 | 17 | 2 | 16 | 6056.2793 | -0.0010 | 0.025 | -0.0011 | 0.25 |
| 314: | 18 | 2 | 17 | 17 | 1 | 16 | 6056.2793 | -0.0010 | 0.025 | -0.0011 | 0.25 |
| 315: | 18 | 2 | 17 | 17 | 2 | 16 | 6056.2793 | -0.0010 | 0.025 | -0.0011 | 0.25 |
| 316: | 17 | 4 | 13 | 16 | 4 | 12 | 6080.5595 | 0.0007 | 0.025 | 0.0008 | 0.25 |
| 317: | 17 | 4 | 13 | 16 | 5 | 12 | 6080.5595 | 0.0007 | 0.025 | 0.0008 | 0.25 |
| 318: | 17 | 5 | 13 | 16 | 4 | 12 | 6080.5595 | 0.0007 | 0.025 | 0.0008 | 0.25 |
| 319: | 17 | 5 | 13 | 16 | 5 | 12 | 6080.5595 | 0.0007 | 0.025 | 0.0008 | 0.25 |
| 320: | 15 | 9 | 6 | 14 | 9 | 5 | 6083.1945 | 0.0043 | 0.025 | | |
| 321: | 17 | 5 | 12 | 16 | 5 | 11 | 6197.8670 | 0.0024 | 0.025 | 0.0024 | 0.25 |
| 322: | 17 | 5 | 12 | 16 | 6 | 11 | 6197.8670 | 0.0024 | 0.025 | 0.0024 | 0.25 |
| 323: | 17 | 6 | 12 | 16 | 6 | 11 | 6197.8670 | 0.0024 | 0.025 | 0.0024 | 0.25 |
| 324: | 17 | 6 | 12 | 16 | 5 | 11 | 6197.8670 | 0.0024 | 0.025 | 0.0024 | 0.25 |
| 325: | 16 | 8 | 8 | 15 | 9 | 7 | 6228.0575 | 0.0027 | 0.025 | | |
| 326: | 16 | 9 | 8 | 15 | 9 | 7 | 6228.7838 | -0.0472 | 0.025 | | |
| 327: | 16 | 8 | 8 | 15 | 8 | 7 | 6232.2890 | -0.0335 | 0.025 | | |
| 328: | 16 | 9 | 8 | 15 | 8 | 7 | 6233.0977 | -0.0009 | 0.025 | | |
| 329: | 14 | 12 | 3 | 13 | 11 | 2 | 6261.2656 | 0.0011 | 0.025 | | |
| 330: | 19 | 0 | 19 | 18 | 0 | 18 | 6266.0807 | -0.0006 | 0.025 | -0.0007 | 0.25 |
| 331: | 19 | 1 | 19 | 18 | 1 | 18 | 6266.0807 | -0.0006 | 0.025 | -0.0007 | 0.25 |
| 332: | 19 | 1 | 19 | 18 | 0 | 18 | 6266.0807 | -0.0006 | 0.025 | -0.0007 | 0.25 |
| 333: | 19 | 0 | 19 | 18 | 1 | 18 | 6266.0807 | -0.0006 | 0.025 | -0.0007 | 0.25 |
| 334: | 14 | 11 | 3 | 13 | 10 | 4 | 6270.6680 | -0.0026 | 0.025 | | |
| 335: | 14 | 12 | 2 | 13 | 11 | 3 | 6276.4587 | 0.0023 | 0.025 | | |
| 336: | 18 | 3 | 15 | 17 | 3 | 14 | 6290.1469 | 0.0003 | 0.025 | 0.0003 | 0.25 |
| 337: | 18 | 4 | 15 | 17 | 4 | 14 | 6290.1469 | 0.0003 | 0.025 | 0.0003 | 0.25 |
| 338: | 18 | 4 | 15 | 17 | 3 | 14 | 6290.1469 | 0.0003 | 0.025 | 0.0003 | 0.25 |
| 339: | 18 | 3 | 15 | 17 | 4 | 14 | 6290.1469 | 0.0003 | 0.025 | 0.0003 | 0.25 |
| 340: | 14 | 13 | 2 | 13 | 12 | 1 | 6332.5865 | 0.0016 | 0.025 | | |
| 341: | 14 | 13 | 1 | 13 | 12 | 2 | 6333.3511 | 0.0037 | 0.025 | | |
| 342: | 19 | 1 | 18 | 18 | 1 | 17 | 6382.9706 | -0.0004 | 0.025 | -0.0004 | 0.25 |
| 343: | 19 | 2 | 18 | 18 | 2 | 17 | 6382.9706 | -0.0004 | 0.025 | -0.0004 | 0.25 |
| 344: | 19 | 2 | 18 | 18 | 1 | 17 | 6382.9706 | -0.0004 | 0.025 | -0.0004 | 0.25 |

| | | | | | | | | | | | | |
|---|---|---|---|---|---|---|---|---|---|---|---|---|
| 345: | 19 | 1 | 18 | 18 | 2 | 17 | 6382.9706 | -0.0004 | 0.025 | -0.0004 | 0.25 | |
| 346: | 14 | 14 | 1 | 13 | 13 | 0 | 6395.4218 | 0.0088 | 0.025 | 0.0012 | 0.50 | |
| 347: | 14 | 14 | 0 | 13 | 13 | 1 | 6395.4218 | -0.0063 | 0.025 | 0.0012 | 0.50 | |
| 348: | 14 | 14 | 0 | 13 | 13 | 0 | 6395.4216 | 0.0042 | 0.025 | 0.0010 | 0.50 | uc |
| 349: | 14 | 14 | 1 | 13 | 13 | 1 | 6395.4216 | -0.0021 | 0.025 | 0.0010 | 0.50 | uc |
| 350: | 18 | 4 | 14 | 17 | 4 | 13 | 6407.1957 | 0.0005 | 0.025 | 0.0005 | 0.25 | |
| 351: | 18 | 5 | 14 | 17 | 5 | 13 | 6407.1957 | 0.0005 | 0.025 | 0.0005 | 0.25 | |
| 352: | 18 | 5 | 14 | 17 | 4 | 13 | 6407.1957 | 0.0005 | 0.025 | 0.0005 | 0.25 | |
| 353: | 18 | 4 | 14 | 17 | 5 | 13 | 6407.1957 | 0.0005 | 0.025 | 0.0005 | 0.25 | |
| 354: | 16 | 11 | 6 | 15 | 11 | 5 | 6429.3058 | -0.0303 | 0.025 | | | |
| 355: | 18 | 5 | 13 | 17 | 5 | 12 | 6524.4206 | 0.0019 | 0.025 | 0.0020 | 0.25 | |
| 356: | 18 | 5 | 13 | 17 | 6 | 12 | 6524.4206 | 0.0019 | 0.025 | 0.0020 | 0.25 | |
| 357: | 18 | 6 | 13 | 17 | 5 | 12 | 6524.4206 | 0.0019 | 0.025 | 0.0020 | 0.25 | |
| 358: | 18 | 6 | 13 | 17 | 6 | 12 | 6524.4206 | 0.0019 | 0.025 | 0.0020 | 0.25 | |
| 359: | 16 | 10 | 6 | 15 | 10 | 5 | 6549.0699 | 0.0017 | 0.025 | | | |
| 360: | 20 | 0 | 20 | 19 | 0 | 19 | 6592.7706 | 0.0006 | 0.025 | 0.0006 | 0.25 | |
| 361: | 20 | 1 | 20 | 19 | 1 | 19 | 6592.7706 | 0.0006 | 0.025 | 0.0006 | 0.25 | |
| 362: | 20 | 1 | 20 | 19 | 0 | 19 | 6592.7706 | 0.0006 | 0.025 | 0.0006 | 0.25 | |
| 363: | 20 | 0 | 20 | 19 | 1 | 19 | 6592.7706 | 0.0006 | 0.025 | 0.0006 | 0.25 | |
| 364: | 19 | 3 | 16 | 18 | 3 | 15 | 6616.8206 | 0.0013 | 0.025 | 0.0013 | 0.25 | |
| 365: | 19 | 4 | 16 | 18 | 4 | 15 | 6616.8206 | 0.0013 | 0.025 | 0.0013 | 0.25 | |
| 366: | 19 | 4 | 16 | 18 | 3 | 15 | 6616.8206 | 0.0013 | 0.025 | 0.0013 | 0.25 | |
| 367: | 19 | 3 | 16 | 18 | 4 | 15 | 6616.8206 | 0.0013 | 0.025 | 0.0013 | 0.25 | |
| 368: | 18 | 6 | 12 | 17 | 7 | 11 | 6641.9667 | 0.0010 | 0.025 | 0.0009 | 0.25 | |
| 369: | 18 | 7 | 12 | 17 | 6 | 11 | 6641.9667 | 0.0008 | 0.025 | 0.0009 | 0.25 | |
| 370: | 18 | 6 | 12 | 17 | 6 | 11 | 6641.9667 | 0.0008 | 0.025 | 0.0009 | 0.25 | |
| 371: | 18 | 7 | 12 | 17 | 7 | 11 | 6641.9667 | 0.0010 | 0.025 | 0.0009 | 0.25 | |
| 372: | 17 | 10 | 8 | 16 | 10 | 7 | 6674.9303 | 0.0112 | 0.025 | | | |
| 373: | 15 | 12 | 3 | 14 | 11 | 4 | 6702.4727 | 0.0027 | 0.025 | | | |
| 374: | 20 | 1 | 19 | 19 | 1 | 18 | 6709.6573 | -0.0002 | 0.025 | -0.0002 | 0.25 | |
| 375: | 20 | 2 | 19 | 19 | 2 | 18 | 6709.6573 | -0.0002 | 0.025 | -0.0002 | 0.25 | |
| 376: | 20 | 2 | 19 | 19 | 1 | 18 | 6709.6573 | -0.0002 | 0.025 | -0.0002 | 0.25 | |
| 377: | 20 | 1 | 19 | 19 | 2 | 18 | 6709.6573 | -0.0002 | 0.025 | -0.0002 | 0.25 | |
| 378: | 15 | 13 | 3 | 14 | 12 | 2 | 6724.5662 | -0.0005 | 0.025 | | | |
| 379: | 15 | 13 | 2 | 14 | 12 | 3 | 6732.1593 | 0.0048 | 0.025 | | | |
| 380: | 19 | 4 | 15 | 18 | 4 | 14 | 6733.8400 | 0.0007 | 0.025 | 0.0007 | 0.25 | |
| 381: | 19 | 4 | 15 | 18 | 5 | 14 | 6733.8400 | 0.0007 | 0.025 | 0.0007 | 0.25 | |
| 382: | 19 | 5 | 15 | 18 | 4 | 14 | 6733.8400 | 0.0007 | 0.025 | 0.0007 | 0.25 | |
| 383: | 19 | 5 | 15 | 18 | 5 | 14 | 6733.8400 | 0.0007 | 0.025 | 0.0007 | 0.25 | |
| 384: | 18 | 7 | 11 | 17 | 8 | 10 | 6760.1197 | 0.0045 | 0.025 | 0.0015 | 0.25 | |
| 385: | 18 | 8 | 11 | 17 | 8 | 10 | 6760.1197 | 0.0038 | 0.025 | 0.0015 | 0.25 | |
| 386: | 18 | 7 | 11 | 17 | 7 | 10 | 6760.1197 | -0.0008 | 0.025 | 0.0015 | 0.25 | |
| 387: | 18 | 8 | 11 | 17 | 7 | 10 | 6760.1197 | -0.0015 | 0.025 | 0.0015 | 0.25 | |
| 388: | 17 | 10 | 7 | 16 | 11 | 6 | 6762.8570 | 0.0045 | 0.025 | | | |
| 389: | 15 | 14 | 2 | 14 | 13 | 1 | 6791.8634 | 0.0042 | 0.025 | | | |
| 390: | 15 | 14 | 1 | 14 | 13 | 2 | 6792.1938 | -0.0034 | 0.025 | | | |
| 391: | 20 | 2 | 18 | 19 | 2 | 17 | 6826.5592 | 0.0012 | 0.025 | 0.0013 | 0.25 | |
| 392: | 20 | 3 | 18 | 19 | 3 | 17 | 6826.5592 | 0.0012 | 0.025 | 0.0013 | 0.25 | |
| 393: | 20 | 3 | 18 | 19 | 2 | 17 | 6826.5592 | 0.0012 | 0.025 | 0.0013 | 0.25 | |
| 394: | 20 | 2 | 18 | 19 | 3 | 17 | 6826.5592 | 0.0012 | 0.025 | 0.0013 | 0.25 | |
| 395: | 15 | 15 | 1 | 14 | 14 | 0 | 6854.4206 | 0.0041 | 0.025 | 0.0011 | 0.50 | |
| 396: | 15 | 15 | 0 | 14 | 14 | 1 | 6854.4206 | -0.0019 | 0.025 | 0.0011 | 0.50 | |
| 397: | 15 | 15 | 0 | 14 | 14 | 0 | 6854.4204 | 0.0021 | 0.025 | 0.0009 | 0.50 | uc |
| 398: | 15 | 15 | 1 | 14 | 14 | 1 | 6854.4204 | -0.0004 | 0.025 | 0.0009 | 0.50 | uc |
| 399: | 20 | 3 | 17 | 19 | 3 | 16 | 6943.4910 | 0.0002 | 0.025 | 0.0003 | 0.25 | |
| 400: | 20 | 4 | 17 | 19 | 4 | 16 | 6943.4910 | 0.0002 | 0.025 | 0.0003 | 0.25 | |
| 401: | 20 | 4 | 17 | 19 | 3 | 16 | 6943.4910 | 0.0002 | 0.025 | 0.0003 | 0.25 | |
| 402: | 20 | 3 | 17 | 19 | 4 | 16 | 6943.4910 | 0.0002 | 0.025 | 0.0003 | 0.25 | |
| 403: | 19 | 6 | 13 | 18 | 6 | 12 | 6968.4199 | 0.0038 | 0.025 | 0.0039 | 0.25 | |
| 404: | 19 | 6 | 13 | 18 | 7 | 12 | 6968.4199 | 0.0038 | 0.025 | 0.0039 | 0.25 | |
| 405: | 19 | 7 | 13 | 18 | 6 | 12 | 6968.4199 | 0.0038 | 0.025 | 0.0039 | 0.25 | |
| 406: | 19 | 7 | 13 | 18 | 7 | 12 | 6968.4199 | 0.0038 | 0.025 | 0.0039 | 0.25 | |
| 407: | 18 | 9 | 9 | 17 | 9 | 8 | 7002.1242 | 0.0064 | 0.025 | | | |
| 408: | 18 | 10 | 9 | 17 | 9 | 8 | 7002.4844 | 0.0062 | 0.025 | | | |
| 409: | 17 | 11 | 6 | 16 | 11 | 5 | 7006.2995 | 0.0119 | 0.025 | | | |
| 410: | 20 | 4 | 16 | 19 | 4 | 15 | 7060.4898 | 0.0021 | 0.025 | 0.0021 | 0.25 | |
| 411: | 20 | 5 | 16 | 19 | 5 | 15 | 7060.4898 | 0.0021 | 0.025 | 0.0021 | 0.25 | |
| 412: | 20 | 5 | 16 | 19 | 4 | 15 | 7060.4898 | 0.0021 | 0.025 | 0.0021 | 0.25 | |
| 413: | 20 | 4 | 16 | 19 | 5 | 15 | 7060.4898 | 0.0021 | 0.025 | 0.0021 | 0.25 | |
| 414: | 19 | 7 | 12 | 18 | 8 | 11 | 7086.2932 | 0.0059 | 0.025 | 0.0056 | 0.25 | |

| | | | | | | | | | | | | |
|---|---|---|---|---|---|---|---|---|---|---|---|---|
| 415: | 19 | 8 | 12 | 18 | 7 | 11 | 7086.2932 | 0.0052 | 0.025 | 0.0056 | 0.25 | |
| 416: | 19 | 7 | 12 | 18 | 7 | 11 | 7086.2932 | 0.0052 | 0.025 | 0.0056 | 0.25 | |
| 417: | 19 | 8 | 12 | 18 | 8 | 11 | 7086.2932 | 0.0058 | 0.025 | 0.0056 | 0.25 | |
| 418: | 16 | 13 | 4 | 15 | 12 | 3 | 7097.5752 | 0.0013 | 0.025 | | | |
| 419: | 18 | 10 | 8 | 17 | 11 | 7 | 7115.6421 | 0.0051 | 0.025 | | | |
| 420: | 16 | 13 | 3 | 15 | 12 | 4 | 7144.5933 | 0.0032 | 0.025 | | | |
| 421: | 16 | 14 | 3 | 15 | 13 | 2 | 7185.8695 | -0.0015 | 0.025 | | | |
| 422: | 16 | 14 | 2 | 15 | 13 | 3 | 7189.5499 | -0.0003 | 0.025 | | | |
| 423: | 16 | 12 | 4 | 15 | 11 | 5 | 7198.4618 | 0.0069 | 0.025 | | | |
| 424: | 19 | 8 | 11 | 18 | 9 | 10 | 7205.0065 | 0.0166 | 0.025 | 0.0062 | 0.25 | |
| 425: | 19 | 9 | 11 | 18 | 9 | 10 | 7205.0065 | 0.0141 | 0.025 | 0.0062 | 0.25 | |
| 426: | 19 | 8 | 11 | 18 | 8 | 10 | 7205.0065 | -0.0017 | 0.025 | 0.0062 | 0.25 | |
| 427: | 19 | 9 | 11 | 18 | 8 | 10 | 7205.0065 | -0.0042 | 0.025 | 0.0062 | 0.25 | |
| 428: | 16 | 15 | 2 | 15 | 14 | 1 | 7250.9952 | 0.0062 | 0.025 | | | |
| 429: | 16 | 15 | 1 | 15 | 14 | 2 | 7251.1349 | -0.0015 | 0.025 | | | |
| 430: | 20 | 6 | 14 | 19 | 7 | 13 | 7294.9241 | 0.0061 | 0.025 | 0.0061 | 0.25 | |
| 431: | 20 | 7 | 14 | 19 | 6 | 13 | 7294.9241 | 0.0060 | 0.025 | 0.0061 | 0.25 | |
| 432: | 20 | 6 | 14 | 19 | 6 | 13 | 7294.9241 | 0.0060 | 0.025 | 0.0061 | 0.25 | |
| 433: | 20 | 7 | 14 | 19 | 7 | 13 | 7294.9241 | 0.0061 | 0.025 | 0.0061 | 0.25 | |
| 434: | 16 | 16 | 1 | 15 | 15 | 0 | 7313.4151 | 0.0070 | 0.025 | 0.0058 | 0.50 | |
| 435: | 16 | 16 | 0 | 15 | 15 | 1 | 7313.4151 | 0.0045 | 0.025 | 0.0058 | 0.50 | |
| 436: | 17 | 15 | 3 | 16 | 14 | 2 | 7646.0781 | -0.0011 | 0.025 | | | |
| 437: | 17 | 15 | 2 | 16 | 14 | 3 | 7647.8226 | 0.0019 | 0.025 | | | |
| 438: | 17 | 17 | 1 | 16 | 16 | 0 | 7772.3827 | -0.0065 | 0.025 | -0.0070 | 0.50 | |
| 439: | 17 | 17 | 0 | 16 | 16 | 1 | 7772.3827 | -0.0075 | 0.025 | -0.0070 | 0.50 | |
| 440: | 17 | 17 | 1 | 16 | 16 | 1 | 7772.3825 | -0.0074 | 0.025 | -0.0072 | 0.50 | uc |
| 441: | 17 | 17 | 0 | 16 | 16 | 0 | 7772.3825 | -0.0069 | 0.025 | -0.0072 | 0.50 | uc |
| 442: | 15 | 5 | 10 | 14 | 3 | 11 | 7866.3448 | -0.0258 | 0.025 | -0.0260 | 0.25 | |
| 443: | 15 | 6 | 10 | 14 | 4 | 11 | 7866.3448 | -0.0261 | 0.025 | -0.0260 | 0.25 | |
| 444: | 15 | 6 | 10 | 14 | 3 | 11 | 7866.3448 | -0.0261 | 0.025 | -0.0260 | 0.25 | |
| 445: | 15 | 5 | 10 | 14 | 4 | 11 | 7866.3448 | -0.0258 | 0.025 | -0.0260 | 0.25 | |
| 446: | 5 | 5 | 0 | 4 | 4 | 0 | 2260.4634 | 0.0021 | 0.025 | | | uc-lines |
| 447: | 5 | 5 | 1 | 4 | 4 | 1 | 2267.8169 | -0.0012 | 0.025 | | | |
| 448: | 6 | 4 | 2 | 5 | 3 | 2 | 2626.0601 | 0.0083 | 0.025 | | | |
| 449: | 6 | 5 | 1 | 5 | 4 | 1 | 2652.5989 | 0.0040 | 0.025 | | | |
| 450: | 6 | 5 | 2 | 5 | 4 | 2 | 2678.8178 | 0.0007 | 0.025 | | | |
| 451: | 6 | 6 | 0 | 5 | 5 | 0 | 2720.9328 | -0.0050 | 0.025 | | | |
| 452: | 6 | 6 | 1 | 5 | 5 | 1 | 2725.0950 | -0.0081 | 0.025 | | | |
| 453: | 7 | 5 | 2 | 6 | 4 | 2 | 3065.1134 | 0.0024 | 0.025 | | | |
| 454: | 7 | 4 | 3 | 6 | 3 | 3 | 3075.9602 | 0.0026 | 0.025 | | | |
| 455: | 7 | 3 | 4 | 6 | 2 | 4 | 3096.3579 | 0.0010 | 0.025 | | | |
| 456: | 7 | 4 | 4 | 6 | 3 | 4 | 3099.6346 | -0.0027 | 0.025 | | | |
| 457: | 7 | 2 | 5 | 6 | 1 | 5 | 3101.9912 | 0.0012 | 0.025 | | | |
| 458: | 7 | 3 | 5 | 6 | 2 | 5 | 3102.1222 | -0.0065 | 0.025 | | | |
| 459: | 7 | 5 | 3 | 6 | 4 | 3 | 3103.9063 | 0.0005 | 0.025 | | | |
| 460: | 7 | 6 | 1 | 6 | 5 | 1 | 3109.5835 | 0.0008 | 0.025 | | | |
| 461: | 7 | 6 | 2 | 6 | 5 | 2 | 3131.2157 | 0.0011 | 0.025 | | | |
| 462: | 7 | 7 | 0 | 6 | 6 | 0 | 3181.0454 | 0.0013 | 0.025 | | | |
| 463: | 7 | 7 | 1 | 6 | 6 | 1 | 3183.1674 | -0.0013 | 0.025 | | | |
| 464: | 8 | 5 | 3 | 7 | 4 | 3 | 3505.8373 | -0.0007 | 0.025 | | | |
| 465: | 8 | 6 | 2 | 7 | 5 | 2 | 3509.4185 | -0.0005 | 0.025 | | | |
| 466: | 8 | 4 | 4 | 7 | 3 | 4 | 3532.4168 | -0.0007 | 0.025 | | | |
| 467: | 8 | 5 | 4 | 7 | 4 | 4 | 3540.7713 | -0.0010 | 0.025 | | | |
| 468: | 8 | 3 | 5 | 7 | 2 | 5 | 3542.6089 | -0.0003 | 0.025 | | | |
| 469: | 8 | 4 | 5 | 7 | 3 | 5 | 3543.1482 | -0.0002 | 0.025 | | | |
| 470: | 8 | 2 | 6 | 7 | 1 | 6 | 3545.9832 | 0.0056 | 0.025 | -0.0024 | 0.50 | |
| 471: | 8 | 3 | 6 | 7 | 2 | 6 | 3545.9832 | -0.0105 | 0.025 | -0.0024 | 0.50 | |
| 472: | 8 | 6 | 3 | 7 | 5 | 3 | 3550.1171 | 0.0002 | 0.025 | | | |
| 473: | 8 | 7 | 1 | 7 | 6 | 1 | 3570.3311 | 0.0021 | 0.025 | | | |
| 474: | 8 | 8 | 0 | 7 | 7 | 0 | 3640.6973 | 0.0026 | 0.025 | | | |
| 475: | 8 | 8 | 1 | 7 | 7 | 1 | 3641.7064 | -0.0020 | 0.025 | | | |
| 476: | 9 | 6 | 3 | 8 | 5 | 3 | 3937.7732 | -0.0036 | 0.025 | | | |
| 477: | 9 | 7 | 2 | 8 | 6 | 2 | 3960.0257 | -0.0004 | 0.025 | | | |
| 478: | 9 | 5 | 4 | 8 | 4 | 4 | 3964.6725 | 0.0001 | 0.025 | | | |
| 479: | 9 | 6 | 4 | 8 | 5 | 4 | 3982.4120 | -0.0028 | 0.025 | | | |
| 480: | 9 | 8 | 1 | 8 | 7 | 1 | 4032.1945 | -0.0015 | 0.025 | | | |
| 481: | 9 | 8 | 2 | 8 | 7 | 2 | 4041.5759 | 0.0009 | 0.025 | | | |
| 482: | 9 | 9 | 0 | 8 | 8 | 0 | 4100.0471 | 0.0036 | 0.025 | | | |
| 483: | 9 | 9 | 1 | 8 | 8 | 1 | 4100.5034 | -0.0026 | 0.025 | | | |
| 484: | 10 | 7 | 3 | 9 | 6 | 3 | 4374.2582 | -0.0012 | 0.025 | | | |

| | | | | | | | | | | |
|---|---|---|---|---|---|---|---|---|---|---|
| 485: | 10 | 6 | 4 | 9 | 5 | 4 | 4393.2783 | 0.0006 | 0.025 | | |
| 486: | 10 | 8 | 2 | 9 | 7 | 2 | 4416.9556 | -0.0319 | 0.025 | | |
| 487: | 10 | 5 | 5 | 9 | 4 | 5 | 4419.0223 | -0.0018 | 0.025 | | |
| 488: | 10 | 6 | 5 | 9 | 5 | 5 | 4423.4327 | 0.0035 | 0.025 | | |
| 489: | 10 | 7 | 4 | 9 | 6 | 4 | 4425.2376 | 0.0012 | 0.025 | | |
| 490: | 10 | 4 | 6 | 9 | 3 | 6 | 4427.5567 | 0.0042 | 0.025 | | |
| 491: | 10 | 5 | 6 | 9 | 4 | 6 | 4427.8383 | 0.0171 | 0.025 | | |
| 492: | 10 | 3 | 7 | 9 | 2 | 7 | 4431.2899 | 0.0047 | 0.025 | 0.0002 | 0.50 |
| 493: | 10 | 4 | 7 | 9 | 3 | 7 | 4431.2899 | -0.0042 | 0.025 | 0.0002 | 0.50 |
| 494: | 10 | 2 | 8 | 9 | 1 | 8 | 4433.5615 | 0.0038 | 0.025 | 0.0037 | 0.50 |
| 495: | 10 | 3 | 8 | 9 | 2 | 8 | 4433.5615 | 0.0036 | 0.025 | 0.0037 | 0.50 |
| 496: | 10 | 8 | 3 | 9 | 7 | 3 | 4449.2364 | -0.0127 | 0.025 | | |
| 497: | 10 | 9 | 2 | 9 | 8 | 2 | 4498.8018 | -0.0019 | 0.025 | | |
| 498: | 10 | 10 | 0 | 9 | 9 | 0 | 4559.2313 | 0.0050 | 0.025 | | |
| 499: | 10 | 10 | 1 | 9 | 9 | 1 | 4559.4347 | 0.0040 | 0.025 | | |
| 500: | 11 | 8 | 3 | 10 | 7 | 3 | 4816.8089 | -0.0008 | 0.025 | | |
| 501: | 11 | 6 | 5 | 10 | 5 | 5 | 4853.2449 | -0.0026 | 0.025 | | |
| 502: | 11 | 7 | 5 | 10 | 6 | 5 | 4863.2755 | 0.0036 | 0.025 | | |
| 503: | 11 | 5 | 6 | 10 | 4 | 6 | 4866.8644 | 0.0014 | 0.025 | | |
| 504: | 11 | 8 | 4 | 10 | 7 | 4 | 4869.8537 | -0.0076 | 0.025 | | |
| 505: | 11 | 3 | 8 | 10 | 2 | 8 | 4875.2241 | 0.0021 | 0.025 | 0.0016 | 0.50 |
| 506: | 11 | 4 | 8 | 10 | 3 | 8 | 4875.2241 | 0.0011 | 0.025 | 0.0016 | 0.50 |
| 507: | 11 | 2 | 9 | 10 | 1 | 9 | 4877.2760 | 0.0033 | 0.025 | 0.0033 | 0.50 |
| 508: | 11 | 3 | 9 | 10 | 2 | 9 | 4877.2760 | 0.0033 | 0.025 | 0.0033 | 0.50 |
| 509: | 11 | 9 | 2 | 10 | 8 | 2 | 4878.5326 | 0.0026 | 0.025 | | |
| 510: | 11 | 9 | 3 | 10 | 8 | 3 | 4902.2542 | 0.0000 | 0.025 | | |
| 511: | 11 | 10 | 1 | 10 | 9 | 1 | 4954.1561 | 0.0094 | 0.025 | | |
| 512: | 11 | 10 | 2 | 10 | 9 | 2 | 4956.8507 | 0.0011 | 0.025 | | |
| 513: | 12 | 8 | 4 | 11 | 7 | 4 | 5249.3488 | -0.0081 | 0.025 | | |
| 514: | 12 | 9 | 3 | 11 | 8 | 3 | 5266.4047 | 0.0021 | 0.025 | | |
| 515: | 12 | 7 | 5 | 11 | 6 | 5 | 5283.6308 | 0.0013 | 0.025 | | |
| 516: | 12 | 8 | 5 | 11 | 7 | 5 | 5303.6053 | 0.0000 | 0.025 | | |
| 517: | 12 | 6 | 6 | 11 | 5 | 6 | 5304.7106 | 0.0021 | 0.025 | | |
| 518: | 12 | 4 | 8 | 11 | 3 | 8 | 5316.3882 | 0.0018 | 0.025 | -0.0004 | 0.50 |
| 519: | 12 | 5 | 8 | 11 | 4 | 8 | 5316.3882 | -0.0027 | 0.025 | -0.0004 | 0.50 |
| 520: | 12 | 9 | 4 | 11 | 8 | 4 | 5316.7478 | -0.0046 | 0.025 | | |
| 521: | 12 | 2 | 10 | 11 | 1 | 10 | 5320.9577 | -0.0019 | 0.025 | -0.0019 | 0.50 |
| 522: | 12 | 3 | 10 | 11 | 2 | 10 | 5320.9577 | -0.0019 | 0.025 | -0.0019 | 0.50 |
| 523: | 12 | 10 | 2 | 11 | 9 | 2 | 5341.7855 | -0.0056 | 0.025 | | |
| 524: | 12 | 10 | 3 | 11 | 9 | 3 | 5357.2223 | -0.0081 | 0.025 | | |
| 525: | 12 | 11 | 1 | 11 | 10 | 1 | 5414.0537 | 0.0016 | 0.025 | | |
| 526: | 12 | 11 | 2 | 11 | 10 | 2 | 5415.3884 | 0.0038 | 0.025 | | |
| 527: | 13 | 9 | 4 | 12 | 8 | 4 | 5683.2017 | -0.0117 | 0.025 | | |
| 528: | 13 | 8 | 5 | 12 | 7 | 5 | 5710.1944 | 0.0011 | 0.025 | | |
| 529: | 13 | 7 | 6 | 12 | 6 | 6 | 5740.4519 | 0.0006 | 0.025 | | |
| 530: | 13 | 9 | 5 | 12 | 8 | 5 | 5745.0963 | 0.0046 | 0.025 | | |
| 531: | 13 | 6 | 7 | 12 | 5 | 7 | 5751.6033 | -0.0021 | 0.025 | | |
| 532: | 13 | 7 | 7 | 12 | 6 | 7 | 5752.0005 | 0.0004 | 0.025 | | |
| 533: | 13 | 5 | 8 | 12 | 4 | 8 | 5756.9950 | 0.0061 | 0.025 | -0.0029 | 0.50 |
| 534: | 13 | 6 | 8 | 12 | 5 | 8 | 5756.9950 | -0.0119 | 0.025 | -0.0029 | 0.50 |
| 535: | 13 | 3 | 10 | 12 | 2 | 10 | 5762.8464 | -0.0041 | 0.025 | | |
| 536: | 13 | 10 | 4 | 12 | 9 | 4 | 5766.1507 | 0.0098 | 0.025 | | |
| 537: | 13 | 11 | 2 | 12 | 10 | 2 | 5804.6366 | -0.0052 | 0.025 | | |
| 538: | 13 | 11 | 3 | 12 | 10 | 3 | 5813.7501 | -0.0006 | 0.025 | | |
| 539: | 13 | 12 | 1 | 12 | 11 | 1 | 5873.5559 | 0.0049 | 0.025 | | |
| 540: | 13 | 12 | 2 | 12 | 11 | 2 | 5874.1816 | -0.0025 | 0.025 | | |
| 541: | 13 | 13 | 0 | 12 | 12 | 0 | 5936.4051 | 0.0003 | 0.025 | -0.0074 | 0.50 |
| 542: | 13 | 13 | 1 | 12 | 12 | 1 | 5936.4051 | -0.0152 | 0.025 | -0.0074 | 0.50 |
| 543: | 14 | 10 | 4 | 13 | 9 | 4 | 6123.7692 | 0.0042 | 0.025 | | |
| 544: | 14 | 9 | 5 | 13 | 8 | 5 | 6134.8485 | 0.0076 | 0.025 | | |
| 545: | 14 | 9 | 6 | 13 | 8 | 6 | 6184.4200 | -0.0052 | 0.025 | | |
| 546: | 14 | 11 | 3 | 13 | 10 | 3 | 6185.2294 | 0.0019 | 0.025 | | |
| 547: | 14 | 10 | 5 | 13 | 9 | 5 | 6188.3726 | -0.0028 | 0.025 | | |
| 548: | 14 | 4 | 10 | 13 | 3 | 10 | 6204.3982 | 0.0301 | 0.025 | 0.0301 | 0.50 |
| 549: | 14 | 5 | 10 | 13 | 4 | 10 | 6204.3982 | 0.0300 | 0.025 | 0.0301 | 0.50 |
| 550: | 14 | 3 | 11 | 13 | 2 | 11 | 6206.5629 | -0.0288 | 0.025 | -0.0289 | 0.50 |
| 551: | 14 | 4 | 11 | 13 | 3 | 11 | 6206.5629 | -0.0288 | 0.025 | -0.0289 | 0.50 |
| 552: | 14 | 2 | 12 | 13 | 1 | 12 | 6208.2839 | 0.0080 | 0.025 | 0.0080 | 0.50 |
| 553: | 14 | 3 | 12 | 13 | 2 | 12 | 6208.2839 | 0.0080 | 0.025 | 0.0080 | 0.50 |
| 554: | 14 | 1 | 13 | 13 | 0 | 13 | 6209.5954 | 0.0089 | 0.025 | 0.0089 | 0.50 |

```
 555: 14  2 13   13  1 13                     6209.5954   0.0089  0.025   0.0089 0.50
 556: 14 11  4   13 10  4                     6218.0084   0.0061  0.025
 557: 14 12  2   13 11  2                     6266.3598  -0.0006  0.025
 558: 14 12  3   13 11  3                     6271.3665   0.0061  0.025
 559: 14 13  1   13 12  1                     6332.8148  -0.0050  0.025
 560: 14 13  2   13 12  2                     6333.1110  -0.0013  0.025
 561: 15  9  7   14  8  7                     6629.1002   0.0164  0.025
 562: 15  7  8   14  6  8                     6636.1776   0.0006  0.025
 563: 15  8  8   14  7  8                     6636.3695   0.0076  0.025
 564: 15  6  9   14  5  9                     6641.7082   0.0049  0.025   0.0007 0.50
 565: 15  7  9   14  6  9                     6641.7082  -0.0036  0.025   0.0007 0.50
 566: 15  5 10   14  4 10                     6645.4767  -0.0011  0.025  -0.0013 0.50
 567: 15  6 10   14  5 10                     6645.4767  -0.0014  0.025  -0.0013 0.50
 568: 15  4 11   14  3 11                     6648.2274   0.0019  0.025   0.0019 0.50
 569: 15  5 11   14  4 11                     6648.2274   0.0019  0.025   0.0019 0.50
 570: 15 12  3   14 11  3                     6649.7984  -0.0031  0.025
 571: 15 13  3   14 12  3                     6729.6571  -0.0056  0.025
---------------------------------------------------------------------------------

PARAMETERS IN FIT:

        10000            A  /MHz         229.5257(3)                        1
        20000            B  /MHz         215.0008(3)                        2
        30000            C  /MHz         163.3655(2)                        3
          200           DJ  /kHz           0.0084(8)                        4
         1100          DJK  /kHz           0.068(4)                         5
         2000           DK  /kHz          -0.055(4)                         6
        40100           d1  /kHz          -0.0028(3)                        7
        50000           d2  /kHz          -0.0031(2)                        8

 MICROWAVE AVG =        0.000153 MHz, IR AVG =        0.00000
 MICROWAVE RMS =        0.009707 MHz, IR RMS =        0.00000
 END OF ITERATION  1 OLD, NEW RMS ERROR=        0.38828        0.38828

  distinct frequency lines in fit:   348
       distinct parameters of fit:     8

 for standard errors previous errors are multiplied by:   0.392821

 PARAMETERS IN FIT WITH STANDARD ERRORS ON THOSE THAT ARE FITTED:

        10000            A  /MHz         229.5257(1)                        1
        20000            B  /MHz         215.0008(1)                        2
        30000            C  /MHz         163.3655(1)                        3
          200           DJ  /kHz           0.0084(3)                        4
         1100          DJK  /kHz           0.068(1)                         5
         2000           DK  /kHz          -0.055(1)                         6
        40100           d1  /kHz          -0.0028(1)                        7
        50000           d2  /kHz          -0.00318(8)                       8

 CORRELATION COEFFICIENTS, C.ij:

           A        B        C        -DJ      -DJK     -DK      d1       d2

A            1.0000
B           -0.2947   1.0000
C           -0.1721   0.1226   1.0000
-DJ          0.4883  -0.7297  -0.3860   1.0000
-DJK        -0.5893   0.4463   0.1460  -0.8613   1.0000
-DK          0.4190  -0.3126  -0.0575   0.7486  -0.9621   1.0000
d1           0.2851  -0.7901   0.2251   0.7082  -0.5885   0.4965   1.0000
d2          -0.2451  -0.0791   0.2140  -0.3525   0.5637  -0.6087  -0.0006   1.0000

 Mean value of |C.ij|, i.ne.j =   0.4248
 Mean value of  C.ij,  i.ne.j =  -0.0774


 Worst fitting lines (obs-calc/error):

    30:    5.4        326:   -1.9         22:    1.4        327:   -1.3
```

```
  486:   -1.3         354:   -1.2         548:    1.2         550:   -1.2
  442:   -1.0         184:   -0.7         491:    0.7          35:    0.7
  561:    0.7           9:    0.6         496:   -0.5           5:   -0.5
  191:   -0.5         409:    0.5         527:   -0.5         372:    0.4
  241:    0.4         536:    0.4          34:   -0.4         162:    0.4
  511:    0.4         554:    0.4         448:    0.3         513:   -0.3
  452:   -0.3         524:   -0.3         552:    0.3         504:   -0.3
  544:    0.3         563:    0.3         541:   -0.3         440:   -0.3
    7:    0.3         438:   -0.3         138:   -0.3         423:    0.3
  458:   -0.3         127:   -0.3         407:    0.3         428:    0.2
  408:    0.2         424:    0.2         430:    0.2         556:    0.2
  558:    0.2         434:    0.2
                                       _______________________________________
_____________________________________/ SPFIT output reformatted with PIFORM
```
